# Fine Tuning of the Redox‐Active Diguanidine Ligand in Cationic Cobalt Complexes for Electronic Structure Control

**DOI:** 10.1002/chem.202501382

**Published:** 2025-08-02

**Authors:** Johanna Osterbrink, Meret Kaliske, Maximilian Schulz, Hanna Koepcke, Olaf Hübner, Elisabeth Kaifer, Hans‐Jörg Himmel

**Affiliations:** ^1^ Inorganic Chemistry Ruprecht‐Karls Universität Heidelberg Im Neuenheimer Feld 270 69120 Heidelberg Germany

**Keywords:** bistability, cobalt, electronic structure, guanidine, redox‐active ligand

## Abstract

In this work we show that the redox chemistry of hexacoordinated cobalt complexes with redox‐active diguanidine ligands could be tuned by modifying the diguanidine and/or the co‐ligands. In this way, one‐electron oxidation could be directed to be either ligand‐ or metal‐centered, leading to high‐spin Co^II^ complexes with an oxidized, radical‐monocationic diguanidine ligand (ligand‐centered oxidation) or low‐spin Co^III^ complexes with a reduced, neutral diguanidine ligand. Further fine‐tuning of the redox‐active diguanidine ligand leads to a harmonization of the energies of both redox isomers. Consequently, ligand‐ as well as metal‐centered one‐electron oxidation is observed, resulting in a mixture of both redox isomers. Quantum‐chemical calculations confirm the energetic proximity of the two redox isomers in this bistable system. The results of this work contribute to the development of a directed approach toward the design of bistable cobalt‐guanidine complexes and their possible applications in spin‐switching devices.

## Graphical abstract

Oxidation in Co complexes with redox‐active diguanidine ligands is either ligand‐ or metal‐centered, leading to paramagnetic high‐spin Co^II^ complexes with radical monocationic diguanidine ligands or low‐spin Co^III^ redox isomers with neutral diguanidine ligands. Careful ligand design leads to the energetic harmonization of the two redox isomers and realization of one‐electron oxidation processes that are both metal‐ and ligand‐centered.

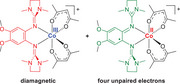

## 1 Introduction

**Figure 1:**
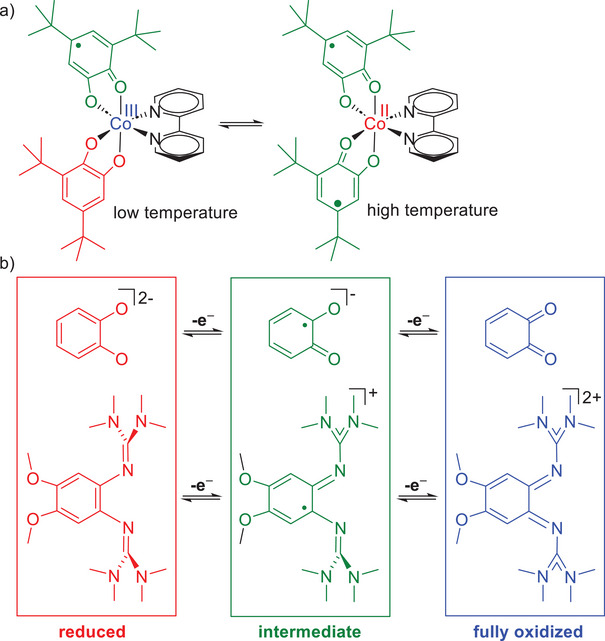
a) Redox isomerism (valence tautomerism) of a cobalt complex with two redox‐active oxolene ligands. b) Comparison between the redox states and their charge regime of oxolene and diguanidine ligands.

The possibility to trigger intramolecular metal‐ligand electron transfer by temperature, light, or pressure renders cobalt complexes with redox‐active ligands attractive for several applications in catalysis and materials science.^[^ ^1^, ^2^, ^3^, ^4^, ^5^, ^6^, ^7^, ^8^, ^9^, ^10^, ^11^, ^12^, ^13^, ^14^, ^15^, ^16^, ^17^ ^]^. The shift of an electron from a high‐spin Co^II^ atom with three unpaired electrons to a redox‐active acceptor ligand is often accompanied by spin‐crossover to give a low‐spin Co^III^ atom, massively changing the magnetic and optical properties. An archetypical example, reported already in 1980 by Pierpont et al., is sketched in Figure 1a.^[^ ^18^ ^]^ In this example, the Co atom is hexacoordinated by two dioxolene ligands. Two redox isomers are in a thermal equilibrium: a Co^III^ complex with partially oxidized ligands (one unpaired electron on the two dioxolene ligands) and a Co^II^ complex with two 3,5‐ditertbutyl‐semiquinonato ligands. The low‐spin Co^III^ redox isomer is slightly favored by enthalpy, but the high‐spin Co^II^ redox isomer exhibits the higher entropy (due to vibrational (weaker bonds) and (less important) spin entropy contributions). Thereby, it is possible to interconvert the two redox isomers quantitatively by temperature. For such an equilibrium between redox isomers, Pierpont has coined the term “valence tautomerism.”^[^ ^9^ ^]^

**Figure 2:**
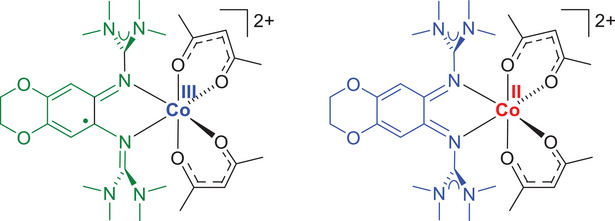
A dicationic cobalt complex for which the low‐spin Co^III^ redox isomer with radical monocationic diguanidine ligand is favored in dichloromethane, and the corresponding high‐spin Co^II^ electromer with fully oxidized dicationic diguanidine ligand is favored in acetonitrile and tetrahydrofurane.

For the further development of this field, it is essential to increase the classes of redox‐active ligands that could be integrated in cobalt complexes showing redox isomerism. Our group developed redox‐active guanidines as a novel class of redox‐active ligands, with a charge regime (neutral in the reduced and dicationic in the fully oxidized redox state) complementary to that of dioxolene‐type ligands (Figure 1b). Recently, we synthesized the first hexacoordinated cobalt complexes with a redox‐active diguanidine ligand and two acetylacetonate (acac), trifluoroacetylacetonate (tfac) or hexafluoroacetylacetonate (hfac) coligands.^[^ ^19^, ^20^ ^]^ Oxidation of the neutral high‐spin Co^II^ complexes with neutral, reduced diguanidine ligand is either metal‐ or ligand‐centered. For the diguanidines with relatively low redox potential used in our previous work, the choice of co‐ligands determined the redox behavior. The complexes with acac co‐ligands were oxidized at the metal to give diamagnetic low‐spin Co^III^ complexes with a neutral diguanidine ligand. On the other hand, oxidation of the complexes with hfac co‐ligands was ligand‐centered, leading to high‐spin Co^II^ complexes with a radical‐monocationic, oxidized diguanidine ligand. We also reported a first dicationic cobalt‐guanidine system (see Figure 2) with two tfac co‐ligands that varies its electronic structure with the solvent.^[^ ^21^ ^]^ In Figure 2, the two possible redox isomers of this dicationic complex are sketched. The structure on the left is a low‐spin Co^III^ complex with a radical‐monocationic diguanidine ligand, and the structure on the right is a Co^II^ complex with a fully oxidized, dicationic diguanidine ligand. The Co^III^ redox isomer is favored in dichloromethane solution, and the Co^II^ redox isomer in acetonitrile or tetrahydrofurane.

**Figure 3:**
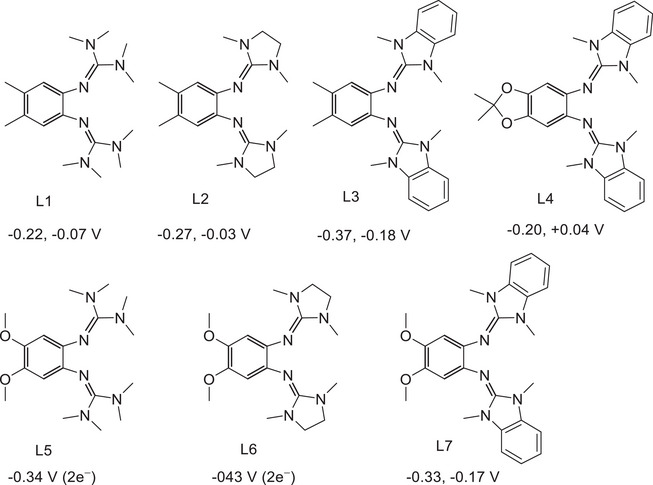
Molecular structures of the redox‐active diguanidine ligands used in this work. The redox potentials (*E*_1/2_ values vs. Fc^+^/Fc) from CV measurements in CH_2_Cl_2_ solutions are given underneath each structure.

In this work, we vary the diguanidine ligand as well as the co‐ligands (acac, tfac, or hfac) in monocationic cobalt complexes. The aim is to realize monocationic cobalt‐diguanidine systems for which the relative energies of the high‐spin Co^II^ redox isomers with radical monocationic diguanidine ligand (four unpaired electrons) are close to those of the low‐spin Co^III^ redox isomers with neutral diguanidine ligand (no unpaired electrons), thereby opening up the possibility for stimulated interconversions of this bistable systems. Figure 3 illustrates the structures of the seven diguanidine ligands applied in this work. The synthesis and characterization of the free ligands were reported previously (ref. [22] for ligands L1‐L4, L6, and L7; ref. [23] for ligand L5). In cyclic voltammetry (CV) measurements, ligands L1‐L4 and L7 show two reversible one‐electron redox steps, and L5 and L6 only one two‐electron redox step. The ligands vary in their first redox potential (values given underneath each structure) between −0.43 V for L6 and −0.20 V for L4. Obviously, the redox potential of the diguanidine ligand is one of the parameters determining the favored electron distribution in the cobalt complexes. Generally, the redox potentials of GFAs increase upon change from the 1,4‐bis(*N*,*N*’‐dimethyl)ethylene‐guanidino group to the tetramethylguanidino group or the phenylene‐1,4‐bis(*N*,*N*’‐dimethyl)guanidino group.^[^ ^24^ ^]^ Additional substituents were added to the benzene backbone. These substituents fulfill two tasks. They prohibit undesired nucleophilic attack upon oxidation (see the discussion in ref. [25]). Moreover, they allow a fine‐tuning of the redox potential. The introduction of an alkoxy group in the ligand backbone decreases the potential due to the +M effect that dominates over the −I effect. Generally, the +I effect arising from the introduction of methyl groups is weaker and leads to higher *E*_1/2_ values; an exception is ligand L3, which exhibits a remarkably low *E*_1/2_ value. A relatively high redox potential disfavors ligand oxidation, thereby stabilizing the Co^III^ redox isomer with neutral diguanidine ligand. On the other hand, a low‐potential diguanidine ligand stabilizes the Co^II^ redox isomer with an oxidized, radical‐monocationic diguanidine ligand. However, the redox potential is obviously not the only important parameter. Previous work on copper complexes with redox‐active guanidine ligands demonstrated that other parameters, such as the solvent effect, could dominate, especially regarding the entropy change upon redox isomerism.^[^ ^22^, ^26^ ^]^

## 2 Results and discussion

### 2.1 Synthesis of Neutral Co^II^ Complexes

**Scheme 1:**
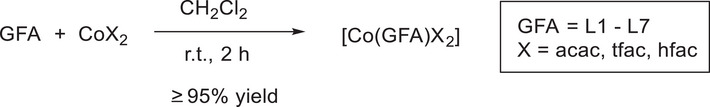
Synthesis of the neutral high‐spin Co^II^ complexes with redox‐active GFA ligands.

First, we synthesized the neutral Co^II^ complexes from the reaction between CoX_2_ (X = acac, tfac, or hfac) and one of the diguanidines in dichloromethane solutions at room temperature (Scheme 1). In this way, 21 different complexes were obtained. The synthesis protocols and analytical data are included in the Supporting Information for this article.

**Figure 4:**
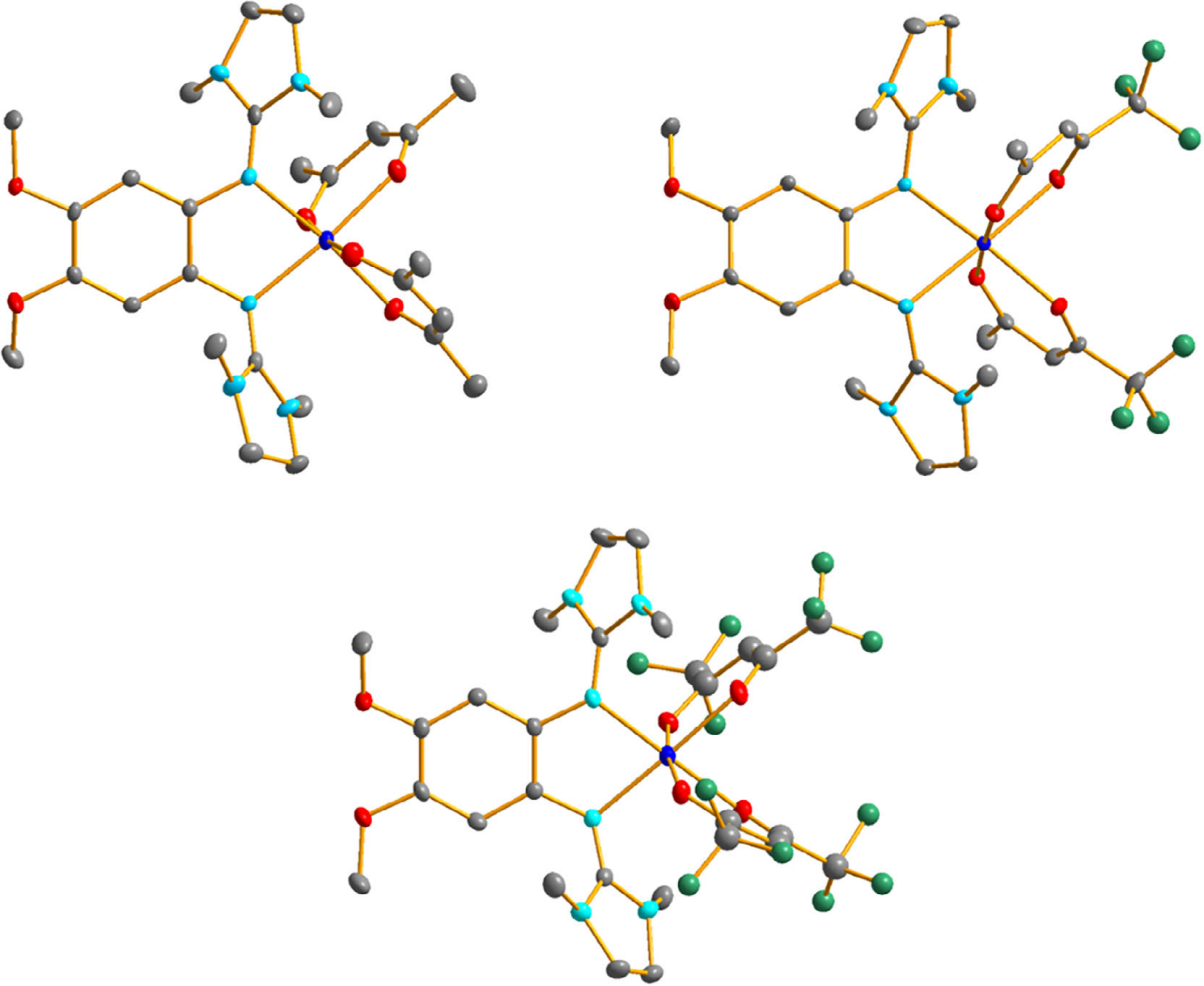
Illustration of the structures of the complexes [Co(acac)_2_(L6)], [Co(tfac)_2_(L6)] and [Co(hfac)_2_(L6)] in the solid state. Displacement ellipsoids drawn at the 50% probability level. Hydrogen atoms omitted. Color code: Co dark blue, O red, N light blue, C grey, F green.

Of the 21 new complexes, 16 compounds crystallized and were structurally characterized by SC‐XRD (see Supporting Information for details). As an example, Figure 4 illustrates the structures of the three compounds [Co(acac)_2_(L6)], [Co(tfac)_2_(L6)], and [Co(hfac)_2_(L6)], and Table 1 contains selected structural parameters. The Co‐N and Co‐O bond lengths (all values above 2 Å) clearly indicate the presence of high‐spin Co^II^ atoms in all three complexes, being stabilized by the weak field evoked by the π‐donor diguanidine ligands.^[^ ^27^, ^28^ ^]^ The structural parameters of the diguanidine ligand confirm the presence of the reduced, neutral redox states with an intact aromatic benzene ring. The CN_3_ units of the guanidino groups are highly tilted with respect to the C_6_ aromatic plane. This was generally found to be the energetically favored conformation both in complexes and in the free guanidine ligands.^[^ ^26^, ^29^ ^]^ Tables with selected bond lengths of the other complexes are included in the Supporting Information. There are no significant differences in the bonding parameters. All complexes are high‐spin Co^II^ complexes, in line with the weak ligand field evoked by the diguanidine ligand. In all cases, coordination leads to a significant elongation of the N═C double bonds of the diguanidine ligand.

**Table 1:** Selected bond lengths (in Å) for the three complexes [Co(acac)_2_(L6)], [Co(tfac)_2_(L6)], and [Co(hfac)_2_(L6)] in the solid state. Structural parameters of the other 13 characterized neutral complexes are included in the Supporting Information.

bond	[Co(acac)_2_(L6)]	[Co(tfac)_2_(L6)]	[Co(hfac)_2_(L6)]	
Co1‐O3	2.071(2)	2.064(2)	2.0914(18)	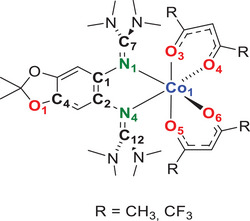
Co1‐O4	2.078(2)	2.111(2)	2.0862(18)	
Co1‐O5	2.073(2)	2.086(2)	2.0919(18)	
Co1‐O6	2.091(2)	2.081(2)	2.1096(18)	
Co1‐N1	2.206(2)	2.201(3)	2.081(2)	
Co1‐N4	2.106(2)	2.103(2)	2.124(2)	
N1‐C1	1.405(4)	1.409(4)	1.406(3)	
N4‐C2	1.410(3)	1.409(4)	1.407(3)	
N1‐C7	1.313(4)	1.311(4)	1.324(3)	
N4‐C12	1.320(4)	1.326(4)	1.325(3)	
C1‐C2	1.404(4)	1.392(4)	1.403(4)	
C4‐O1	1.378(3)	1.377(4)	1.388(3)	

The UV‐vis spectra of the different complexes (see Supporting Information) readily explain the color of the complexes. The spectra show tails of the strong UV absorption that reach far into the visible region, up to wavelengths between 600 and 750 nm. Superimposed on these tails, there are additional weak absorptions in the visual region. The calculation of the UV‐vis spectrum (time‐dependent density functional theory (TDDFT)) of [Co(acac)_2_(L7)] (^4^A electronic term) yields very weak absorptions in the near IR and visible range at 789, 478, 466, and 434 nm and a strong absorption at 419 nm. These are in line with the observation of a tail of the experimental spectrum, extending up to 750 nm (with superimposed, weak absorptions at 465 and 568 nm), and a pronounced shoulder at 370 nm. For the absorption calculated at 789 nm, the calculations indicate contributions of d‐d transitions, whereas the absorption at 419 nm mainly corresponds to a shift of the charge from the central aromatic ring of the ligand L7 to the peripheral aromatic rings.

### 2.2 CV Measurements

**Figure 5:**
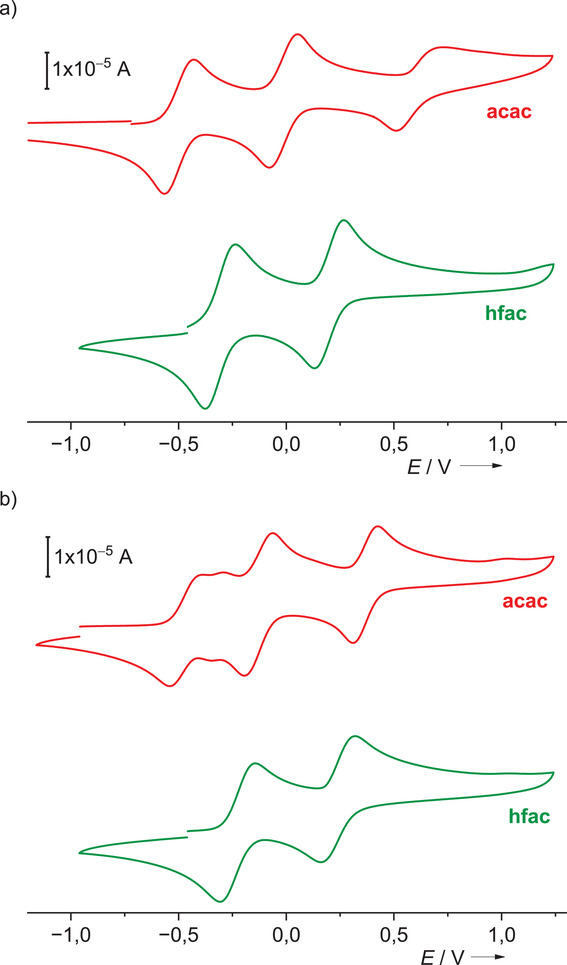
Comparison between the CV curves for a) [Co(acac)_2_(L4)] and [Co(hfac)_2_(L4)] and b) [Co(acac)_2_(L7)] and [Co(hfac)_2_(L7)] in CH_2_Cl_2_ (Ag/AgCl reference electrode, 0.1 M N(*n*Bu)_4_(PF_6_) as supporting electrolyte, scan rate 30 mV s^−1^). Potentials given vs. the Fc^+^/Fc reference redox couple.

Figure 5 reproduces the CV curves for [Co(acac)_2_(L4)] and [Co(hfac)_2_(L4)] as well as [Co(acac)_2_(L7)] and [Co(hfac)_2_(L7)]. The CV curves of all 21 neutral complexes, dissolved in CH_2_Cl_2_, can be found in the Supporting Information. They show that the neutral complexes could be oxidized reversibly at relatively low potential (all *E*_1/2_ and *E*_ox_ values are given with respect to the reference redox couple ferrocenium/ferrocene (Fc^+^/Fc)). A maximum number of three electrons could be removed, one from the cobalt atom (Co^II^ → Co^III^) and two from the diguanidine ligand. Therefore, a maximum number of three redox waves is expected to show. The red curve in Figure 5a shows the CV curve for [Co(acac)_2_(L4)]. Here, the maximal number of three redox waves is visible; they are located at *E*_1/2_ = ‐0.36, 0.09, and 0.68 V. It should be noted that the third oxidation wave is slightly broader and the corresponding reduction wave weaker, pointing to low solubility upon oxidation to the tricationic complex (see also discussion below). We will see in the following that the first oxidation event is metal‐centered for this complex. For [Co(hfac)_2_(L4)] (green curve in Figure 5a), two waves appear, belonging to two reversible redox processes at *E*_1/2_ = ‐0.31 V and 0.20 V. We will show in the following that the first one‐electron oxidation is ligand‐centered. The hfac coligands shift the metal‐centered redox event (Co^II^ → Co^III^) to higher values.

Figure 5b compares the CV curves for the complexes [Co(acac)_2_(L7)] (red curve) and [Co(hfac)_2_(L7)] (green curve). The curve recorded for [Co(hfac)_2_(L7)] looks similar to the curve for [Co(hfac)_2_(L4)]. Two one‐electron redox steps are visible, with *E*_1/2_ values of ‐0.23 and 0.24 V. At least the first of these redox events is again due to oxidation of the diguanidine ligand, as shown below. For all complexes with hfac coligands, only two (ligand‐centered) reversible redox events are observed (see the CV curves included in the Supporting Information). The highest potential for the first redox process of all complexes with hfac coligands is observed for [Co(hfac)_2_(L1)] (*E*_1/2_ = ‐0.04 V), and the lowest one for [Co(hfac)_2_(L6)] (*E*_1/2_ = ‐0.33 V). This is roughly in line with the trend in the redox potentials of the free diguanidine ligands; L6 exhibits the lowest *E*_1/2_ value of all free ligands (‐0.43 V), and L1 exhibits one of the highest *E*_1/2_ values (‐0.22 V).

On the other hand, the curves for the complexes [Co(acac)_2_(L4)] and [Co(acac)_2_(L7)] are significantly different. Both curves for the complexes with acac coligands show three waves, but the first wave is much broader and even split into two contributions for the complex with L7. This result points to the simultaneous presence of ligand‐ and metal‐centered contributions to the first oxidation event. Interestingly, the CV curve recorded for [Co(acac)_2_(L6)] looks similar; a broad first oxidation wave appears. The further analysis indeed confirms that for both [Co(acac)_2_(L6)] and [Co(acac)_2_(L7)], the first one‐electron oxidation event is ligand‐ as well as metal‐centered.

A comparison of the first oxidation wave for all complexes with acac coligands indicates that the *E*_ox_ value for Co^II^ → Co^III^ is only slightly affected by variation of the guanidino group (e.g., *E*_ox_ = ‐0.47 V for [Co(acac)_2_(L1)] and ‐0.44 V for [Co(acac)_2_(L2)] for ligands with two methyl groups in the backbone, and *E*_ox_ = ‐0.29 V for [Co(acac)_2_(L4)] as well as [Co(acac)_2_(L5)] for ligands with two alkoxy groups in the backbone). In the case of [Co(acac)_2_(L7)], the first oxidation wave consists of two contributions with maxima at ‐0.40 and ‐0.29 V (Figure 5b). Since L7 also exhibits two alkoxy groups in the ligand backbone, the contribution with the maximum at ‐0.29 V is tentatively assigned to metal‐centered oxidation (as in the complexes with L4 and L5), and the maximum at ‐0.40 V to ligand‐centered oxidation. For [Co(acac)_2_(L6)], the broad oxidation wave covers approximately the region from ‐0.40 to ‐0.31 V, and is likely to consist also of these two contributions. The low sensitivity of the *E*_ox_ values regarding modifications at the guanidino groups hampered the assignment of Lever E_L_ values to the diguanidine ligands.^[^ ^30^ ^]^

### 2.3 Synthesis and Structural Characterization of the Monocationic Complexes

**Scheme 2:**
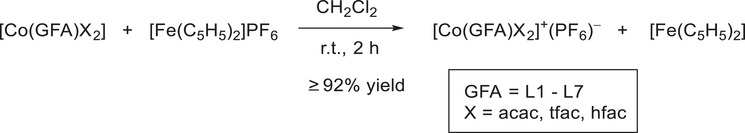
Synthesis of the cationic Co complexes with redox‐active GFA ligands.

Having studied the neutral complexes, we next chemically oxidized the complexes using one equivalent of FcPF_6_ as an oxidant. All complexes exhibit negative *E*_1/2_ values for the first one‐electron oxidation step (given vs. the reference redox couple ferrocenium/ferrocene), the only exception being [Co(tfac)_2_(L1)], for which an *E*_1/2_ value very close to zero (*E*_1/2_ = 0.03 V) was found. Therefore, all complexes could be oxidized with FcPF_6_ to the monocation (Scheme 2). The monocationic complexes were obtained as hexafluorophosphate salts in yields of 92% and more.

Of the 21 cationic complexes synthesized by reaction with ferrocenium hexafluorophosphate, we were able to grow crystals suitable for a structural characterization with SC‐XRD for ten different cations. Please note that the cations [Co(hfac)_2_(L1)]^+^ and [Co(hfac)_2_(L2)]^+^ crystallized in tiny amounts directly from the reaction mixture with [Co(hfac)_3_]^−^ counterions instead of PF_6_^−^.

**Figure 6:**
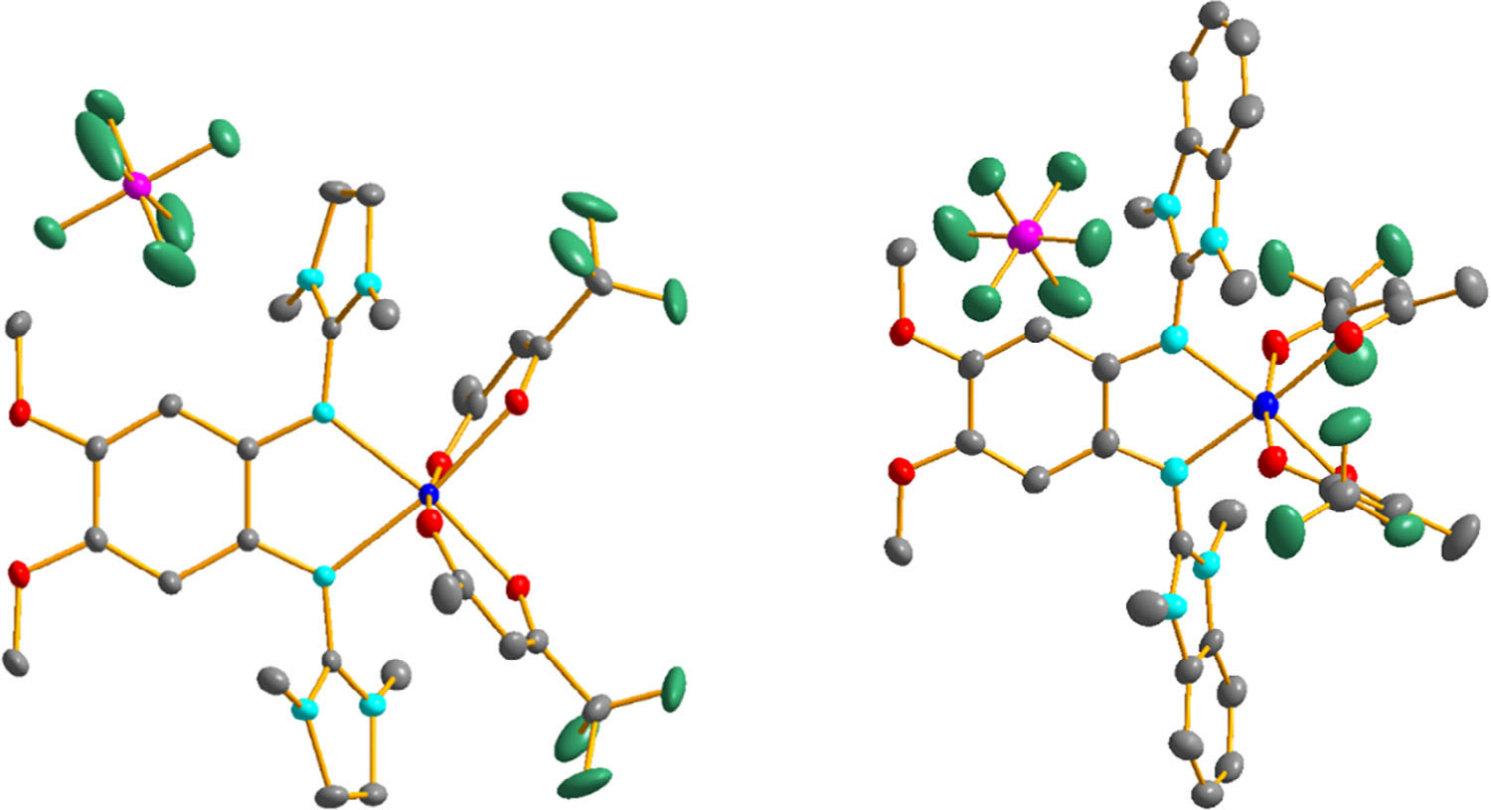
Illustration of the solid‐state structures of the two compounds [Co(tfac)_2_(L6)]PF_6_ and [Co(tfac)_2_(L7)]PF_6_. Displacement ellipsoids drawn at the 50% probability level. Hydrogen atoms omitted. Color code: Co dark blue, O red, N light blue, C grey, P pink, F green.

**Figure 7:**
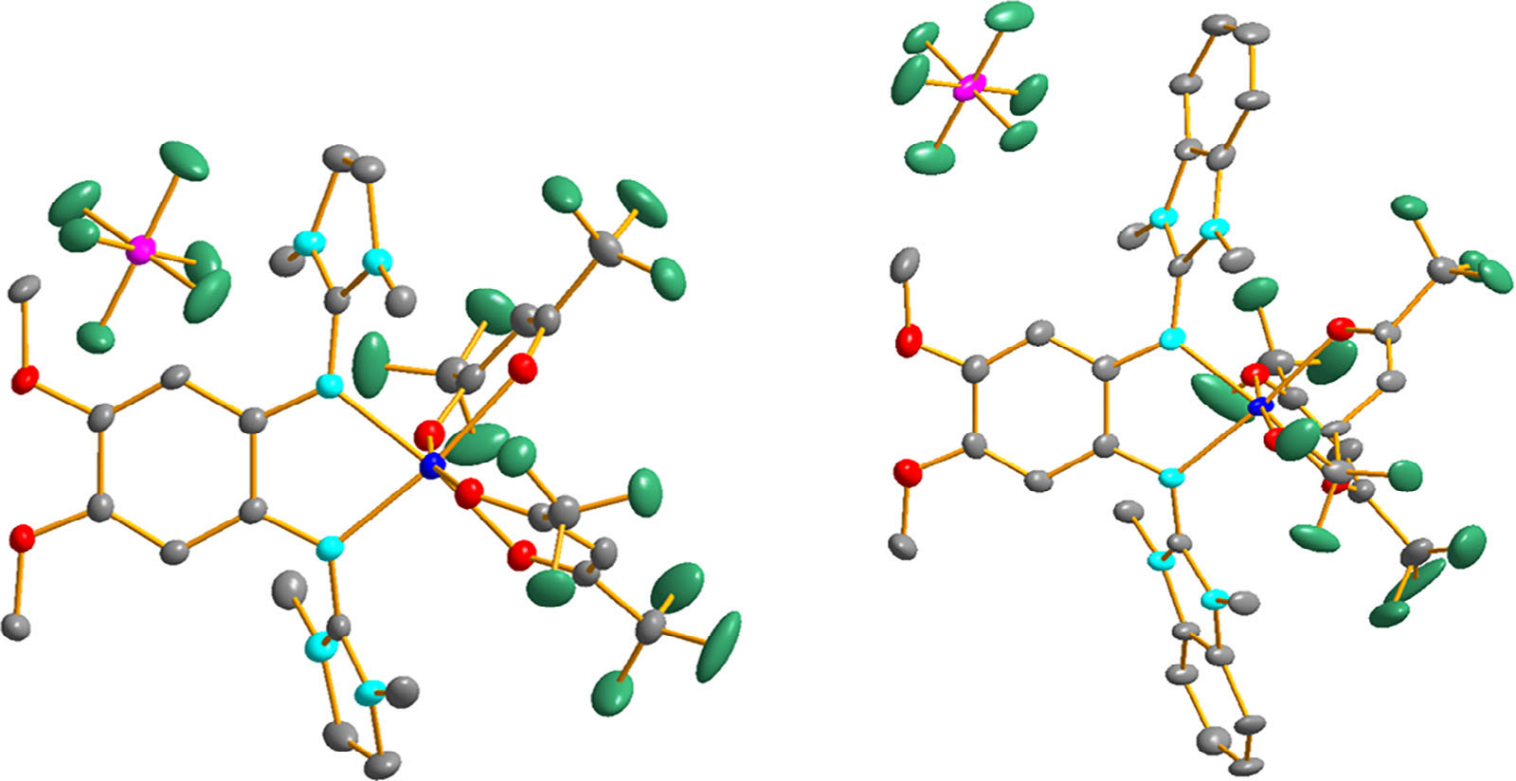
Illustration of the solid‐state structures of the two compounds [Co(hfac)_2_(L6)]PF_6_ and [Co(hfac)_2_(L7)]PF_6_. Displacement ellipsoids drawn at the 50% probability level. Hydrogen atoms omitted. Color code: Co dark blue, O red, N light blue, C grey, P pink, F green.

The Co‐O and Co‐N bond lengths in the crystallized monocationic complexes with tfac and hfac coligands are all above 2 Å (Figures 6, 7 and Tables 2, 3), and similar to those found in the neutral complexes. This is in line with ligand‐centered oxidation to give high‐spin Co^II^ complexes with radical monocationic diguanidine ligands. The C‐N bond lengths from the C_6_ ring carbon atoms to the directly bound N atoms of the guanidino groups (N1‐C1 and N4‐C2) are significantly shorter in the monocationic complexes with tfac or hfac coligands compared to the neutral complexes. On the other hand, the imino N‐C bond lengths within the guanidino groups are significantly longer. Moreover, the C‐C bond lengths within the C_6_ ring vary significantly, indicating loss of aromaticity. Based on these structures, one could conclude that oxidation is ligand‐centered for all complexes with hfac or tfac coligands, leading to high‐spin Co^II^ complexes with radical‐monocationic ligands (four unpaired electrons).

**Table 2:** Selected bond lengths (in Å) for cationic complexes with tfac coligands (atom numbering scheme, see Table 1).

bond	[Co(tfac)_2_(L5)]PF_6_	[Co(tfac)_2_(L6)]PF_6_	[Co(tfac)_2_(L7)]PF_6_
Co1‐O3	2.075(4)	2.0711(12)	2.051(3)
Co1‐O4	2.069(4)	2.0587(13)	2.069(3)
Co1‐O5	2.035(4)	2.0711(12)	2.047(3)
Co1‐O6	2.070(4)	2.0587(13)	2.044(3)
Co1‐N1	2.104(4)	2.103(2)	2.086(4)
Co1‐N4	2.095(4)	2.103(2)	2.097(4)
N1‐C1	1.348(6)	1.351(2)	1.359(5)
N4‐C2	1.362(6)	1.351(2)	1.357(5)
N1‐C7	1.360(7)	1.360(2)	1.360(5)
N4‐C12	1.357(7)	1.360(2)	1.333(5)
C1‐C2	1.443(7)	1.452(3)	1.446(6)

**Table 3:** Selected bond lengths (in Å) for cationic complexes with hfac coligands (atom numbering scheme, see Table 1)

bond	[Co(hfac)_2_(L1)] [Co(hfac)_3_]	[Co(hfac)_2_(L2)] [Co(hfac)_3_]	[Co(hfac)_2_(L3)]PF_6_	[Co(hfac)_2_(L6)]PF_6_	[Co(hfac)_2_(L7)]PF_6_
Co1‐O3	2.0840(18)	2.053(2)	2.087(2)	2.0947(17)	2.081(3)
Co1‐O4	2.0817(17)	2.056(2)	2.083(2)	2.0816(17)	2.048(3)
Co1‐O5	2.0638(17)	2.071(2)	2.080(2)	2.0874(16)	2.076(3)
Co1‐O6	2.0764(18)	2.085(2)	2.047(2)	2.0680(16)	2.062(3)
Co1‐N1	2.086(2)	2.094(3)	2.072(2)	2.0909(19)	2.121(4)
Co1‐N4	2.089(2)	2.097(2)	2.103(3)	2.0909(19)	2.086(4)
N1‐C1	1.354(3)	1.353(4)	1.352(4)	1.362(3)	1.355(6)
N4‐C2	1.355(3)	1.358(4)	1.360(4)	1.350(3)	1.350(6)
N1‐C7	1.376(3)	1.365(4)	1.375(4)	1.360(3)	1.347(6)
N4‐C12	1.377(3)	1.363(4)	1.368(4)	1.367(3)	1.365(6)
C1‐C2	1.448(3)	1.446(4)	1.443(4)	1.450(3)	1.468(6)

**Figure 8:**
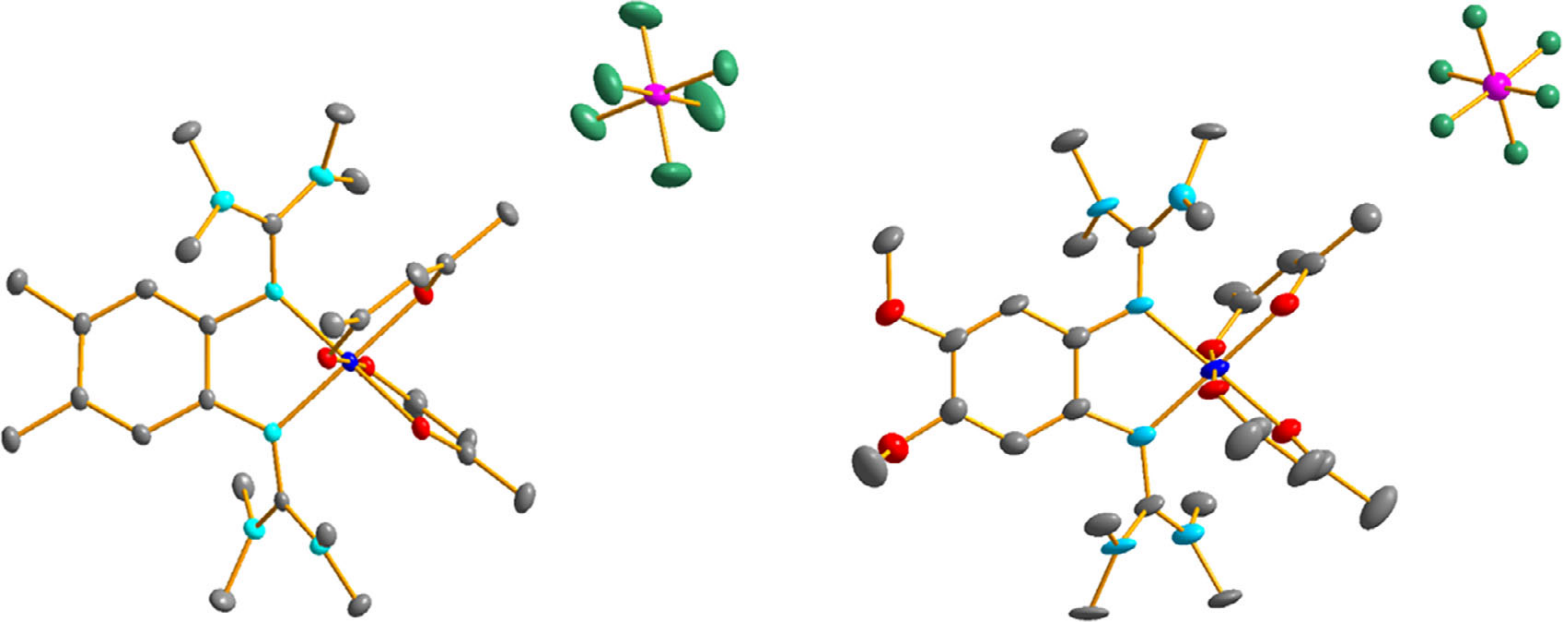
Illustration of the structures of [Co(acac)_2_(L1)]PF_6_ and [Co(acac)_2_(L5)]PF_6_. Displacement ellipsoids drawn at the 50% probability level (P and F atoms in [Co(acac)_2_(L5)]PF_6_ drawn as ball‐and‐stick representations). Hydrogen atoms omitted. Color code: Co dark blue, O red, N light blue, C grey, P pink, F green.

Finally, two cationic complexes with acac coligands crystallized, namely the compounds [Co(acac)_2_(L1)]PF_6_ and [Co(acac)_2_(L5)]PF_6_. Their structures are illustrated in Figure 8; selected bond lengths are collected in Table 4. The Co‐O and Co‐N bond lengths (all below 2 Å) are much shorter than in the neutral complexes, indicating metal‐centered oxidation and formation of low‐spin Co^III^ complexes. Moreover, the bond parameters within the diguanidine ligand do not change significantly upon oxidation, indicating the preservation of the neutral redox state. Hence, oxidation of the complexes with diguanidines L1 and L5 as redox‐active ligands and acac coligands yielded the low‐spin Co^III^ redox isomer with neutral diguanidine ligand (closed‐shell singlet state). However, we will see in the following that for two complexes with acac coligands (complexes with L6 and L7), the oxidation is metal‐ as well as ligand‐centered.

**Table 4:** Selected bond lengths (in Å) for the two crystallized oxidized complexes with acac coligands (atom numbering scheme, see Table 1).

bond	[Co(acac)_2_(L1)]PF_6_	[Co(acac)_2_(L5)]PF_6_
Co1‐O3	1.897(2)	1.882(5)
Co1‐O4	1.894(2)	1.899(5)
Co1‐O5	1.887(2)	1.898(5)
Co1‐O6	1.894(2)	1.899(5)
Co1‐N1	1.964(2)	1.955(5)
Co1‐N4	1.976(2)	1.957(6)
N1‐C1	1.414(3)	1.387(9)
N4‐C2	1.411(3)	1.414(8)
N1‐C7	1.344(3)	1.357(8)
N4‐C12	1.342(3)	1.364(8)
C1‐C2	1.394(4)	1.404(9)

**Figure 9:**
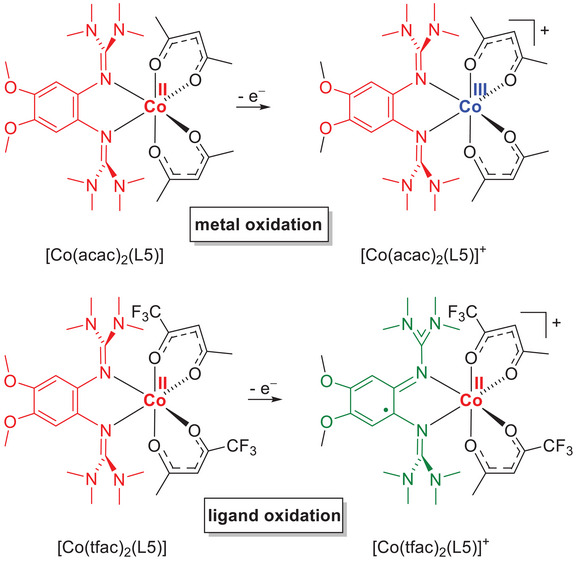
Illustration of the different electron distributions that result upon one‐electron oxidation of the Co complexes with L5 ligand and acac coligands or tfac coligands.

To highlight the differences in the redox chemistry, Figure 9 compares the structures before and after one‐electron oxidation of the cobalt complexes with L5 ligand and acac or tfac coligands. From the structural parameters, it is clear that [Co(acac)_2_(L5)]PF_6_ is present as a low‐spin Co^III^ complex with a neutral GFA ligand. The Co‐O and Co‐N bond lengths are below 2 Å, and also the C‐C bond lengths within the C_6_ ring of the diguanidine vary only slightly, indicating the presence of the diguanidine in its reduced, neutral redox state. Indeed, magnetometric measurements on solid [Co(acac)_2_(L5)]PF_6_ (see below) found a χT value close to zero. Nevertheless, the NMR spectrum did not show sharp signals, presumably due to the presence of tiny amounts of the paramagnetic high‐spin Co^II^ redox isomer in solution. By contrast, one‐electron oxidation of [Co(tfac)_2_(L5)]PF_6_ is ligand‐centered, yielding a paramagnetic high‐spin Co^II^ complex (all Co‐O and Co‐N bond lengths above 2 Å) with a radical‐monocationic diguanidine ligand and four unpaired electrons.

It should be stressed at this point that crystals suitable for a structural characterization with SC‐XRD were not obtained for [Co(acac)_2_(L6)]PF_6_ or Co(acac)_2_(L7)]PF_6_. For these two complexes, the analysis indicates the presence of a paramagnetic mixture of the two redox isomers (see below) that is likely to hamper their crystallization.

The decrease of the N═C bond order upon oxidation should also lead to changes in the IR spectra. Unfortunately, the IR absorptions of C═N double bonds usually are not very characteristic, due to variable intensity and the interaction with C═C double bonds that appear in the same range. Nevertheless, the calculations (B3LYP) on [Co(acac)_2_(L7)]^+^ yield for the high‐spin Co^II^ redox isomer (^5^A term) and the low‐spin Co^III^ redox isomer (^1^A term) strong absorptions at 1605 and 1620 cm^−1^, respectively, the vibrational modes of which are mainly ν(C═N) in character. Thus, there is a small difference of 15 cm^−1^ between the vibrational transitions of the two different electronic states. For comparison, vibrational modes of neutral [Co(acac)_2_(L7)] with wavenumbers of 1704.9, 1673.9, and 1649.2 cm^−1^ show large contributions of the C═N stretching motion. The symmetric combination of the C═N stretching vibrations contributes to the band at 1704.9 cm^−1^. The antisymmetric combination contributes to the bands at 1673.9 and 1649.2 cm^−1^, the latter being the most intense band with high ν(C═N) character. Thus, the most intense band shifts from 1649.2 cm^−1^ in the reduced form to 1619.8 cm^−1^ (^1^A) or 1605.3 cm^−1^ (^5^A) in the oxidized, monocationic form. In the experimental spectra, several bands show in the region 1700‐1500 cm^−1^. In line with the calculations, the bands generally shift to lower wavenumbers upon oxidation. In the case of [Co(acac)_2_(L7)], a strong band shifts from 1541 cm^−1^ before to 1517 cm^−1^ after oxidation. The shift value might indicate a dominating ^1^A state, but it is certainly not possible to distinguish between the states of the monocation from the IR spectra (see Supporting Information for more details).

### 2.4 Structural Characterization of a Salt of a Dicationic Complex

**Scheme 3:**
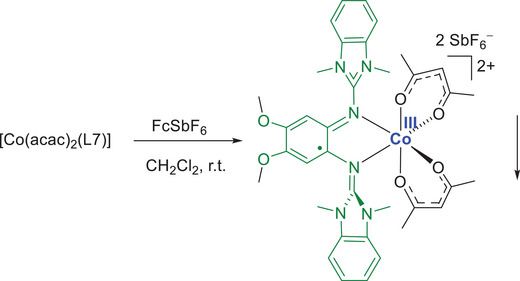
Formation of the salt [Co(acac)_2_(L7)](SbF_6_)_2_ in the course of reaction between [Co(acac)_2_(L7)] and FcSbF_6_.

**Figure 10:**
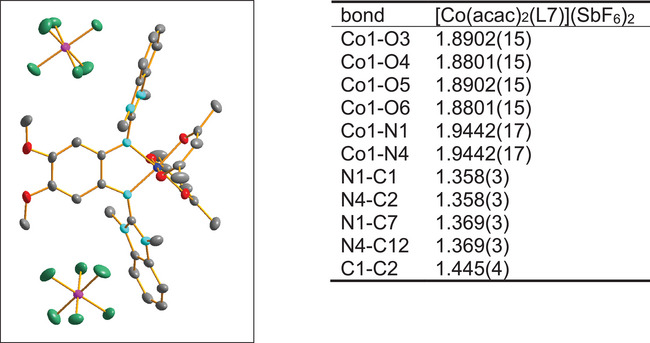
Illustration of the structure of [Co(acac)_2_(L7)](SbF_6_)_2_ in the solid state. Displacement ellipsoids drawn at the 50% probability level. Hydrogen atoms omitted. Color code: Co dark blue, O red, N light blue, C grey, P pink, F green. A table with selected bond lengths is included (atom numbering scheme, see Table 1).

Reaction of [Co(acac)_2_(L7)] with FcSbF_6_ in place of FcPF_6_ led to the formation of a small amount of crystalline salt [Co(acac)_2_(L7)](SbF_6_)_2_, being the product of two‐electron oxidation of the neutral complex (Scheme 3 and Figure 10). Since this salt is insoluble in CH_2_Cl_2_, a small amount of crystals precipitates from the reaction mixture. No effort was made to isolate this salt in larger amounts, since this work is concerned with the monocationic complexes, but the structure was analyzed by SC‐XRD. The Co‐O and Co‐N bond lengths in the dicationic complex (see Table imbedded in Figure 10) are all smaller than 2 Å, indicating the presence of low‐spin Co^III^. Moreover, the structural parameters in the ligand (long C1‐C2 and short N1‐C1 and N4‐C2 bonds) clearly indicate the presence of a radical monocationic diguanidine ligand. Therefore, the dication [Co(acac)_2_(L7)]^2+^ is a low‐spin Co^III^ complex with a radical monocationic ligand and a doublet electronic state.

### 2.5 UV‐vis Spectra of the Monocationic Complexes

**Figure 11:**
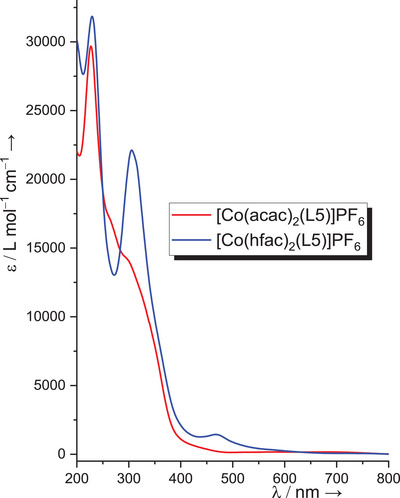
Comparison between the UV‐vis spectra of [Co(acac)_2_(L5)]PF_6_ and [Co(hfac)_2_(L5)]PF_6_ in CH_3_CN solutions.

In the UV‐vis spectra, all neutral cobalt complexes show a strong absorption in the region 270–340 nm. Only weak absorptions appear in the visible region above 400 nm. Figure 11 displays the UV‐vis spectra of the two compounds [Co(acac)_2_(L5)]PF_6_ and [Co(hfac)_2_(L5)]PF_6_ in CH_3_CN solutions. It can be seen that the complex with hfac coligands exhibits a strong band at 306 nm and a small, broad band centered at 468 nm that point to the presence of the radical monocationic ligand, (L5)**^·^** ^+^. These bands are missing in the spectrum of the complex with acac co‐ligands. This result is in line with the structural data that clearly point to the presence of the Co^III^ redox isomer with neutral diguanidine in [Co(acac)_2_(L5)]PF_6_, and the Co^II^ redox isomer with radical monocationic diguanidine in [Co(hfac)_2_(L5)]PF_6_. However, an unambiguous assignment of the electron distribution based on the UV‐vis spectra is not possible. The weak and broad absorptions in the visible region from excitations centered at the radical cationic diguanidine ligand are often superposed by equally weak metal d‐d transitions. Nevertheless, it is worth mentioning one observation. The UV‐vis spectrum of [Co(acac)_2_(L6)]PF_6_ displays a band around 462 nm and a very weak band at 630 nm, and absorption in the visible region is also visible for the complex [Co(acac)_2_(L7)]PF_6_ (at 525 and 735 nm, see Supporting Information). This observation points to the presence of radical monocationic diguanidine ligands in these complexes (see TDDFT calculations below). This assumption is supported by the results of the CV measurements and will be further substantiated by the results of magnetometric measurements and quantum‐chemical calculations.

### 2.6 Magnetometric Measurements on the Monocationic Complexes

The hfac and tfac coligands favor ligand‐centered oxidation, leading to a paramagnetic high‐spin Co^II^ redox isomer with a radical monocationic diguanidine ligand, summing up to four unpaired electrons. On the other hand, the acac co‐ligands generally favor the formation of diamagnetic low‐spin Co^III^ complexes upon oxidation, but there are some indications that ligand‐ and metal‐centered oxidation occur simultaneously in the case of the complexes with L6 and L7.

**Figure 12:**
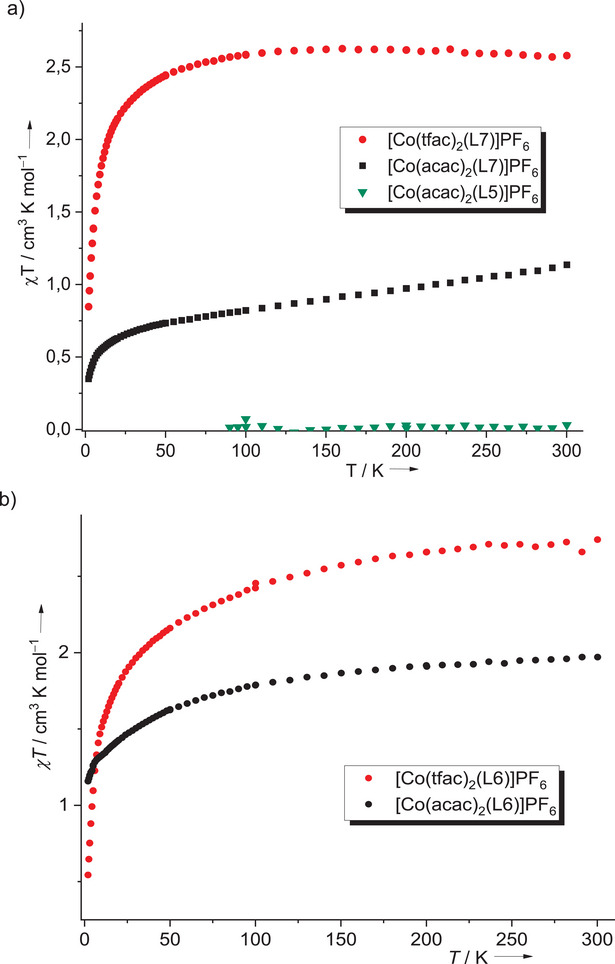
a) Magnetometric (SQUID) curves for the complexes a) [Co(tfac)_2_(L7)]PF_6_, [Co(acac)_2_(L7)]PF_6_ and [Co(acac)_2_(L5)]PF_6_, and b) [Co(tfac)_2_(L6)]PF_6_ and [Co(acac)_2_(L6)]PF_6_.

To obtain more information, we studied the five complexes [Co(acac)_2_(L5)]PF_6_, [Co(acac)_2_(L6)]PF_6_, [Co(acac)_2_(L7)]PF_6_, [Co(tfac)_2_(L6)]PF_6_, and [Co(tfac)_2_(L7)]PF_6_ with magnetometric (superconducting quantum interference divice (SQUID)) measurements. The SQUID curves found a χT value of 2.58 cm^3^ K mol^−1^ for [Co(tfac)_2_(L7)]PF_6_ and 2.74 cm^3^ K mol^−1^ for [Co(tfac)_2_(L6)]PF_6_ at room temperature. These high values clearly indicate the presence of high‐spin Co^II^. For a system with a high‐spin Co^II^ atom (three unpaired electrons) and a radical cationic ligand (one unpaired electron), a χT value of (1.875 + 0.375) cm^3^ K mol^−1^ = 2.250 cm^3^ K mol^−1^ is expected from the spin‐only formula. The larger experimental values indicate significant spin‐orbit coupling, as is typical for octahedral high‐spin Co^II^ complexes. Next, we recorded curves for the three complexes with acac co‐ligands. The two structurally characterized compounds with acac coligand, [Co(acac)_2_(L1)]PF_6_ and [Co(acac)_2_(L5)]PF_6_, clearly are predominantly present as low‐spin Co^III^ complexes with neutral diguanidine ligand. The magnetometric measurements on [Co(acac)_2_(L5)]PF_6_ indeed found a χT value close to zero (Figure 12a, green triangles). By contrast, the compounds [Co(acac)_2_(L6)]PF_6_ and [Co(acac)_2_(L7)]PF_6_ turned out to be paramagnetic, with χT values of 1.97 cm^3^ K mol^−1^ for [Co(acac)_2_(L6)]PF_6_ and 1.13 cm^3^ K mol^−1^ for [Co(acac)_2_(L7)]PF_6_ at room temperature. Hence, they could not be present exclusively as diamagnetic low‐spin Co^III^ complexes with neutral diguanidine ligands. On the other hand, the values are too low for high‐spin Co^II^ complexes with radical monocationic diguanidine ligands. The obvious inference is that Co^III^ and Co^II^ redox isomers coexist. Based on the χT values of the high‐spin Co^II^ complexes with tfac complexes, one could estimate that [Co(acac)_2_(L6)]PF_6_ consists of roughly 70% and [Co(acac)_2_(L7)]PF_6_ of roughly 40% of the Co^II^ redox isomer. In line with the higher reduction potential for L7^+^/L7 compared with L6^+^/L6, the complex with L7 has the lower χT value. The higher potential of L7^+^/L7 renders the Co^II^ redox isomer with radical monocationic diguanidine L7^+^ less favorable compared to the complex with L6^+^. The data are also consistent with the results of the CV and UV‐vis measurements.

Moreover, the Evans NMR technique was used to obtain information about the magnetic properties of the complex [Co(acac)_2_(L7)]^+^ in dichloromethane solution (see Supporting Information for details). A χT value of 0.572 cm^3^ K mol^−1^ was obtained at 298 K. This value is smaller than the 1.13 cm^3^ K mol^−1^ measured for solid [Co(acac)_2_(L7)]PF_6_ at room temperature with SQUID but is in line with the presence of a mixture of several states. The χT value decreases with decreasing temperature; a value of 0.325 cm^3^ K mol^−1^ was obtained at 234 K in dichloromethane solution. This trend is in line with a singlet ground state.

### 2.7 Quantum‐Chemical Calculations on the Monocationic Complexes

Finally, quantum chemical (DFT) calculations were carried out to obtain minimum‐energy structures and their energies for all low‐energy electronic states of [Co(acac)_2_(L7)]^+^ with and without inclusion of the PF_6_^−^ counter‐ion and the solvent effect (COSMO, ε_r_ = 37.5). The complex with L7 was chosen since the experimental (SQUID, CV, and UV‐vis) data showed that oxidation leads to a significant percentage of the Co^II^ redox isomer; the Co^III^ and the Co^II^ redox isomers are likely to have similar energies.

Table 5 includes the relative energies of the terms calculated for [Co(acac)_2_(L7)]^+^. The calculations predict a quintet term, ^5^A, to be of lowest energy. However, a closed‐shell singlet term is only 6.8 kJ mol^−1^ higher in energy (calculations with the def2‐TZVP basis set and without inclusion of a dielectric environment). Moreover, a broken‐symmetry state with *S*_z_ = 1, ^(3)^A, is located at 7.0 kJ mol^−^^1^, and a broken‐symmetry state with *S*_z_ = 0, ^(1)^A, at 43.4 kJ mol^−1^. These states belong to a high‐spin (^(^^3)^A) and a low‐spin Co^II^ (^(1)^A) complex, respectively, in which the ligand spin couples antiferromagnetically with the metal spins. Hence, the small energy difference between the ^5^A and ^1^A states argues for the presence of a mixture of both redox isomers, in line with the results from the magnetometric measurements.

**Table 5:** Relative energies (in kJ mol^−1^) of the different states according to B3LYP calculations of the cobalt complex without and with counterion and solvent effect (COSMO, ε_r_ = 37.5).

system	ε_r_	term	description	*E*_el_(SV(P))	*E*_el_(TZVP)
[Co(acac)_2_(L7)]^+^	1	^5^A	*hs*‐Co^II^, L7**^·^** ^+^ (↑↑↑,↑)	0	0
	1	^(3)^A^[a]^	*hs*‐Co^II^, L7**^·^** ^+^ (↑↑↑,↓)	6.8	7.0
	1	^(1)^A^[b]^	*ls*‐Co^II^, L7**^·^** ^+^ (↑,↓)	48.6	43.4
	1	^1^A	*ls*‐Co^III^, L7^0^	8.7	6.8
[Co(acac)_2_(L7)]PF_6_	1	^5^A	*hs*‐Co^II^, L7**^·^** ^+^ (↑↑↑,↑)	0	0
	1	^(3)^A^[a]^	*hs*‐Co^II^, L7**^·^** ^+^ (↑↑↑,↓)	4.4	4.7
	1	^(1)^A^[b]^	*ls*‐Co^II^, L7**^·^** ^+^ (↑,↓)	48.5	43.8
	1	^1^A	*ls*‐Co^III^, L7^0^	21.4	20.6
	37.5	^5^A	*hs*‐Co^II^, L7**^·^** ^+^ (↑↑↑,↑)	0	0
	37.5	^(3)^A^[a]^	*hs*‐Co^II^, L7**^·^** ^+^ (↑↑↑,↓)	3.1	2.5
	37.5	^(1)^A^[b]^	*ls*‐Co^II^, L7**^·^** ^+^ (↑,↓)	50.6	45.3
	37.5	^1^A	*ls*‐Co^III^, L7^0^	20.7	23.5

^[a]^ Broken‐symmetry state with S_z_ = 1.

^[b]^ Broken‐symmetry state with S_z_ = 0.

**Figure 13:**
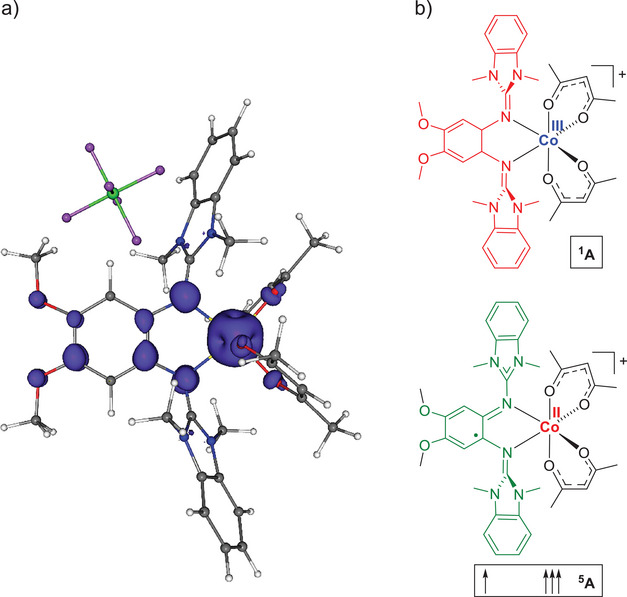
a) Illustration of the spin‐density distribution in the ^5^A state of [Co(acac)_2_(L7)]PF_6_, calculated with B3LYP/def2‐TZVP. b) Illustration of the electron distribution in the ^1^A and ^5^A states of [Co(acac)_2_(L7)]^+^.

In the case of the ^5^A term, the cobalt‐ligand bond lengths are relatively large (Co‐N bond lengths of 2.169 and 2.169 Å and Co−O bond lengths of 2.047, 2.067, 2.047, und 2.067 Å), arguing for a high‐spin Co^II^ complex. Moreover, the C‐C bond lengths within the C_6_ ring vary (1.443, 1.410, 1.410, 1.377, 1.377, and 1.441 Å), indicating the presence of a radical monocationic ligand. The calculated ^5^A state is not a state with four unpaired electrons localized at the metal ion, as can be expected for a complex of a d^6^ high‐spin metal ion (corresponding to a ^5^T_2g_ term in O_h_ symmetry), but it is a state with a d^7^ high‐spin metal ion and, furthermore, one oxidation equivalent residing on the ligand (L**^·^** ^+^), see Figure 13. In contrast, the ^1^A state conforms to the complex of a d^6^ low‐spin Co^III^ metal ion, corresponding to a ^1^A_1g_ state in O_h_ symmetry. Unfortunately, the calculations cannot provide the energy of a state with Co^III^ in a high‐spin state because the calculations for the high‐spin states converge to a state with an oxidized ligand and Co^II^.

In the case of the closed‐shell singlet state, the short cobalt‐ligand bond lengths (Co‐N bond lengths of 1.973 Å and Co−O bond lengths of 1.907, 1.908, 1.907, and 1.908 Å) and the moderate variations of the C‐C bond lengths within the C_6_ ring (1.389, 1.401, 1.401, 1.387, 1.387, and 1.414 Å) indicate the presence of a low‐spin Co^III^ complex with a neutral diguanidine ligand.

Next, we carried out calculations with the inclusion of the PF_6_^−^ counterion. As a starting structure, we cut out a section with one dication and two counterions from the crystal structure of [Co(acac)_2_(L7)](SbF_6_)_2_, deleted the SbF_6_^−^ counterion with a larger distance to the complex, and replaced Sb by P in the remaining counterion to obtain a starting structure with one [Co(acac)_2_(L7)]^+^ and one PF_6_^−^. Then, the structure was optimized. The calculated relative energies of terms with counterions for [Co(acac)_2_(L7)]PF_6_ are included in Table 5. In particular, the relative energy of the closed‐shell singlet state increases in the presence of the counterion. The ^5^A term again is the lowest‐energy term, and the closed‐shell singlet term is 20.6 kJ mol^−^^1^ higher (calculations with the TZVP basis set and without the inclusion of a dielectric environment). The broken‐symmetry ^(3)^A state is located at 4.7 kJ mol^−^^1^, and the broken‐symmetry ^(1)^A state at 43.8 kJ mol^−1^.

The bond lengths in the states of [Co(acac)_2_(L7)]PF_6_ are similar to those in the corresponding states of the cation in the absence of the counter‐ion. Hence, the electrons should be similarly distributed. In the ^5^A term, the Co‐N bond lengths measure 2.162 and 2.173 Å, and the Co‐O bond lengths measure 2.047, 2.071, 2.068, and 2.075 Å. Together with the significant variations in the C‐C bond lengths of the aromatic ring (1.443, 1.406, 1.411, 1.378, 1.375, and 1.441 Å), these values indicate the presence of high‐spin Co^II^ and an oxidized ligand. Figure 13 shows a plot of the spin‐density distribution for the ^5^A state of [Co(acac)_2_(L7)]PF_6_. In line with the description of a high‐spin Co^II^ complex with a radical‐monocationic diguanidine ligand, the spin density is predominantly located on the cobalt atom and the diguanidine ligand.

For the ^1^A term, the Co‐N bond lengths (1.983 und 1.990 Å) and the Co‐O bond lengths (1.897, 1.910, 1.915, und 1.917 Å) are shorter. Also, the C‐C bond lengths in the aromatic ring are shorter and vary only slightly (1.387, 1.396, 1.400, 1.387, 1.388, and 1.414 Å). All these structural parameters argue for the presence of low‐spin Co^III^ and a neutral ligand. The inclusion of a dielectric environment (ε_r_ = 37.5) does not significantly change the relative energies of the terms of [Co(acac)_2_(L7)]PF_6_.

Still, the energy difference between the two redox isomers is small. Hence, the quantum‐chemical calculations support the conclusions from the experimental results. The complex [Co(acac)_2_(L7)]PF_6_ is likely to be present as a mixture of Co^II^ and Co^III^ redox isomers. Due to the significant deviation of the structural parameters in the Co^II^ redox isomer and the Co^III^ redox isomer, their interconversion is likely to be subjected to a significant barrier.

The SQUID data point to a low‐spin ground state, whereas the density functional calculations with the B3LYP functional yield a high‐spin ground state (^5^A). However, the energy difference of the different states, and in particular between the ^1^A and the ^5^A term, strongly depends on the functional. To study this dependency in more detail, calculations were carried out with three different functionals. The BLYP functional is used as representative of a gradient‐corrected functional. The TPSSh and B3LYP functionals are hybrid functionals with different amounts of HF exchange, 10 and 20%, respectively. For the bare cation [Co(acac)_2_(L7)]^+^, the values of the energy of the ^5^A term with respect to the ^1^A state obtained with the B3LYP, the TPSSh, and the BLYP functionals amount to ‐6.8, 33.7, and 52.2 kJ mol^−1^, respectively (Table 6). Likewise, for [Co(acac)_2_(L7)]PF_6_, the values amount to ‐20.6, 22.0, and 46.2 kJ mol^−1^. Thus, with the TPSSh functional (for example), the low‐spin ^1^A state is the lowest‐lying state, in agreement with the SQUID data.

**Table 6:** Calculated relative energy of the ^5^A term of [Co(acac)_2_(L7)]^+^ and [Co(acac)_2_(L7)]PF_6_ with respect to the ^1^A state obtained with different functionals (Δ*E* = *E*(^5^A) − *E*(^1^A), in kJ mol^−1^). A positive value implies a ^1^A ground state.

functional	Δ*E*([Co(acac)_2_(L7)]^+^)	Δ*E*([Co(acac)_2_(L7)]PF_6_)
B3LYP	−6.77	−20.56
TPSSh	33.73	21.95
BLYP	52.22	46.16

Calculations of the electronic excitation energies (TDDFT) for the ^1^A low‐spin state of [Co(acac)_2_(L7)]^+^ yield very weak transitions at 836 and 564 nm, weak transitions at 765 and 454 nm, and a transition of medium strength at 414 nm. These results are in line with the experimental spectrum, which shows a shoulder of the UV absorptions extending between 450 and 650 nm and, in particular, an additional very weak absorption at 735 nm.

## 3 Conclusions

Herein, the redox chemistry of a series of 21 cobalt complexes with redox‐active diguanidine ligands is studied. The electronic structure in the monocationic complexes obtained upon one‐electron oxidation is adjusted by variation of the redox‐active diguanidine and the coligands. Oxidation is ligand‐centered for all complexes with tfac and hfac coligands, leading to high‐spin Co^II^ complexes with radical monocationic diguanidine ligand. In the case of the complexes with acac coligands, metal‐centered oxidation is generally observed, giving low‐spin Co^III^ complexes with neutral diguanidine ligand. On the other hand, by combining low‐potential diguanidine ligands with acac coligands, the energies of the redox isomers are harmonized, leading to ligand‐ as well as metal‐centered one‐electron oxidation. Especially, diguanidine ligands with methoxy groups in the backbone adjust the energies of the highest occupied diguanidine π orbital with the energies of the occupied cobalt d orbitals, leading to a small energy difference between the possible redox isomers (the electron distributions are sketched in Figure 13b). The experimental data point to a possible thermal equilibrium between both redox isomers, with the low‐spin Co^III^ redox isomer exhibiting a slightly lower enthalpy and entropy. Consequently, mixtures of Co^III^ and Co^II^ redox isomers are obtained. In line with the experimental results, quantum‐chemical calculations found a very low energy difference between the Co^III^ and Co^II^ redox isomers. A table with the most adequate descriptions of the electron distributions for all monocationic complexes is included in the Supporting Information.

In future work the barrier for the interconversion of the two redox isomers in this bistable system will be tuned by variations in the reorganization energy, and the switching behavior in the solid state optimized by the introduction of cooperative effects (e.g. π‐π‐stacking or hydrogen bonding). The optimization of the reorganization energy and the intermolecular interactions prepares these systems for applications in spin‐switching devices.

## 4 Experimental Details

The synthetic details and analytical data for all compounds, as well as the details of the quantum chemical (DFT) calculations, are included in the Supporting Information. Deposition Numbers 2439643 (for [Co(hfac)_2_(L1)]), 2439644 (for [Co(acac)_2_(L1)]PF_6_), 2439645 (for [Co(tfac)_2_(L2)]), 2439646 (for [Co(acac)_2_(L7)](SbF_6_)_2_), 2439647 (for [Co(hfac)_2_(L1)][Co(hfac)_3_]), 2439648 (for [Co(hfac)_2_(L2)][Co(hfac)_3_]), 2439649 (for [Co(acac)_2_(L3)]), 2439650 (for [Co(hfac)_2_(L3)]PF_6_), 2439651 (for [Co(acac)_2_(L4)]), 2439652 (for [Co(hfac)_2_(L4)]), 2439653 (for [Co(acac)_2_(L5)]), 2439654 (for [Co(hfac)_2_(L5)]), 2439655 (for [Co(tfac)_2_(L7)]), and 2439656 (for [Co(acac)_2_(L2)]), 2439767 (for [Co(tfac)_2_(L6)]), 2439768 (for [Co(hfac)_2_(L6)]), 2439769 (for [Co(acac)_2_(L5)]PF_6_), 2439770 (for [Co(tfac)_2_(L6)]PF_6_), 2439771 (for [Co(tfac)_2_(L5)]PF_6_), 2439772 (for [Co(hfac)_2_(L6)]PF_6_), 2439773 (for [Co(hfac)_2_(L7)]), 2439774 (for [Co(acac)_2_(L6)]), 2439775 (for [Co(tfac)_2_(L7)]PF_6_), 2439776 (for [Co(hfac)_2_(L7)]PF_6_), and 2439777 (for [Co(tfac)_2_(L5)]) contain the supplementary crystallographic data for this paper. These data are provided free of charge by the joint Cambridge Crystallographic Data Centre and Fachinformationszentrum Karlsruhe Access Structures service.

## Conflict of Interest

The authors declare no conflict of interests.

## Supporting information



Supporting Information

## Data Availability

The data that support the findings of this study are available in the supplementary material of this article.
