# Supplementary material for: Fine Tuning of the Redox‐Active Diguanidine Ligand in Cationic Cobalt Complexes for Electronic Structure Control
Source: Chemistry. 2025 Aug 2;31(51):e202501382. doi: 10.1002/chem.202501382 (PMC12434436; doi:10.1002/chem.202501382)
Supplement: Supplementary file 1 — Supporting Information [file CHEM-31-e202501382-s001.pdf]

Content

1. General Information 2
2. Synthesis and analytical data for the cobalt complexes 3
2.1 Overview of the synthesized new complexes 3
2.2 Synthesis details of neutral complexes 5
2.2.1 Neutral cobalt complexes of ligand L1 5
2.2.2 Neutral cobalt complexes of ligand L2 7
2.2.3 Neutral cobalt complexes of ligand L3 9
2.2.3 Neutral cobalt complexes of ligand L4 11
2.2.5 Neutral cobalt complexes of ligand L5 13
2.2.6 Neutral cobalt complexes of ligand L6 15
2.2.7 Neutral cobalt complexes of ligand L7 17
2.3 Analytical data of neutral complexes 19
2.3.1 UV-Vis spectra of neutral complexes 19
2.3.2 Cyclic Voltammetry curves of the neutral complexes 30
2.3.3 Crystallographic data of the neutral complexes 51
2.4 Synthesis details of oxidized, monocationic complexes 60
2.4.1 Monocationic cobalt complexes of ligand L1 60
2.4.2 Monocationic cobalt complexes of ligand L2 62
2.4.3 Monocationic cobalt complexes of ligand L3 64
2.4.4 Monocationic cobalt complexes of ligand L4 66
2.4.5 Monocationic cobalt complexes of ligand L5 68
2.4.6 Monocationic cobalt complexes of ligand L6 70
2.4.7 Monocationic cobalt complexes of ligand L7 72
2.5 Analytical data for the oxidized, monocationic complexes 74
2.5.1 UV-Vis and IR spectra 74
2.5.2 Crystallografic data for the oxidized, monocationic complexes 90
2.5.3 Magnetometric (SQUID, Evans NMR) measurements for the oxidized, monocationic complexes 100
2.5.4 Summary of the electron distributions within the complexes (neutral and monocationic) in the solid state 106
3. Quantum-chemical calculations on the cobalt complexes 108
3.1 Details of quantum-chemical calculations 108
3.2 Total energies and optimized coordinates 110
3.2.1 Total energies 110
3.2.2 Optimized coordinates by B3LYP calculations 110
3.2.3 Optimized coordinates by TPSSh calculations 136
3.2.4 Optimized coordinates by BLYP calculations 148

3.3 Calculated UV-vis spectra for [Co(acac)$_2$(L7)] and [Co(acac)$_2$(L7)]$^+$ 160
3.4 Calculated vibrational spectra of [Co(acac)$_2$(L7)] and[Co(acac)$_2$(L7)]$^+$ 164

1. General Information

All reactions described in this work were carried out under a dry argon or nitrogen atmosphere by using dry glass ware and standard Schlenk techniques to avoid hydrolysis. The reactions were prepared in a glove box in a dry argon or nitrogen atmosphere (*MBRAUN LABmaster DP* (*MB-20-G*); *MBRAUN LABstar* (*MB-10-G*); *Sylatech Y-05-7986*). The solvents dichloromethane, diethylether and tetrahydrofuran were dried with an *MBRAUN MB-SPS-800*, degassed by the freeze-pump-thaw method and stored over molecular sieves (3 Å or 4 Å). Other solvents were purchased from Acros Organics and degassed and stored similarly.
All elemental analyses to determine the mass percentage of hydrogen, carbon and nitrogen within a substance were carried out at the Microanalytical Laboratory at the Chemical Institute at Heidelberg University using a *vario EL* and *vario MICRO cube* by *Elementar Analysensysteme GmbH*.
All mass spectrum measurements were performed by the MS-Laboratory at the Chemical Institute at Heidelberg University using a *Bruker Apex-Qe FT-ICR 9.4T* (HR-ESI) or a *Finnigan LCQ quadrupole ion trap* (ESI).
Infrared spectra were recorded using an *ALPHA Platinum ATR* spectrometer under ambient conditions.
Crystals suitable for solid structure determination were taken from the parent solution and immersed instantaneously into perfluorinated polyether oil and fixed on a cryo loop. Full shells of intensity data were collected at low temperature with a *Bruker D8 Venture* diffractometer (Mo-$K\alpha$ radiation, microfocus X-ray tube, Photon III Detector).
Data were processed with the standard Bruker (SAINT, APEX3/4) software package.[1] Multiscan absorption correction was applied using the SADABS program.[2] The structures were solved by intrinsic phasing [3] and refined using the SHELXTL software package (Version 2018/3).[4] Graphical handling of the structural data during solution and refinement were performed with OLEX2.[5] All non-hydrogen atoms were given anisotropic displacement parameters. Hydrogen atoms bound to carbon were input at calculated positions and refined with a riding model. Hydrogen atoms bound to boron or nitrogen were located in difference Fourier syntheses and fully refined. When found necessary, suitable geometry and adp restraints were applied.
The visualization of the structures was performed using *Mercury 4.1.0*. Displacement ellipsoids are depicted with 50 % probability.
Crystallographic data for the structures reported in this paper have been deposited in the Cambridge Crystallographic Data Centre (Deposition Numbers). These data can be obtained free of charge from The Cambridge Crystallographic Data Centre via www.ccdc.cam.ac.uk/data_request/cif.

2. Synthesis and analytical data for the cobalt complexes

2.1 Overview of the synthesized new complexes

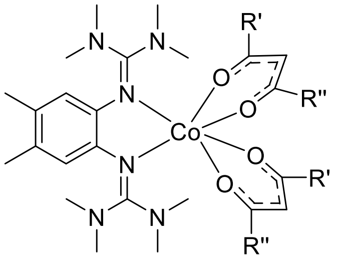

1. [Co(acac)$_2$(L1)]
2. [Co(tfac)$_2$](L1)]
3. [Co(hfac)$_2$(L1)]

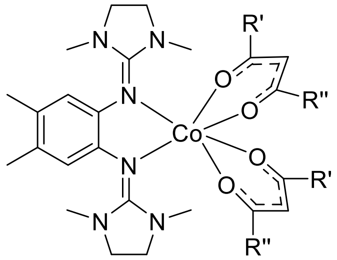

1. [Co(acac)$_2$(L2)]
2. [Co(tfac)$_2$(L2)]
3. [Co(hfac)$_2$(L2)]

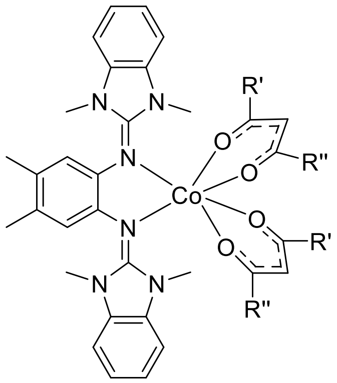

1. [Co(acac)$_2$(L3)]
2. [Co(tfac)$_2$(L3)]
3. [Co(hfac)$_2$(L3)]

1.: R',R''= CH_3

2.: R' = CH_3
R''= CF_3

3.: R',R''= CF_3

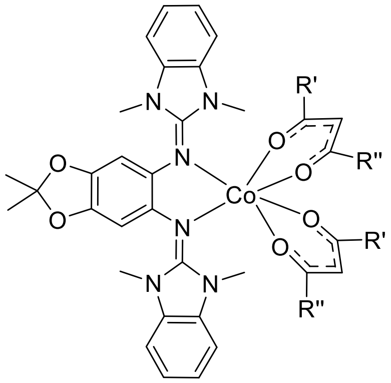

1. [Co(acac)$_2$(L4)]
2. [Co(tfac)$_2$(L4)]
3. [Co(hfac)$_2$(L4)]

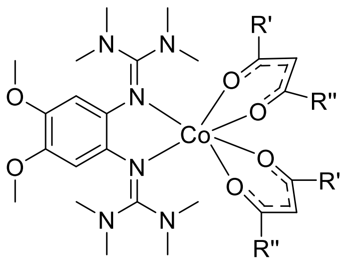

1. [Co(acac)$_2$(L5)]
2. [Co(tfac)$_2$(L5)]
3. [Co(hfac)$_2$(L5)]

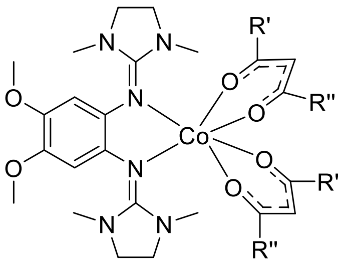

1. [Co(acac)$_2$(L6)]
2. [Co(tfac)$_2$(L6)]
3. [Co(hfac)$_2$(L6)]

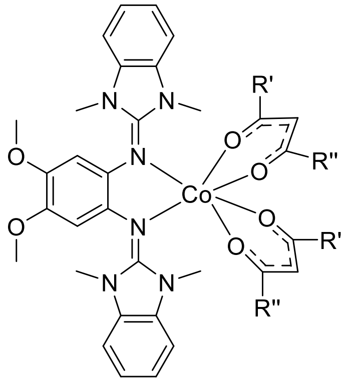

1. [Co(acac)$_2$(L7)]
2. [Co(tfac)$_2$(L7)]
3. [Co(hfac)$_2$(L7)]

Lewis structures of the new Co^{II} complexes with redox-active diguanidine ligand synthesized in this work.

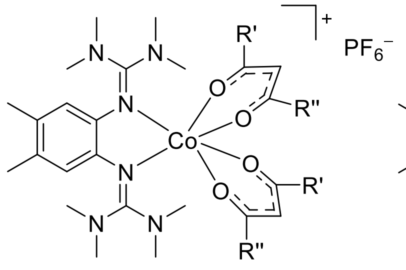

1. $[Co(acac)_2(L1)]PF_6$
2. $[Co(tfac)_2(L1)]PF_6$
3. $[Co(hfac)_2(L1)]PF_6$

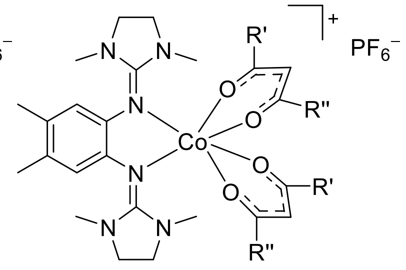

1. $[Co(acac)_2(L2)]PF_6$
2. $[Co(tfac)_2(L2)]PF_6$
3. $[Co(hfac)_2(L2)]PF_6$

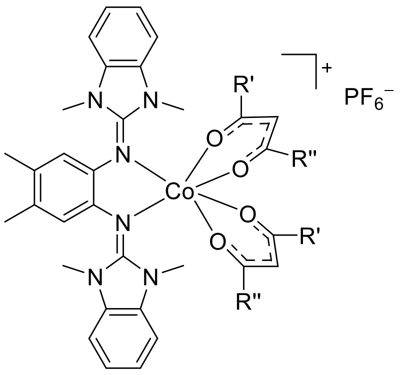

1. $[Co(acac)_2(L3)]PF_6$
2. $[Co(tfac)_2(L3)]PF_6$
3. $[Co(hfac)_2(L3)]PF_6$

1.: R',R''= CH_3

2.: R' = CH_3
R''= CF_3

3.: R',R''= CF_3

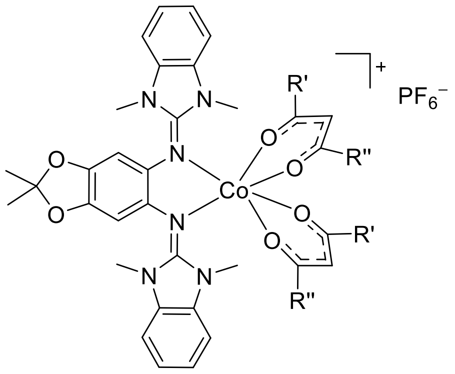

1. $[Co(acac)_2(L4)]PF_6$
2. $[Co(tfac)_2(L4)]PF_6$
3. $[Co(hfac)_2(L4)]PF_6$

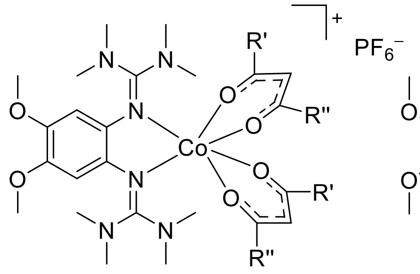

1. $[Co(acac)_2(L5)]PF_6$
2. $[Co(tfac)_2(L5)]PF_6$
3. $[Co(hfac)_2(L5)]PF_6$

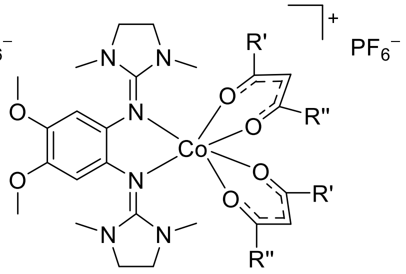

1. $[Co(acac)_2(L6)]PF_6$
2. $[Co(tfac)_2(L6)]PF_6$
3. $[Co(hfac)_2(L6)]PF_6$

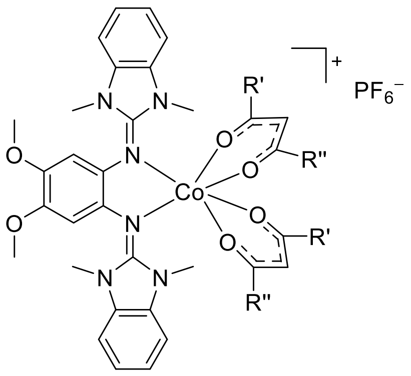

1. $[Co(acac)_2(L7)]PF_6$
2. $[Co(tfac)_2(L7)]PF_6$
3. $[Co(hfac)_2(L7)]PF_6$

Lewis structures of the oxidized complexes synthesized in this work.

2.2 Synthesis details of neutral complexes

2.2.1 Neutral cobalt complexes of ligand L1

Please note that the neutral complexes are sensitive to oxidation and water. Deviations in the EA are caused by brief air contact in the course of the EA measurements.

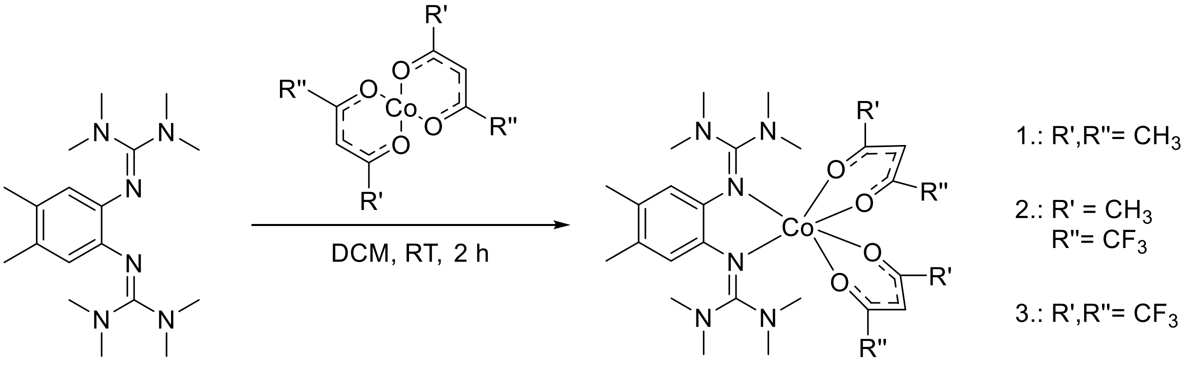

[Co(acac)$_2$(L1)]

Ligand L1 (33.2 mg, 0.1 mmol) and [Co(acac)$_2$] (25.7 mg, 0.1 mmol) were dissolved in dichloromethane (3 mL). The red solution was stirred at room temperature for a period of 2 h. Then, the solvent was removed in vacuo to yield 57 mg (96%, 0.096 mmol) of [Co(acac)$_2$(L1)] as a dark red solid.

Elemental analysis ($C_{28}H_{48}CoN_6O_4$ + 0.33 CH_2Cl_2):

calcd.:	C 54.54%	H 8.51%	N 13.74%
found:	C 55.06%	H 8.41%	N 13.66%

UV-vis (CH_3CN): λ_{max} (ε / M^{-1} cm^{-1}): 249 ($2.23\cdot10^4$), 312 ($1.81\cdot10^4$), 567 (65) nm.

ESI-HRMS (pos., CH_2Cl_2): *m/z* (M-acac) = calcd. 490.2545, found 490.2461.

CV (CH_2Cl_2, Ag/AgCl, ^{n}Bu$_4$NPF$_6$ as supporting electrolyte, scan speed 30 mV s^{-1}): $E_{1/2}$ = -0.63, 0.22, 0.69 V vs. ferrocenium/ferrocene.

[Co(tfac)$_2$(L1)]

Ligand L1 (33.2 mg, 0.1 mmol) and [Co(tfac)$_2$] (36.7 mg, 0.1 mmol) were dissolved in acetonitrile (3 mL). The red solution was stirred at room temperature for a period of 2 h. Then, the solvent was removed in vacuo to yield 68 mg (97%, 0.097 mmol) of [Co(tfac)$_2$(L1)] as a red solid. The solid was washed with diethylether.

Elemental analysis ($C_{28}H_{40}CoN_6F_6O_4$+CH_3CN+Et_2O):

calcd.:	C 51.25%	H 6.71%	N 12.31%
found:	C 50.30%	H 6.04%	N 13.15%

UV-vis (CH_3CN): λ_{max} (ε / M^{-1} cm^{-1}): 249 ($3.75 \cdot 10^4$), 312 ($3.68 \cdot 10^4$), 563 (138) nm.

ESI-HRMS (pos., CH_2Cl_2): *m/z* (M-tfac) = calcd. 544.2184, found 544.2190.

CV (CH_2Cl_2, Ag/AgCl, $^nBu_4NPF_6$ as supporting electrolyte, scan speed 30 mV s^{-1}): $E_{1/2}$ = 0.07, 0.52, 1,05 V vs. ferrocenium/ferrocene.

[Co(hfac)$_2$(L1)]

Ligand L1 (33.2 mg, 0.1 mmol) and [Co(hfac)$_2$] (47.5 mg, 0.1 mmol) were dissolved in dichloromethane (3 mL). The colour of the solution immediately changed to red. After 2 h of stirring the solvent was removed in vacuo to yield 79 mg (98% 0.098 mmol) of [Co(tfac)$_2$(L1)] as a red solid. Crystals suitable for X-ray analysis were obtained through overlayering a saturated CH_2Cl_2 solution with pentane.

Elemental analysis ($C_{28}H_{36}CoN_6F_{12}O_4$):

calcd.:	C 41.85%	H 4.01%	N 10.02%
found:	C 42.38%	H 4.51%	N 9.36%

UV-vis (CH_3CN): λ_{max} (ε / M^{-1} cm^{-1}): 249 ($1.91 \cdot 10^4$), 336 ($1.87 \cdot 10^4$), 465 (304), 575 (109) nm.

ESI-HRMS (pos., CH_2Cl_2): *m/z* (M-2hfac+H) = calcd. 333.2761, found 333.2765.

CV (CH_2Cl_2, Ag/AgCl, $^nBu_4NPF_6$ as supporting electrolyte, scan speed 30 mV s^{-1}): $E_{1/2}$ = 0.07, 0.52 V vs. ferrocenium/ferrocene.

2.2.2 Neutral cobalt complexes of ligand L2

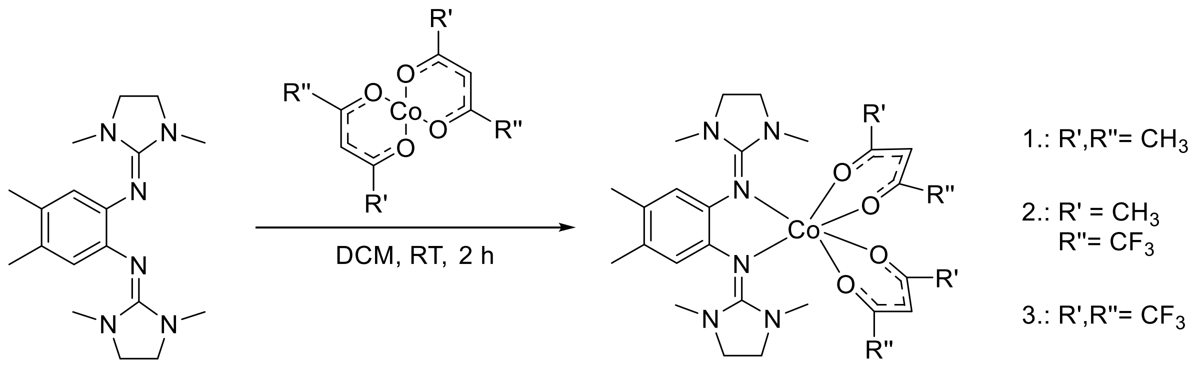

[Co(acac)₂L2]

Ligand L2 (32.8 mg, 0.1 mmol) and [Co(acac)₂] (25.7 mg, 0.1 mmol) were dissolved in dichloromethane (3 mL). The red solution was stirred at room temperature for a period of 2 h. Then, the solvent was removed in vacuo to yield 57 mg (96%, 0.096 mmol) of [Co(acac)₂(L2)] as a dark red solid. Crystals suitable for X-ray analysis were obtained through overlayering a saturated CH_2Cl_2 solution with pentane.

Elemental analysis ($C_{28}H_{44}CoN_6O_4$):

calcd.:	C 57.23%	H 7.55%	N 14.30%
found:	C 56.84%	H 8.18%	N 14.20%

UV-vis (CH_2Cl_2): λ_{max} (ε / M^{-1} cm^{-1}): 231 ($2.05\cdot10^4$), 290 ($1.78\cdot10^4$), 564 (39) nm.

ESI-HRMS (pos., CH_2Cl_2): *m/z* (M-acac) = calcd. 486.2153, found 486.2154.

CV (CH_2Cl_2, Ag/AgCl, $^nBu_4NPF_6$ as supporting electrolyte, scan speed 30 mV s^{-1}): $E_{1/2}$ = -0.50, 0.01, 0.63 V vs. ferrocenium/ferrocene.

[Co(tfac)$_2$L2]

Ligand L2 (32.8 mg, 0.1 mmol) and [Co(tfac)$_2$] (36.7 mg, 0.1 mmol) were dissolved in dichloromethane (3 mL). The red solution was stirred at room temperature for a period of 2 h. Then, the solvent was removed in to yield 68 mg (97%, 0.097 mmol) of [Co(tfac)$_2$(L2)] as a red solid. Crystals suitable for X-ray analysis were obtained through overlayering a saturated CH_2Cl_2 solution with pentane.

Elemental analysis ($C_{28}H_{38}CoF_6N_6O_4$):

calcd.:	C 48.35%	H 5.51%	N 12.08%
found:	C 48.07%	H 6.05%	N 12.01%

UV-vis (CH_3CN): λ_{max} (ε / M^{-1} cm^{-1}): 249 ($3.75\cdot10^4$), 312 ($3.68\cdot10^4$), 560 (156) nm.

ESI-HRMS (pos., CH_2Cl_2): *m/z* (M)= calcd. 693.2034, found 693.2038.

CV (CH_2Cl_2, Ag/AgCl, $^nBu_4NPF_6$ as supporting electrolyte, scan speed 30 mV s^{-1}): $E_{1/2}$ = -0.23, 0.28, 0.83 V vs. ferrocenium/ferrocene.

[Co(hfac)$_2$L2]

Ligand L2 (32.8 mg, 0.1 mmol) and [Co(hfac)$_2$] (47.5 mg, 0.1 mmol) were dissolved in dichloromethane (3 mL). The red solution was stirred at room temperature for a period of 2 h. Then, the solvent was removed in vacuo to yield 79 mg (98% 0.098 mmol) of [L2Co(tfac)$_2$] as a red solid.

Elemental analysis ($C_{28}H_{32}CoF_{12}N_6O_4$):

calcd.:	C 41.85%	H 4.01%	N 10.02%
found:	C 41.65%	H 4.49%	N 10.41%

UV-vis (CH_3CN): λ_{max} (ε / M^{-1} cm^{-1}): 249 ($1.91\cdot10^4$), 336 ($1.87\cdot10^4$) nm (broad weak absorption in the visible region).

ESI-HRMS (pos., CH_2Cl_2): *m/z* (M)= calcd. 801.1469, found 801.1461.

CV (CH_2Cl_2, Ag/AgCl, $^{n}Bu_4NPF_6$ as supporting electrolyte, scan speed 30 mV s^{-1}): $E_{1/2}$ = -0.14, 0.44 V vs. ferrocenium/ferrocene.

2.2.3 Neutral cobalt complexes of ligand L3

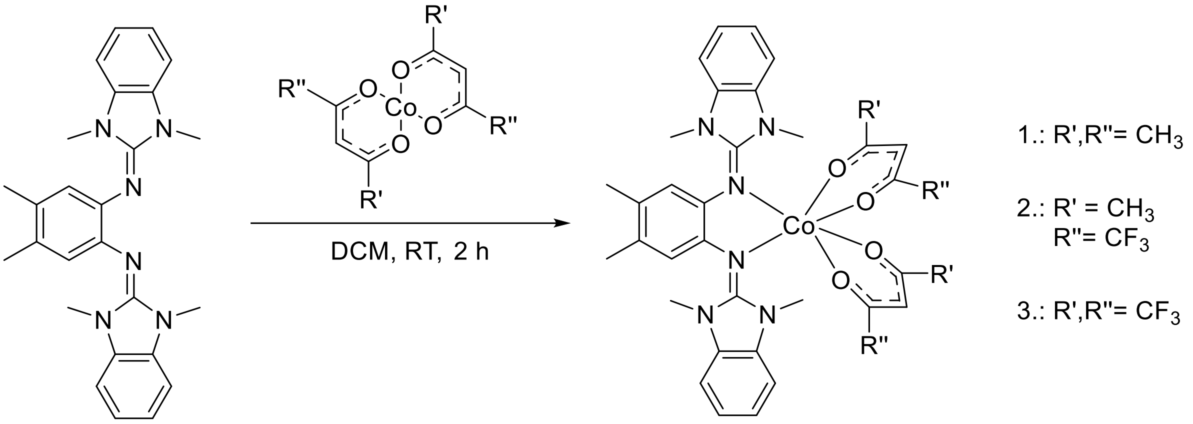

[Co(acac)₂(L3)]

Ligand L3 (32.8 mg, 0.1 mmol) and $Co(acac)_2$ (25.7 mg, 0.1 mmol) was dissolved in dichloromethane (3 mL). The red solution was stirred at room temperature for a period of 2 h. Then, the solvent was removed in vacuo to yield 57 mg (96%, 0.096 mmol) of $[Co(acac)_2(L3)]$ as a dark red solid. Crystals suitable for X-ray analysis were obtained through overlayering a saturated CH_2Cl_2 solution with pentane.

Elemental analysis ($C_{36}H_{42}CoN_6O_4$ +DCM):

calcd.:	C 57.52%	H 6.52%	N 10.88%
found:	C 58.65%	H 5.99%	N 11.50%

UV-vis (CH_3CN): λ_{max} (ε / M^{-1} cm^{-1}): 212 ($2.86\cdot10^4$), 271 ($0.92\cdot10^4$), 309 ($0.98\cdot10^4$) 324 ($1.01\cdot10^4$), 560 (21) nm.

ESI-HRMS (pos., CH_2Cl_2): *m/z* (M-acac) = calcd. 582.2153, found 582.2151.

CV (CH_2Cl_2, Ag/AgCl, $^{n}Bu_4NPF_6$ as supporting electrolyte, scan speed 30 mV s^{-1}): $E_{1/2}$ = -0.20, 0.04,0.67 V vs. ferrocenium/ferrocene.

[Co(tfac)$_2$(L3)]

Ligand L3 (32.8 mg, 0.1 mmol) and Co(tfac)$_2$ (36.7 mg, 0.1 mmol) were dissolved in dichloromethane (3 mL). The red solution was stirred at room temperature for a period of 2 h. Then, the solvent was removed in to yield 68 mg (97%, 0.097 mmol) of [Co(tfac)$_2$(L3)] as a red solid.

Elemental analysis ($C_{36}H_{36}CoF_6N_6O_4$):

calcd.:	C 54.34%	H 5.32%	N 10.56%
found:	C 55.99%	H 5.01%	N 11.45%

UV-vis (CH_3CN): λ_{max} (ε / M^{-1} cm^{-1}): 211 ($1.60 \cdot 10^4$), 305 ($0.61 \cdot 10^4$), 330 ($0.63 \cdot 10^4$) nm (broad, weak absorption in the region 400-600 nm).

ESI-HRMS (pos., CH_2Cl_2): *m/z* (M)= calcd. 789.2034, found 789.2036.

CV (CH_2Cl_2, Ag/AgCl, $^nBu_4NPF_6$ as supporting electrolyte, scan speed 30 mV s^{-1}): $E_{1/2}$ = -0.21, 0.31, 0.81 V vs. ferrocenium/ferrocene.

[Co(hfac)$_2$(L3)]

Ligand L3 (32.8 mg, 0.1 mmol) and Co(hfac)$_2$ (47.5 mg, 0.1 mmol) were dissolved in dichloromethane (3 mL). The red solution was stirred at room temperature for a period of 2 h. Then, the solvent was removed in to yield 78 mg (97%, 0.097 mmol) of [Co(hfac)$_2$(L3)] as a red solid.

Elemental analysis ($C_{36}H_{30}CoF_{12}N_6O_4$ + 2DCM):

calcd.:	C 42.76%	H 3.21%	N 7.87%
found:	C 41.76%	H 3.49%	N 7.13%

UV-vis (CH_3CN): λ_{max} (ε / M^{-1} cm^{-1}): 205 ($6.15 \cdot 10^4$), 319 ($2.97 \cdot 10^4$), 484 (543), 570 (287) nm.

ESI-HRMS (pos., CH_2Cl_2): *m/z* (M-acac) = calcd. 897.1469, found 897.1461.

CV (CH_2Cl_2, Ag/AgCl, $^nBu_4NPF_6$ as supporting electrolyte, scan speed 30 mV s^{-1}): $E_{1/2}$ = -0.10, 0.48 V vs. ferrocenium/ferrocene.

2.2.3 Neutral cobalt complexes of ligand L4

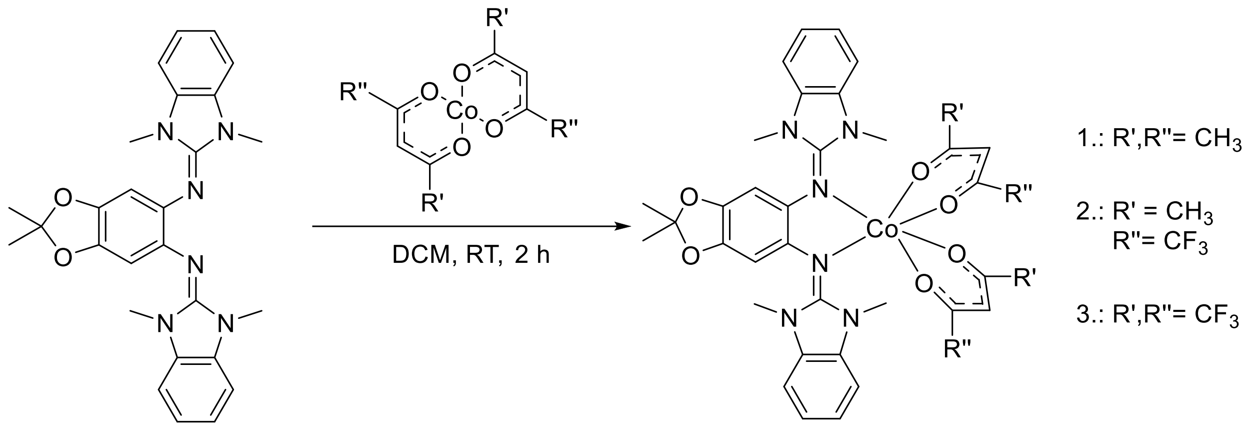

[Co(acac)$_2$(L4)]

Ligand L4 (46.8 mg, 0.1 mmol) and Co(acac)$_2$ (25.7 mg, 0.1 mmol) were dissolved in dichloromethane (3 mL). The red solution was stirred at room temperature for a period of 2 h. Then, the solvent was removed in vacuo to yield 70 mg (96%, 0.096 mmol) of [Co(acac)$_2$(L4)] as a dark red solid. Crystals suitable for X-ray analysis were obtained through overlayering a saturated CH_2Cl_2 solution with pentane.

Elemental analysis ($C_{37}H_{42}CoN_6O_6$ + 0.5DCM):

calcd.:	C 58.18%	H 6.38%	N 10.86%
found:	C 57.63%	H 6.02%	N 11.07%

UV-vis (CH_3CN): λ_{max} (ε / M^{-1} cm^{-1}): 204 ($4.51 \cdot 10^4$), 292 ($2.00 \cdot 10^4$), 420 ($5.91 \cdot 10^3$) nm.

ESI-HRMS (pos., CH_2Cl_2): *m/z* (M-acac) = calcd. 725.2498, found 725.2491.

CV (CH_2Cl_2, Ag/AgCl, $^nBu_4NPF_6$ as supporting electrolyte, scan speed 30 mV s^{-1}): $E_{1/2}$ = -0.35, -0.09, 0.67 V vs. ferrocenium/ferrocene.

[Co(tfac)$_2$(L4)]:

Ligand L4 (46.8 mg, 0.1 mmol) and Co(tfac)$_2$ (36.7 mg, 0.1 mmol) were dissolved in dichloromethane (3 mL). The red solution was stirred at room temperature for a period of 2 h. Then, the solvent was removed in vacuo to yield 81 mg (97%, 0.097 mmol) of [Co(tfac)$_2$(L4)] as a red solid.

Elemental analysis ($C_{37}H_{36}CoF_6N_6O_6$):

calcd.:	C 52.92%	H 5.04%	N 10.01%
found:	C 53.58%	H 4.89%	N 10.20%

UV-vis (CH_3CN): λ_{max} (ε / M^{-1} cm^{-1}): 204 ($5.21 \cdot 10^4$), 290 ($2.32 \cdot 10^4$), 363 ($1.03 \cdot 10^4$), 420 ($5.14 \cdot 10^3$) nm.

ESI-HRMS (pos., CH_2Cl_2): *m/z* (M-acac$^-$) = calcd. 833.1932, found 833.1931.

CV (CH_2Cl_2, Ag/AgCl, $^n Bu_4NPF_6$ as supporting electrolyte, scan speed 30 mV s^{-1}): $E_{1/2}$ = -0.46, -0.17, 0.39, 1.00 V vs. ferrocenium/ferrocene.

[Co(hfac)$_2$(L4)]

Ligand L4 (46.8 mg, 0.1 mmol) and Co(hfac)$_2$ (47.5 mg, 0.1 mmol) were dissolved in dichloromethane (3 mL). in vacuo. The red solution was stirred at room temperature for a period of 2 h. Then, the solvent was removed in to yield 91 mg (97%, 0.097 mmol) of [Co(hfac)$_2$(L4)] as a red solid. Crystals suitable for X-ray analysis were obtained through overlayering a saturated CH_2Cl_2 solution with pentane.

Elemental analysis ($C_{37}H_{30}CoF_{12}N_6O_6$):

calcd.:	C 47.20%	H 3.21%	N 8.93%
found:	C 47.76%	H 3.49%	N 8.13%

UV-vis (CH_3CN): λ_{max} (ε / M^{-1} cm^{-1}): 204 ($5.68 \cdot 10^4$), 310 ($2.25 \cdot 10^4$), 349 ($2.17 \cdot 10^4$), 501 (673), 565 (547) nm.

ESI-HRMS (pos., CH_2Cl_2): *m/z* (M) = calcd. 941.1367, found 941.1361.

CV (CH_2Cl_2, Ag/AgCl, $^{n}Bu_4NPF_6$ as supporting electrolyte, scan speed 30 mV s^{-1}): $E_{1/2}$ = -0.30, 0.20 V vs. ferrocenium/ferrocene.

2.2.5 Neutral cobalt complexes of ligand L5

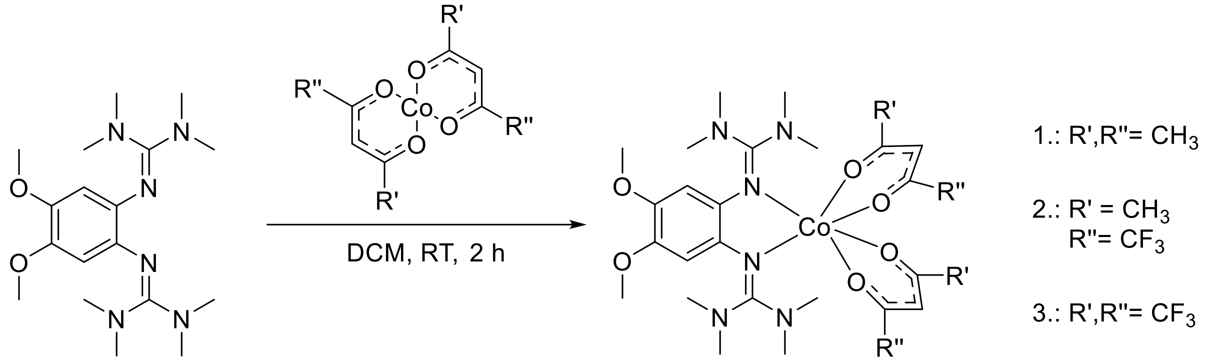

[Co(acac)$_2$(L5)]

Ligand L5 (36.4 mg, 0.1 mmol) and [Co(acac)$_2$] (25.7 mg, 0.1 mmol) were dissolved in dichloromethane (3 mL). The red solution was stirred at room temperature for a period of 2 h. Then, the solvent was removed in vacuo to yield 60 mg (97%, 0.097 mmol) of [Co(acac)$_2$(L5)] as a dark red solid. Crystals suitable for X-ray analysis were obtained through overlayering a saturated CH_2Cl_2 solution with pentane.

Elemental analysis ($C_{28}H_{46}CoN_6O_6$):

calcd.:	C 54.10%	H 7.46%	N 13.52%
found:	C 53.26%	H 7.49%	N 13.57%

UV-vis (CH_3CN): λ_{max} (ε / M^{-1} cm^{-1}): 231 ($1.36 \cdot 10^4$), 293 ($1.01 \cdot 10^4$), 461 (65), 563 (27) nm.

ESI-HRMS (pos., CH_2Cl_2): *m/z* (M^+) = calcd. 621.2811, found 621.2813.

CV (CH_2Cl_2, Ag/AgCl, $^{n}Bu_4NPF_6$ as supporting electrolyte, scan speed 30 mV s^{-1}): $E_{1/2}$ = -0.32 (-0.29/-0.35), -0.04 (0.03/-0.10), 0.40 (0.46/0.34) V vs. ferrocenium/ferrocene.

[Co(tfac)$_2$(L5)]

Ligand L5 (36.4 mg, 0.1 mmol) and [Co(tfac)$_2$] (36.7 mg, 0.1 mmol) were dissolved in dichloromethane (3 mL). The red solution was stirred at room temperature for a period of 2 h. Then, the solvent was removed in to yield 69 mg (95%, 0.095 mmol) of [Co(tfac)$_2$(L5)] as a red solid. Crystals suitable for X-ray analysis were obtained through overlayering a saturated CH_2Cl_2 solution with pentane.

Elemental analysis ($C_{28}H_{34}CoF_6N_6O_6$ + 0.5 DCM):

calcd.:	C 44.34%	H 5.35%	N 10.89%
found:	C 44.86%	H 6.08%	N 10.82%

UV-vis (CH_3CN): λ_{max} (ε / M^{-1} cm^{-1}): 230 ($3.29\cdot10^4$), 297 ($2.76\cdot10^4$), 460 (245), 568 (102) nm.

ESI-HRMS (pos., CH_2Cl_2): *m/z* (M^+) = calcd. 729.2246, found 729.2244.

CV (CH_2Cl_2, Ag/AgCl, $^nBu_4NPF_6$ as supporting electrolyte, scan speed 100 mV s^{-1}): $E_{1/2}$ = -0.29 (-0.24/-0.34), 0.06 (0.10/0.01) V vs. ferrocenium/ferrocene.

[Co(hfac)$_2$(L5)]

Ligand L5 (36.4 mg, 0.1 mmol) and [Co(hfac)$_2$] (47.5 mg, 0.1 mmol) were dissolved in dichloromethane (3 mL). The red solution was stirred at room temperature for a period of 2 h. Then, the solvent was removed in vacuo to yield 81 mg (97% 0.097 mmol) of [Co(tfac)$_2$(L5)] as a red solid. Crystals suitable for X-ray analysis were obtained from a saturated solution in pentane.

Elemental analysis ($C_{28}H_{28}CoF_{12}N_6O_6$):

calcd.:	C 40.06%	H 4.32%	N 10.01%
found:	C 40.45%	H 4.21%	N 10.38%

UV-vis (CH_3CN): λ_{max} (ε / M^{-1} cm^{-1}): 231 ($2.78\cdot10^4$), 304 ($2.74\cdot10^4$), 470 (349), 566 (190) nm.

ESI-HRMS (pos., CH_2Cl_2): *m/z* (M^+) = calcd. 837.1680, found 837.1676.

CV (CH_2Cl_2, Ag/AgCl, $^nBu_4NPF_6$ as supporting electrolyte, scan speed 100 mV s^{-1}): $E_{1/2}$ = -0.19 (-0.13/-0.25), 0.19 (0.24/0.13) V vs. ferrocenium/ferrocene.

2.2.6 Neutral cobalt complexes of ligand L6

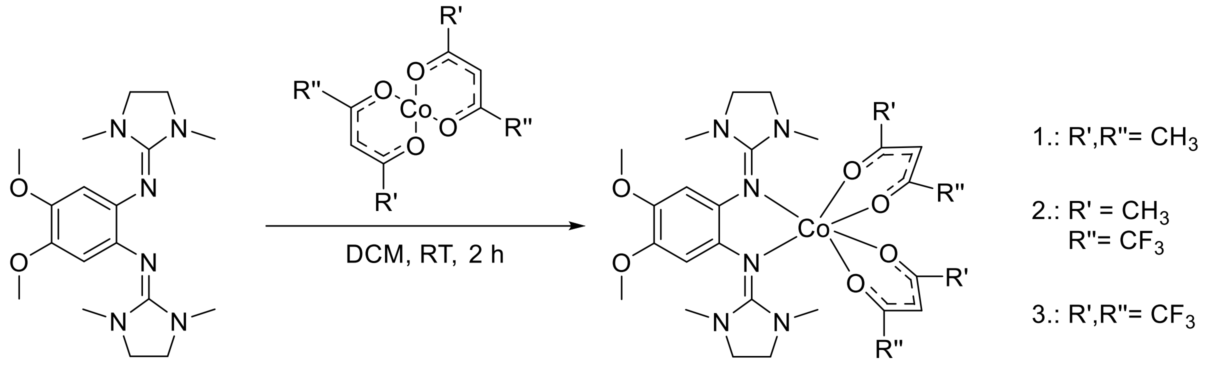

[Co(acac)$_2$(L6)]

Ligand L6 (36.0 mg, 0.1 mmol) and [Co(acac)$_2$] (25.7 mg, 0.1 mmol) were dissolved in dichloromethane (3 mL). The red solution was stirred at room temperature for a period of 2 h. Then, the solvent was removed in vacuo to yield 59 mg (95%, 0.095 mmol) of [Co(acac)$_2$(L6)] as a dark red solid. Crystals suitable for X-ray analysis were obtained through overlayering a saturated CH_2Cl_2 solution with pentane.

Elemental analysis ($C_{28}H_{42}CoN_6O_6$ + pentane + 0.5 DCM):

calcd.:	C 54.95%	H 7.57%	N 11.48%
found:	C 54.74%	H 8.19%	N 12.40%

UV-vis (CH_3CN): λ_{max} (ε / M^{-1} cm^{-1}): 215 ($2.03\cdot10^4$), 280 ($1.38\cdot10^4$) nm (weak, broad absorptions in the region 400-600 nm).

ESI-HRMS (pos., CH_2Cl_2): *m/z* ($[M\text{-}acac]^+$)= calcd. 518.2052, found 518.2046.

CV (CH_2Cl_2, Ag/AgCl, $^{n}Bu_4NPF_6$ as supporting electrolyte, scan speed 100 mV s^{-1}): $E_{1/2}$ = -0.46 (-0.33/-0.58), -0.25 (-0.15/-0.35), 0.31 (0.37/0.24) V vs. ferrocenium/ferrocene.

[Co(tfac)$_2$(L6)]

Ligand L6 (36.0 mg, 0.1 mmol) and [Co(tfac)$_2$] (36.7 mg, 0.1 mmol) were dissolved in dichloromethane (3 mL). The red solution was stirred at room temperature for a period of 2 h. Then, the solvent was removed in to yield 71 mg (98%, 0.098 mmol) of [Co(tfac)$_2$(L6)] as a red solid. Crystals suitable for X-ray analysis were obtained through overlayering a saturated CH_2Cl_2 solution with pentane.

Elemental analysis ($C_{28}H_{36}CoF_6N_6O_6$):

calcd.:	C 46.35%	H 5.00%	N 11.58%
found:	C 46.05%	H 5.15%	N 12.05%

UV-vis (CH_3CN): λ_{max} (ε / M^{-1} cm^{-1}): 221 ($2.42 \cdot 10^4$), 291 ($1.98 \cdot 10^4$), 565 (83) nm.

ESI-HRMS (pos., CH_2Cl_2): *m/z* ($[M]^+$)= calcd. 725.1933, found 725.1929.

CV (CH_2Cl_2, Ag/AgCl, $^{n}Bu_4NPF_6$ as supporting electrolyte, scan speed 100 mV s^{-1}): $E_{1/2}$ = -0.41 (-0.36/-0.46), 0.01 (0.05/-0.04), 0.75 (0.86/0.64) V vs. ferrocenium/ferrocene.

[Co(hfac)$_2$(L6)]

Ligand L6 (36.0 mg, 0.1 mmol) and [Co(hfac)$_2$] (47.5 mg, 0.1 mmol) were dissolved in dichloromethane (3 mL). The red solution was stirred at room temperature for a period of 2 h. Then, the solvent was removed in vacuo to yield 80 mg (96% 0.096 mmol) of [Co(tfac)$_2$(L6)] as a red solid. Crystals suitable for X-ray analysis were obtained through overlayering a saturated CH_2Cl_2 solution with pentane.

Elemental analysis ($C_{28}H_{30}CoF_{12}N_6O_6$ + 0.5 DCM):

calcd.:	C 40.35%	H 3.63%	N 10.08%
found:	C 41.10%	H 4.20%	N 10.03%

UV-vis (CH_3CN): λ_{max} (ε / M^{-1} cm^{-1}): 223 ($1.67 \cdot 10^4$), 303 ($1.53 \cdot 10^4$), 470 (302), 570 (176) nm.

ESI-HRMS (pos., CH_2Cl_2): *m/z* ($[M]^+$)= calcd. 833.1367, found 833.1358.

CV (CH_2Cl_2, Ag/AgCl, $^nBu_4NPF_6$ as supporting electrolyte, scan speed 100 mV s^{-1}): $E_{1/2}$ = -0.33 (-0.27/-0.39), 0.13 (0.18/0.07) V vs. ferrocenium/ferrocene.

2.2.7 Neutral cobalt complexes of ligand L7

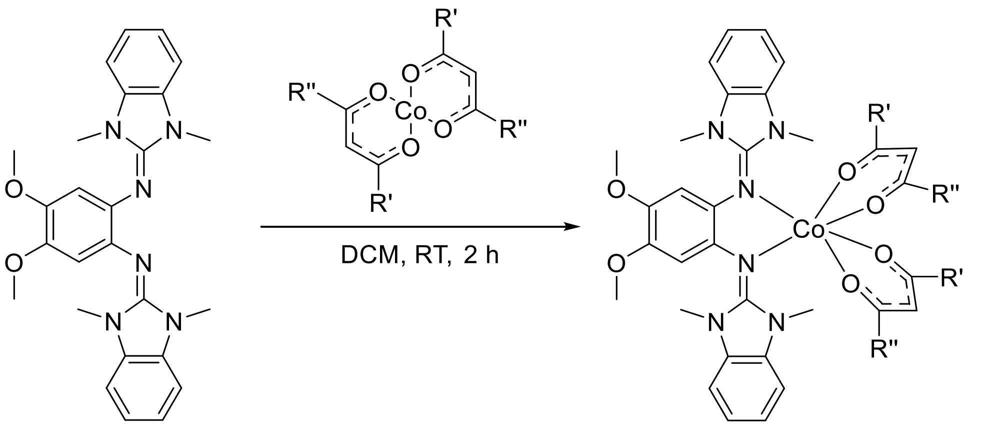

1.: R',R''= CH_3

2.: R' = CH_3
R''= CF_3

3.: R',R''= CF_3

[Co(acac)$_2$(L7)]

Ligand L7 (45.7 mg, 0.1 mmol) and [Co(acac)$_2$] (25.7 mg, 0.1 mmol) were dissolved in dichloromethane (3 mL). The red solution was stirred at room temperature for a period of 2 h. Then, the solvent was removed in vacuo to yield 70 mg (98%, 0.098 mmol) of [Co(acac)$_2$(L7)] as a dark red solid.

Elemental analysis ($C_{36}H_{42}CoN_6O_6$ + DCM):

calcd.:	C 55.65%	H 5.55%	N 10.52%
found:	C 55.97%	H 6.24%	N 9.54%

UV-vis (CH_3CN): λ_{max} (ε / M^{-1} cm^{-1}): 213 ($6.62 \cdot 10^4$), 278 ($2.40 \cdot 10^4$), shoulder at 370 ($1.21 \cdot 10^4$), 465 (63), 568 (27) nm.

ESI-HRMS (pos., CH_2Cl_2): *m/z* ($[M]^+$)= calcd. 614.2052, found 614.2050.

CV (CH_2Cl_2, Ag/AgCl, $^nBu_4NPF_6$ as supporting electrolyte, scan speed 100 mV s^{-1}): $E_{1/2}$ = -0.47 (-0.40/-0.54), -0.13 (-0.06/-0.19), 0.37 (0.42/0.31) V vs. ferrocenium/ferrocene.

[Co(tfac)$_2$(L7)]

Ligand L7 (45.7 mg, 0.1 mmol) and [Co(tfac)$_2$] (36.7 mg, 0.1 mmol) was dissolved in dichloromethane (3 mL). The red solution was stirred at room temperature for a period of 2 h. Then, the solvent was removed in to yield 79 mg (96%, 0.096 mmol) of [Co(tfac)$_2$(L7)] as a red solid. Crystals suitable for X-ray analysis were obtained through overlayering a saturated CH_2Cl_2 solution with pentane.

Elemental analysis ($C_{36}H_{36}CoF_6N_6O_6$ + 2 DCM + pentane):

calcd.:	C 48.56%	H 4.93%	N 7.90%
found:	C 47.99%	H 4.91%	N 7.99%

UV-vis (CH_3CN): λ_{max} (ε / M^{-1} cm^{-1}): 203 ($6.55\cdot10^4$), 349 ($2.35\cdot10^4$), 460 (473), 558 (151) nm.

ESI-HRMS (pos., CH_2Cl_2): *m/z* ([M]$^+$)= calcd. 821.1933, found 821.1925.

CV (CH_2Cl_2, Ag/AgCl, $^nBu_4NPF_6$ as supporting electrolyte, scan speed 100 mV s^{-1}): $E_{1/2}$ = -0.36 (-0.29/-0.42), 0.10 (0.16/0.04), 0.68 (0.77/0.58) V vs. ferrocenium/ferrocene.

[Co(hfac)$_2$(L7)]

Ligand L7 (45.7 mg, 0.1 mmol) and [Co(hfac)$_2$] (47.5 mg, 0.1 mmol) was dissolved in dichloromethane (3 mL). The red solution was stirred at room temperature for a period of 2 h. Then, the solvent was removed in vacuo to yield 91 mg (98% 0.098 mmol) of [Co(hfac)$_2$(L7)] as a red solid. Crystals suitable for X-ray analysis were obtained through overlayering a saturated CH_2Cl_2 solution with pentane.

Elemental analysis ($C_{36}H_{30}CoF_{12}N_6O_6$ + DCM + pentane):

calcd.:	C 46.42%	H 4.08%	N 7.73%
found:	C 45.65%	H 4.52%	N 7.28%

UV-vis (CH_3CN): λ_{max} (ε / M^{-1} cm^{-1}): 201 ($3.39 \cdot 10^4$), 317 ($1.37 \cdot 10^4$), 346 ($1.24 \cdot 10^4$), 500 (260), 565 (198) nm.

ESI-HRMS (pos., CH_2Cl_2): *m/z* ($[M]^+$) = calcd. 929.1367, found 929.1361.

CV (CH_2Cl_2, Ag/AgCl, $^{n}Bu_4NPF_6$ as supporting electrolyte, scan speed 100 mV s^{-1}): $E_{1/2}$ = -0.23 (-0.15/-0.31), 0.24 (0.32/0.16) V vs. ferrocenium/ferrocene.

2.3 Analytical data of neutral complexes

2.3.1 UV-Vis spectra of neutral complexes

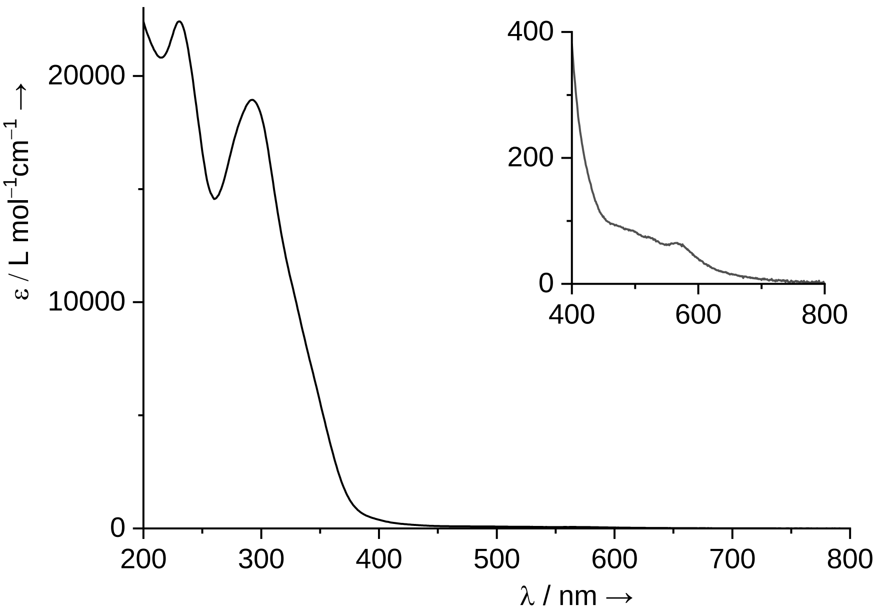

UV-vis spectrum (CH_3CN) of [Co(acac)$_2$(L1)].

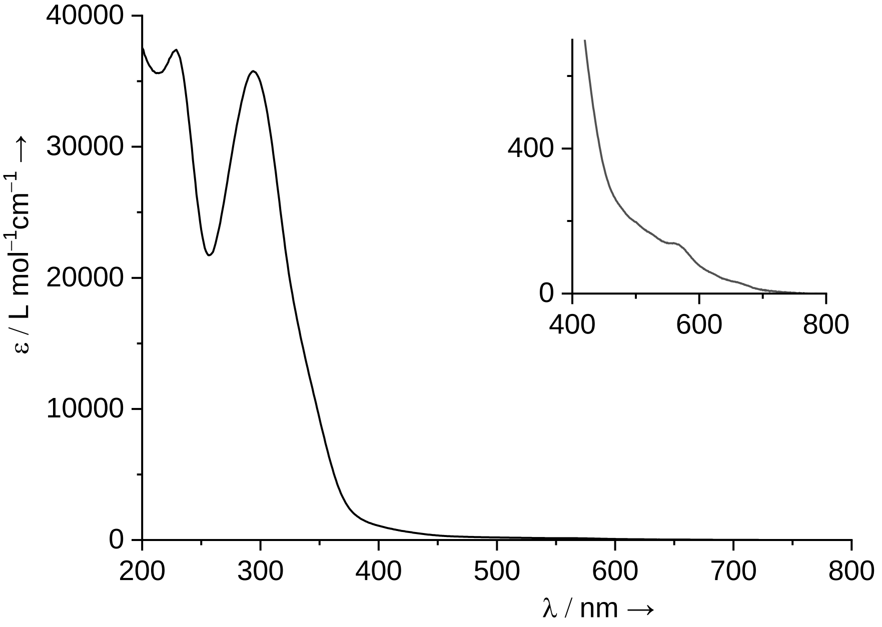

UV-vis spectrum (CH_3CN) of [Co(tfac)$_2$(L1)].

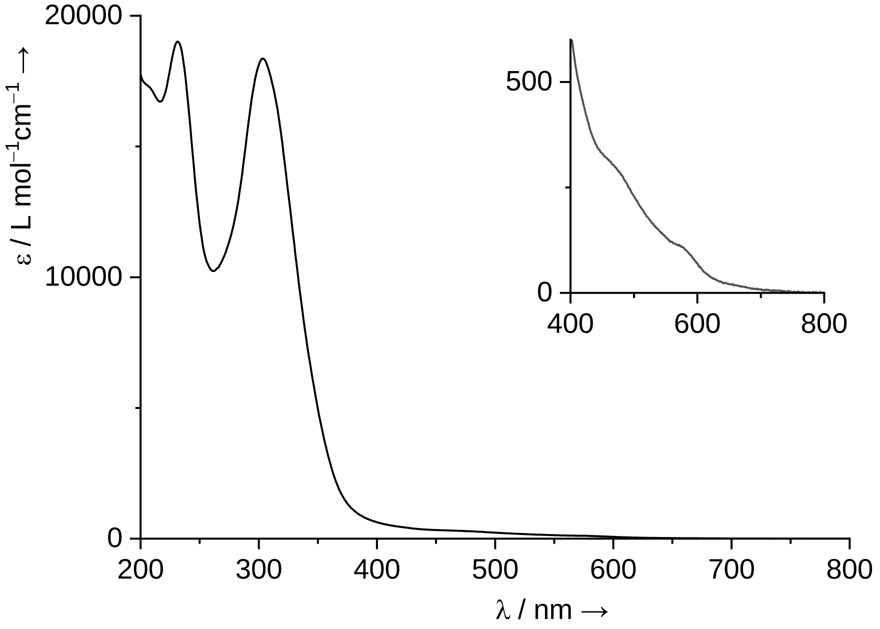

UV-vis spectrum (CH_3CN) of [Co(hfac)$_2$(L1)].

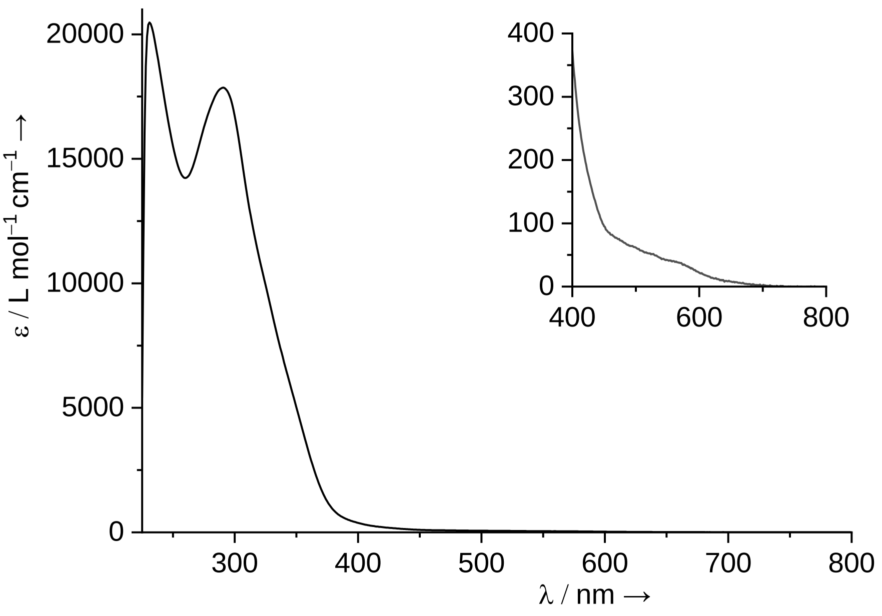

UV-vis spectrum (CH_2Cl_2) of [Co(acac)$_2$(L2)].

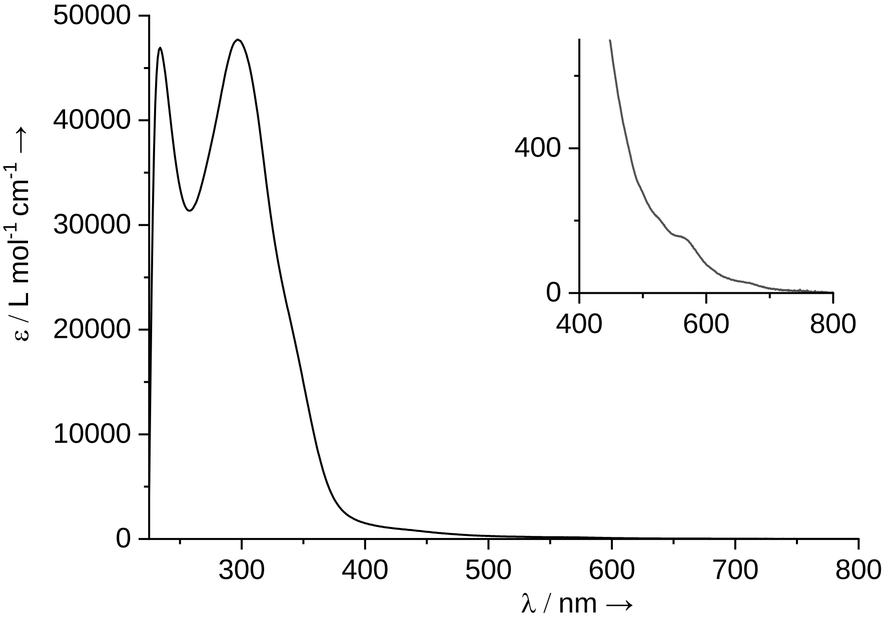

UV-vis spectrum (CH_2Cl_2) of [Co(tfac)$_2$(L2)].

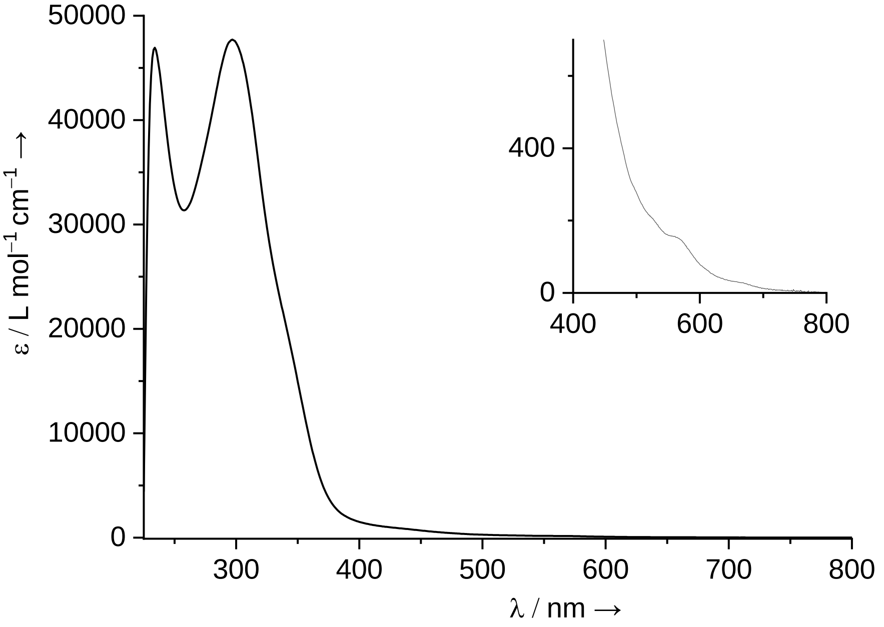

UV-vis spectrum (CH_2Cl_2) of [Co(hfac)$_2$(L2)].

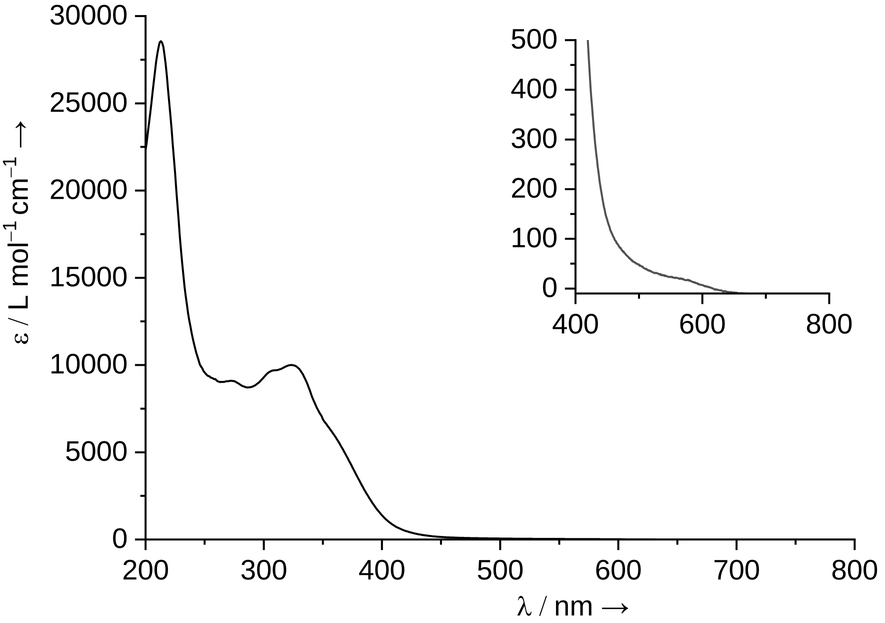

UV-vis spectrum (CH_3CN) of [Co(acac)$_2$(L3)].

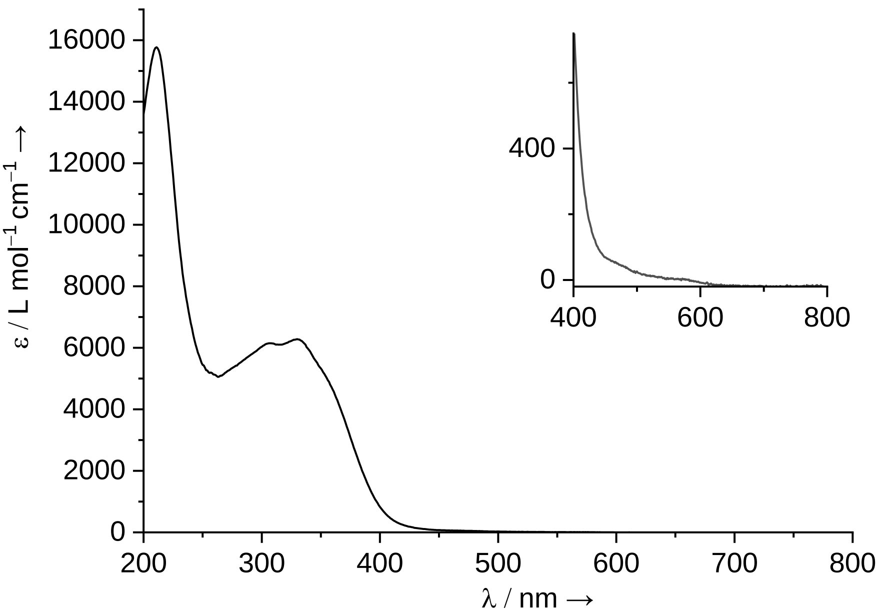

UV-vis spectrum (CH_3CN) of [Co(tfac)$_2$(L3)].

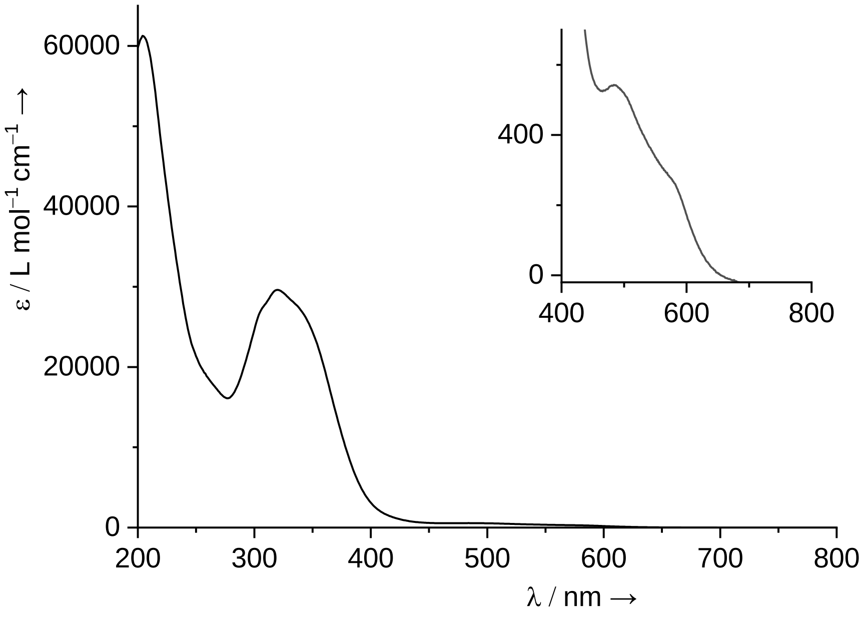

UV-vis spectrum (CH_3CN) of [Co(hfac)$_2$(L3)].

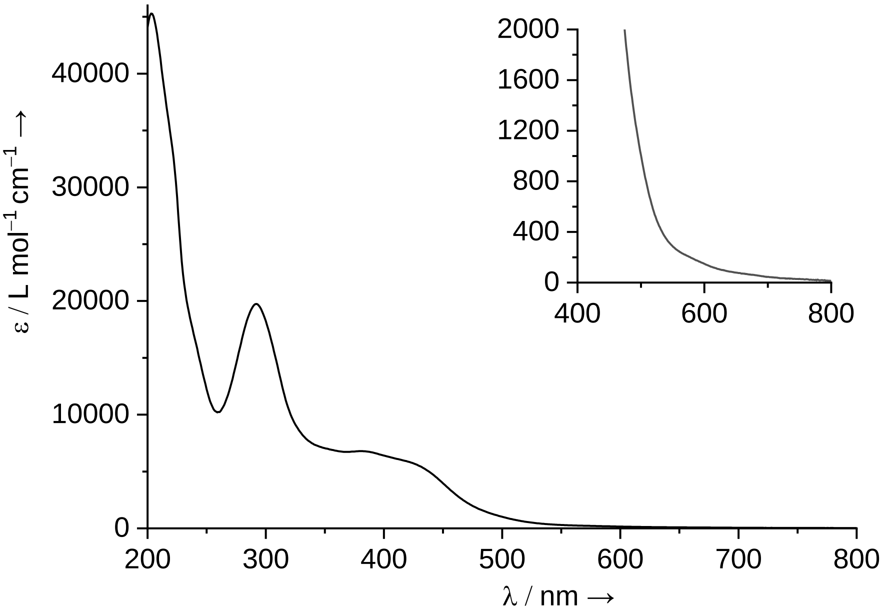

UV-vis spectrum (CH_3CN) of [Co(acac)$_2$(L4)].

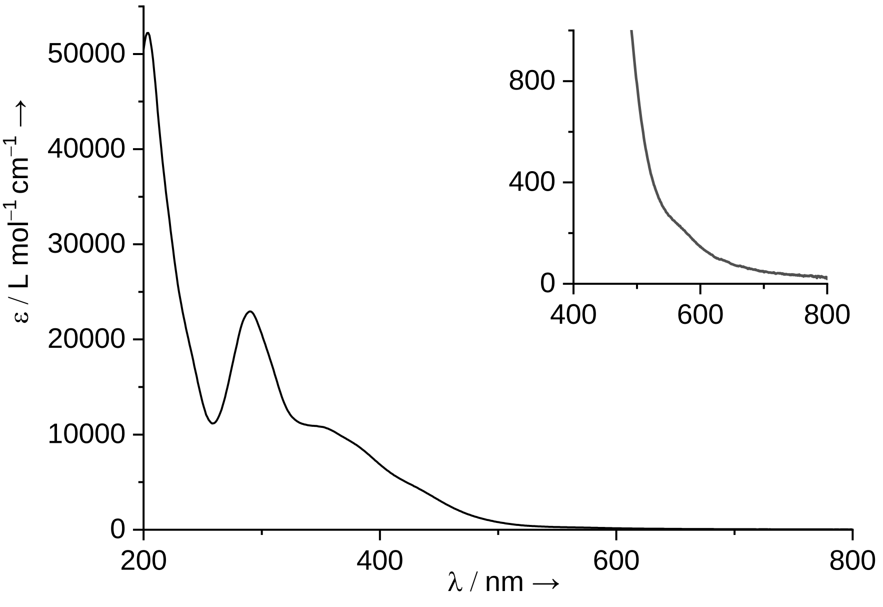

UV-vis spectrum (CH_3CN) of [Co(tfac)$_2$(L4)].

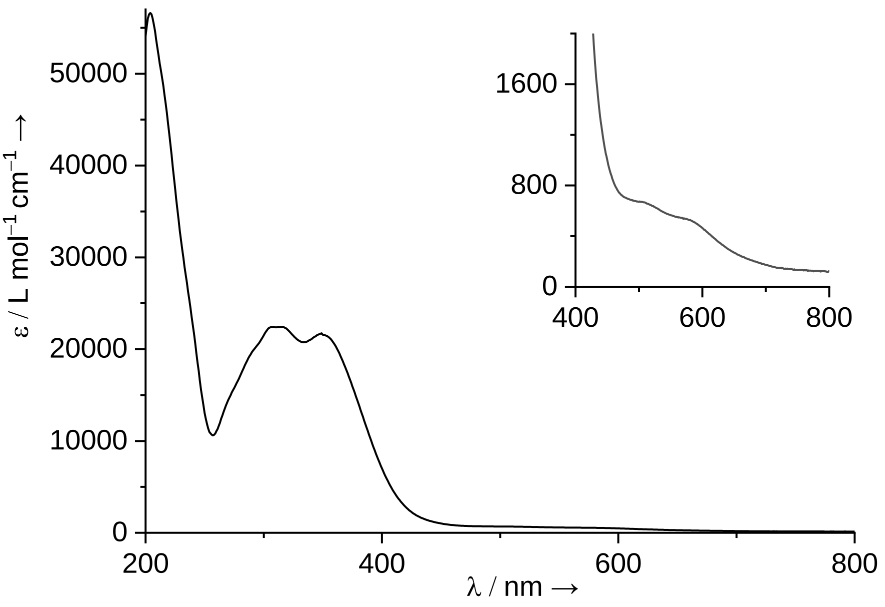

UV-vis spectrum (CH_3CN) of [Co(hfac)$_2$(L4)].

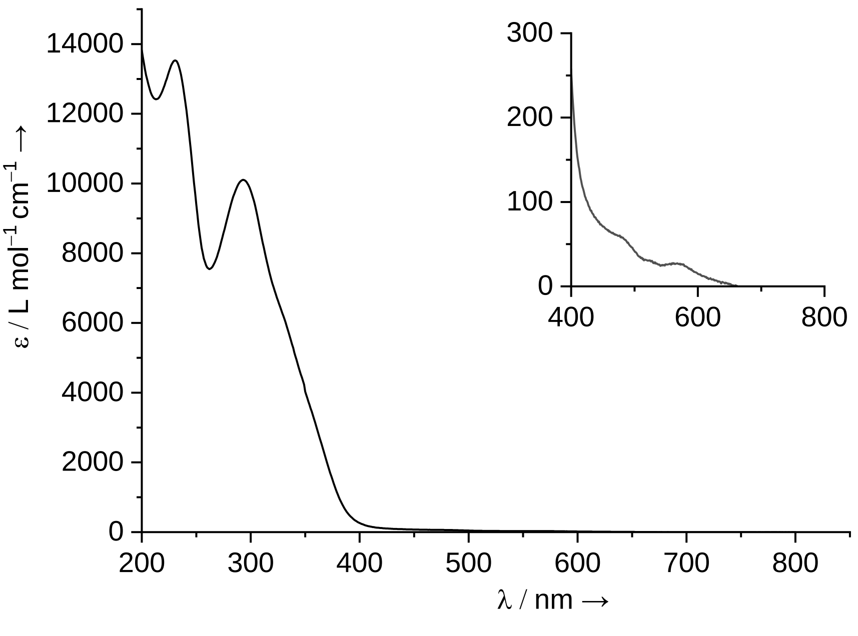

UV-vis spectrum (CH_3CN) of [Co(acac)$_2$(L5)].

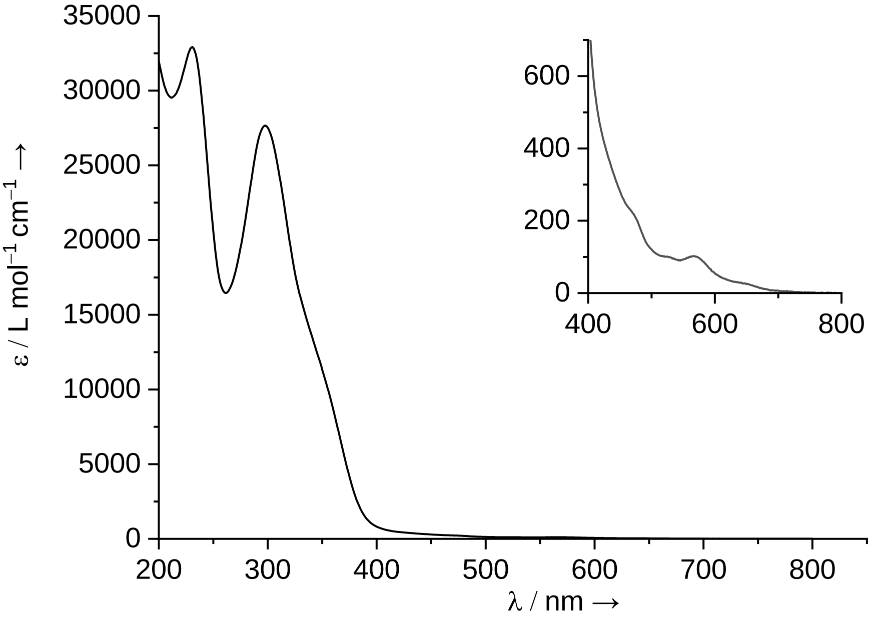

UV-vis spectrum (CH_3CN) of [Co(tfac)$_2$(L5)].

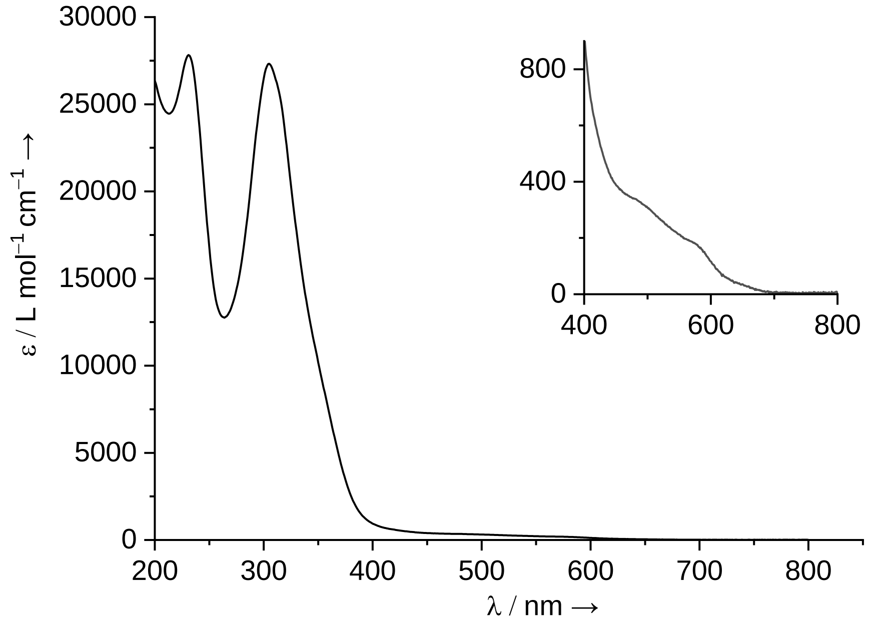

UV-vis spectrum (CH_3CN) of [Co(hfac)$_2$(L5)].

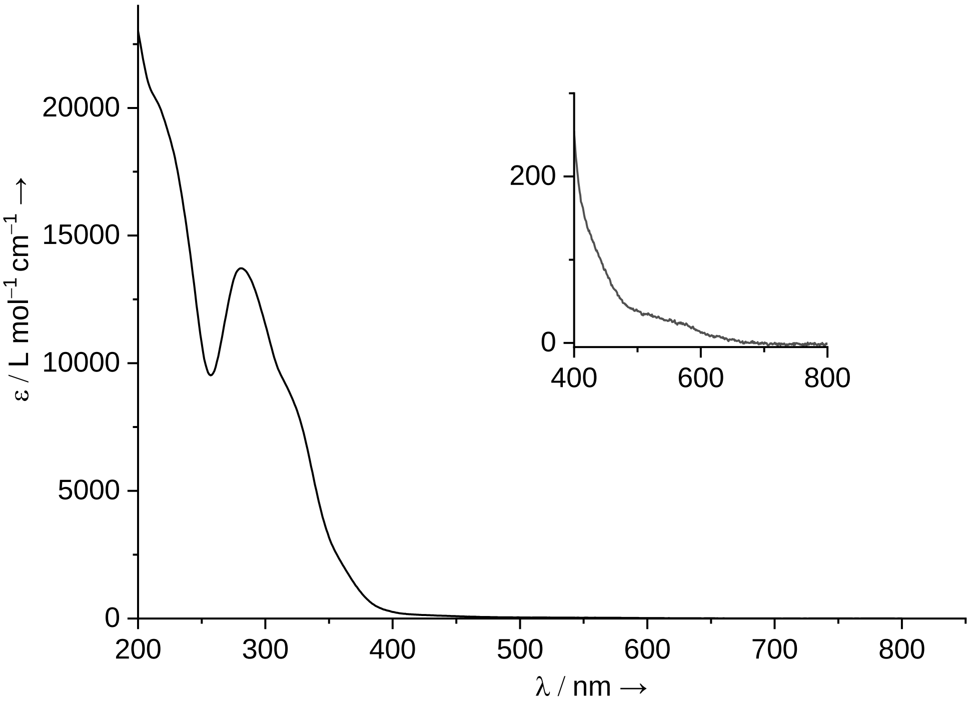

UV-vis spectrum (CH_3CN) of [Co(acac)$_2$(L6)].

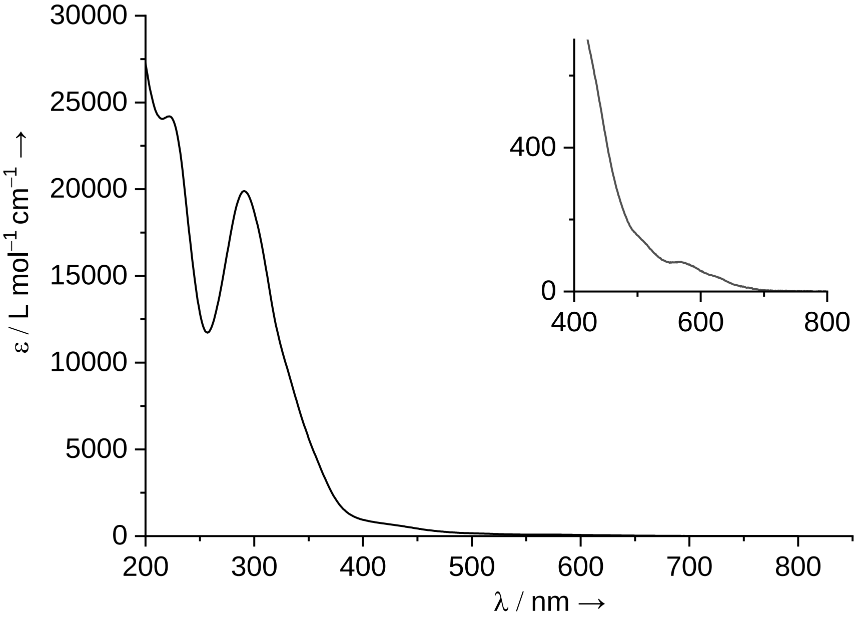

UV-vis spectrum (CH_3CN) of [Co(tfac)$_2$(L6)].

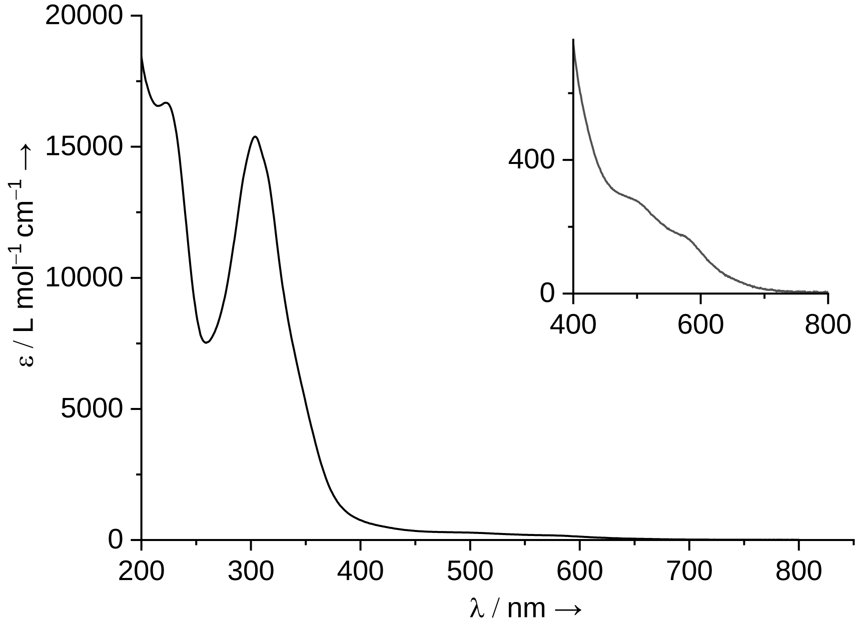

UV-vis spectrum (CH_3CN) of [Co(hfac)$_2$(L6)].

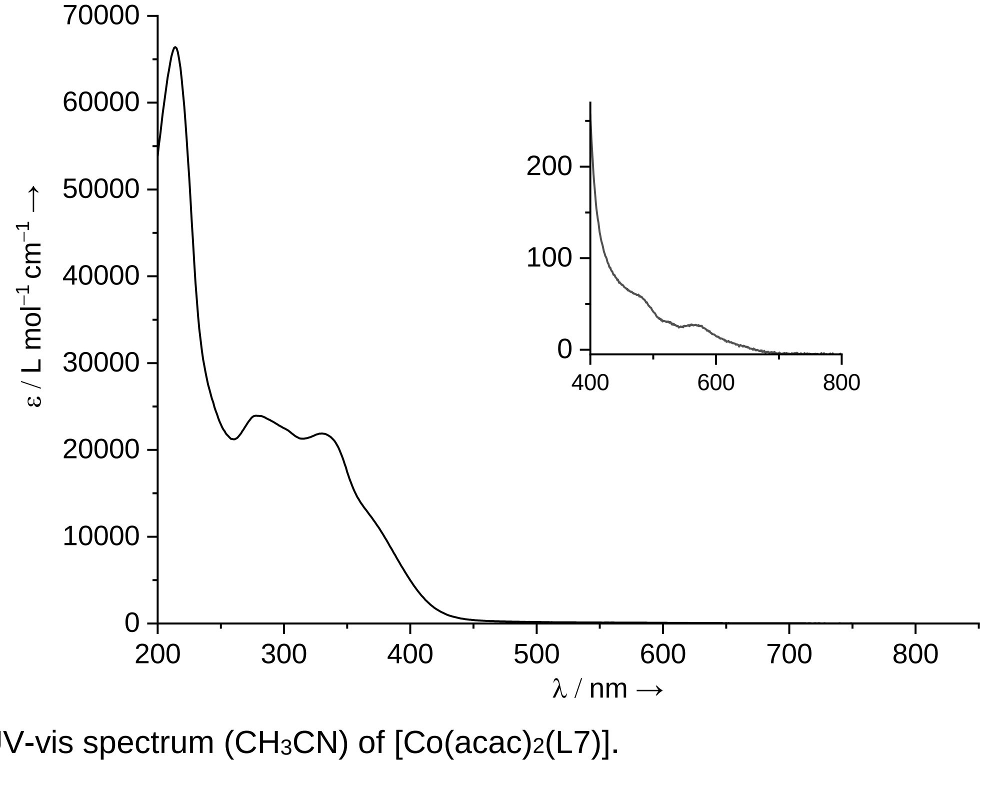

UV-vis spectrum (CH_3CN) of [Co(acac)$_2$(L7)].

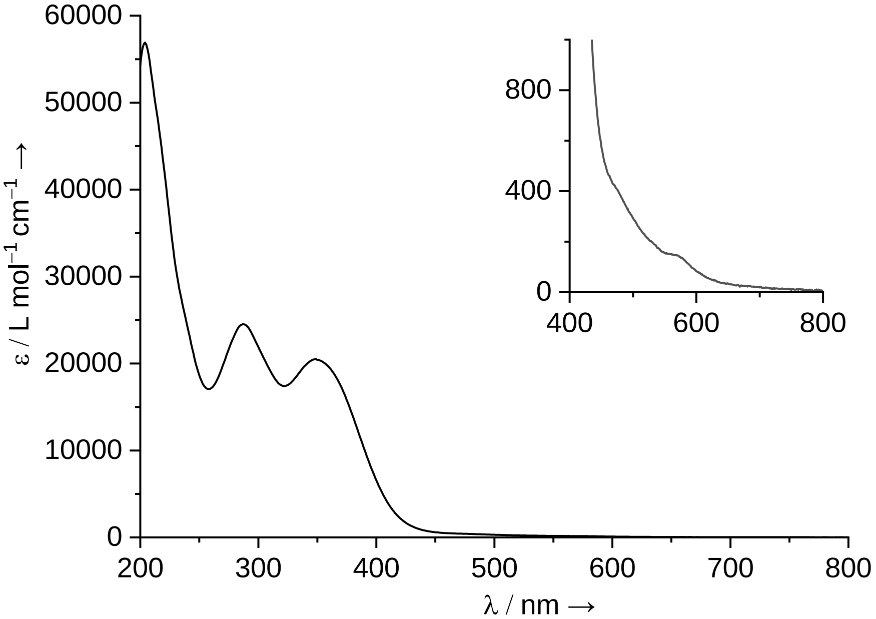

UV-vis spectrum (CH_3CN) of [Co(tfac)$_2$(L7)].

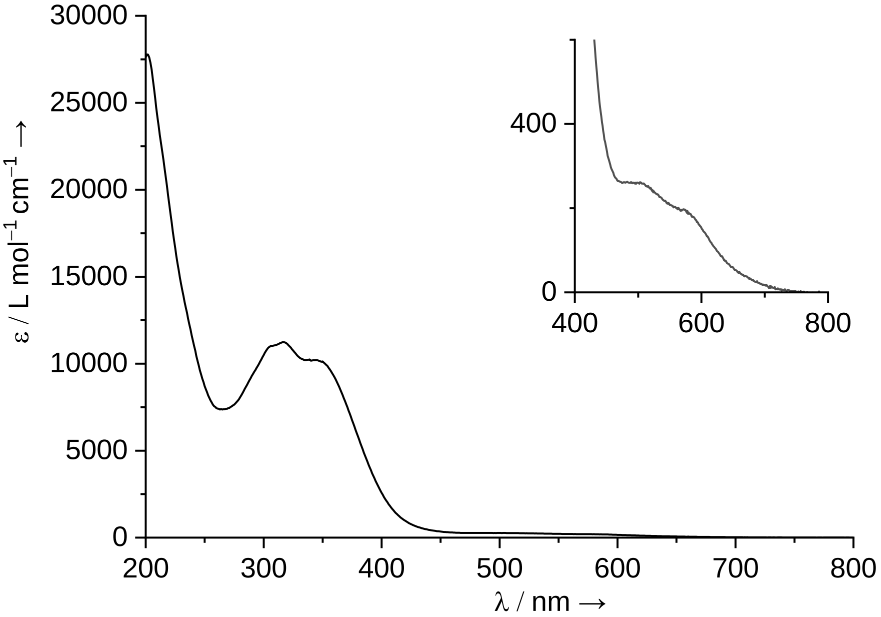

UV-vis spectrum (CH_3CN) of [Co(hfac)$_2$(L7)].

2.3.2 Cyclic Voltammetry curves of the neutral complexes

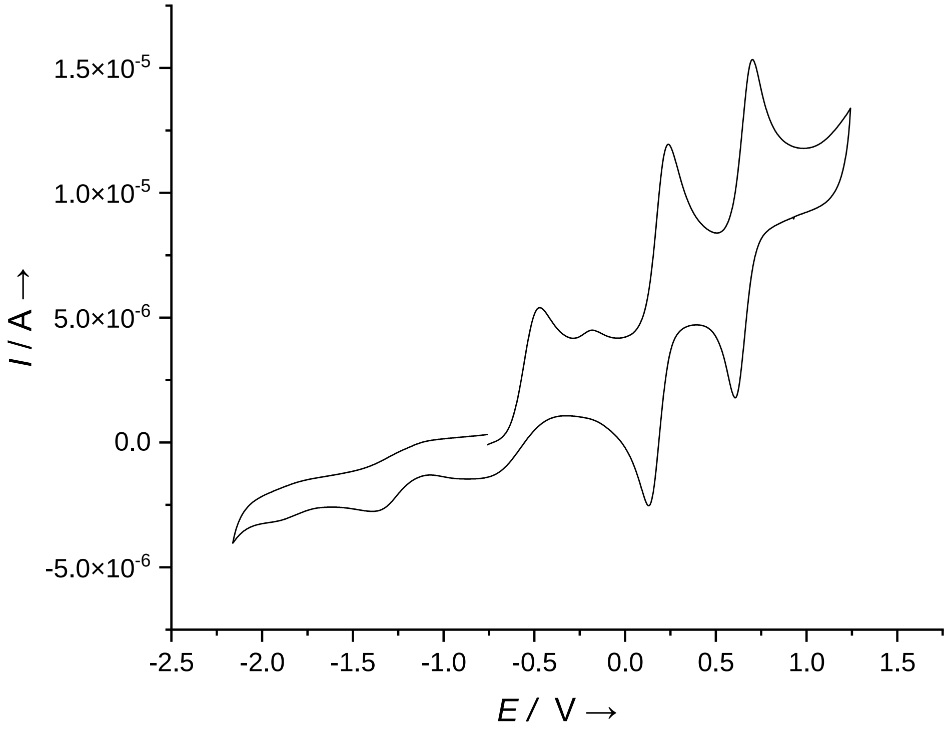

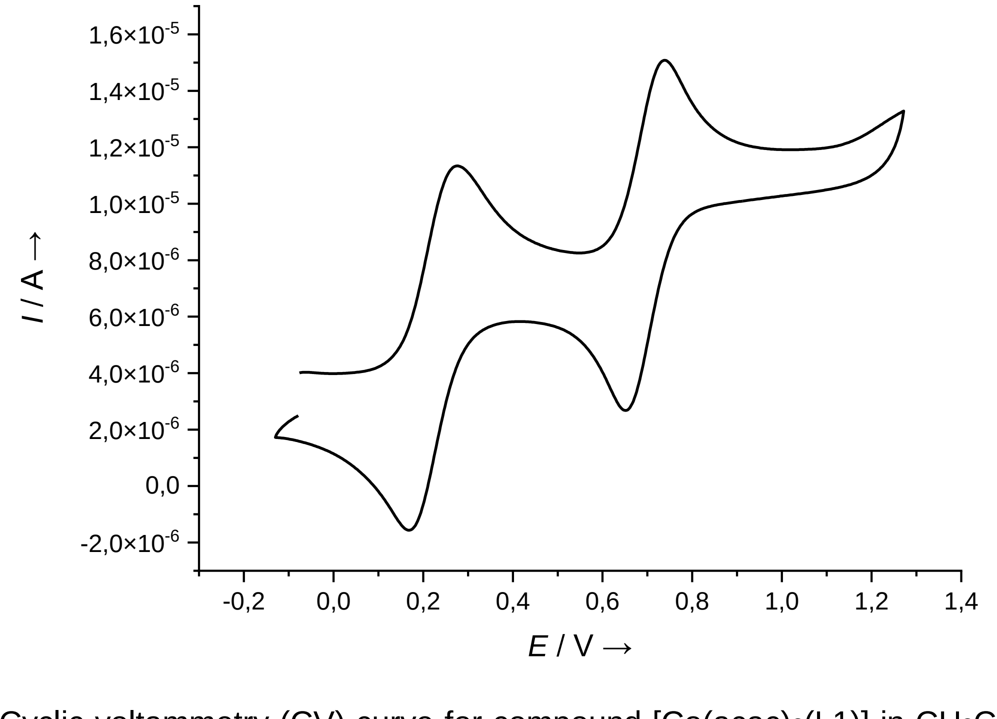

Cyclic voltammetry (CV) curve for compound [Co(acac)$_2$(L1)] in CH_2Cl_2 (Ag/AgCl reference electrode, 0.1 M N(*n*Bu)$_4$(PF$_6$) as supporting electrolyte, scan rate 30 mV s^{-1}). Potentials given vs. the Fc^+/Fc redox couple. Top: Full range curve. Bottom: Measurement in the smaller potential region.

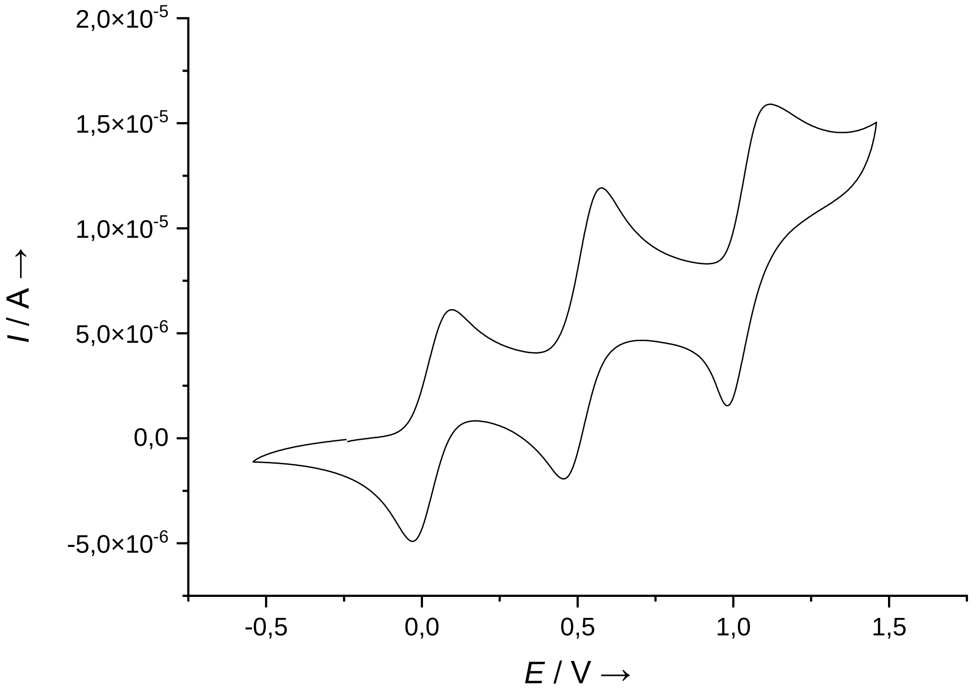

Cyclic voltammetry (CV) curve for compound [Co(tfac)$_2$(L1)] in CH_2Cl_2 (Ag/AgCl reference electrode, 0.1 M N(nBu)$_4$(PF_6) as supporting electrolyte, scan rate 30 mV s^{-1}). Potentials given vs. the Fc^+/Fc redox couple.

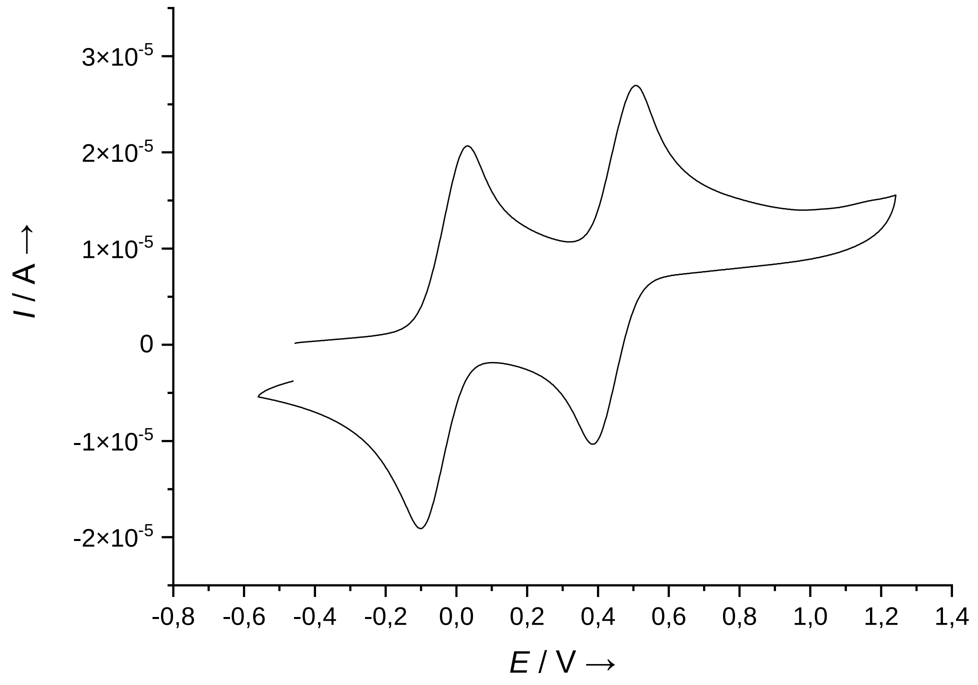

Cyclic voltammetry (CV) curve for compound [Co(hfac)$_2$(L1)] in CH_2Cl_2 (Ag/AgCl reference electrode, 0.1 M N(nBu)$_4$(PF_6) as supporting electrolyte, scan rate 30 mV s^{-1}). Potentials given vs. the Fc^+/Fc redox couple.

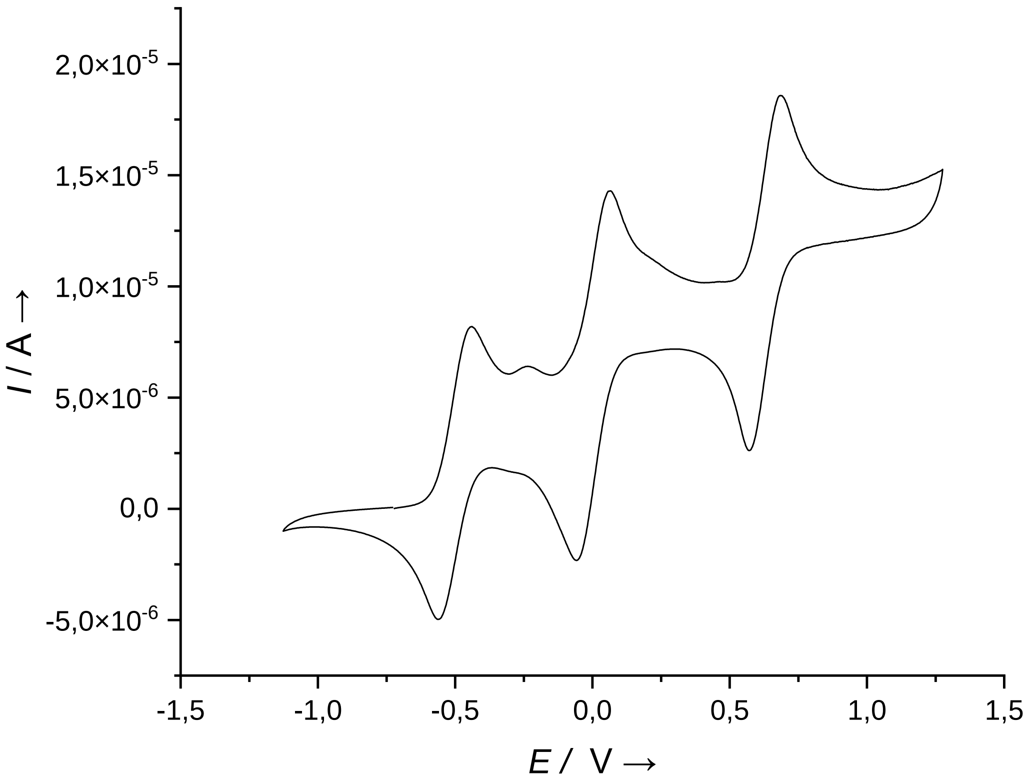

Cyclic voltammetry (CV) curve for compound [Co(acac)$_2$(L2)] in CH_2Cl_2 (Ag/AgCl reference electrode, 0.1 M N(*n*Bu)$_4$(PF$_6$) as supporting electrolyte, scan rate 30 mV s^{-1}). Potentials given vs. the Fc^+/Fc redox couple.

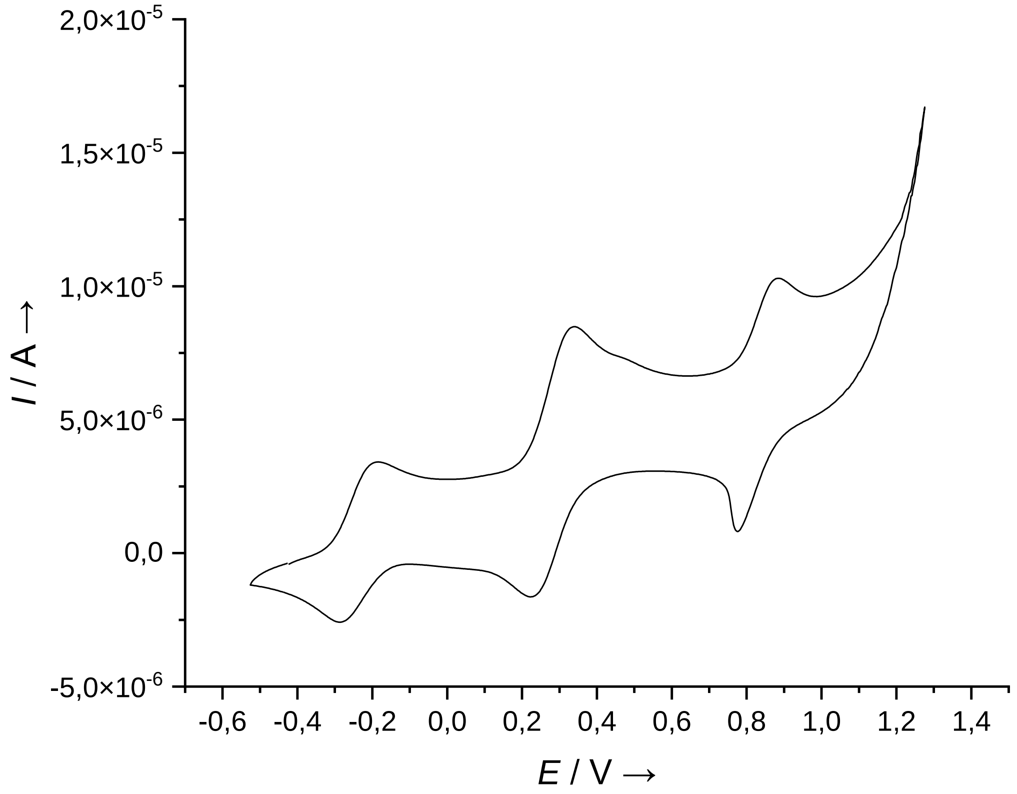

Cyclic voltammetry (CV) curve for compound [Co(tfac)$_2$(L2)] in CH_2Cl_2 (Ag/AgCl reference electrode, 0.1 M N(*n*Bu)$_4$(PF_6) as supporting electrolyte, scan rate 30 mV s^{-1}). Potentials given vs. the Fc^+/Fc redox couple.

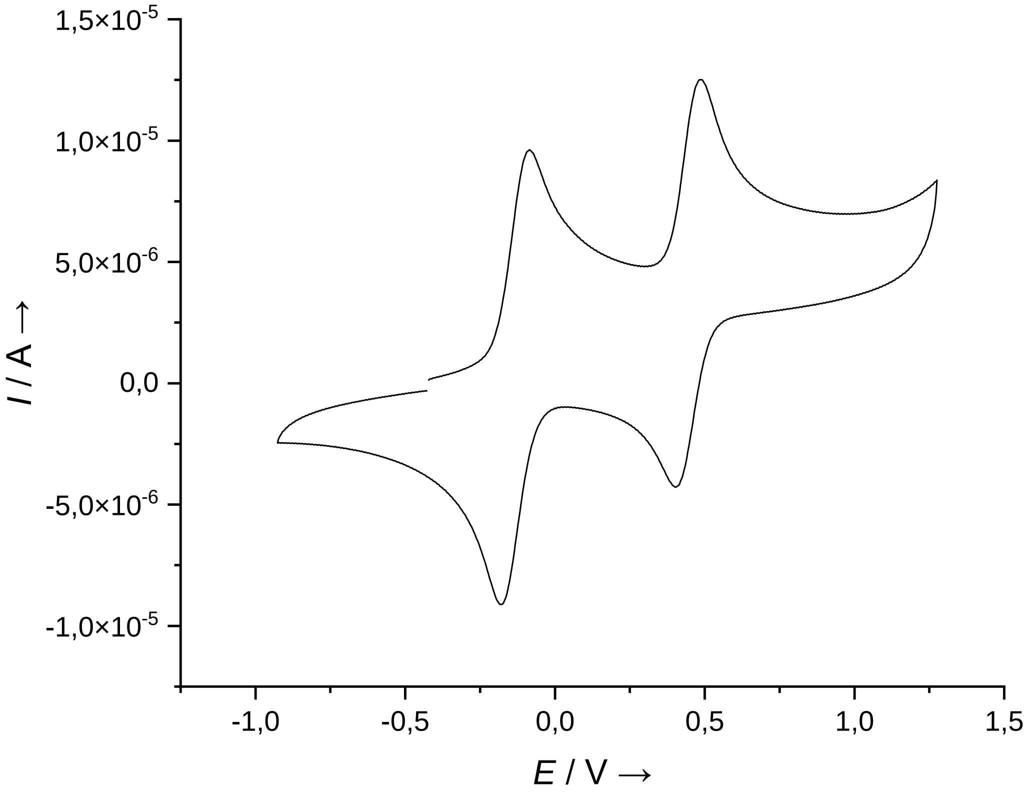

Cyclic voltammetry (CV) curve for compound [Co(hfac)$_2$(L2)] in CH_2Cl_2 (Ag/AgCl reference electrode, 0.1 M N(*n*Bu)$_4$(PF_6) as supporting electrolyte, scan rate 100 mV s^{-1}). Potentials given vs. the Fc^+/Fc redox couple.

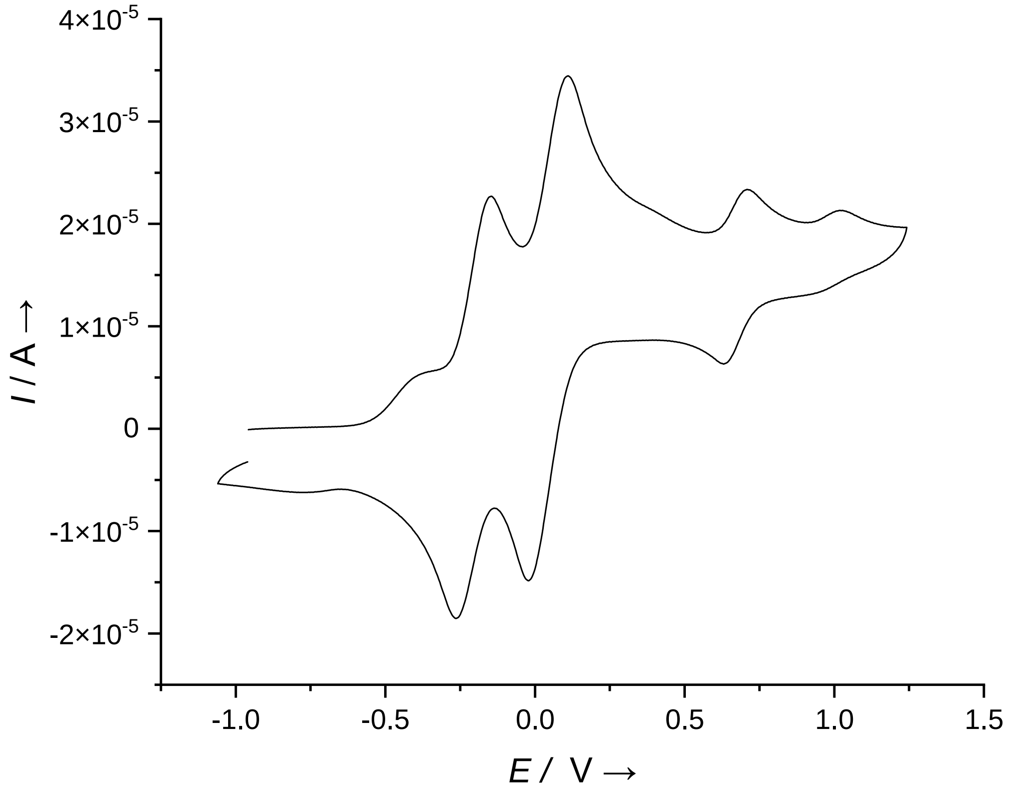

Cyclic voltammetry (CV) curve for compound [Co(acac)$_2$(L3)] in CH_2Cl_2 (Ag/AgCl reference electrode, 0.1 M N(*n*Bu)$_4$(PF$_6$) as supporting electrolyte, scan rate 100 mV s^{-1}). Potentials given vs. the Fc$^+$/Fc redox couple.

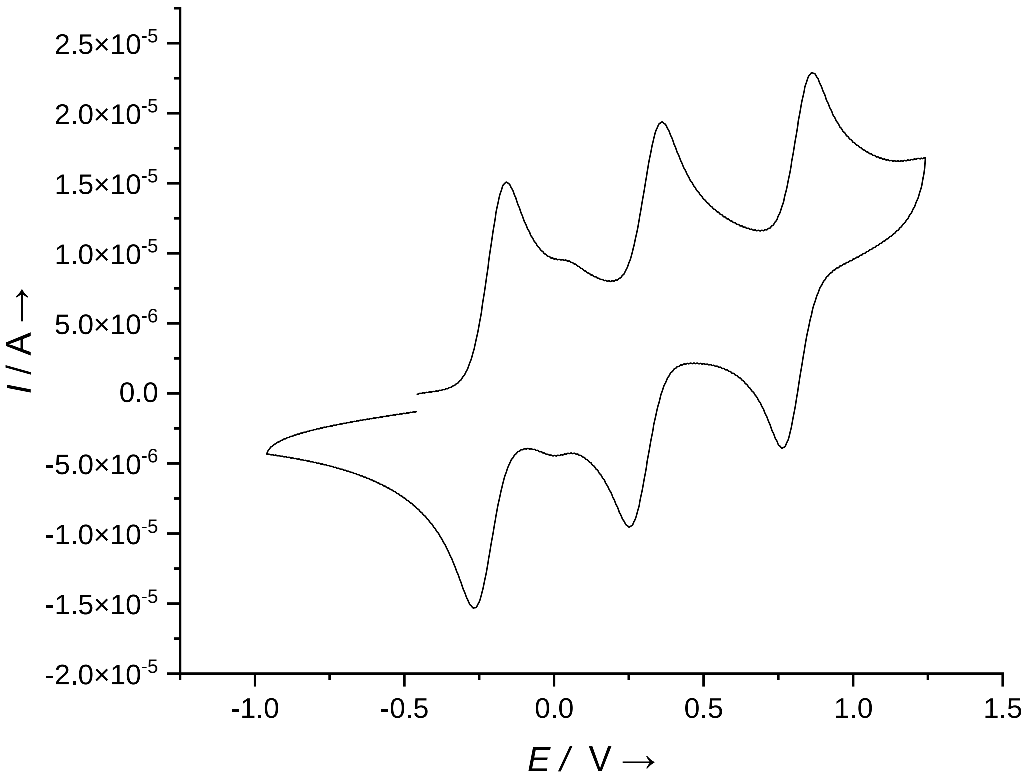

Cyclic voltammetry (CV) curve for compound [Co(tfac)$_2$(L3)] in CH_2Cl_2 (Ag/AgCl reference electrode, 0.1 M N(*n*Bu)$_4$(PF_6) as supporting electrolyte, scan rate 100 mV s^{-1}). Potentials given vs. the Fc^+/Fc redox couple.

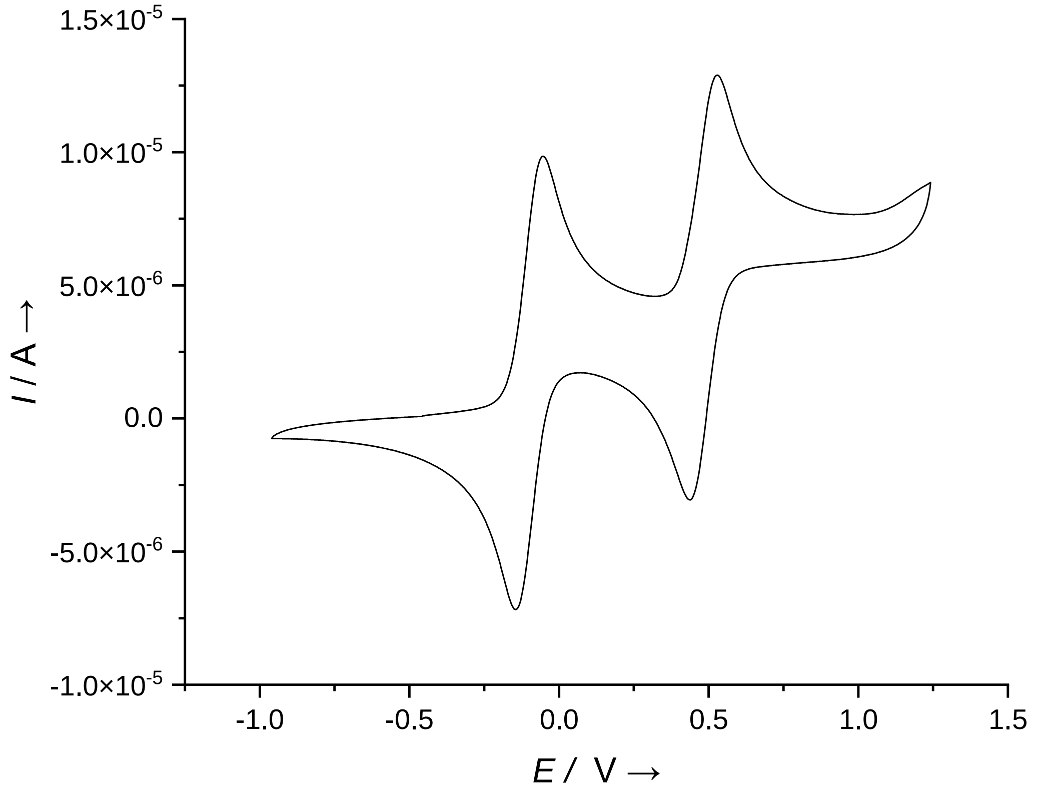

Cyclic voltammetry (CV) curve for compound [Co(hfac)$_2$(L3)] in CH_2Cl_2 (Ag/AgCl reference electrode, 0.1 M N(nBu)$_4$(PF_6) as supporting electrolyte, scan rate 100 mV s^{-1}). Potentials given vs. the Fc^+/Fc redox couple.

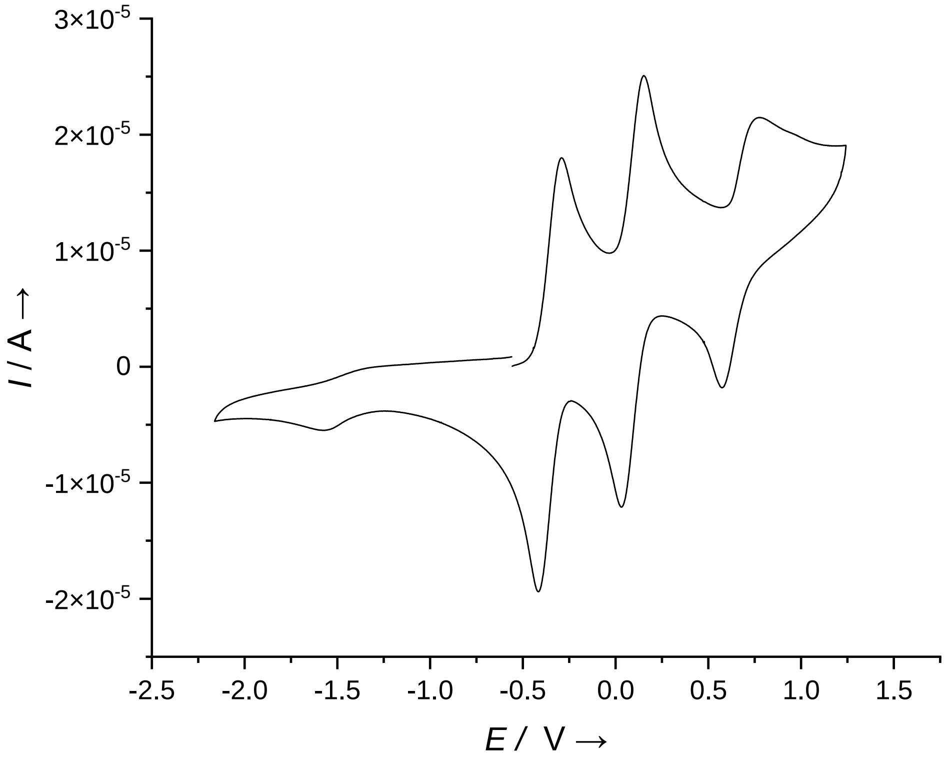

Cyclic voltammetry (CV) curve for compound [Co(acac)$_2$(L4)] in CH_2Cl_2 (Ag/AgCl reference electrode, 0.1 M N(*n*Bu)$_4$(PF_6) as supporting electrolyte, scan rate 30 mV s^{-1}). Potentials given vs. the Fc^+/Fc redox couple.

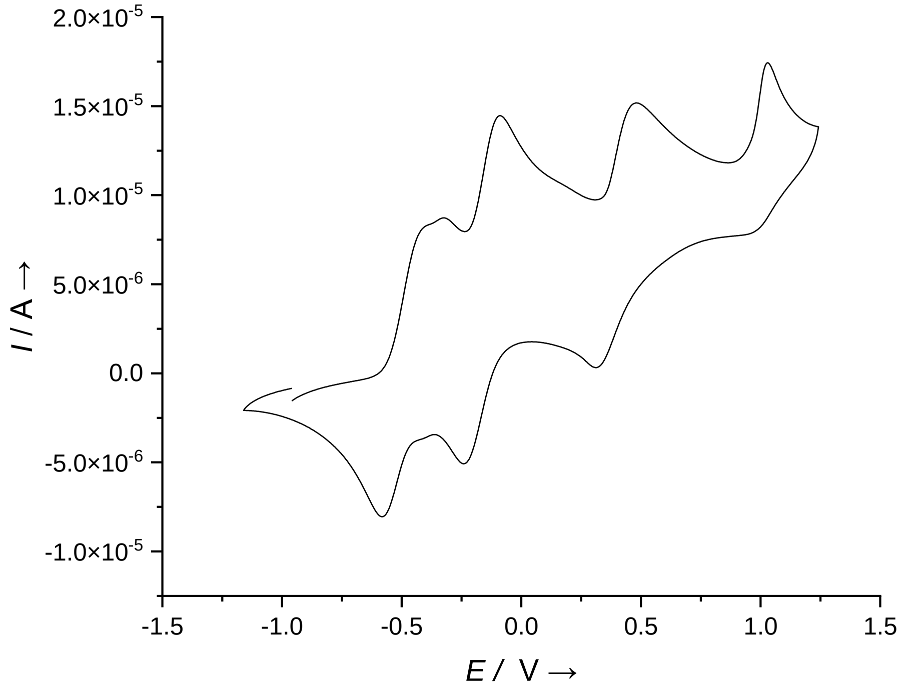

Cyclic voltammetry (CV) curve for compound [Co(tfac)$_2$(L4)] in CH_2Cl_2 (Ag/AgCl reference electrode, 0.1 M N(*n*Bu)$_4$(PF$_6$) as supporting electrolyte, scan rate 30 mV s^{-1}). Potentials given vs. the Fc^+/Fc redox couple.

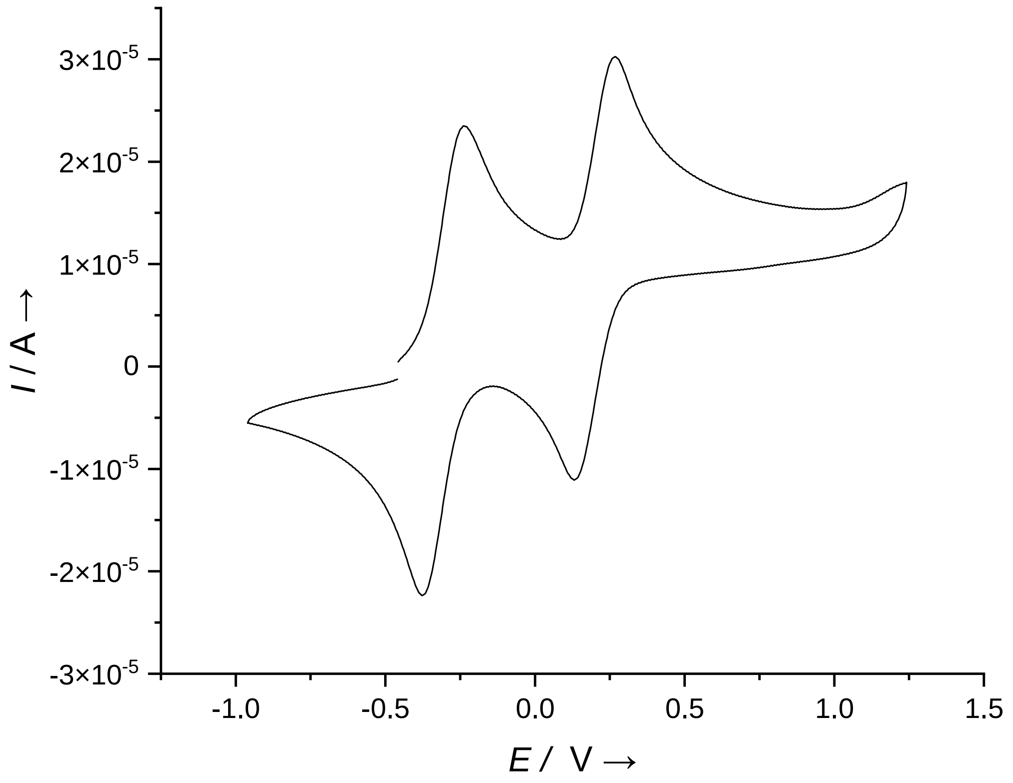

Cyclic voltammetry (CV) curve for compound [Co(hfac)$_2$(L4)] in CH_2Cl_2 (Ag/AgCl reference electrode, 0.1 M N(*n*Bu)$_4$(PF_6) as supporting electrolyte, scan rate 30 mV s^{-1}). Potentials given vs. the Fc^+/Fc redox couple.

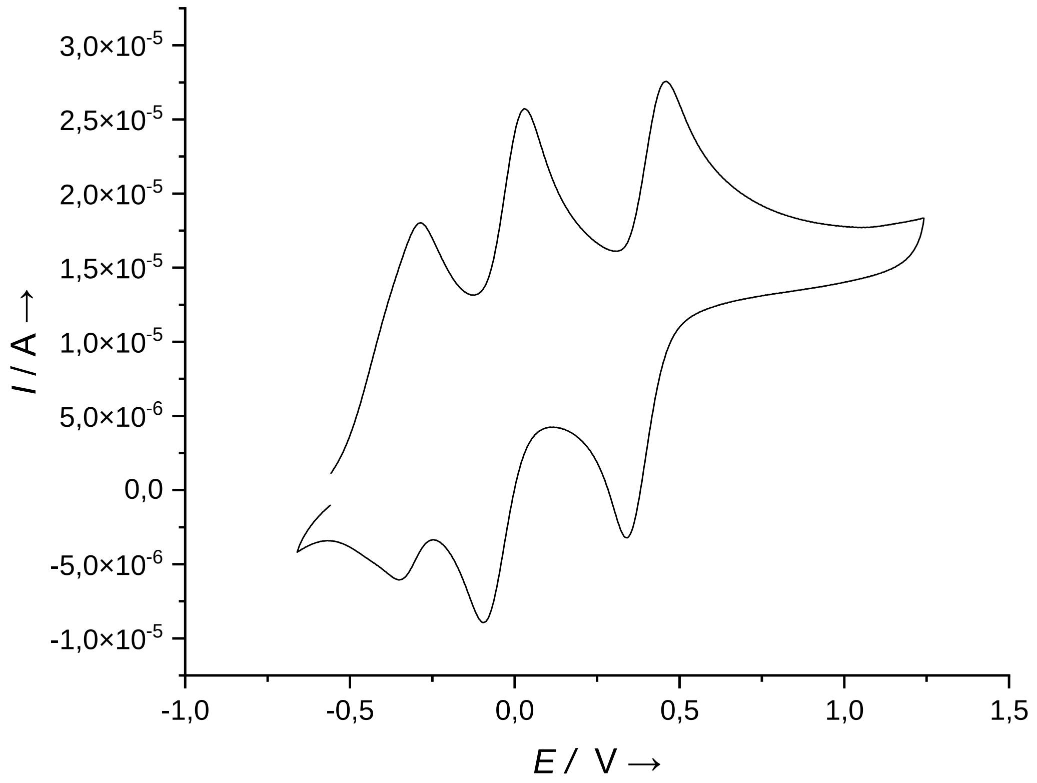

Cyclic voltammetry (CV) curve for compound [Co(acac)$_2$(L5)] in CH_2Cl_2 (Ag/AgCl reference electrode, 0.1 M N(*n*Bu)$_4$(PF_6) as supporting electrolyte, scan rate 30 mV s^{-1}). Potentials given vs. the Fc^+/Fc redox couple.

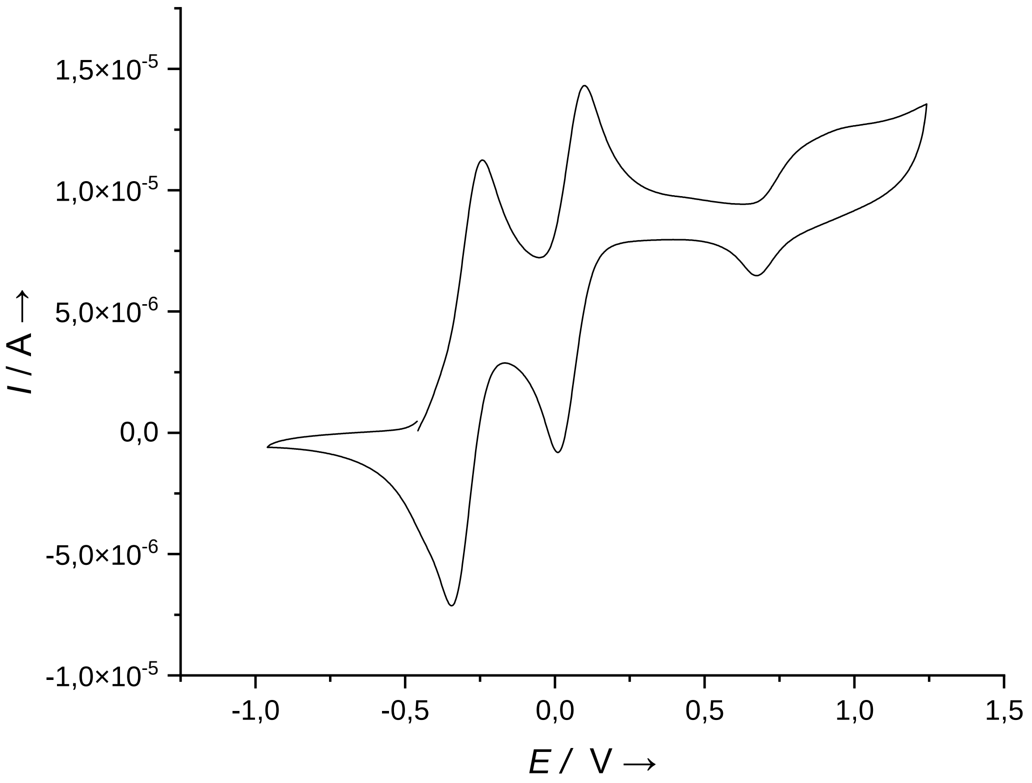

Cyclic voltammetry (CV) curve for compound [Co(tfac)$_2$(L5)] in CH_2Cl_2 (Ag/AgCl reference electrode, 0.1 M N(*n*Bu)$_4$(PF_6) as supporting electrolyte, scan rate 30 mV s^{-1}). Potentials given vs. the Fc^+/Fc redox couple.

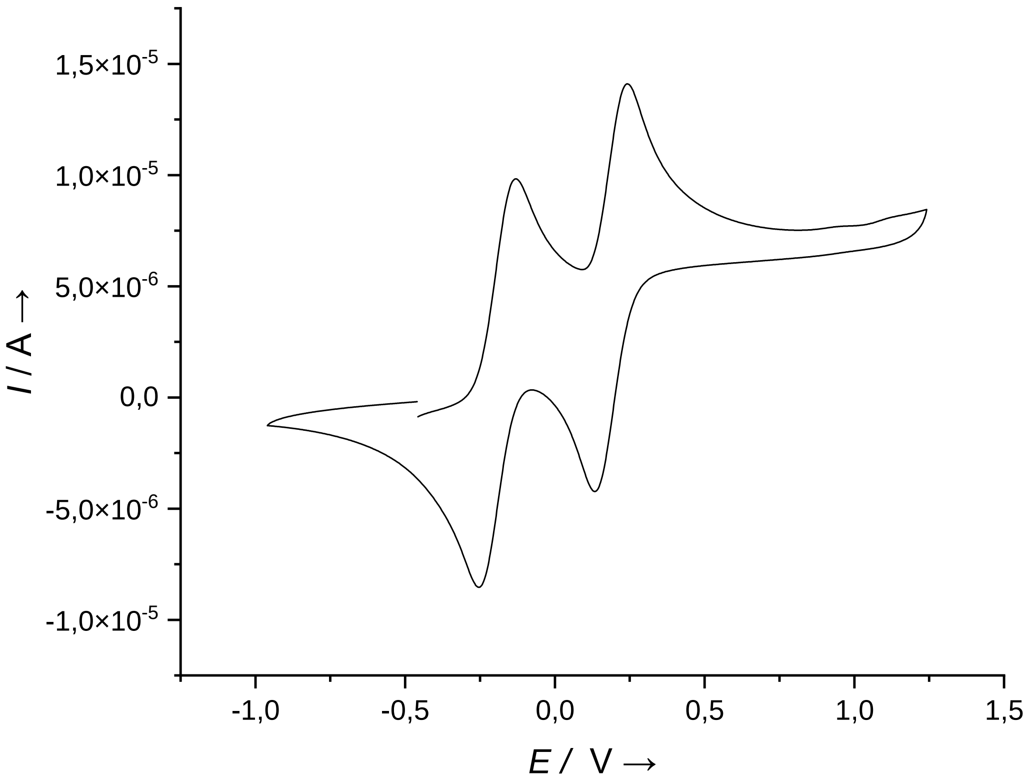

Cyclic voltammetry (CV) curve for compound [Co(hfac)$_2$(L5)] in CH_2Cl_2 (Ag/AgCl reference electrode, 0.1 M N(*n*Bu)$_4$(PF$_6$) as supporting electrolyte, scan rate 30 mV s^{-1}). Potentials given vs. the Fc^+/Fc redox couple.

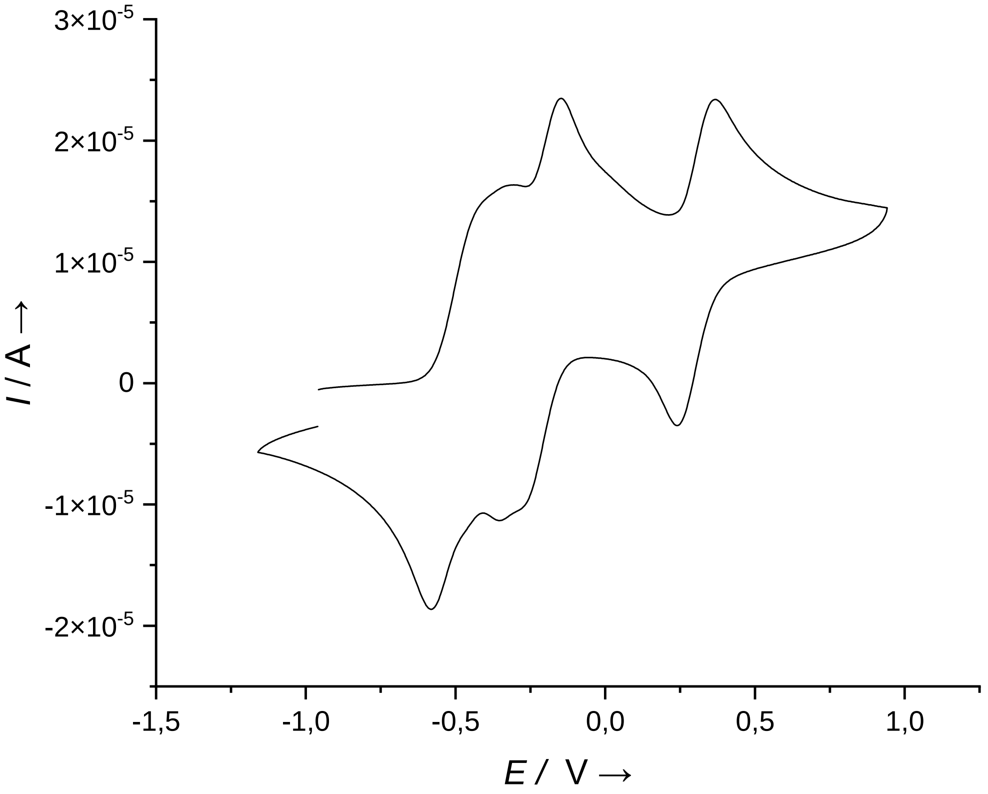

Cyclic voltammetry (CV) curve for compound [Co(acac)$_2$(L6)] in CH_2Cl_2 (Ag/AgCl reference electrode, 0.1 M N(*n*Bu)$_4$(PF_6) as supporting electrolyte, scan rate 30 mV s^{-1}). Potentials given vs. the Fc^+/Fc redox couple.

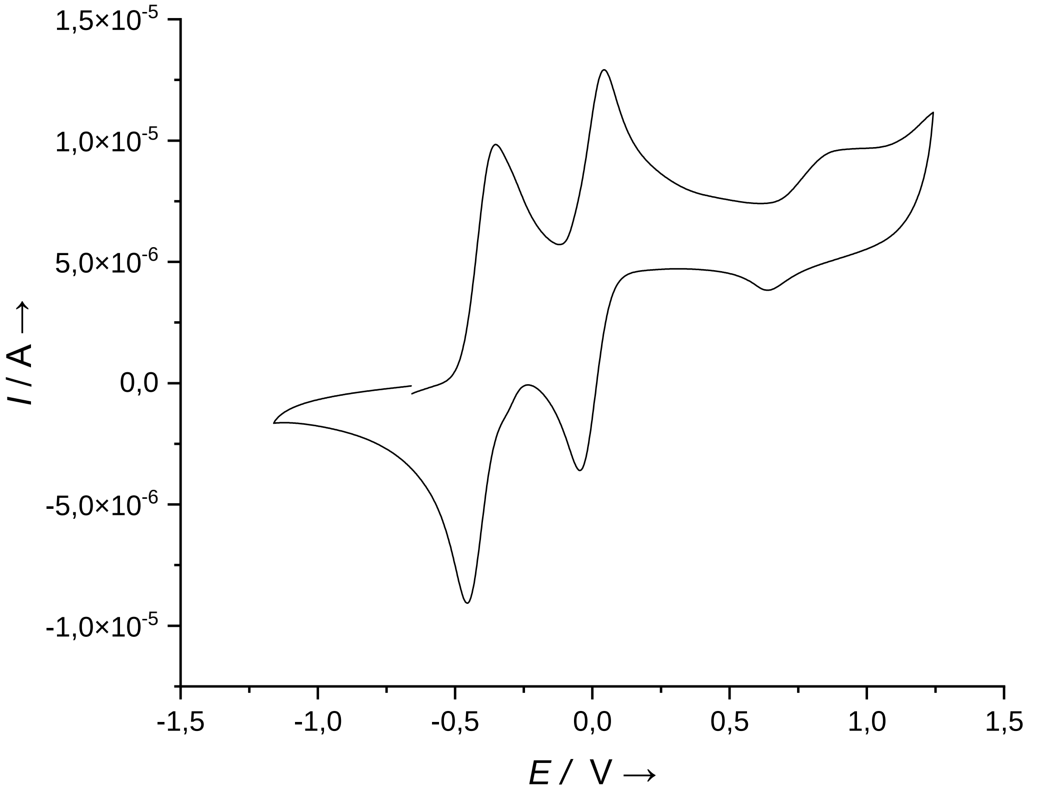

Cyclic voltammetry (CV) curve for compound [Co(tfac)$_2$(L6)] in CH_2Cl_2 (Ag/AgCl reference electrode, 0.1 M N(*n*Bu)$_4$(PF_6) as supporting electrolyte, scan rate 30 mV s^{-1}). Potentials given vs. the Fc^+/Fc redox couple.

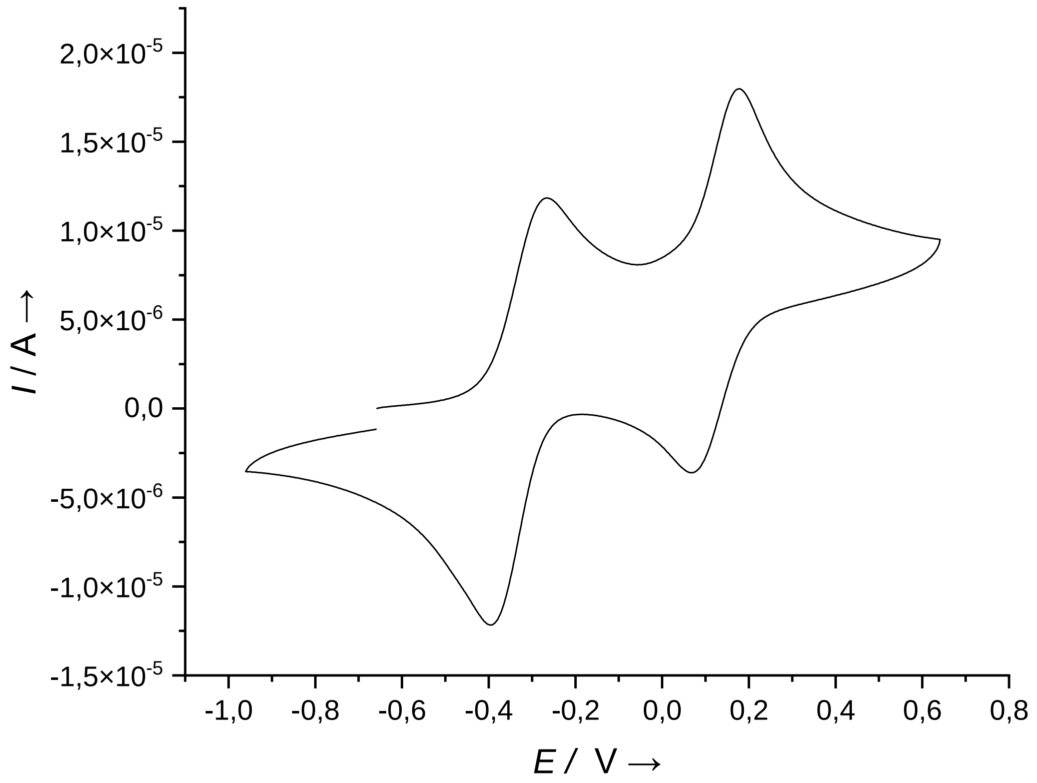

Cyclic voltammetry (CV) curve for compound [Co(hfac)$_2$(L6)] in CH_2Cl_2 (Ag/AgCl reference electrode, 0.1 M N(*n*Bu)$_4$(PF_6) as supporting electrolyte, scan rate 30 mV s^{-1}). Potentials given vs. the Fc^+/Fc redox couple.

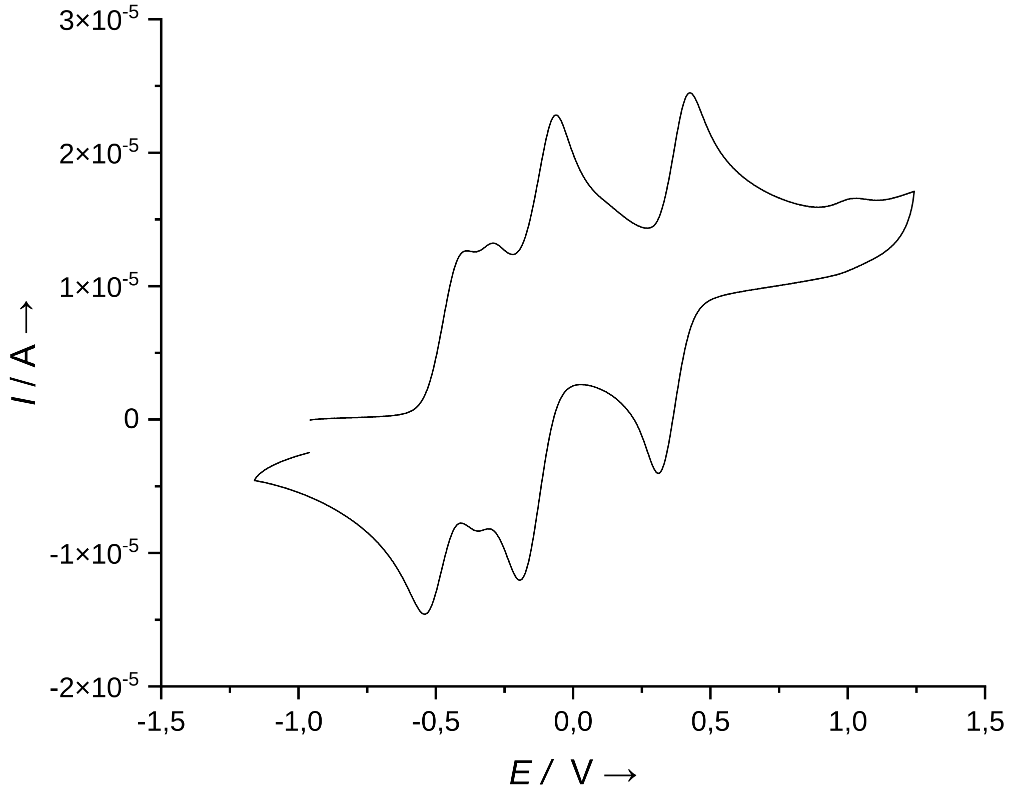

Cyclic voltammetry (CV) curve for compound [Co(acac)$_2$(L7)] in CH_2Cl_2 (Ag/AgCl reference electrode, 0.1 M N(*n*Bu)$_4$(PF_6) as supporting electrolyte, scan rate 100 mV s^{-1}). Potentials given vs. the Fc^+/Fc redox couple.

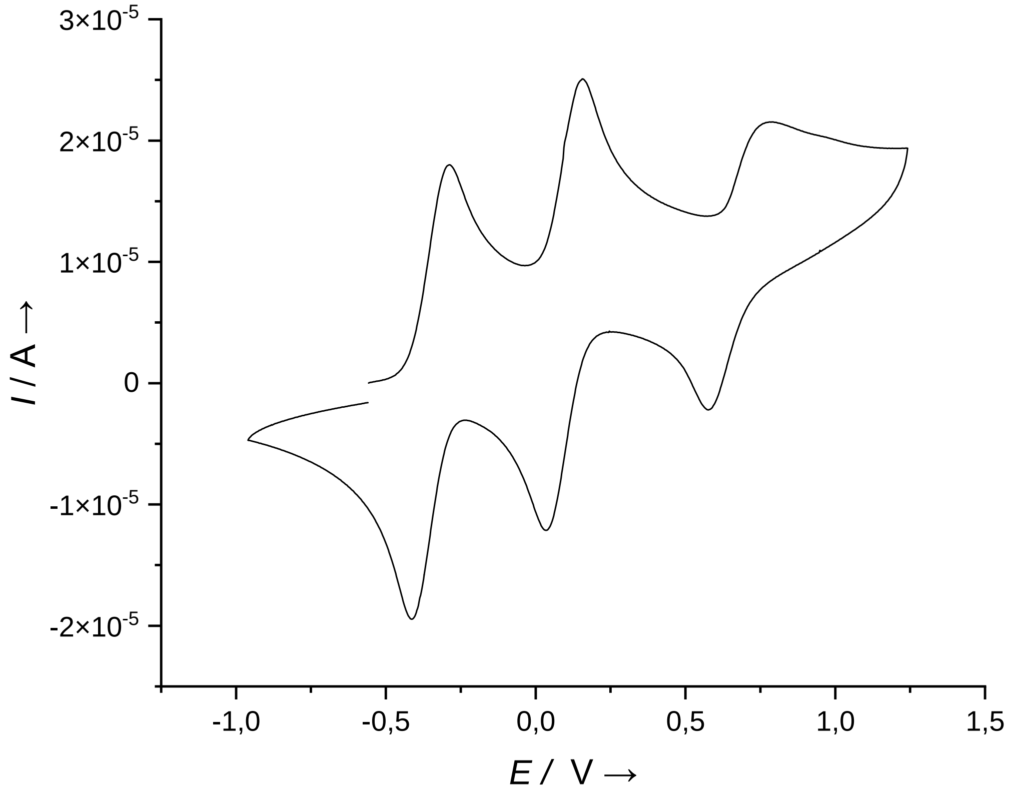

Cyclic voltammetry (CV) curve for compound [Co(tfac)$_2$(L7)] in CH_2Cl_2 (Ag/AgCl reference electrode, 0.1 M N(nBu)$_4$(PF_6) as supporting electrolyte, scan rate 100 mV s^{-1}). Potentials given vs. the Fc^+/Fc redox couple.

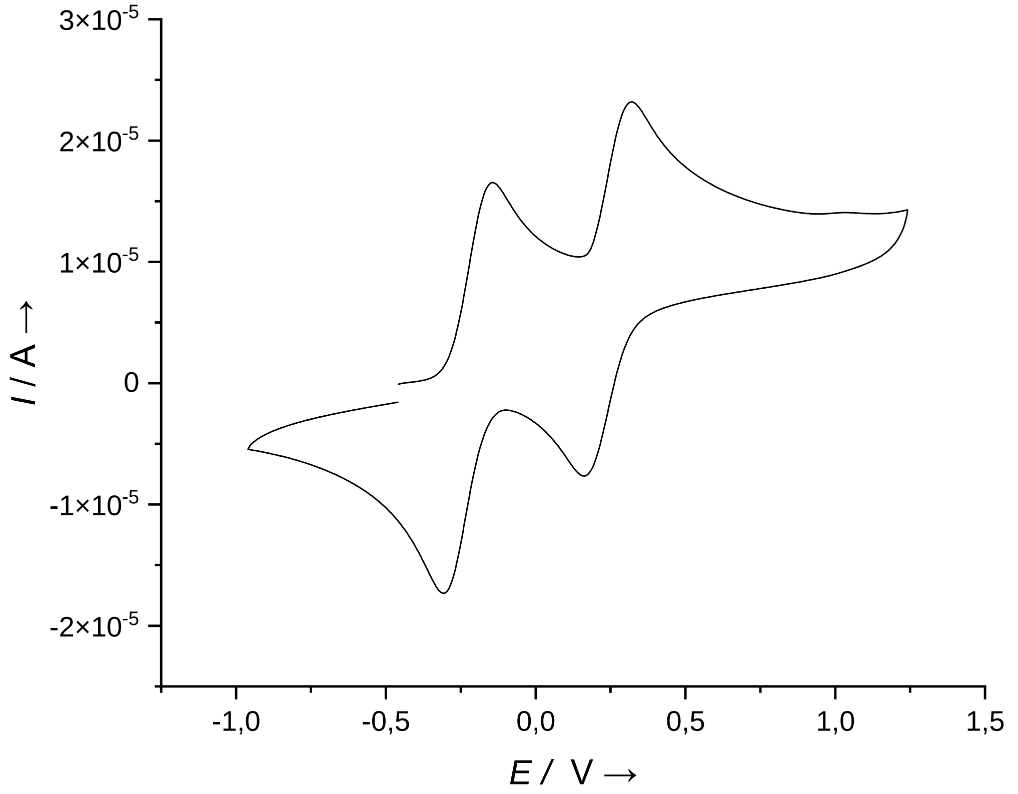

Cyclic voltammetry (CV) curve for compound [Co(hfac)$_2$(L7)] in CH_2Cl_2 (Ag/AgCl reference electrode, 0.1 M N(nBu)$_4$(PF_6) as supporting electrolyte, scan rate 100 mV s^{-1}). Potentials given vs. the Fc^+/Fc redox couple.

Redox potentials ($E_{1/2}$ and E_{ox} values) from CV measurements on solutions of the neutral complexes in CH_2Cl_2 solutions.

**Compound**	$E_{1/2}$ (E_{ox})	$E_{1/2}$ (E_{ox})	$E_{1/2}$ (E_{ox})
[Co(acac)$_2$L1]	- (-0.47)	0.19 (0.24)	0.65 (0.70)
[Co(tfac)$_2$L1]	0.03 (0.09)	0.52 (0.58)	1.05 (1.12)
[Co(hfac)$_2$L1)]	-0.04 (0.03)	0.45 (0.51)	-
[Co(acac)$_2$L2]	-0.50 (-0.44)	0.01 (0.07)	0.63 (0.69)
[Co(tfac)$_2$L2]	-0.24 (-0.18)	0.28 (0.34)	0.83 (0.88)
[Co(hfac)$_2$L2)]	-0.13 (-0.08)	0.45 (0.49)	-
[Co(acac)$_2$L3]	- (-0.38)	-0.20 (-0.15)	0.04 (0.11)
[Co(tfac)$_2$L3]	-0.22 (-0.16)	0.31 (0.36)	0.81 (0.86)
[Co(hfac)$_2$L3)]	-0.10 (-0.05)	0.48 (0.53)	-
[Co(acac)$_2$L4]	-0.36 (-0.29)	0.09 (0.15)	0.68 (0.77)
[Co(tfac)$_2$L4]	-0.48 (-0.38)	-0.17 (-0.09)	0.40 (0.48)
[Co(hfac)$_2$L4)]	-0.31 (-0.24)	0.20 (0.27)	-
[Co(acac)$_2$L5]	-0.32 (-0.28)	-0.04 (0.03)	0.40 (0.46)
[Co(tfac)$_2$L5]	-0.30 (-0.24)	0.06 (0.10)	-
[Co(hfac)$_2$L5)]	-0.19 (-0.13)	0.19 (0.24)	-
[Co(acac)$_2$L6]	-0.46 (-0.33)	-0.25 (-0.15)	0.31 (0.37)
[Co(tfac)$_2$L6]	-0.40 (-0.35)	0.00 (0.04)	-
[Co(hfac)$_2$L6)]	-0.33 (-0.27)	0.13 (0.18)	-
[Co(acac)$_2$L7]	-0.47 (-0.39)	-0.13 (-0.06)	0.37 (0.42)
[Co(tfac)$_2$L7]	-0.35 (-0.29)	0.10 (0.16)	-
[Co(hfac)$_2$L7)]	-0.23 (-0.14)	0.24 (0.32)	-

2.3.3 Crystallographic data of the neutral complexes

Selected bond lengths in Å for the neutral complexes [Co(tfac)$_2$(L1)], [Co(hfac)$_2$(L1)], [Co(acac)$_2$(L2)], [Co(tfac)$_2$(L2)] and [Co(hfac)$_2$(L2)] in the solid-state from SCXRD experiments at 100 K.

Bond	[Co(hfac)$_2$(L1)]	[Co(acac)$_2$(L2)]	[Co(tfac)$_2$(L2)]
Name	Jo198cohfac	Jo212	Jo213
Co1 O1	2.074(3)	2.0650(17)	2.100(4)
Co1 O2	2.127(3)	2.0837(18)	2.055(4)
Co1 O3	2.123(3)	2.1003(17)	2.089(4)
Co1 O4	2.143(3)	2.0802(17)	2.088(4)
Co1 N1	2.077(4)	2.1462(19)	2.124(5)
Co1 N4	2.064(4)	2.171(2)	2.107(5)
N1 C1	1.419(6)	1.407(3)	1.420(7)
N4 C2	1.408(6)	1.409(3)	1.417(7)
C1 C2	1.406(6)	1.415(3)	1.405(8)
C1 C6	1.402(6)	1.393(3)	1.397(8)
C2 C3	1.395(6)	1.397(3)	1.405(8)
C3 C4	1.389(7)	1.393(3)	1.389(9)
C4 C5	1.399(7)	1.406(3)	1.405(9)
C5 C6	1.392(7)	1.385(3)	1.378(8)

Selected bond lengths in Å for the neutral complexes [Co(acac)$_2$(L3)], [Co(acac)$_2$(L4)], [Co(tfac)$_2$(L4)] and [Co(hfac)$_2$(L4)] in the solid-state from SCXRD experiments at 100 K.

Bond	[Co(acac)$_2$(L3)]	[Co(acac)$_2$(L4)]	[Co(hfac)$_2$(L4)]
Name	Jol3acac	Jol4acac	Jol4hfac
Co1 O1	2.0704(10)	2.0840(15)	2.115(2)
Co1 O2	2.0893(10)	2.0787(14)	2.095(2)
Co1 O3	2.0647(10)	2.0873(14)	2.092(2)
Co1 O4	2.0849(10)	2.0657(14)	2.109(2)
Co1 N1	2.1721(11)	2.1851(16)	2.067(2)
Co1 N4	2.1438(11)	2.1339(16)	2.111(2)
N1 C1	1.4071(17)	1.419(2)	1.417(4)
N4 C2	1.4077(17)	1.401(2)	1.412(4)
C1 C2	1.4086(18)	1.414(2)	1.408(4)
C1 C6	1.4014(18)	1.407(3)	1.409(4)
C2 C3	1.3976(18)	1.411(2)	1.413(4)
C3 C4	1.3930(19)	1.374(3)	1.373(4)
C4 C5	1.401(2)	1.379(2)	1.382(4)
C5 C6	1.3934(19)	1.375(3)	1.374(4)

Selected bond lengths in Å for the neutral complexes [Co(acac)$_2$(L5)], [Co(tfac)$_2$(L5)] and [Co(hfac)$_2$(L5)] in the solid-state from SCXRD experiments at 100 K.

Bond	[Co(acac)$_2$(L5)]	[Co(tfac)$_2$(L5)]	[Co(hfac)$_2$(L5)]
Name	Jomk1	JoCoacac	Jomk3
Co1 O3	2.037(2)	2.1046(18)	2.1166(12)

Co1 O4	2.068(2)	2.1165(18)	2.1138(12)
Co1 O5	2.115(2)	2.0532(18)	2.0742(12)
Co1 O6	2.087(2)	2.0963(18)	2.0994(12)
Co1 N1	2.141(2)	2.106(2)	2.0761(13)
Co1 N4	2.133(3)	2.124(2)	2.1190(14)
N1 C1	1.407(4)	1.420(3)	1.413(2)
N4 C2	1.402(4)	1.413(3)	1.4146(19)
C1 C2	1.405(4)	1.400(3)	1.395(2)
C1 C6	1.402(4)	1.400(3)	1.407(2)
C2 C3	1.403(5)	1.406(3)	1.406(2)
C3 C4	1.381(5)	1.381(3)	1.385(2)
C4 C5	1.408(6)	1.406(3)	1.405(2)
C5 C6	1.372(5)	1.384(3)	1.385(2)

Selected bond lengths in Å for the neutral complexes [Co(acac)$_2$(L6)], [Co(tfac)$_2$(L6)] and [Co(hfac)$_2$(L6)] in the solid-state from SCXRD experiments at 100 K.

Bond	[Co(acac)$_2$(L6)]	[Co(tfac)$_2$(L6)]	[Co(hfac)$_2$(L6)]
Name	Jomk30	Jomk12	Jomk13_1
Co1 O3	2.071(2)	2.064(2)	2.0914(18)
Co1 O4	2.078(2)	2.111(2)	2.0862(18)
Co1 O5	2.073(2)	2.086(2)	2.0919(18)
Co1 O6	2.091(2)	2.081(2)	2.1096(18)
Co1 N1	2.206(2)	2.201(3)	2.081(2)
Co1 N4	2.106(2)	2.103(2)	2.124(2)
N1 C1	1.405(4)	1.409(4)	1.407(3)
N4 C2	1.410(3)	1.409(4)	1.407(3)
C1 C2	1.404(4)	1.392(4)	1.403(4)
C1 C6	1.406(4)	1.408(4)	1.399(4)
C2 C3	1.399(4)	1.408(4)	1.399(4)
C3 C4	1.384(4)	1.386(4)	1.379(4)
C4 C5	1.396(4)	1.406(4)	1.392(4)
C5 C6	1.383(4)	1.390(4)	1.394(4)

Selected bond lengths in Å for the neutral complexes [Co(tfac)$_2$(L7)] and [Co(hfac)$_2$(L7)] in the solid-state from SCXRD experiments at 100 K.

Bond	[Co(tfac)$_2$(L7)]	[Co(hfac)$_2$(L7)]
Name	Jomk9_1 = jomk27	Jomk28
Co-O3	2.084(2)	2.081(2)
Co-O4	2.074(2)	2.120(2)
Co-O5	2.061(2)	2.081(3)
Co-O6	2.073(2)	2.1081(3)
Co1-N1	2.138(3)	2.083(3)
Co1-N4	2.107(3)	2.096(3)

N1-C1	1.410(4)	1.418(4)
N4-C2	1.406(4)	1.420(5)
C1-C2	1.400(5)	1.403(5)
C1-C6	1.407(5)	1.402(5)
C2-C3	1.397(5)	1.396(5)
C3-C4	1.390(5)	1.376(6)
C4-C5	1.394(5)	1.385(6)
C5-C6	1.388(5)	1.389(5)

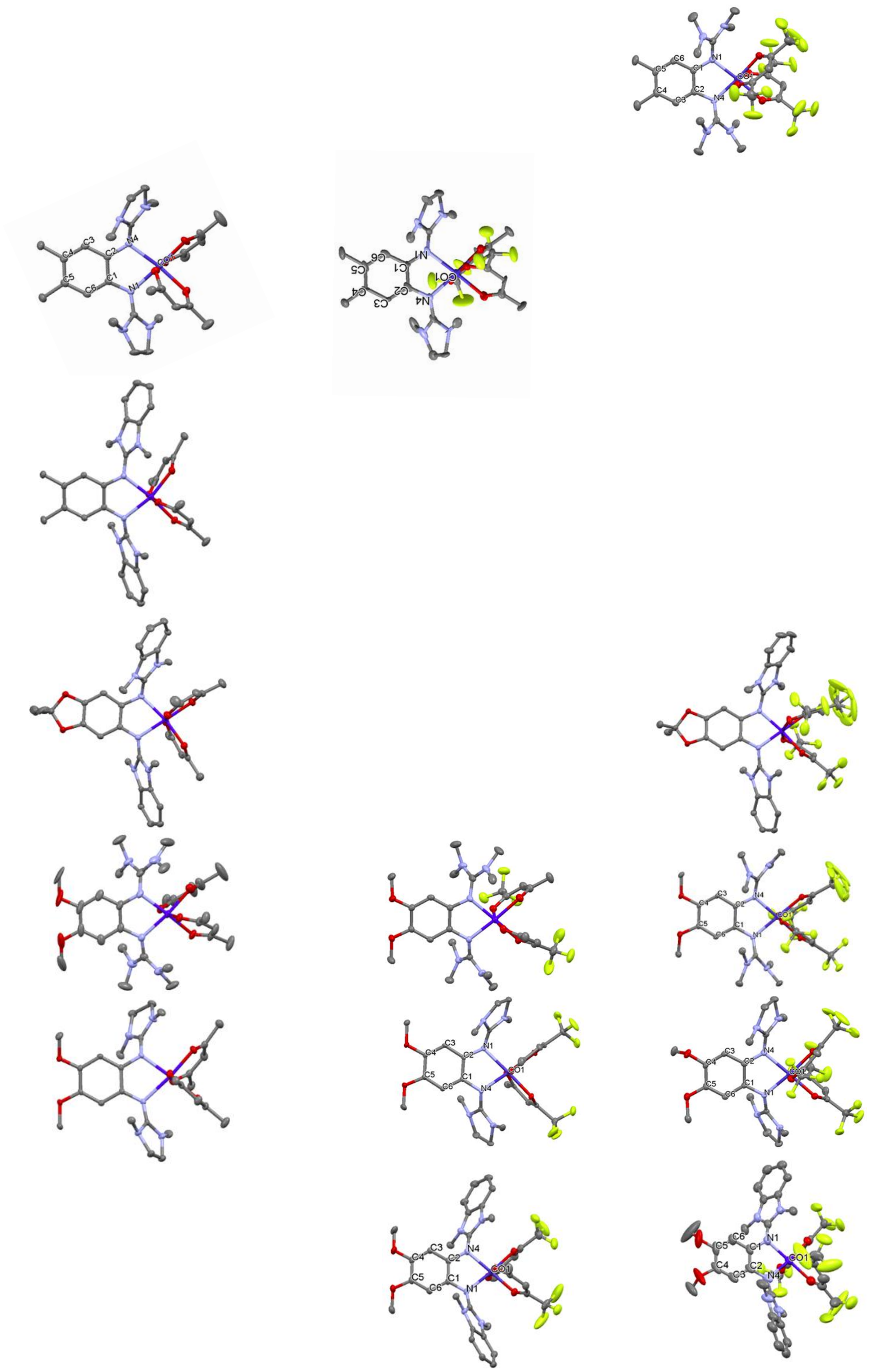

Illustration of the solid-state structures of all neutral cobalt complexes obtained in this work. Color code: Co dark blue, N pale blue, F light- green, C grey, O red. Displacement ellipsoids drawn at the 50% probability level. Hydrogen atoms omitted.

Compound	$[Co(hfac)_2(L1)]$	[Co(acac)2(L2)]	[Co(tfac)2(L2)]
Identification code	mo_2021_jo198Cohfac_0ma	mo_2021_jo212_01	mo_2022_jo213_02
Empirical formula	$C_{28}H_{34}CoF_{12}N_6O_4$	$C_{28}H_{42}CoN_6O_4$	$C_{28}H_{36}CoF_6N_6O_4$
Formula weight	805.54	585.60	693.56
Temperature/K	100.00	100.00	100.00
Crystal system	monoclinic	triclinic	orthorhombic
Space group	$P2_1/c$	P-1	$P2_12_12_1$
a/Å	13.290(2)	11.4512(10)	9.688(2)
b/Å	10.9120(15)	14.2868(13)	17.016(4)
c/Å	24.220(4)	18.7844(16)	19.522(5)
α/°	90	96.372(4)	90
β/°	96.044(5)	94.472(3)	90
γ/°	90	90.250(3)	90
Volume/$Å^3$	3492.9(9)	3044.6(5)	3218.2(13)
Z	4	4	4
ρ_{calc}g/cm^3	1.532	1.278	1.431
μ/mm^{-1}	0.598	0.605	0.610
F(000)	1644.0	1244.0	1436.0
Crystal size/mm^3	0.204 × 0.135 × 0.12	0.244 × 0.163 × 0.134	0.352 × 0.229 × 0.138
Radiation	MoKα (λ = 0.71073)	MoKα (λ = 0.71073)	MoKα (λ = 0.71073)
2Θ range for data collection/°	4.098 to 51.998	3.798 to 52	4.172 to 51.982
Index ranges	-16 ≤ h ≤ 16, -13 ≤ k ≤ 13, -29 ≤ l ≤ 29	-14 ≤ h ≤ 14, -17 ≤ k ≤ 17, -23 ≤ l ≤ 23	-11 ≤ h ≤ 11, -20 ≤ k ≤ 20, -23 ≤ l ≤ 24
Reflections collected	92985	143090	38103
Independent reflections	6865 [R_{int} = 0.1503, R_{sigma} = 0.0720]	11956 [R_{int} = 0.1181, R_{sigma} = 0.0486]	6296 [R_{int} = 0.1646, R_{sigma} = 0.1017]
Data/restraints/parameters	6865/6/479	11956/0/723	6296/0/415
Goodness-of-fit on F^2	1.112	1.032	1.032
Final R indexes [I>=2σ (I)]	R_1 = 0.0682, wR_2 = 0.1644	R_1 = 0.0411, wR_2 = 0.0911	R_1 = 0.0551, wR_2 = 0.1207
Final R indexes [all data]	R_1 = 0.1026, wR_2 = 0.1966	R_1 = 0.0559, wR_2 = 0.0997	R_1 = 0.0712, wR_2 = 0.1332
Largest diff. peak/hole / e Å^{-3}	0.85/-0.88	0.31/-0.43	0.54/-0.44

Compound	[Co(acac)$_2$(L3)]	[Co(acac)$_2$(L4)]	[Co(hfac)$_2$(L4)]	[Co(acac)$_2$(L5)]
Identification code	mo_2023_jol3acac_0ma	mo_2023_jol4acac_0ma	mo_2023_jol4hfac_2_0ma	mo_2022_jomk1_0m
Empirical formula	$C_{36}H_{42}CoN_6O_4$ * CH_2Cl_2	$C_{37}H_{42}CoN_6O_6$ * 2 CH_2Cl_2	$C_{37}H_{30}CoF_{12}N_6O_6$	$C_{28}H_{46}CoN_6O_6$
Formula weight	766.61	895.55	941.60	621.64
Temperature/K	100.00	100.00	100.00	100.00
Crystal system	monoclinic	monoclinic	monoclinic	triclinic
Space group	$P2_1/c$	$P2_1/c$	$P2_1/c$	P-1
a/Å	14.7804(14)	12.7323(17)	13.2304(8)	10.0548(12)
b/Å	14.4890(16)	13.680(2)	11.4339(7)	10.4473(13)
c/Å	17.465(2)	24.262(3)	27.0839(16)	16.208(2)
α/°	90	90	90	107.207(4)
β/°	95.298(4)	95.253(6)	94.632(2)	91.770(5)
γ/°	90	90	90	103.927(4)
Volume/Å^3	3724.2(7)	4208.1(10)	4083.7(4)	1569.0(3)
Z	4	4	4	2
ρ_{calc}g/cm^3	1.367	1.414	1.532	1.316
μ/mm^{-1}	0.652	0.714	0.527	0.596
F(000)	1604.0	1860.0	1908.0	662.0
Crystal size/mm^3	0.391 × 0.324 × 0.269	0.43 × 0.315 × 0.239	0.296 × 0.092 × 0.074	0.251 × 0.121 × 0.05
Radiation	MoKα (λ = 0.71073)	MoKα (λ = 0.71073)	MoKα (λ = 0.71073)	MoKα (λ = 0.71073)
2Θ range for data collection/°	3.944 to 54.992	4.38 to 52.998	3.868 to 52.998	4.2 to 51.996
Index ranges	-19 ≤ h ≤ 19, -18 ≤ k ≤ 18, -22 ≤ l ≤ 22	-15 ≤ h ≤ 15, -17 ≤ k ≤ 17, -30 ≤ l ≤ 30	-16 ≤ h ≤ 16, -14 ≤ k ≤ 14, -33 ≤ l ≤ 33	-12 ≤ h ≤ 12, -12 ≤ k ≤ 12, -19 ≤ l ≤ 19
Reflections collected	173180	232148	406597	50720
Independent reflections	8550 [R_{int} = 0.0768, R_{sigma} = 0.0279]	8710 [R_{int} = 0.1028, R_{sigma} = 0.0346]	8468 [R_{int} = 0.0805, R_{sigma} = 0.0149]	6161 [Rint = 0.1088, Rsigma = 0.0766]
Data/restraints/parameters	8550/0/461	8710/36/571	8468/129/594	6161/6/384
Goodness-of-fit on F^2	1.038	1.034	1.149	1.016
Final R indexes [I>=2σ (I)]	R_1 = 0.0294, wR_2 = 0.0738	R_1 = 0.0376, wR_2 = 0.1006	R_1 = 0.0577, wR_2 = 0.1320	R1 = 0.0511, wR2 = 0.1140
Final R indexes [all data]	R_1 = 0.0348, wR_2 = 0.0789	R_1 = 0.0428, wR_2 = 0.1062	R_1 = 0.0629, wR_2 = 0.1348	R1 = 0.0778, wR2 = 0.1287
Largest diff. peak/hole / e Å^{-3}	0.42/-0.40	0.95/-0.81	1.80/-0.77	0.36/-0.74

Compound	**[Co(tfac)$_2$(L5)]**	**[Co(hfac)$_2$(L5)]**	**[Co(acac)$_2$(L6)]**	**[Co(tfac)$_2$(L6)]**	**[Co(hfac)$_2$(L6)]**
Identification code	mo_2023_joCoacac_0ma	mo_2022_jomk3_0m	mo_2022_jomk30_01a	mo_jomk12_0m	mo_jomk13_1_0m
Empirical formula	$C_{28}H_{40}CoF_6N_6O_6$	$C_{28}H_{34}CoF_{12}N_6O_6$	$C_{28}H_{42}CoN_6O_6$	$C_{28}H_{36}CoF_6N_6O_6$ * CH_2Cl_2	2 [$C_{28}H_{30}CoF_{12}N_6O_6$] * CH_2Cl_2
Formula weight	729.59	837.54	617.60	810.48	1751.94
Temperature/K	100.00	100.00	100.00	100.00	100.00
Crystal system	monoclinic	triclinic	monoclinic	monoclinic	triclinic
Space group	P21/c	P-1	$P2_1/c$	$P2_1/c$	P-1
a/Å	15.948(6)	10.2710(4)	13.6716(19)	8.2739(5)	13.4180(4)
b/Å	13.116(5)	12.3112(4)	15.214(2)	12.2328(8)	16.3041(5)
c/Å	16.355(5)	15.5348(6)	15.3737(18)	34.269(2)	16.6630(5)
α/°	90	86.2170(10)	90	90	83.0710(10)
β/°	102.518(13)	73.1280(10)	112.272(4)	92.477(2)	80.1960(10)
γ/°	90	67.3050(10)	90	90	82.4530(10)
Volume/Å^3	3340(2)	1731.89(11)	2959.2(7)	3465.2(4)	3542.92(19)
Z	4	2	4	4	2
ρ_{calc}g/cm^3	1.451	1.606	1.386	1.554	1.642
μ/mm^{-1}	0.596	0.610	0.631	0.732	0.674
F(000)	1516.0	854.0	1308.0	1668.0	1776.0
Crystal size/mm^3	0.257 × 0.197 × 0.086	0.443 × 0.181 × 0.128	0.376 × 0.22 × 0.177	0.3 × 0.3 × 0.3	0.3 × 0.3 × 0.2
Radiation	MoKα (λ = 0.71073)	MoKα (λ = 0.71073)	MoKα (λ = 0.71073)	MoKα (λ = 0.71073)	MoKα (λ = 0.71073)
2Θ range for data collection/°	4.018 to 53	4.404 to 57.578	3.92 to 51.996	4.092 to 55.164	3.774 to 51.998
Index ranges	-20 ≤ h ≤ 20, -16 ≤ k ≤ 16, -20 ≤ l ≤ 20	-13 ≤ h ≤ 13, -16 ≤ k ≤ 16, -21 ≤ l ≤ 21	-16 ≤ h ≤ 16, -18 ≤ k ≤ 18, -18 ≤ l ≤ 18	-10 ≤ h ≤ 10, -15 ≤ k ≤ 15, -44 ≤ l ≤ 44	-16 ≤ h ≤ 16, -20 ≤ k ≤ 20, -20 ≤ l ≤ 20
Reflections collected	144178	116181	87445	151625	197300
Independent reflections	6929 [Rint = 0.0984, Rsigma = 0.0305]	9007 [R_{int} = 0.0714, R_{sigma} = 0.0288]	5797 [R_{int} = 0.1381, R_{sigma} = 0.0484]	8022 [R_{int} = 0.0868, R_{sigma} = 0.0282]	13921 [R_{int} = 0.1084, R_{sigma} = 0.0382]
Data/restraints/parameters	6929/0/436	9007/90/542	5797/0/380	8022/7/488	13921/52/1012
Goodness-of-fit on F^2	1.023	1.051	1.116	1.174	1.084
Final R indexes [I>=2σ (I)]	R1 = 0.0437, wR2 = 0.1109	R_1 = 0.0382, wR_2 = 0.0920	R_1 = 0.0508, wR_2 = 0.1219	R_1 = 0.0627, wR_2 = 0.1143	R_1 = 0.0409, wR_2 = 0.0889

Final R indexes [all data]	R1 = 0.0535, wR2 = 0.1187	R_1 = 0.0427, wR_2 = 0.0952	R_1 = 0.0642, wR_2 = 0.1363	R_1 = 0.0695, wR_2 = 0.1173	R_1 = 0.0549, wR_2 = 0.1047
Largest diff. peak/hole / e $Å^{-3}$	0.96/-0.59	0.99/-0.65	0.77/-0.52	0.49/-0.69	1.51/-0.67

Compound	$[Co(tfac)_2(L7)]$	$[Co(hfac)_2(L7)]$
Identification code	Jomk9_1_01a	mo_2022_jomk28_0ma
Empirical formula	$C_{36}H_{36}CoF_6N_6O_6 * CH_2Cl_2$	$C_{36}H_{30}CoF_{12}N_6O_6 * 0.7 CH_2Cl_2$
Formula weight	906.56	929.59
Temperature/K	100.00	100.00
Crystal system	monoclinic	monoclinic
Space group	$P2_1/n$	$P2_1/n$
a/Å	16.6332(17)	11.538(3)
b/Å	15.8427(17)	22.843(6)
c/Å	16.8456(18)	15.708(4)
α/°	90	90
β/°	113.967(4)	96.421(11)
γ/°	90	90
Volume/Å^3	4056.3(7)	4114.0(18)
Z	4	4
ρ_{calc}g/cm^3	1.484	1.501
μ/mm^{-1}	0.635	0.522
F(000)	1860.0	1884.0
Crystal size/mm^3	0.356 × 0.271 × 0.236	0.571 × 0.322 × 0.236
Radiation	MoKα (λ = 0.71073)	MoKα (λ = 0.71073)
2Θ range for data collection/°	3.876 to 51.998	3.974 to 52
Index ranges	-20 ≤ h ≤ 20, -19 ≤ k ≤ 19, -20 ≤ l ≤ 20	-14 ≤ h ≤ 14, -28 ≤ k ≤ 28, -19 ≤ l ≤ 19
Reflections collected	145114	84679
Independent reflections	7979 [R_{int} = 0.0947, R_{sigma} = 0.0340]	8087 [R_{int} = 0.1412, R_{sigma} = 0.0619]
Data/restraints/parameters	7979/0/531	8087/0/656
Goodness-of-fit on F^2	1.093	1.038
Final R indexes [I>=2σ (I)]	R_1 = 0.0610, wR_2 = 0.1641	R_1 = 0.0528, wR_2 = 0.1106
Final R indexes [all data]	R_1 = 0.0691, wR_2 = 0.1722	R_1 = 0.0819, wR_2 = 0.1302
Largest diff. peak/hole / e Å^{-3}	1.44/-0.71	0.53/-0.46

2.4 Synthesis details of oxidized, monocationic complexes

2.4.1 Monocationic cobalt complexes of ligand L1

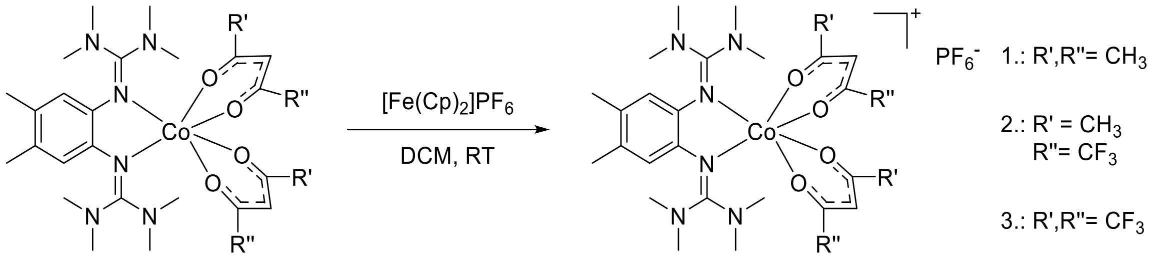

[Co(acac)$_2$(L1)]PF$_6$

The neutral complex [Co(acac)$_2$(L1)] (40 mg, 0.33 mmol) was dissolved in 3 mL dichloromethane. Then, ferrocenium hexafluorophosphate (14 mg, 0.33 mmol) was added; the colour of the solution turned to brown-green. The reaction mixture was stirred for 2 h at room temperature. Subsequently, the solvent was removed in vacuo and the crude brown product washed with *n*-hexane (3 x 3 mL) to yield 41 mg (96%, 0.41 mmol) of [Co(acac)$_2$(L1)]PF$_6$ as a green solid. Crystals suitable for X-ray analysis were obtained through overlayering a saturated CH_2Cl_2 solution with pentane.

Elemental analysis ($C_{28}H_{48}CoN_6O_4PF_6$ + DCM):

calcd.:	C 42.40%	H 6.13%	N 10.23%
found:	C 43.21%	H 6.00%	N 9.88%

UV-vis (CH_3CN): λ_{max} (ε / $M^{-1}cm^{-1}$): 247 ($2.31\cdot10^4$), 281 ($1.50\cdot10^4$), 670 (163) nm.

ESI-HRMS (pos., CH_2Cl_2): *m/z* (M-PF$_6$-acac) = calcd. 490.2545, found 490.2461.

[Co(tfac)$_2$(L1)]PF$_6$

The neutral complex [Co(tfac)$_2$(L1)] (23 mg, 0.33 mmol) was dissolved in 3 mL dichloromethane. Then, ferrocenium-hexafluorophosphate (14 mg, 0.33 mmol) was added; the colour of the solution turned to green. The reaction mixture was stirred for 2 h at room temperature. Subsequently, the solvent was removed in vacuo and the crude green product washed with *n*-hexane (3 x 3 mL) to yield 27 mg (97%, 0.32 mmol) of [Co(tfac)$_2$(L1)]PF$_6$ as a green solid.

Elemental analysis ($C_{28}H_{40}CoN_6O_4PF_6$ + DCM):

calcd.:	C 37.56%	H 4.56%	N 9.06%
found:	C 37.21%	H 5.00%	N 9.88%

UV-vis (CH_3CN): λ_{max} (ε / $M^{-1}cm^{-1}$): 201 ($4.29\cdot10^4$), 247 ($1.61\cdot10^4$), 437 ($0.18\cdot10^4$), 560 ($0.15\cdot10^4$), 835 (288) nm.

ESI-HRMS (pos., CH_2Cl_2): *m/z* (M-PF_6-tfac) = calcd. 544.2184, found 544.2190.

[Co(hfac)$_2$(L1)]PF$_6$

The neutral complex [Co(hfac)$_2$(L1)] (27 mg, 0.33 mmol) was dissolved in 3 mL dichloromethane. Then, ferrocenium hexafluorophosphate (14 mg, 0.33 mmol) was added; the colour of the solution turned dark. The reaction mixture was stirred for 2 h at room temperature. Subsequently, the precipitate was removed and the crude brown product washed with *n*-hexane (3 x 3 mL) to yield 30 mg (95%, 0.31 mmol) of [Co(hfac)$_2$(L1)]PF$_6$ as a brown solid. Crystals suitable for X-ray analysis were obtained through overlayering a saturated CH_2Cl_2 solution with pentane. As the crystalisation was not possible with PF_6^- as counterion it was crystalised with $[Co(hfac)_3]^-$ as counterion.

Elemental analysis ($C_{28}H_{34}CoN_6O_4PF_{18}$ + DCM):

calcd.:	C 33.64%	H 3.50%	N 8.12%
found:	C 33.21%	H 3.00%	N 8.88%

UV-vis (CH_3CN): λ_{max} (ε / $M^{-1}cm^{-1}$): 230 ($4.13\cdot10^4$), 305 ($2.88\cdot10^4$), 470 ($0.20\cdot10^4$), shoulder at 563 (465) nm.

ESI-HRMS (pos., CH_2Cl_2): *m/z* (M-PF_6-2hfac$^-$+H) = calcd. 333.2761, found 333.2765.

2.4.2 Monocationic cobalt complexes of ligand L2

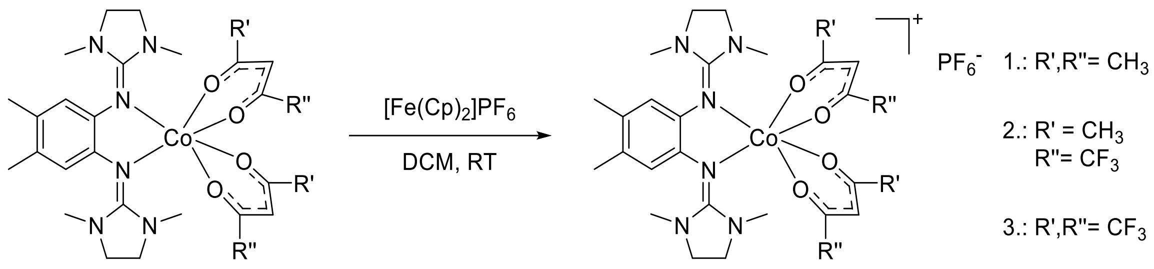

[Co(acac)$_2$(L2)]PF$_6$

The neutral complex [Co(acac)$_2$(L2)] (19 mg, 0.33 mmol) was dissolved in 3 mL dichloromethane. Then ferrocenium-hexafluorophosphate (14 mg, 0.33 mmol) was added; the colour of the solution turned to brown-green. The reaction mixture was stirred for 2 h at room temperature. Subsequently the solvent was removed in vacuo and the crude brown product washed with *n*-hexane (3 x 3 mL) to yield 24 mg (99%, 0.33 mmol) of [Co(acac)$_2$(L2)]PF$_6$ as a green solid.

Elemental analysis ($C_{28}H_{42}CoN_6O_4PF_6$ + DCM):

calcd.:	C 42.71%	H 5.44%	N 10.31%
found:	C 43.21%	H 6.00%	N 9.88%

UV-vis (CH_3CN): λ_{max} (ε / $M^{-1}cm^{-1}$): 207 ($2.38\cdot10^4$), 245 ($1.52\cdot10^4$), 284 ($1.05\cdot10^4$), 567 (167), 650 (163) nm.

ESI-HRMS (pos., CH_2Cl_2): *m/z* (M-PF$_6$) = calcd. 585.2599, found 585.2598.

[Co(tfac)$_2$(L2)]PF$_6$

The neutral complex [Co(tfac)$_2$(L2)] (23 mg, 0.33 mmol) was dissolved in 3 mL dichloromethane. Then ferrocenium-hexafluorophosphate (14 mg, 0.33 mmol) was added; the colour of the solution turned to green. The reaction mixture was stirred for 2 h at room temperature. Subsequently the solvent was removed in vacuo and the crude green product washed with *n*-hexane (3 x 3 mL) to yield 26 mg (94%, 0.31 mmol) of [Co(tfac)$_2$(L2)]PF$_6$ as a green solid.

Elemental analysis ($C_{28}H_{36}CoN_6O_4PF_{12}$):

calcd.:	C 40.11%	H 4.33%	N 10.02%
found:	C 40.21%	H 5.00%	N 9.88%

UV-vis (CH_3CN): λ_{max} (ε / $M^{-1}cm^{-1}$): 205 ($2.82 \cdot 10^4$), 272 ($1.68 \cdot 10^4$), 330 ($1.16 \cdot 10^4$), 437 ($0.22 \cdot 10^4$), 587 (465), 660 (418) nm.

ESI-HRMS (pos., CH_2Cl_2): *m/z* (M-PF_6) = calcd. 693.2034, found 693.2036.

[Co(hfac)$_2$(L2)]PF_6

The neutral complex [Co(hfac)$_2$(L2)] (26 mg, 0.33 mmol) was dissolved in 3 mL dichloromethane. Then, ferrocenium hexafluorophosphate (14 mg, 0.33 mmol) was added; the colour of the solution turned dark. The reaction mixture was stirred for 2 h at room temperature. Subsequently, the precipitate was removed and the crude brown product washed with *n*-hexane (3 x 3 mL) to yield 30 mg (96%, 0.32 mmol) of [Co(hfac)$_2$(L2)]PF_6 as a brown solid. Crystals suitable for X-ray analysis were obtained through overlayering a saturated CH_2Cl_2 solution with pentane.

Elemental analysis ($C_{28}H_{48}CoN_6O_4PF_6$ + DCM):

calcd.:	C 33.77%	H 3.13%	N 8.15%
found:	C 33.21%	H 4.00%	N 9.00%

UV-vis (CH_3CN): λ_{max} (ε / $M^{-1}cm^{-1}$): 209 ($3.19 \cdot 10^4$), 286 ($2.22 \cdot 10^4$), 448 ($0.14 \cdot 10^4$), shoulder at 550 (338), ca. 720 (62) nm.

ESI-HRMS (pos., CH_2Cl_2): *m/z* (M-PF_6) = calcd. 801.1469, found 801.1461.

2.4.3 Monocationic cobalt complexes of ligand L3

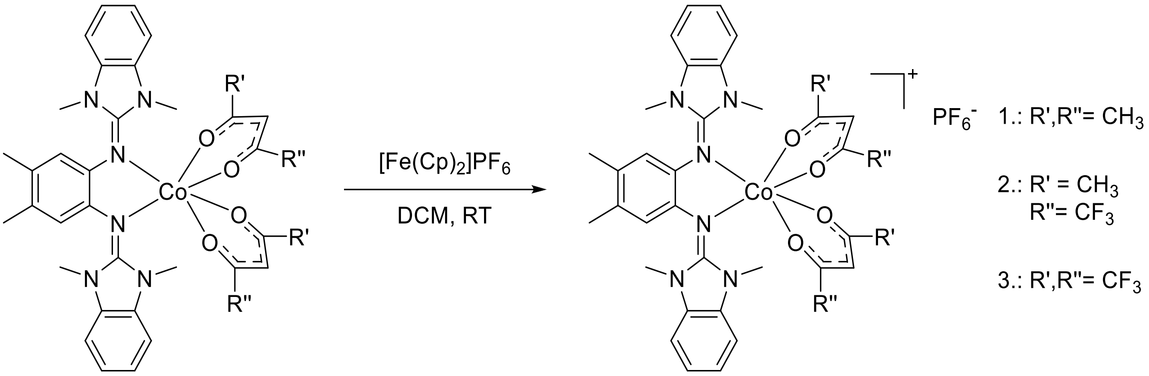

[Co(acac)$_2$(L3)]PF$_6$

The neutral complex [Co(acac)$_2$(L3)] (22 mg, 0.33 mmol) was dissolved in 3 mL dichloromethane. Then, ferrocenium hexafluorophosphate (14 mg, 0.33 mmol) was added; the colour of the solution turned to brown-green. The reaction mixture was stirred for 2 h at room temperature. Subsequently, the solvent was removed in vacuo and the crude brown product washed with *n*-hexane (3 x 3 mL) to yield 26 mg (95%, 0.31 mmol) of [Co(acac)$_2$(L3)]PF$_6$ as a green solid.

Elemental analysis ($C_{36}H_{42}CoN_6O_4PF_6$ + DCM):

calcd.:	C 48.75%	H 4.87%	N 9.22%
found:	C 49.21%	H 5.00%	N 9.88%

UV-vis (CH_3CN): λ_{max} (ε / $M^{-1}cm^{-1}$): 208 ($2.37\cdot10^4$), 245 ($1.53\cdot10^4$), 279 ($1.07\cdot10^4$), 567 (167), 646 (163) nm.

ESI-HRMS (pos., CH_2Cl_2): *m/z* (M-PF$_6$) = calcd. 681.2599, found 681.2598.

[Co(tfac)$_2$(L3)]PF$_6$

The neutral complex [Co(tfac)$_2$(L3)] (26 mg, 0.33 mmol) was dissolved in 3 mL dichloromethane. Then, ferrocenium hexafluorophosphate (14 mg, 0.33 mmol) was added; the colour of the solution turned to green. The reaction mixture was stirred for 2 h at room temperature. Subsequently, the solvent was removed in vacuo and the crude green product washed with *n*-hexane (3 x 3 mL) to yield 30 mg (96%, 0.32 mmol) of [Co(tfac)$_2$(L3)]PF$_6$ as a green solid.

Elemental analysis ($C_{36}H_{36}CoN_6O_4PF_{12}$ + DCM):

calcd.:	C 43.59%	H 3.76%	N 8.24%
found:	C 43.21%	H 3.57%	N 8.29%

UV-vis (CH_3CN): λ_{max} (ε / $M^{-1}cm^{-1}$): 206 ($2.82\cdot10^4$), 272 ($1.68\cdot10^4$), 329 ($1.17\cdot10^4$), 437 ($0.22\cdot10^4$), 593 (465), ca. 655 (419) nm.

ESI-HRMS (pos., CH_2Cl_2): *m/z* (M-PF_6) = calcd. 789.2034, found 789.2031.

[Co(hfac)$_2$(L3)]PF$_6$

The neutral complex [Co(hfac)$_2$(L3)] (30 mg, 0.33 mmol) was dissolved in 3 mL dichloromethane. Then, ferrocenium hexafluorophosphate (mg, 0.33 mmol) was added; the colour of the solution turned dark. The reaction mixture was stirred for 2 h at room temperature. Subsequently, the precipitate was removed and the crude brown product washed with *n*-hexane (3 x 3 mL) to yield 34 mg (99%, 0.33 mmol) of [Co(hfac)$_2$(L3)]PF$_6$ as a brown solid. Crystals suitable for X-ray analysis were obtained through overlayering a saturated CH_2Cl_2 solution with pentane.

Elemental analysis ($C_{36}H_{38}CoN_6O_4PF_{18}$ + DCM):

calcd.:	C 39.42%	H 2.86%	N 7.45%
found:	C 39.21%	H 3.00%	N 7.88%

UV-vis (CH_3CN): λ_{max} (ε / $M^{-1}cm^{-1}$): 212 ($9.98\cdot10^4$), 240 ($8.68\cdot10^4$), 285 ($9.97\cdot10^4$), 535 ($0.85\cdot10^4$), ca. 600 ($0.67\cdot10^4$), 852 (918) nm.

ESI-HRMS (pos., CH_2Cl_2): *m/z* (M-PF_6) = calcd. 897.1469, found 897.1467.

2.4.4 Monocationic cobalt complexes of ligand L4

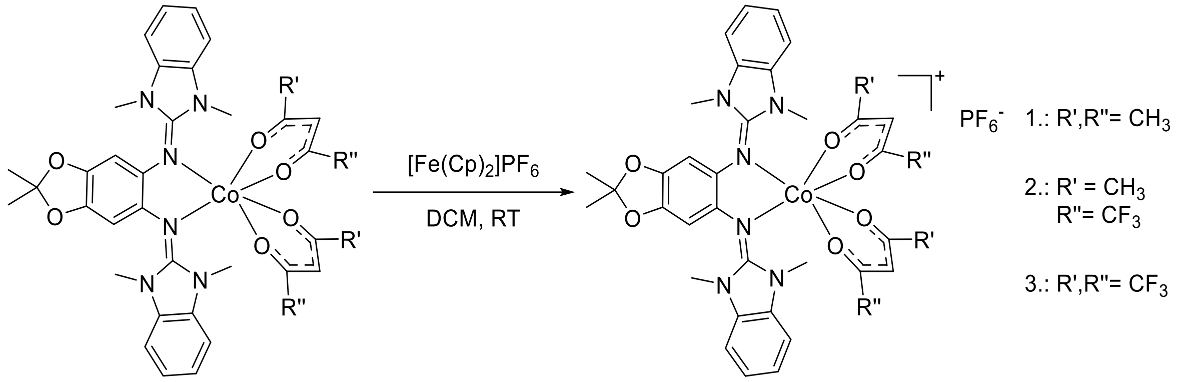

[Co(acac)$_2$(L4)]PF$_6$

The neutral complex [Co(acac)$_2$(L4)] (24 mg, 0.33 mmol) was dissolved in 3 mL dichloromethane. Then, ferrocenium hexafluorophosphate (14 mg, 0.33 mmol) was added; the colour of the solution turned to brown-green. The reaction mixture was stirred for 2 h at room temperature. Subsequently, the solvent was removed in vacuo and the crude brown product washed with *n*-hexane (3 x 3 mL) to yield 28 mg (97%, 0.32 mmol) of [Co(acac)$_2$(L4)]PF$_6$ as a green solid.

Elemental analysis ($C_{27}H_{42}CoN_6O_4PF_6$):

calcd.:	C 51.04%	H 4.86%	N 9.65%
found:	C 51.24%	H 5.16%	N 9.68%

UV-vis (CH_3CN): λ_{max} (ε / $M^{-1}cm^{-1}$): 201 ($6.43\cdot10^4$), 248 ($3.50\cdot10^4$), 280 ($2.87\cdot10^4$), 331 ($1.15\cdot10^4$), 590 ($0.14\cdot10^4$) nm.

ESI-HRMS (pos., CH_2Cl_2): *m/z* (M-acac) = calcd. 725.2498, found 725.2497.

[Co(tfac)$_2$(L4)]PF$_6$

The neutral complex [Co(tfac)$_2$(L4)] (28 mg, 0.33 mmol) was dissolved in 3 mL dichloromethane. Then, ferrocenium hexafluorophosphate (14 mg, 0.33 mmol) was added; the colour of the solution turned to green. The reaction mixture was stirred for 2 h at room temperature. Subsequently, the solvent was removed in vacuo and the crude green product washed with *n*-hexane (3 x 3 mL) to yield 32 mg (99%, 0.33 mmol) of [Co(tfac)$_2$(L4)]PF$_6$ as a green solid.

Elemental analysis ($C_{37}H_{36}CoN_6O_4PF_{12}$ + DCM):

calcd.:	C 42.91%	H 3.60%	N 7.90%
found:	C 42.81%	H 3.60%	N 7.88%

UV-vis (CH_3CN): λ_{max} (ε / $M^{-1}cm^{-1}$): 200 ($1.86 \cdot 10^4$), 277 ($1.35 \cdot 10^4$), 429 ($0.23 \cdot 10^4$), 627 (257) nm.

ESI-HRMS (pos., CH_2Cl_2): *m/z* (M-PF_6) = calcd. 833.1932, found 833.1931.

[Co(hfac)$_2$(L4)]PF$_6$

The neutral complex [Co(hfac)$_2$(L4)] (31 mg, 0.33 mmol) was dissolved in 3 mL dichloromethane. Then, ferrocenium-hexafluorophosphate (14 mg, 0.33 mmol) was added; the colour of the solution turned dark. The reaction mixture was stirred for 2 h at room temperature. Subsequently, the precipitate was removed and the crude brown product washed with *n*-hexane (3 x 3 mL) to yield 35 mg (97%, 0.32 mmol) of [Co(hfac)$_2$(L4)]PF$_6$ as a brown solid.

Elemental analysis ($C_{37}H_{30}CoN_6O_4PF_{18}$ + DCM):

calcd.:	C 38.96%	H 2.75%	N 7.17%
found:	C 39.11%	H 3.02%	N 6.88%

UV-vis (CH_3CN): λ_{max} (ε / $M^{-1}cm^{-1}$): 216 ($1.90 \cdot 10^4$), 297 ($1.35 \cdot 10^4$), 448 ($0.23 \cdot 10^4$), ca. 650 (254) nm.

ESI-HRMS (pos., CH_2Cl_2): *m/z* (M-PF_6) = calcd. 941.1367, found 941.1361.

2.4.5 Monocationic cobalt complexes of ligand L5

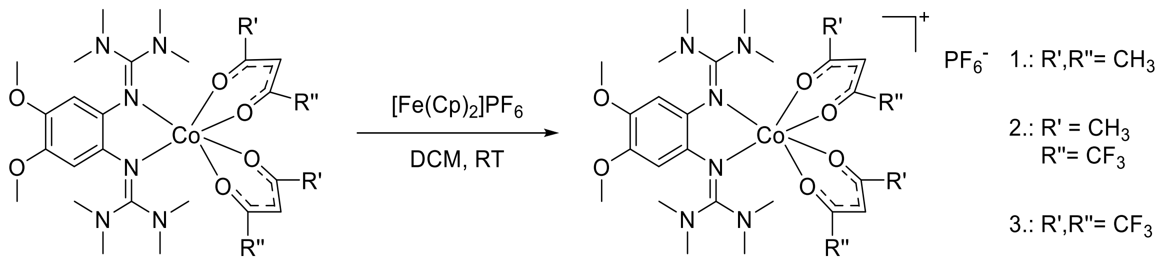

[Co(acac)$_2$(L5)]PF$_6$

The neutral complex [Co(acac)$_2$(L5)] (17 mg, 0.03 mmol) was dissolved in 3 mL dichloromethane. Then, ferrocenium hexafluorophosphate (9.93 mg, 0.03 mmol) was added; the colour of the solution turned to brown-green. The reaction mixture was stirred for 2 h at room temperature. Subsequently the solvent was removed in vacuo and the crude brown product washed with *n*-hexane (3 x 3 mL) to yield 22 mg (96%, 0.029 mmol) of [Co(acac)$_2$(L5)]PF$_6$ as a green solid. Crystals suitable for X-ray analysis were obtained through overlayering a saturated CH_2Cl_2 solution with pentane.

Elemental analysis ($C_{28}H_{48}CoN_6O_6PF_6$ + 0.5 DCM):

calcd.:	C 42.31%	H 5.86%	N 10.39%
found:	C 41.41%	H 6.12%	N 10.97%

UV-vis (CH_3CN): λ_{max} (ε / $M^{-1}cm^{-1}$): 227 ($2.97\cdot10^4$), 264 ($1.71\cdot10^4$), 293 ($1.43\cdot10^4$), 676 (158) nm.

ESI-HRMS (pos., CH_2Cl_2): *m/z* (M-PF$_6$) = calcd. 621.2805, found 621.2807.

[Co(tfac)$_2$(L5)]PF$_6$

The neutral complex [Co(tfac)$_2$(L5)] (18.23 mg, 0.025 mmol) was dissolved in 3 mL dichloromethane. Then, ferrocenium hexafluorophosphate (8.28 mg, 0.025 mmol) was added; the colour of the solution turned to green. The reaction mixture was stirred for 2 h at room temperature. Subsequently, the solvent was removed in vacuo and the crude green product washed with *n*-hexane (3 x 3 mL) to yield 20 mg (91%, 0.023 mmol) of [Co(tfac)$_2$(L5)]PF$_6$ as a green solid. Crystals suitable for X-ray analysis were obtained through overlayering a saturated CH_2Cl_2 solution with pentane.

Elemental analysis ($C_{28}H_{40}CoN_6O_6PF_{12}$ + DCM):

calcd.:	C 36.30%	H 4.41%	N 8.76%
found:	C 35.74%	H 4.78%	N 8.55%

UV-vis (CH_3CN): λ_{max} (ε / $M^{-1}cm^{-1}$): 232 ($2.11\cdot10^4$), 294 ($1.48\cdot10^4$), 362 ($0.80\cdot10^4$), 470 ($0.08\cdot10^4$), 713 (106) nm.

ESI-HRMS (pos., CH_2Cl_2): *m/z* (M-PF_6) = calcd. 729.2240, found 729.2239.

[Co(hfac)$_2$(L5)]PF$_6$

The neutral complex [Co(hfac)$_2$(L5)] (20 mg, 0.024 mmol) was dissolved in 3 mL dichloromethane. Then, ferrocenium hexafluorophosphate (7.9 mg, 0.024 mmol) was added; the colour of the solution turned dark. The reaction mixture was stirred for 2 h at room temperature. Subsequently, the precipitate was removed and the crude brown product washed with *n*-hexane (3 x 3 mL) to yield 23 mg (97%, 0.023 mmol) of [Co(hfac)$_2$(L5)]PF$_6$ as a brown solid. Crystals suitable for X-ray analysis were obtained through overlayering a saturated CH_2Cl_2 solution with pentane.

Elemental analysis ($C_{28}H_{34}CoN_6O_6PF_{18}$):

calcd.:	C 34.23%	H 3.49%	N 8.55%
found:	C 33.85%	H 4.05%	N 9.12%

UV-vis (CH_3CN): λ_{max} (ε / $M^{-1}cm^{-1}$): 230 ($3.18\cdot10^4$), 305 ($2.21\cdot10^4$), 470 ($0.15\cdot10^4$), shoulder at ca. 570 (338) nm.

ESI-HRMS (pos., CH_2Cl_2): *m/z* (M-PF_6) = calcd. 837.1675, found 837.1671.

2.4.6 Monocationic cobalt complexes of ligand L6

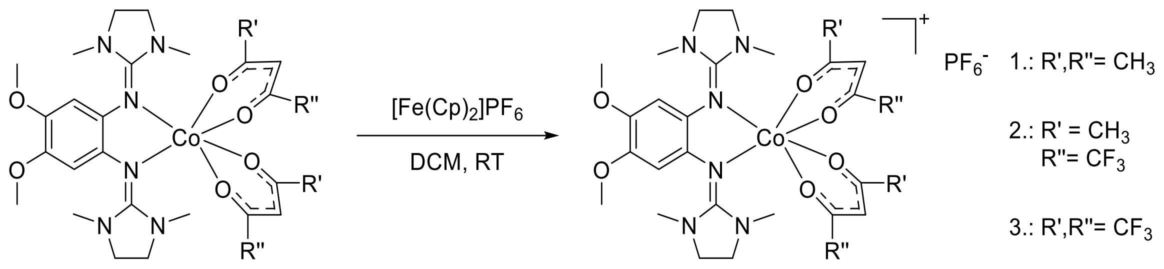

[Co(acac)$_2$(L6)]PF$_6$

The neutral complex [Co(acac)$_2$(L6)] (15.44 mg, 0.025 mmol) was dissolved in 3 mL dichloromethane. Then, ferrocenium hexafluorophosphate (8.28 mg, 0.025 mmol) was added; the colour of the solution turned to brown. The reaction mixture was stirred for 2 h at room temperature. Subsequently, the solvent was removed in vacuo and the crude brown product washed with *n*-hexane (3 x 3 mL) to yield 18 mg (94%, 0.024 mmol) of [Co(acac)$_2$(L6)]PF$_6$ as a red-brown solid.

Elemental analysis ($C_{28}H_{42}CoN_6O_6PF_6$ + 2 CH_2Cl_2):

calcd.:	C 38.64%	H 4.97%	N 9.01%
found:	C 38.32%	H 5.35%	N 10.01%

UV-vis (CH_3CN): λ_{max} (ε / $M^{-1}cm^{-1}$): 223 ($2.48\cdot10^4$), 275 ($1.27\cdot10^4$), 314 ($1.04\cdot10^4$), 462 ($0.11\cdot10^4$), 630 (67) nm.

ESI-HRMS (pos., CH_2Cl_2): *m/z* (M-Co(acac)$_2$PF$_6$) = calcd. 361.2347, found 361.2348.

[Co(tfac)$_2$(L6)]PF$_6$

The neutral complex [Co(tfac)$_2$(L6)] (21.88 mg, 0.03 mmol) was dissolved in 3 mL dichloromethane. Then, ferrocenium hexafluorophosphate (9.93 mg, 0.03 mmol) was added; the colour of the solution turned to green. The reaction mixture was stirred for 2 h at room temperature. Subsequently, the solvent was removed in vacuo and the crude green product washed with *n*-hexane (3 x 3 mL) to yield 24 mg (92%, 0.028 mmol) of [Co(tfac)$_2$(L6)]PF$_6$ as a green solid. Crystals suitable for X-ray analysis were obtained through overlayering a saturated CH_2Cl_2 solution with pentane.

Elemental analysis ($C_{28}H_{36}CoN_6O_6PF_{12}$ + 0.5 CH_2Cl_2):

calcd.:	C 37.49%	H 4.09%	N 9.21%
found:	C 37.49%	H 4.53%	N 9.71%

UV-vis (CH_3CN): λ_{max} (ε / $M^{-1}cm^{-1}$): 224 ($2.82\cdot10^4$), 293 ($1.67\cdot10^4$), 350 ($1.16\cdot10^4$), 457 ($0.22\cdot10^4$), ca. 600 (460) nm.

ESI-HRMS (pos., CH_2Cl_2): *m/z* (M-PF_6) = calcd. 725.1927, found 725.1930.

[Co(hfac)$_2$(L6)]PF$_6$

The neutral complex [Co(hfac)$_2$(L6)] (20.83 mg, 0.025 mmol) was dissolved in 3 mL dichloromethane. Then, ferrocenium hexafluorophosphate (8.28 mg, 0.025 mmol) was added; the colour of the solution turned dark. The reaction mixture was stirred for 2 h at room temperature. Subsequently, the solvent was removed in vacuo and the crude brown product washed with *n*-hexane (3 x 3 mL) to yield 23 mg (94%, 0.024 mmol) of [Co(hfac)$_2$(L6)]PF$_6$ as a green solid. Crystals suitable for X-ray analysis were obtained through overlayering a saturated CH_2Cl_2 solution with pentane.

Elemental analysis ($C_{28}H_{30}CoN_6O_6PF_{18}$):

calcd.:	C 34.37%	H 3.09%	N 8.59%
found:	C 33.76%	H 3.64%	N 8.44%

UV-vis (CH_3CN): λ_{max} (ε / $M^{-1}cm^{-1}$): 226 ($1.90\cdot10^4$), 306 ($1.35\cdot10^4$), 460 ($0.23\cdot10^4$), 657 (257) nm.

ESI-HRMS (pos., CH_2Cl_2): *m/z* (M-PF_6) = calcd. 833.1362, found 833.1360.

2.4.7 Monocationic cobalt complexes of ligand L7

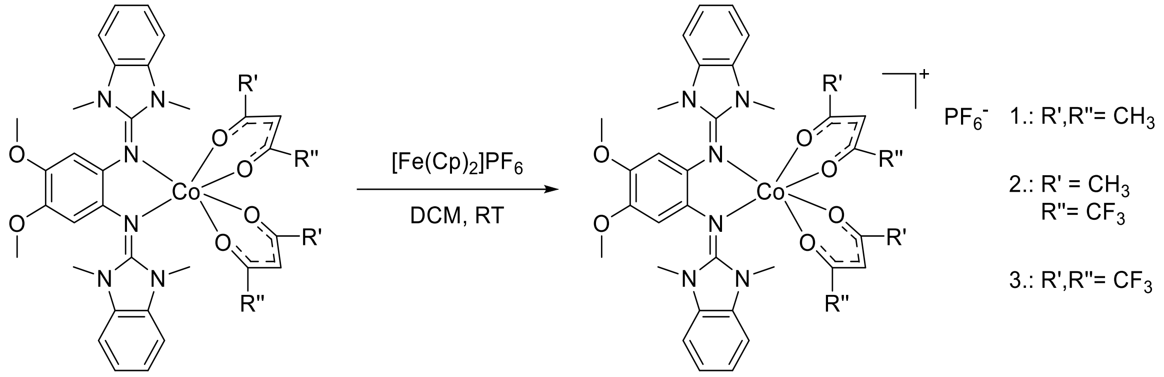

[Co(acac)$_2$(L7)]PF$_6$

The neutral complex [Co(acac)$_2$(L7)] (17.84 mg, 0.025 mmol) was dissolved in 3 mL dichloromethane. Then, ferrocenium hexafluorophosphate (8.28 mg, 0.025 mmol) was added; the colour of the solution turned to brown. The reaction mixture was stirred for 2 h at room temperature. Subsequently, the solvent was removed in vacuo and the crude brown product washed with *n*-hexane (3 x 3 mL) to yield 21 mg (98%, 0.024 mmol) of [Co(acac)$_2$(L1)]PF$_6$ as a red-brown solid. Crystals suitable for X-ray analysis were obtained through overlayering a saturated CH_2Cl_2 solution with pentane. As the crystalisation was not possible with PF_6^- as counterion it was crystalised with SbF_6^- as counterion.

Elemental analysis ($C_{36}H_{42}CoN_6O_6PF_6$ + 2 CH_2Cl_2):

calcd.:	C 44.38%	H 4.51%	N 8.17%
found:	C 44.85%	H 4.99%	N 8.50%

UV-vis (CH_3CN): λ_{max} (ε / $M^{-1}cm^{-1}$): 200 ($5.36 \cdot 10^4$), 226 ($3.21 \cdot 10^4$), 282 ($1.87 \cdot 10^4$), 327 ($1.40 \cdot 10^4$), 525 ($0.13 \cdot 10^4$), 735 (184) nm.

ESI-HRMS (pos., CH_2Cl_2): *m/z* (M-Co(acac)$_2$PF$_6$) = calcd. 456.2274, found 456.2269.

[Co(tfac)$_2$(L7)]PF$_6$

The neutral complex [Co(tfac)$_2$(L7)] (24.65 mg, 0.03 mmol) was dissolved in 3 mL dichloromethane. Then, ferrocenium hexafluorophosphate (9.93 mg, 0.03 mmol) was added; the colour of the solution turned to green. The reaction mixture was stirred for

2 h at room temperature. Subsequently, the solvent was removed in vacuo and the crude green product washed with *n*-hexane (3 x 3 mL) to yield 28 mg (97%, 0.029 mmol) of [Co(tfac)$_2$(L1)]PF$_6$ as a green solid. Crystals suitable for X-ray analysis were obtained through overlayering a saturated CH_2Cl_2 solution with pentane.

Elemental analysis ($C_{36}H_{36}CoN_6O_6PF_{12}$):

calcd.:	C 44.73%	H 3.75%	N 8.69%
found:	C 44.49%	H 4.09%	N 7.97%

UV-vis (CH_3CN): λ_{max} (ε / $M^{-1}cm^{-1}$): 201 ($5.36 \cdot 10^4$), 287 ($2.73 \cdot 10^4$), 379 ($0.99 \cdot 10^4$), 464 ($0.12 \cdot 10^4$), 531 ($0.13 \cdot 10^4$), 728 (164) nm.

ESI-HRMS (pos., CH_2Cl_2): *m/z* (M-PF$_6$) = calcd. 821.1927, found 821.1927.

[Co(hfac)$_2$(L7)]PF$_6$

The neutral complex [Co(hfac)$_2$(L7)] (23.22 mg, 0.025 mmol) was dissolved in 3 mL dichloromethane. Then, ferrocenium hexafluorophosphate (8.28mg, 0.025 mmol) was added; the colour of the solution turned dark. The reaction mixture was stirred for 2 h at room temperature. Subsequently, the solvent was removed in vacuo and the crude brown product washed with *n*-hexane (3 x 3 mL) to yield 25.5 mg (96%, 0.024 mmol) of [Co(hfac)$_2$(L1)]PF$_6$ as a brown solid. Crystals suitable for X-ray analysis were obtained through overlayering a saturated CH_2Cl_2 solution with pentane.

Elemental analysis ($C_{36}H_{30}CoN_6O_6PF_{18}$):

calcd.:	C 40.96%	H 3.60%	N 6.82%
found:	C 40.12%	H 3.83%	N 6.83%

UV-vis (CH_3CN): λ_{max} (ε / $M^{-1}cm^{-1}$): 200 ($5.20 \cdot 10^4$), 285 ($2.08 \cdot 10^4$), 316 ($1.61 \cdot 10^4$), 463 ($0.24 \cdot 10^4$), 742 (108) nm.

ESI-HRMS (pos., CH_2Cl_2): *m/z* (M-PF$_6$) = calcd. 929.1362, found 929.1361.

2.5 Analytical data for the oxidized, monocationic complexes

2.5.1 UV-Vis and IR spectra

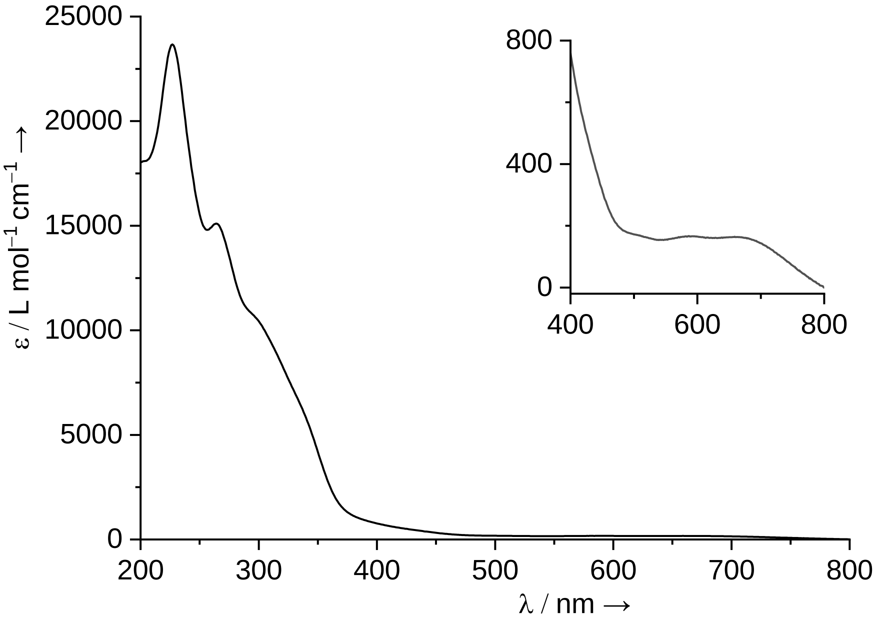

UV-vis spectrum (CH_3CN) of [Co(acac)$_2$(L1)]PF_6.

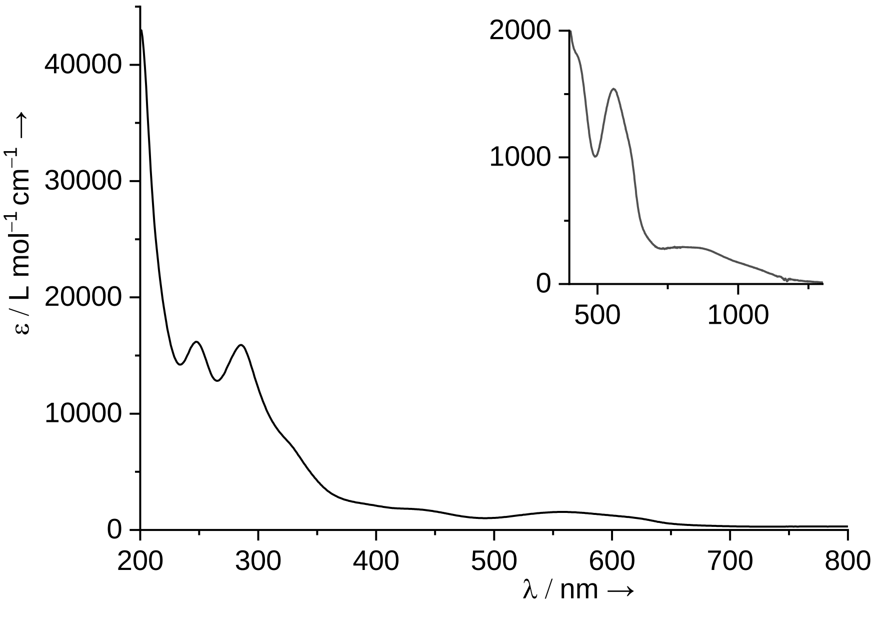

UV-vis spectrum (CH_3CN) of [Co(tfac)$_2$(L1)]PF_6.

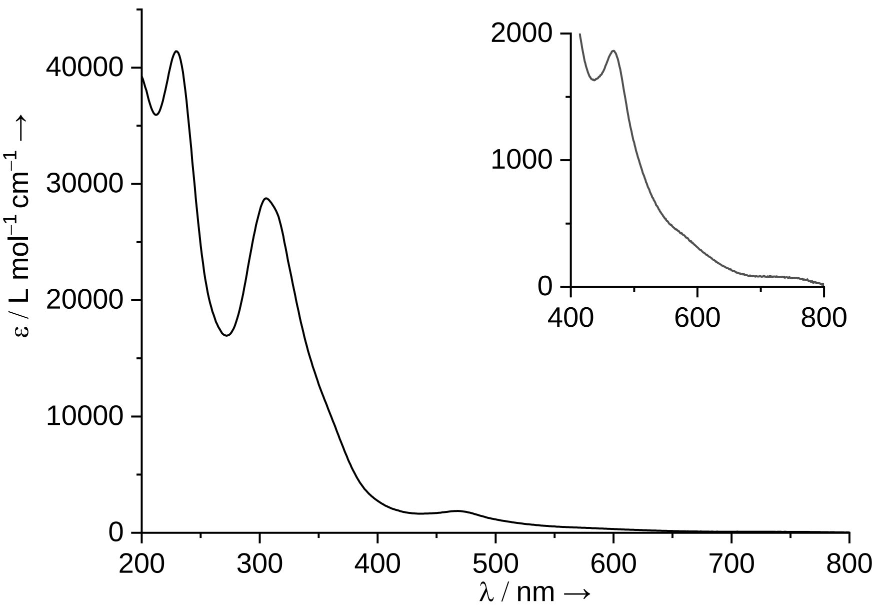

UV-vis spectrum (CH_3CN) of [Co(hfac)$_2$(L1)]PF_6.

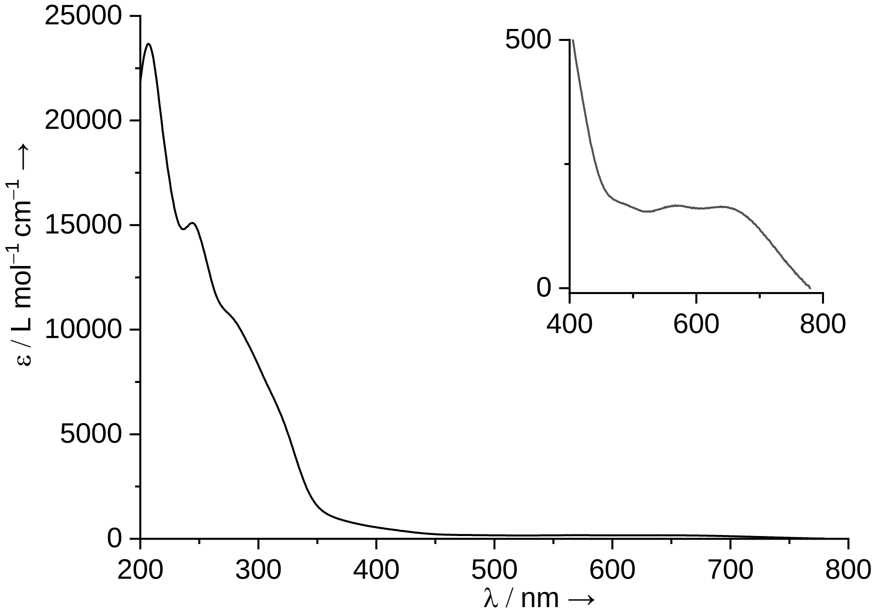

UV-vis spectrum (CH_3CN) of [Co(acac)$_2$(L2)]PF_6.

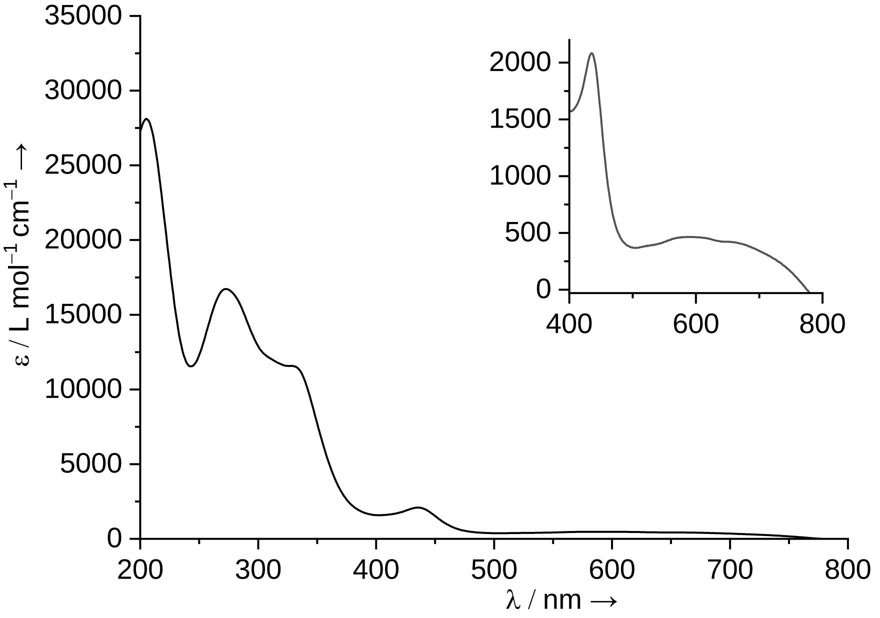

UV-vis spectrum (CH_3CN) of [Co(tfac)$_2$(L2)]PF_6.

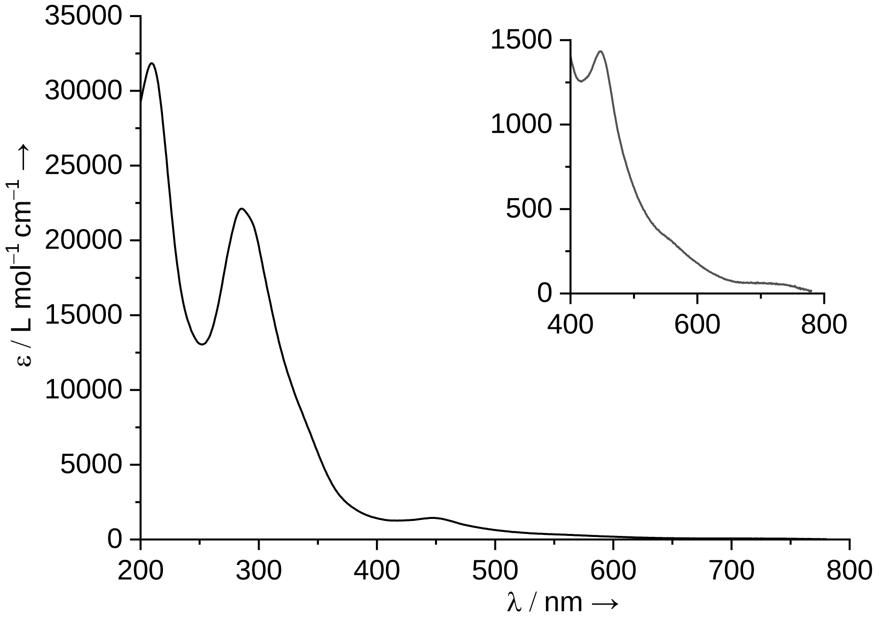

UV-vis spectrum (CH_3CN) of [Co(hfac)$_2$(L2)]PF_6.

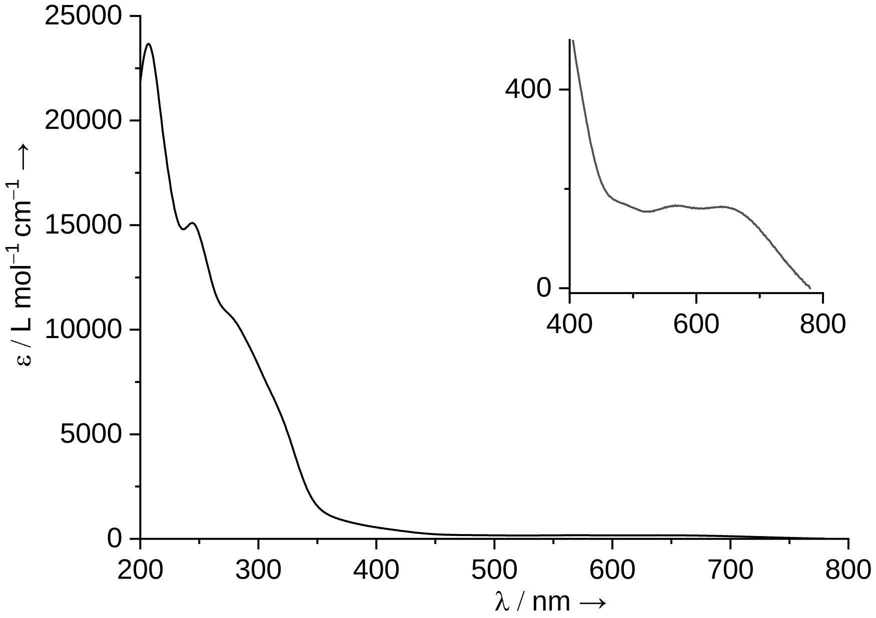

UV-vis spectrum (CH_3CN) of [Co(acac)$_2$(L3)]PF_6.

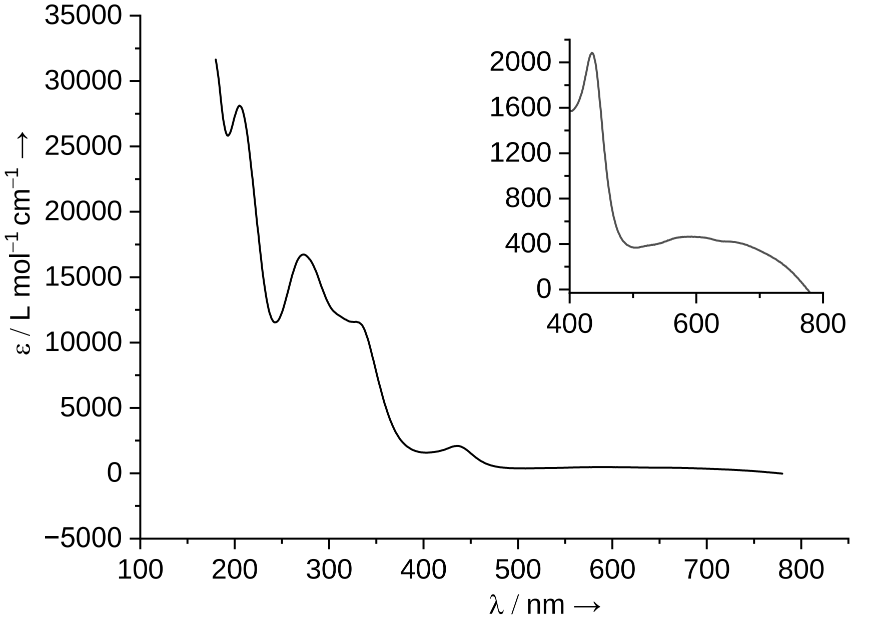

UV-vis spectrum (CH_3CN) of [Co(tfac)$_2$(L3)]PF_6.

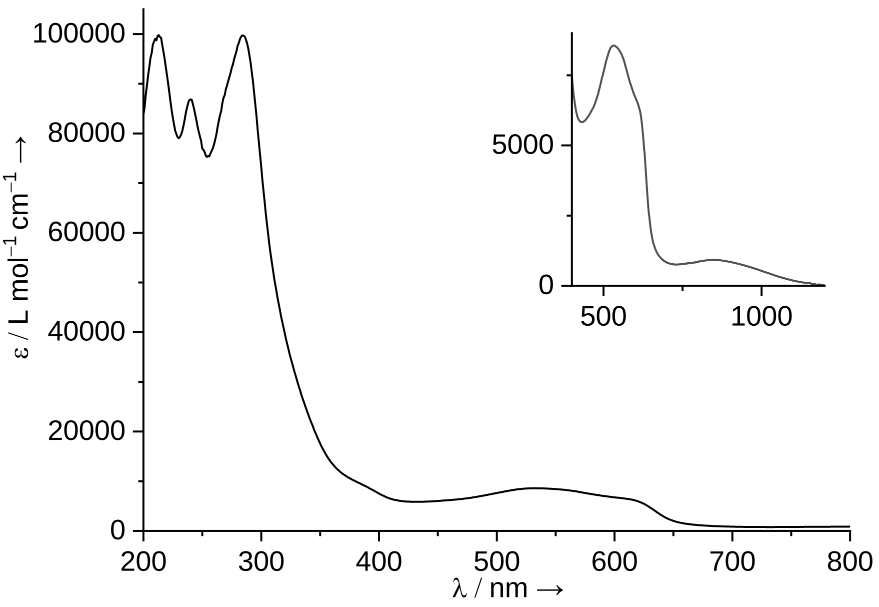

UV-vis spectrum (CH_3CN) of [Co(hfac)$_2$(L3)]PF_6.

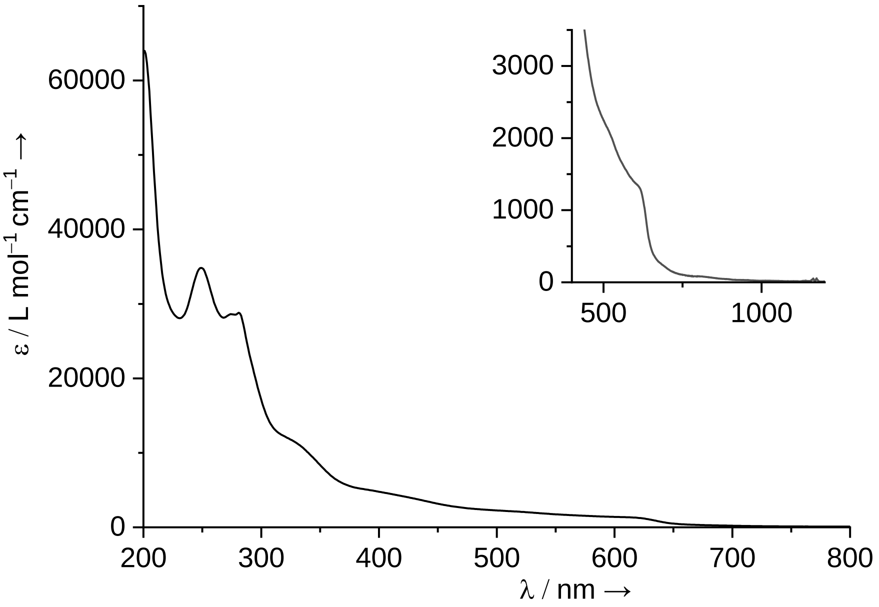

UV-vis spectrum (CH_3CN) of [Co(acac)$_2$(L4)]PF_6.

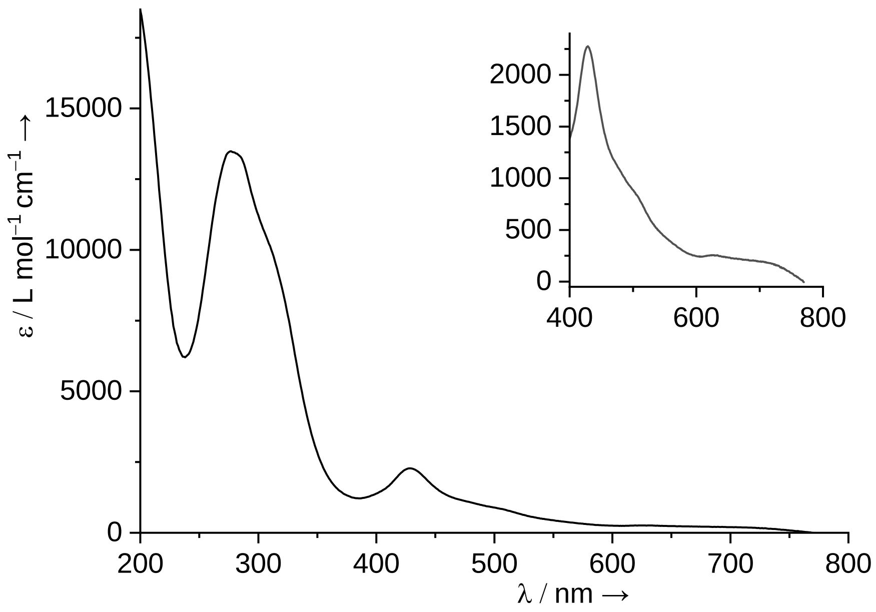

UV-vis spectrum (CH_3CN) of [Co(tfac)$_2$(L4)]PF_6.

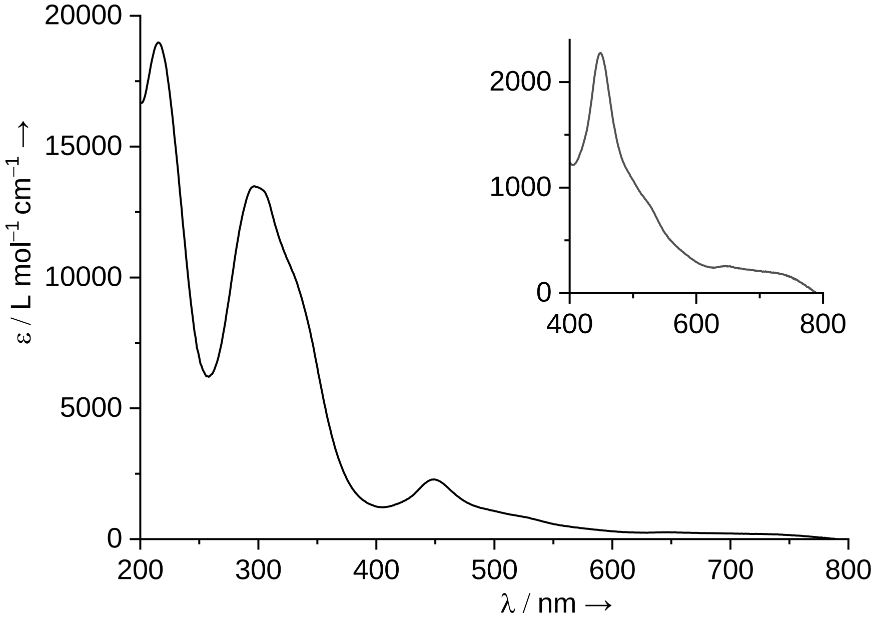

UV-vis spectrum (CH_3CN) of [Co(hfac)$_2$(L4)]PF_6.

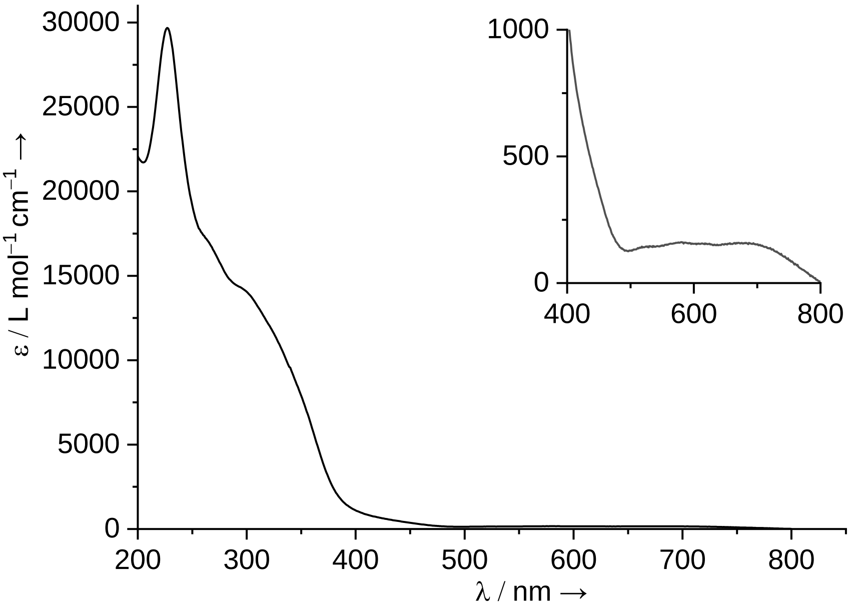

UV-vis spectrum (CH_3CN) of [Co(acac)$_2$(L5)]PF_6.

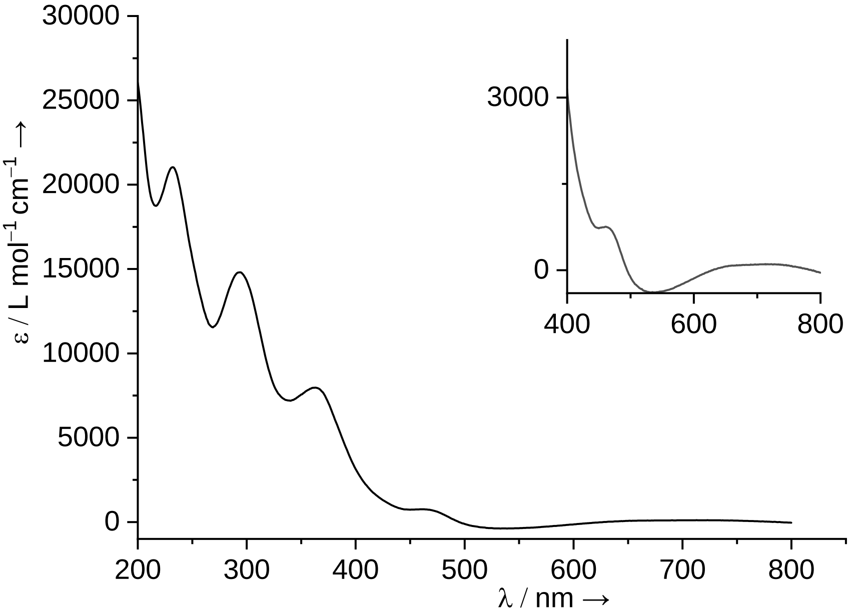

UV-vis spectrum (CH_3CN) of [Co(tfac)$_2$(L5)]PF_6.

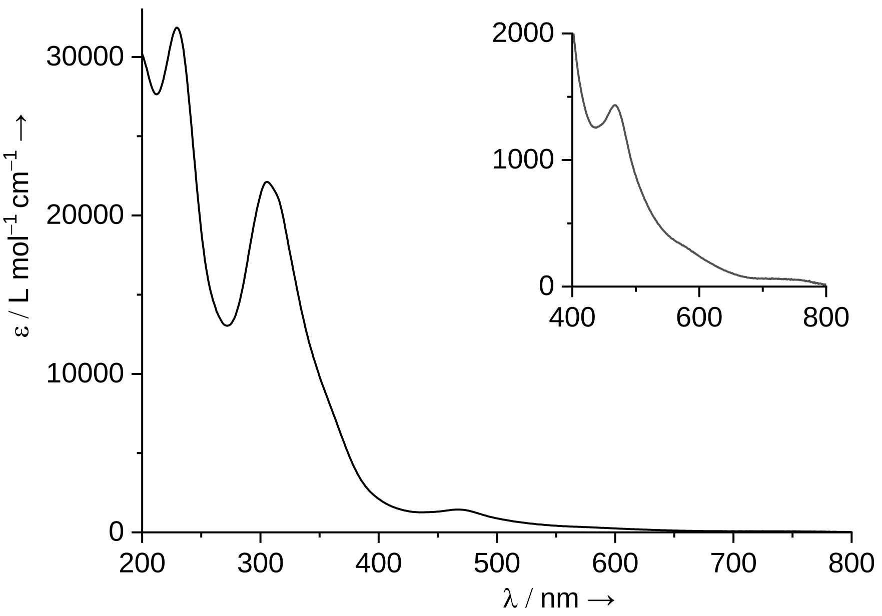

UV-vis spectrum (CH_3CN) of [Co(hfac)$_2$(L5)]PF_6.

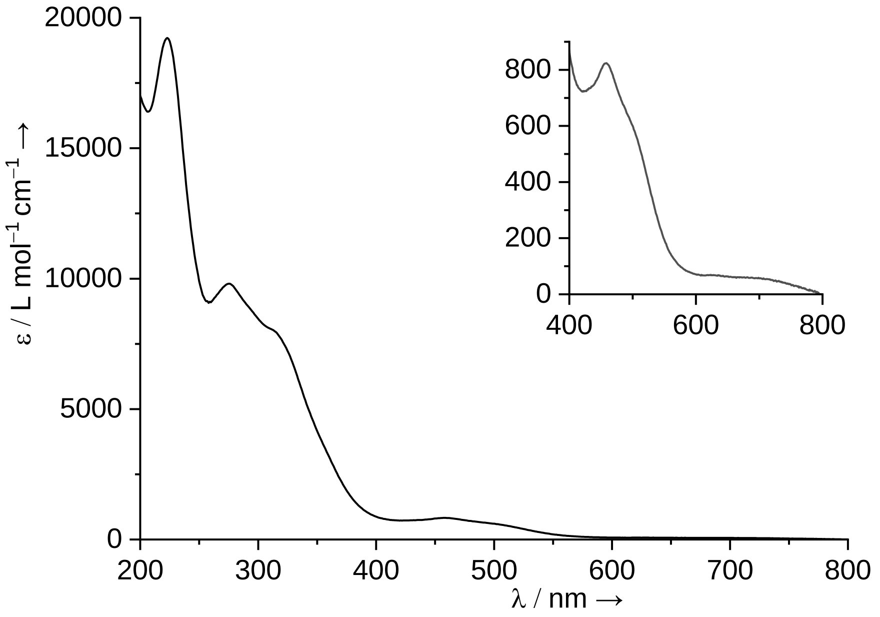

UV-vis spectrum (CH_3CN) of [Co(acac)$_2$(L6)]PF_6.

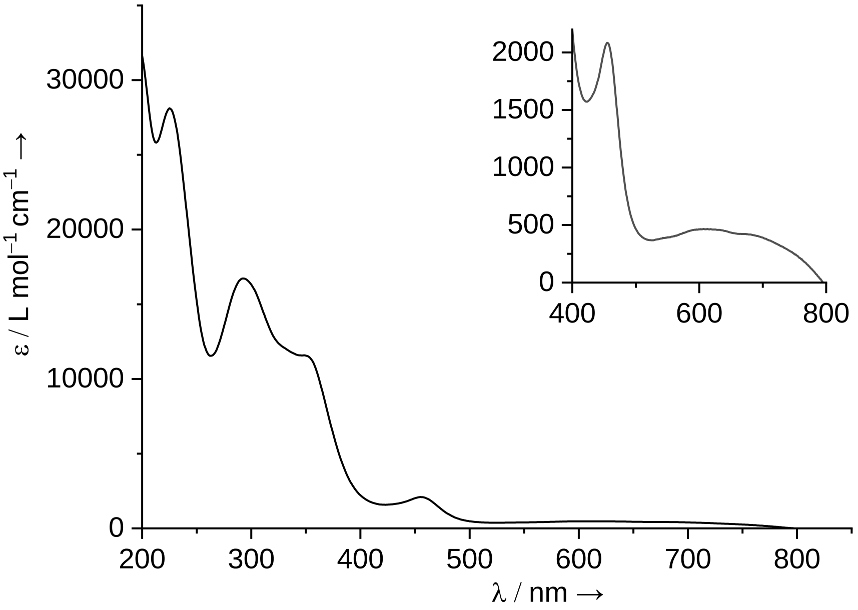

UV-vis spectrum (CH_3CN) of [Co(tfac)$_2$(L6)]PF_6.

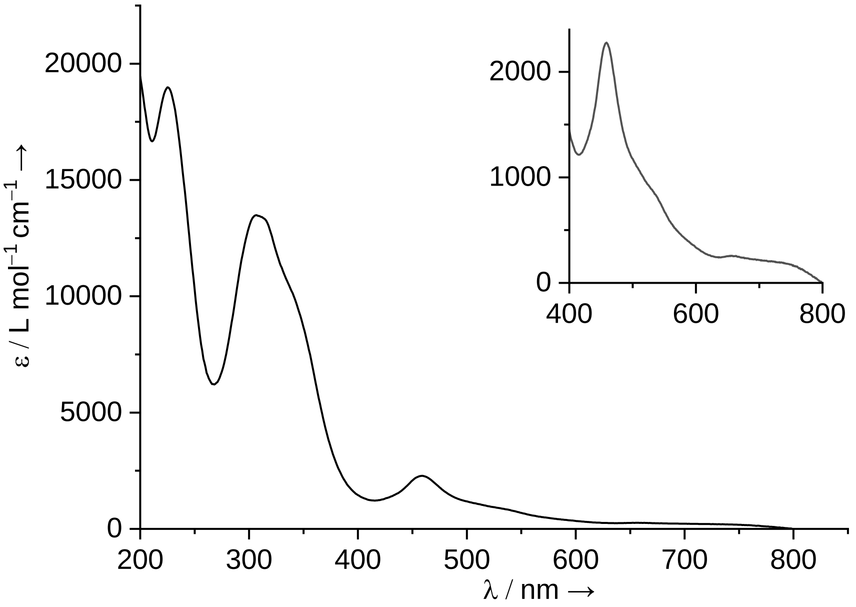

UV-vis spectrum (CH_3CN) of [Co(hfac)$_2$(L6)]PF_6.

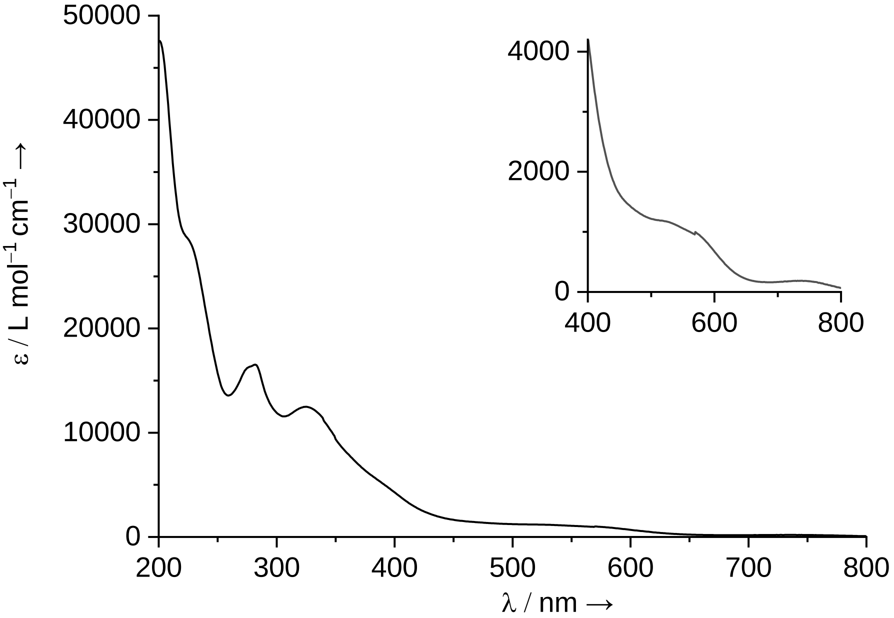

UV-vis spectrum (CH_3CN) of [Co(acac)$_2$(L7)]PF_6.

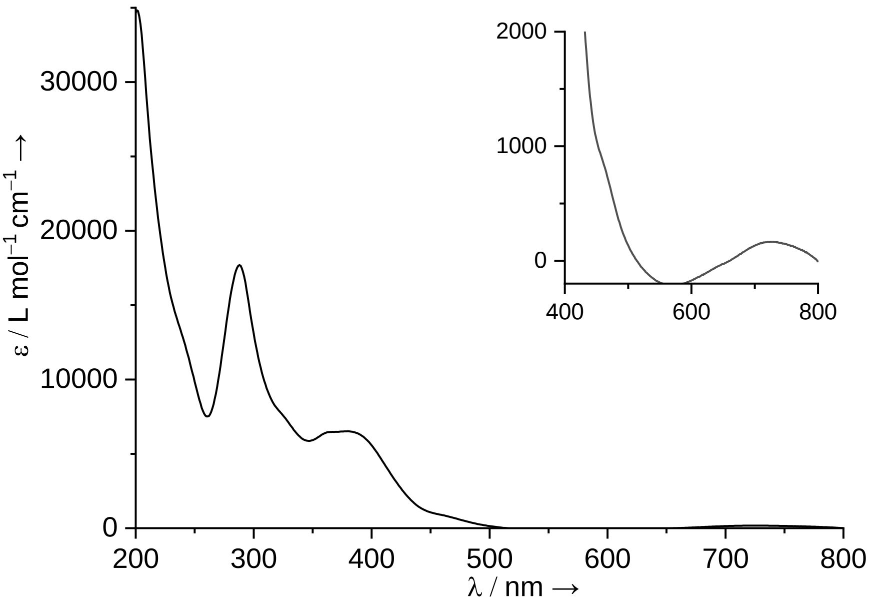

UV-vis spectrum (CH_3CN) of [Co(tfac)$_2$(L7)]PF_6.

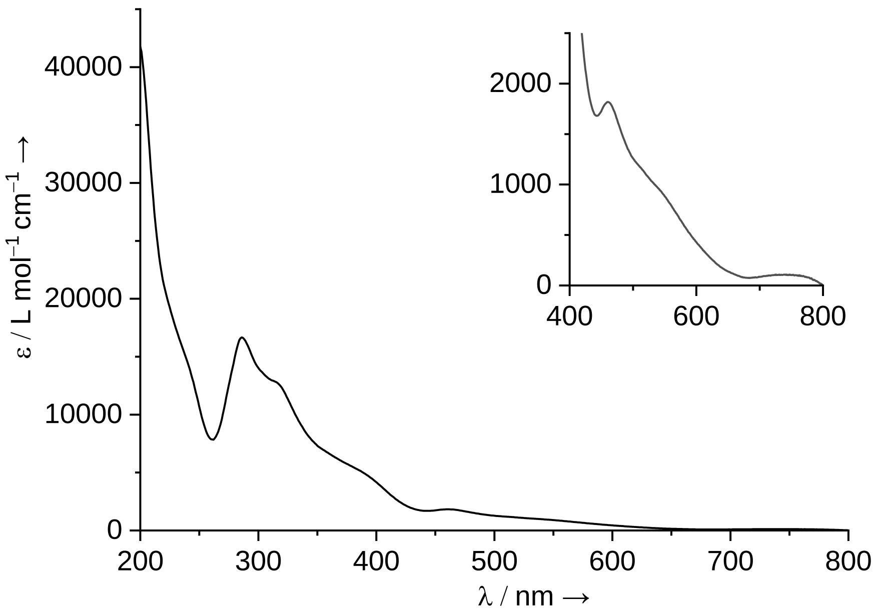

UV-vis spectrum (CH_3CN) of [Co(hfac)$_2$(L7)]PF_6.

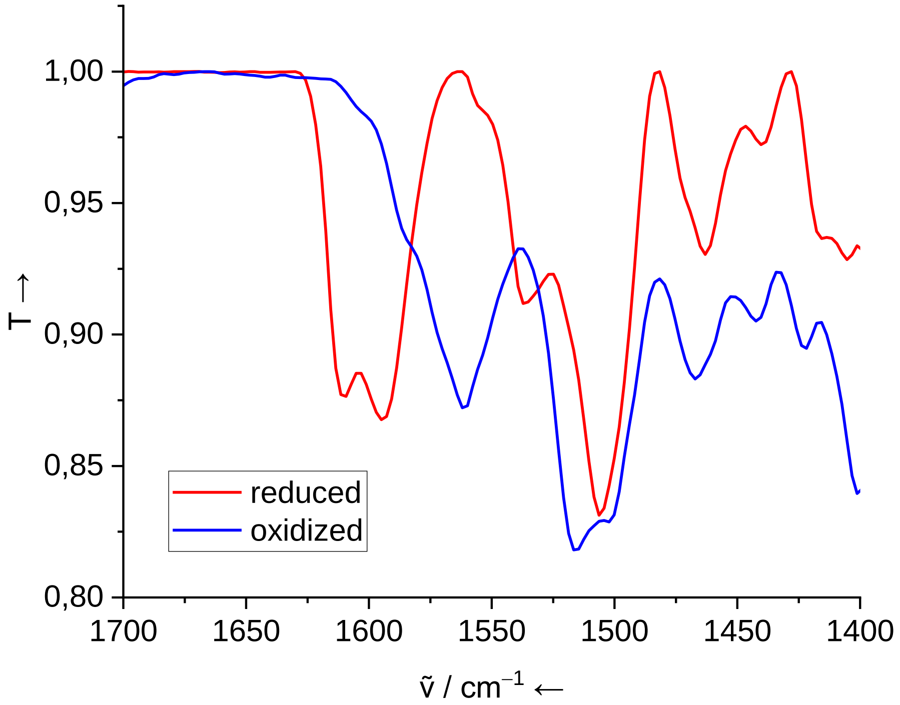

Comparison between the IR spectra of [Co(acac)$_2$(L5)] (reduced form, red curve) and [Co(acac)$_2$(L5)]PF$_6$ (oxidized form, blue curve) in the region 1700-1400 cm^{-1}.

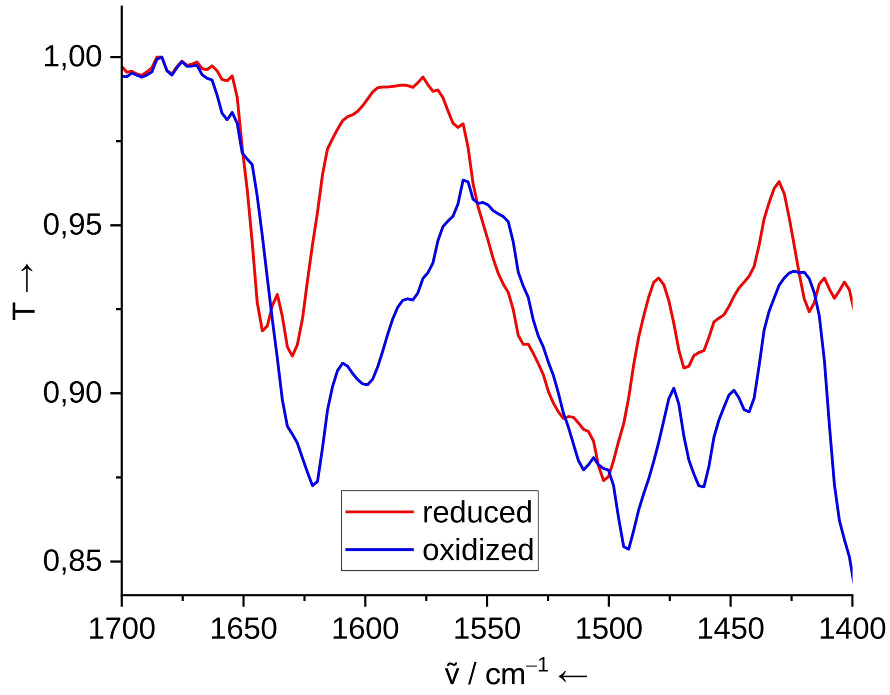

Comparison between the IR spectra of [Co(tfac)$_2$(L5)] (reduced form, red curve) and [Co(tfac)$_2$(L5)]PF$_6$ (oxidized form, blue curve) in the region 1700-1400 cm^{-1}.

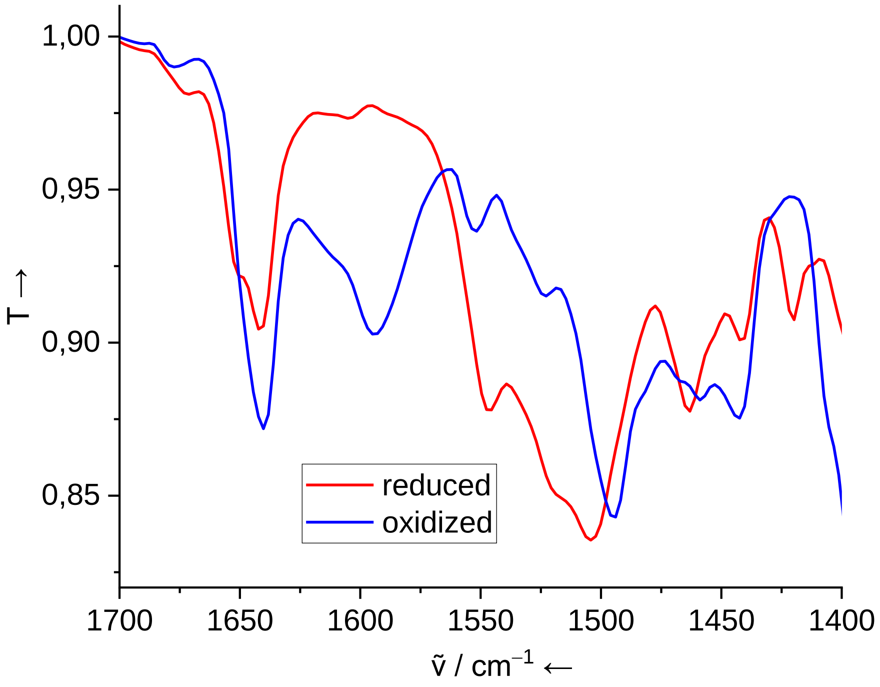

Comparison between the IR spectra of [Co(hfac)$_2$(L5)] (reduced form, red curve) and [Co(hfac)$_2$(L5)]PF$_6$ (oxidized form, blue curve) in the region 1700-1400 cm^{-1}.

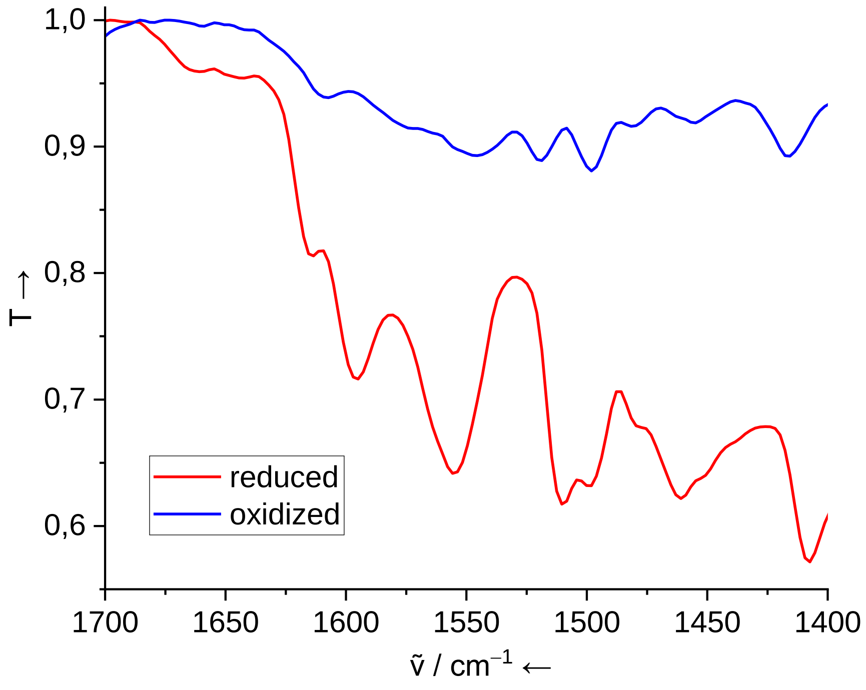

Comparison between the IR spectra of [Co(acac)$_2$(L6)] (reduced form, red curve) and [Co(acac)$_2$(L6)]PF$_6$ (oxidized form, blue curve) in the region 1700-1400 cm^{-1}.

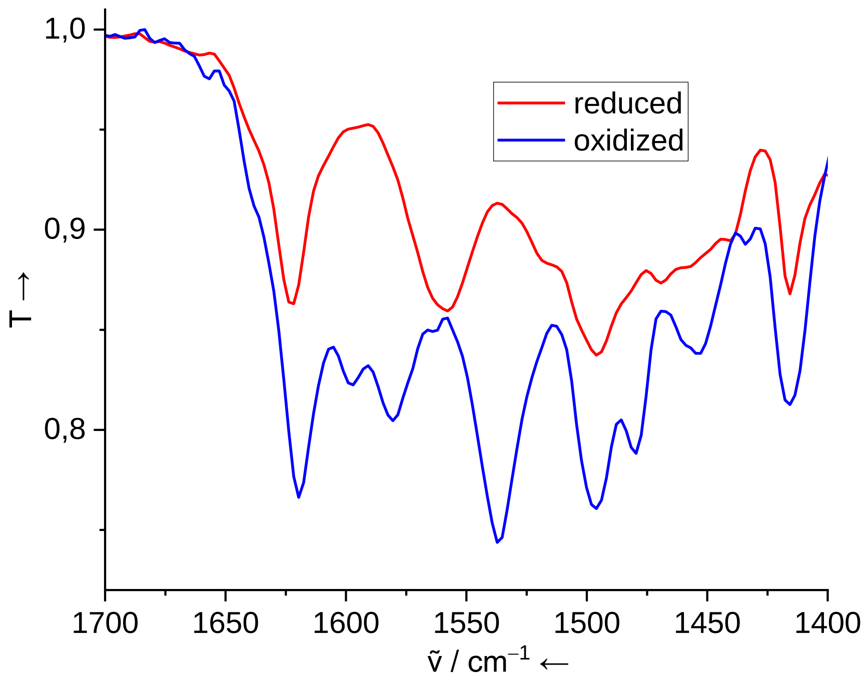

Comparison between the IR spectra of [Co(tfac)$_2$(L6)] (reduced form, red curve) and [Co(tfac)$_2$(L6)]PF_6 (oxidized form, blue curve) in the region 1700-1400 cm^{-1}.

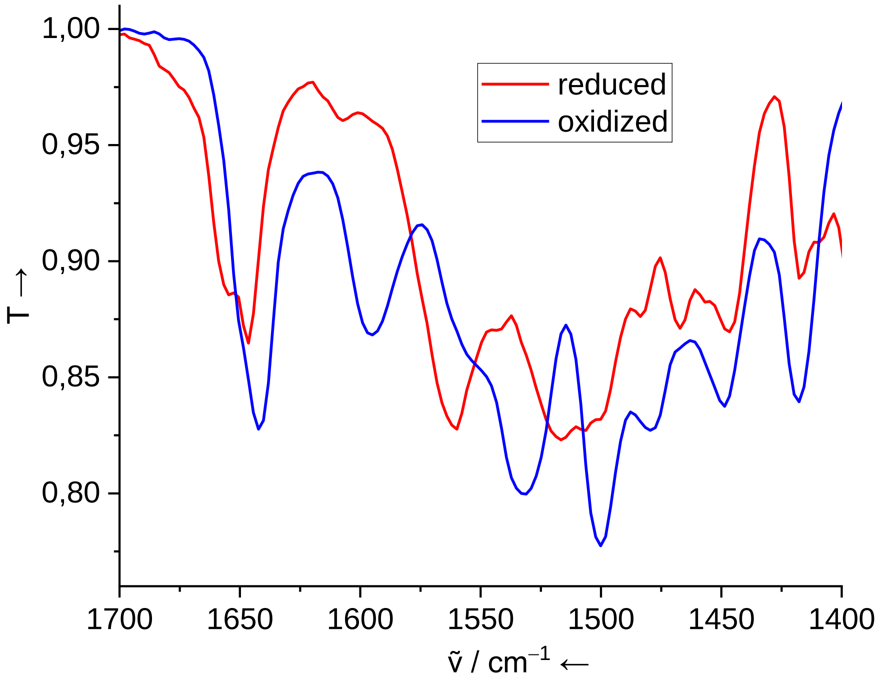

Comparison between the IR spectra of [Co(hfac)$_2$(L6)] (reduced form, red curve) and [Co(hfac)$_2$(L6)]PF_6 (oxidized form, blue curve) in the region 1700-1400 cm^{-1}.

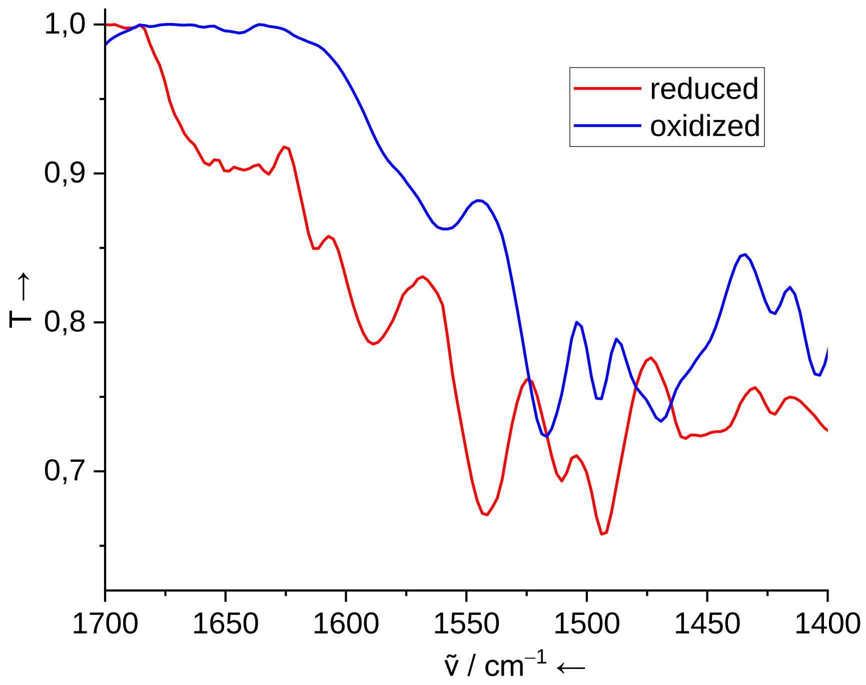

Comparison between the IR spectra of [Co(acac)$_2$(L7)] (reduced form, red curve) and [Co(acac)$_2$(L7)]PF_6 (oxidized form, blue curve) in the region 1700-1400 cm^{-1}.

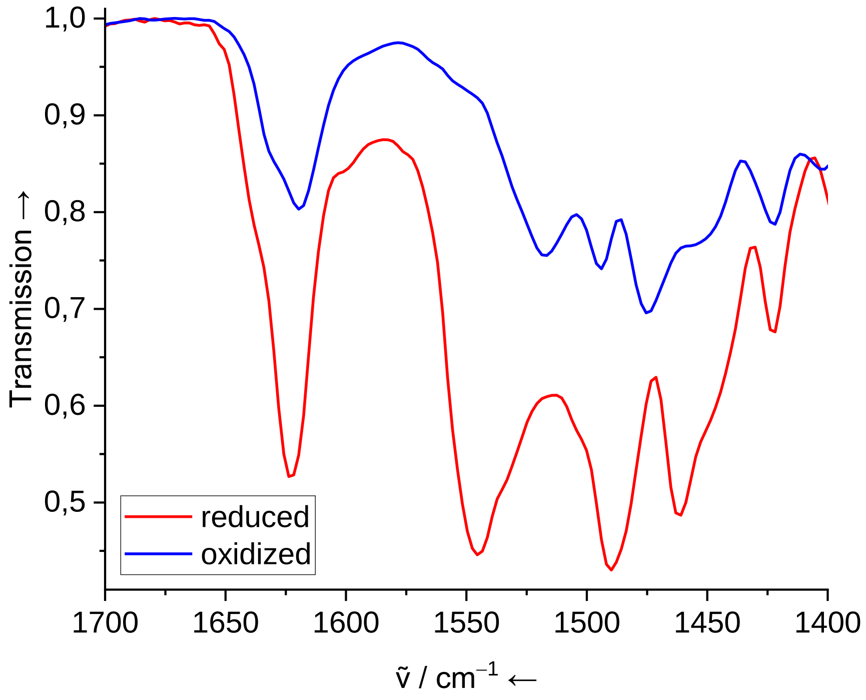

Comparison between the IR spectra of [Co(tfac)$_2$(L7)] (reduced form, red curve) and [Co(tfac)$_2$(L7)]PF_6 (oxidized form, blue curve) in the region 1700-1400 cm^{-1}.

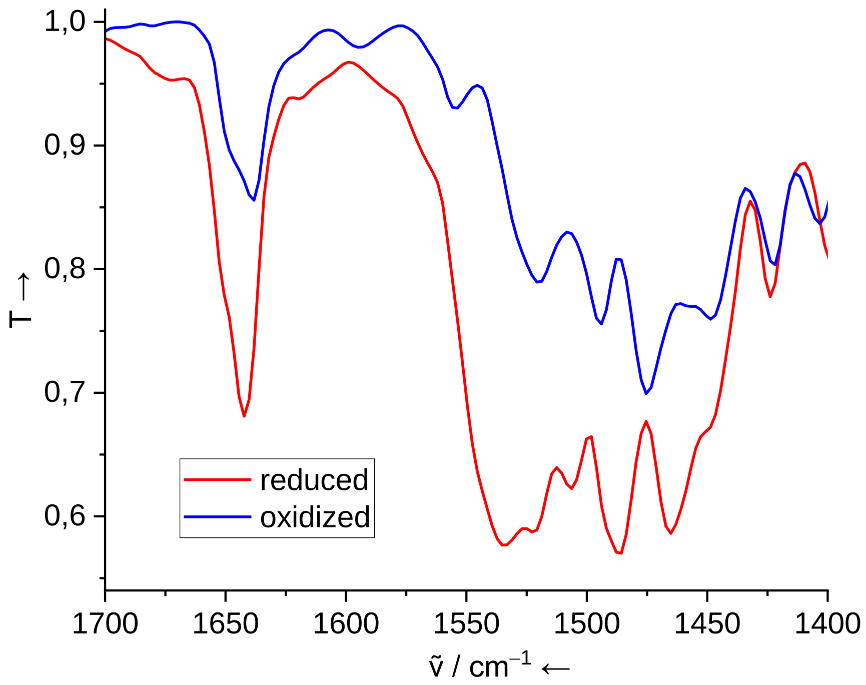

Comparison between the IR spectra of [Co(hfac)$_2$(L7)] (reduced form, red curve) and [Co(hfac)$_2$(L7)]PF_6 (oxidized form, blue curve) in the region 1700-1400 cm^{-1}.

2.5.2 Crystallografic data for the oxidized, monocationic complexes

Selected bond lengths in Å for the oxidized complexes [Co(acac)$_2$(L1)]PF$_6$, [Co(hfac)$_2$(L1)][Co(hfac)$_3$], [Co(hfac)$_2$(L2)]PF$_6$ and [Co(hfac)$_2$(L3)]PF$_6$ in the solid-state from SCXRD experiments at 100 K.

Bond	[Co(acac)$_2$(L1)]PF$_6$	[Co(hfac)$_2$(L1)] [Co(hfac)$_3$]	[Co(hfac)$_2$(L2)] [Co(hfac)$_3$]	[Co(hfac)$_2$(L3)]PF$_6$
Name	Jo198Coox	Jol1hf2ox nein 1ox	Jol2hfox	Jol3hfox
Co1 O1	1.8967(19)	2.0840(18)	2.053(2)	2.087(2)
Co1 O2	1.8942(19)	2.0817(17)	2.056(2)	2.083(2)
Co1 O3	1.8869(19)	2.0638(17)	2.071(2)	2.080(2)
Co1 O4	1.8943(19)	2.0764(18)	2.085(2)	2.047(2)
Co1 N1	1.964(2)	2.086(2)	2.094(3)	2.072(2)
Co1 N4	1.976(2)	2.089(2)	2.097(2)	2.103(3)
N1 C1	1.414(3)	1.354(3)	1.353(4)	1.352(4)
N4 C2	1.410(3)	1.355(3)	1.358(4)	1.360(4)
C1 C2	1.393(4)	1.448(3)	1.446(4)	1.443(4)
C1 C6	1.393(4)	1.415(3)	1.413(4)	1.416(4)
C2 C3	1.406(4)	1.409(3)	1.415(4)	1.413(4)
C3 C4	1.386(4)	1.371(3)	1.373(4)	1.370(5)
C4 C5	1.405(4)	1.442(3)	1.441(4)	1.444(4)
C5 C6	1.389(4)	1.371(3)	1.371(4)	1.374(4)

Selected bond lengths in Å for the oxidized complexes [Co(acac)$_2$(L5)], [Co(tfac)$_2$(L5)]PF$_6$ in the solid-state from SCXRD experiments at 100 K.

Bond	[Co(acac)$_2$(L5)]PF$_6$	[Co(tfac)$_2$(L5)]PF$_6$
Name	Jomk21_2	Jomk24
Co1 O3	1.882(5)	2.075(4)
Co1 O4	1.899(5)	2.069(4)
Co1 O5	1.898(5)	2.035(4)
Co1 O6	1.899(5)	2.070(4)
Co1-N1	1.955(5)	2.104(4)
Co1-N4	1.957(6)	2.095(4)
N1-C1	1.387(9)	1.348(6)
N4-C2	1.414(8)	1.362(6)
C1-C2	1.404(9)	1.443(7)
C1-C6	1.409(9)	1.415(7)
C2-C3	1.388(10)	1.410(7)
C3-C4	1.374(10)	1.376(7)
C4-C5	1.406(10)	1.440(8)
C5-C6	1.375(10)	1.372(7)

Selected bond lengths in Å for the oxidized complexes $[Co(tfac)_2(L6)]PF_6$ and $[Co(hfac)_2(L6)]PF_6$ in the solid-state from SCXRD experiments at 100 K.

Bond	$[Co(tfac)_2(L6)]PF_6$	$[Co(hfac)_2(L6)]PF_6$
Name	Jomk22	Jomk26_grun
Co1 O3	2.0711(12)	2.0947(17)
Co1 O4	2.0587(13)	2.0816(17)
Co1 O5	2.0711(12)	2.0874(16)
Co1 O6	2.0587(13)	2.0680(16)
Co1-N1	2.103(2)	2.0909(19)
Co1-N4	2.103(2)	2.0909(19)
N1-C1	1.351(2)	1.362(3)
N4-C2	1.351(2)	1.350(3)
C1-C2	1.452(3)	1.450(3)
C1-C6	1.416(2)	1.407(3)
C2-C3	1.416(2)	1.417(3)
C3-C4	1.367(2)	1.367(3)
C4-C5	1.448(3)	1.439(3)
C5-C6	1.367(2)	1.367(3)

Selected bond lengths in Å for the oxidized complexes $[Co(acac)_2(L7)](SbF_6)_2$, $[Co(tfac)_2(L7)]PF_6$ and $[Co(hfac)_2(L7)]PF_6$ in the solid-state from SCXRD experiments at 100 K.

Bond	$[Co(acac)_2(L7)](SbF_6)_2$	$[Co(tfac)_2(L7)]PF_6$	$[Co(hfac)_2(L7)]PF_6$
Name	Jocol4	Jomk36	Jomk37
Co1 O3	1.8901(15)	2.051(3)	2.081(3)
Co1 O4	1.8902(15)	2.069(3)	2.048(3)
Co1 O5	1.8801(15)	2.047(3)	2.076(3)
Co1 O6	1.8801(15)	2.044(3)	2.062(3)
Co1 N1	1.9442(17)	2.086(4)	2.121(4)
Co1 N4	1.9442(17)	2.097(4)	2.086(4)
N1 C1	1.358(3)	1.359(2)	1.355(6)
N4 C2	1.358(3)	1.357(5)	1.350(6)
C1 C2	1.445(4)	1.446(6)	1.468(6)
C1 C6	1.403(3)	1.409(6)	1.418(6)
C2 C3	1.403(3)	1.401(6)	1.397(6)
C3 C4	1.368(3)	1.363(6)	1.373(7)
C4 C5	1.458(5)	1.445(6)	1.452(7)
C5 C6	1.368(3)	1.365(6)	1.370(7)

**Compound**	**[Co(acac)$_2$(L1)]PF$_6$**	**[Co(hfac)$_2$(L1)][Co(hfac)$_3$]**	**[Co(hfac)$_2$(L2)][Co(hfac)$_3$]**	**[Co(hfac)$_2$(L3)]PF$_6$**
Identification code	mo_jo198Coox_0ma	mo_2023_jol1hf2ox_0m	mo_2023_jol2hfox_0m	mo_2023_jol3hfox_2_0ma
Empirical formula	$C_{28}H_{46}CoF_6N_6O_4P$	$C_{43}H_{37}Co_2F_{30}N_6O_{10}$	$C_{43}H_{33}Co_2F_{30}N_6O_{10}$	$C_{36}H_{30}CoF_{18}N_6O_4P$ * 0.1 CH_2Cl_2
Formula weight	734.61	1485.64	1481.61	1051.05
Temperature/K	100.00	100.00	100.00	100.00
Crystal system	monoclinic	triclinic	triclinic	monoclinic
Space group	C2/c	P-1	P-1	P2$_1$/c
a/Å	36.274(4)	12.418(4)	12.342(4)	10.001(2)
b/Å	8.2535(7)	15.669(5)	16.115(4)	20.263(6)
c/Å	25.341(3)	16.482(5)	16.426(5)	21.924(6)
α/°	90	110.147(11)	117.704(10)	90
β/°	111.660(6)	96.143(12)	105.002(11)	102.435(10)
γ/°	90	104.566(12)	90.924(11)	90
Volume/Å^3	7050.9(12)	2847.2(15)	2758.0(14)	4339(2)
Z	8	2	2	4
ρ_{calc}g/cm^3	1.384	1.733	1.784	1.609
μ/mm^{-1}	0.604	0.739	0.763	0.568
F(000)	3072.0	1482.0	1474.0	2113.0
Crystal size/mm^3	0.4 × 0.1 × 0.1	0.433 × 0.111 × 0.092	0.246 × 0.15 × 0.074	0.298 × 0.186 × 0.046
Radiation	MoKα (λ = 0.71073)	MoKα (λ = 0.71073)	MoKα (λ = 0.71073)	MoKα (λ = 0.71073)
2Θ range for data collection/°	4.794 to 52	3.912 to 52	4.14 to 52.996	3.804 to 51.998
Index ranges	-44 ≤ h ≤ 44, -10 ≤ k ≤ 10, -31 ≤ l ≤ 31	-15 ≤ h ≤ 15, -19 ≤ k ≤ 19, -20 ≤ l ≤ 20	-15 ≤ h ≤ 15, -20 ≤ k ≤ 20, -20 ≤ l ≤ 20	-12 ≤ h ≤ 12, -24 ≤ k ≤ 24, -27 ≤ l ≤ 27
Reflections collected	185753	124633	134858	179005
Independent reflections	6912 [R_{int} = 0.1130, R_{sigma} = 0.0283]	11171 [R_{int} = 0.1042, R_{sigma} = 0.0455]	11446 [R_{int} = 0.1094, R_{sigma} = 0.0527]	8526 [R_{int} = 0.1991, R_{sigma} = 0.0877]

Data/restraints/parameters	6912/0/429	11171/84/857	11446/0/844	8526/0/629
Goodness-of-fit on F^2	1.087	1.032	1.031	1.048
Final R indexes [I>=2σ (I)]	R_1 = 0.0472, wR_2 = 0.1003	R_1 = 0.0391, wR_2 = 0.0844	R_1 = 0.0463, wR_2 = 0.1095	R_1 = 0.0494, wR_2 = 0.1131
Final R indexes [all data]	R_1 = 0.0598, wR_2 = 0.1104	R_1 = 0.0518, wR_2 = 0.0908	R_1 = 0.0667, wR_2 = 0.1233	R_1 = 0.0728, wR_2 = 0.1346
Largest diff. peak/hole / e $Å^{-3}$	0.46/-0.55	0.52/-0.44	0.72/-0.59	0.53/-0.43

Compound	$[Co(acac)_2(L5)]PF_6$	$[Co(tfac)_2(L5)]PF_6$	$[Co(tfac)_2(L6)]PF_6$	$[Co(hfac)_2(L6)]PF_6$
Identification code	mo_2022_jomk21_2_0ma	mo_2022_jomk24_0m	mo_2022_jomk22_01a	mo_2022_jomk26gruen_01a
Empirical formula	$C_{28}H_{46}CoF_6N_6O_6P$	$C_{28}H_{40}CoF_{12}N_6O_6P$	$C_{28}H_{36}CoF_{12}N_6O_6P$	$C_{28}H_{30}CoF_{18}N_6O_6P$
Formula weight	766.61	874.56	870.53	978.48
Temperature/K	100.00	100.00	100.00	100.00
Crystal system	monoclinic	triclinic	orthorhombic	monoclinic
Space group	$P2_1/c$	P-1	Pcca	C2/c
a/Å	8.3434(8)	12.1355(10)	22.0821(10)	36.901(2)
b/Å	12.6359(13)	12.5385(10)	13.7960(7)	12.5342(7)
c/Å	33.672(3)	16.8109(14)	11.9261(6)	16.6509(8)
α/°	90	108.369(3)	90	90
β/°	90.761(4)	92.816(3)	90	105.347(4)
γ/°	90	118.579(3)	90	90
Volume/$Å^3$	3549.6(6)	2069.6(3)	3633.2(3)	7426.8(7)
Z	4	2	4	8
$\rho_{calc}g/cm^3$	1.434	1.403	1.591	1.750
μ/mm^{-1}	0.608	0.551	0.627	0.646
F(000)	1600.0	896.0	1776.0	3936.0
Crystal size/mm^3	0.204 × 0.201 × 0.072	0.295 × 0.18 × 0.147	0.402 × 0.337 × 0.237	0.254 × 0.229 × 0.17
Radiation	MoKα (λ = 0.71073)	MoKα (λ = 0.71073)	MoKα (λ = 0.71073)	MoKα (λ = 0.71073)
2Θ range for data collection/°	4.03 to 51.998	3.854 to 51.996	4.726 to 55.122	4.094 to 55.286
Index ranges	$-10 \leq h \leq 10, -15 \leq k \leq 15, -41 \leq l \leq 41$	$-14 \leq h \leq 14, -15 \leq k \leq 15, -20 \leq l \leq 20$	$-28 \leq h \leq 28, -17 \leq k \leq 17, -15 \leq l \leq 15$	$-47 \leq h \leq 48, -16 \leq k \leq 16, -21 \leq l \leq 21$
Reflections collected	139110	85549	207417	138189
Independent reflections	6986 [R_{int} = 0.1796, R_{sigma} = 0.0637]	8138 [R_{int} = 0.1209, R_{sigma} = 0.0602]	4207 [R_{int} = 0.0695, R_{sigma} = 0.0169]	8610 [R_{int} = 0.0978, R_{sigma} = 0.0335]
Data/restraints/parameters	6986/182/502	8138/36/611	4207/0/268	8610/0/556
Goodness-of-fit on F^2	1.184	1.109	1.083	1.041
Final R indexes [I>=2σ (I)]	R_1 = 0.1027, wR_2 = 0.2156	R_1 = 0.0856, wR_2 = 0.2131	R_1 = 0.0376, wR_2 = 0.0941	R_1 = 0.0451, wR_2 = 0.0984
Final R indexes [all data]	R_1 = 0.1323, wR_2 = 0.2309	R_1 = 0.1033, wR_2 = 0.2251	R_1 = 0.0385, wR_2 = 0.0948	R_1 = 0.0573, wR_2 = 0.1053
Largest diff. peak/hole / e $Å^{-3}$	1.02/-0.80	1.19/-0.56	0.71/-0.57	0.72/-0.62

Compound	**[Co(acac)$_2$(L7)](SbF$_6$)$_2$**	**[Co(tfac)$_2$(L7)]PF$_6$**	**[Co(hfac)$_2$(L7)]PF$_6$**
Identification code	mo_2022_jocoL4_0m	mo_jomk36_01a	mo_2022_jomk37_0ma
Empirical formula	$C_{36}H_{42}CoF_{12}N_6O_6Sb_2$	$C_{36}H_{36}CoF_{12}N_6O_6P * CH_2Cl_2$	$C_{36}H_{30}CoF_{18}N_6O_6P$
Formula weight	1185.18	1051.53	1074.56
Temperature/K	100.00	100.00	100.00
Crystal system	orthorhombic	monoclinic	monoclinic
Space group	Pbcn	C2/c	P21/n
a/Å	14.1416(13)	40.600(3)	10.2662(7)
b/Å	16.3724(12)	13.364(9)	21.7820(15)
c/Å	21.6400(17)	18.1035(2)	22.8633(16)
α/°	90	90	90
β/°	90	114.512(5)	99.698(3)
γ/°	90	90	90
Volume/Å^3	5010.4(7)	8937.3(12)	5039.6(6)
Z	4	8	4
ρ_{calc}g/cm^3	1.571	1.563	1.416
μ/mm^{-1}	1.487	0.641	0.484
F(000)	2340.0	4272.0	2160.0
Crystal size/mm^3	0.287 × 0.247 × 0.206	0.12 × 0.11 × 0.04	0.373 × 0.36 × 0.108
Radiation	MoKα (λ = 0.71073)	MoKα (λ = 0.71073)	MoKα (λ = 0.71073)
2Θ range for data collection/°	4.246 to 56.752	3.788 to 51.998	4.07 to 53
Index ranges	-18 ≤ h ≤ 18, -21 ≤ k ≤ 21, -28 ≤ l ≤ 28	-49 ≤ h ≤ 49, -16 ≤ k ≤ 16, -22 ≤ l ≤ 22	-12 ≤ h ≤ 12, -27 ≤ k ≤ 27, -28 ≤ l ≤ 28
Reflections collected	123127	68476	333450
Independent reflections	6257 [R_{int} = 0.0720, R_{sigma} = 0.0260]	8787 [Rint = 0.1746, Rsigma = 0.0976]	10434 [Rint = 0.1146, Rsigma = 0.0258]
Data/restraints/parameters	6257/0/354	8787/0/567	10434/12/628
Goodness-of-fit on F^2	1.100	1.015	1.101
Final R indexes [I>=2σ (I)]	R_1 = 0.0294, wR_2 = 0.0700	R1 = 0.0587, wR2 = 0.1300	R1 = 0.0814, wR2 = 0.1804
Final R indexes [all data]	R_1 = 0.0334, wR_2 = 0.0725	R1 = 0.1255, wR2 = 0.1632	R1 = 0.0886, wR2 = 0.1849
Largest diff. peak/hole / e Å^{-3}	0.56/-0.33	0.48/-0.41	1.09/-0.98

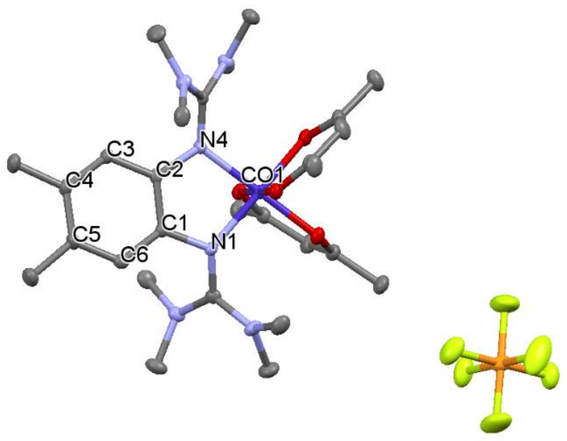

Illustration of the structure of $[Co(acac)_2(L1)]^+$ in the solid state. Color code: Co dark blue, N pale blue, F light-green, C grey, O red, P orange. Displacement ellipsoids drawn at the 50% probability level. Hydrogen atoms omitted.

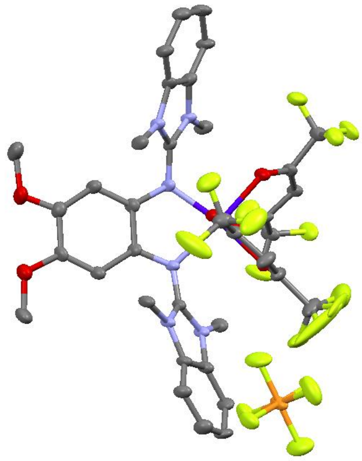

Illustration of the structure of $[Co(hfac)_2(L7)]$ in the solid state. Color code: Co dark blue, N pale blue, C grey, O red, F light-green. Displacement ellipsoids drawn at the 50% probability level. Hydrogen atoms omitted.

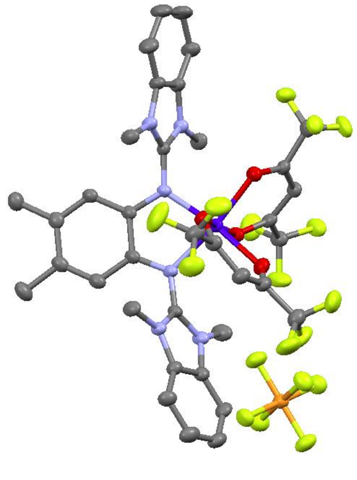

Illustration of the structure of $[Co(hfac)_2(L3)]PF_6$ in the solid state. Color code: Co dark blue, N pale blue, C grey, O red, F light-green. Displacement ellipsoids drawn at the 50% probability level. Hydrogen atoms omitted.

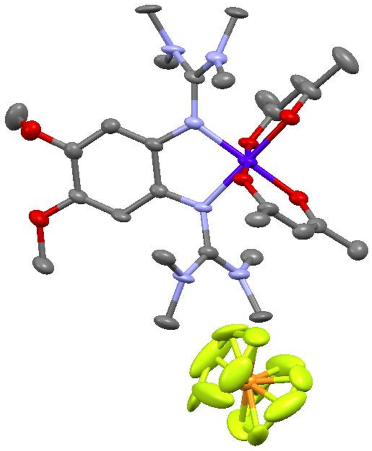

Illustration of the structure of [Co(acac)$_2$(L5)]PF_6 in the solid state. Color code: Co dark blue, N pale blue, C grey, O red, F light-green. Displacement ellipsoids drawn at the 50% probability level. Hydrogen atoms omitted.

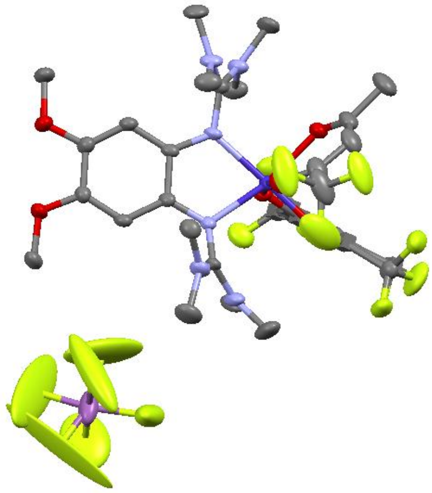

Illustration of the structure of [Co(tfac)$_2$(L5)]SbF_6 in the solid state. Color code: Co dark blue, N pale blue, C grey, O red, F light-green. Displacement ellipsoids drawn at the 50% probability level. Hydrogen atoms omitted.

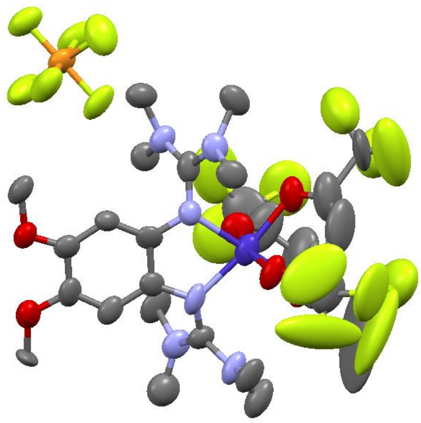

Illustration of the structure of [Co(hfac)$_2$(L5)]PF$_6$ in the solid state. Color code: Co dark blue, N pale blue, C grey, O red, F light-green, P orange. Displacement ellipsoids drawn at the 50% probability level. Hydrogen atoms omitted.

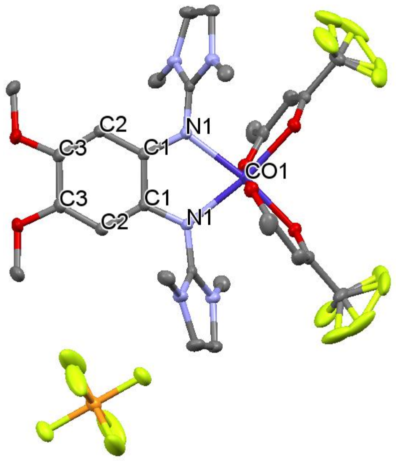

Illustration of the structure of [Co(tfac)$_2$(L6)]PF$_6$ in the solid state. Color code: Co dark blue, N pale blue, C grey, O red, F light-green, P orange. Displacement ellipsoids drawn at the 50% probability level. Hydrogen atoms omitted.

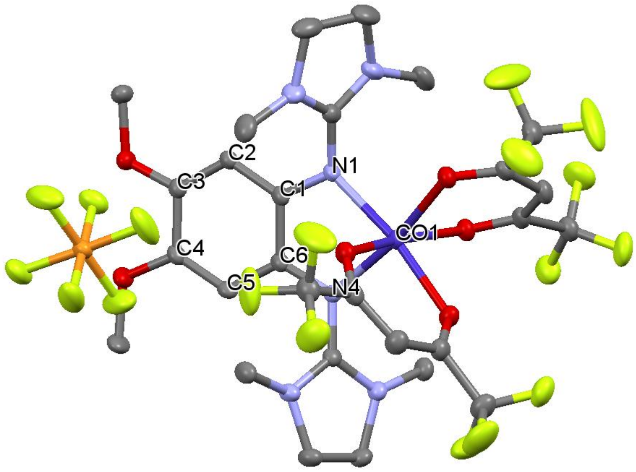

Illustration of the structure of $[Co(hfac)_2(L6)]PF_6$ in the solid state. Color code: Co dark blue, N pale blue, C grey, O red, F light-green, P orange. Displacement ellipsoids drawn at the 50% probability level. Hydrogen atoms omitted.

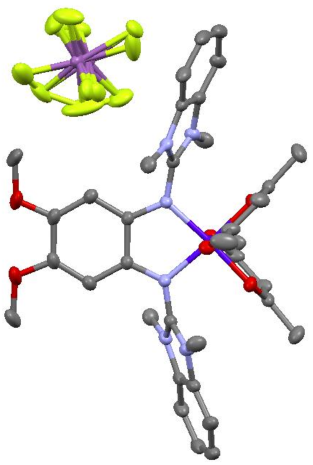

Illustration of the structure of $[Co(acac)_2(L7)](SbF_6)_2$ in the solid state. Color code: Co dark blue, N pale blue, C grey, O red, F light-green, P orange. Displacement ellipsoids drawn at the 50% probability level. Hydrogen atoms omitted.

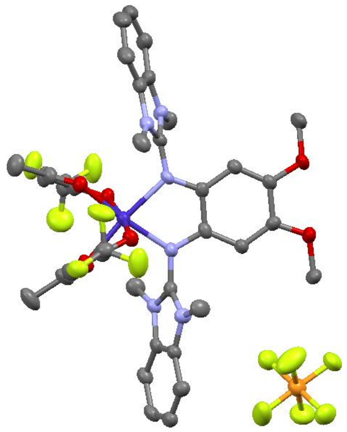

Illustration of the structure of $[Co(tfac)_2(L7)]PF_6$ in the solid state. Color code: Co dark blue, N pale blue, C grey, O red, F light-green. Displacement ellipsoids drawn at the 50% probability level. Hydrogen atoms omitted.

2.5.3 Magnetometric (SQUID, Evans NMR) measurements for the oxidized, monocationic complexes

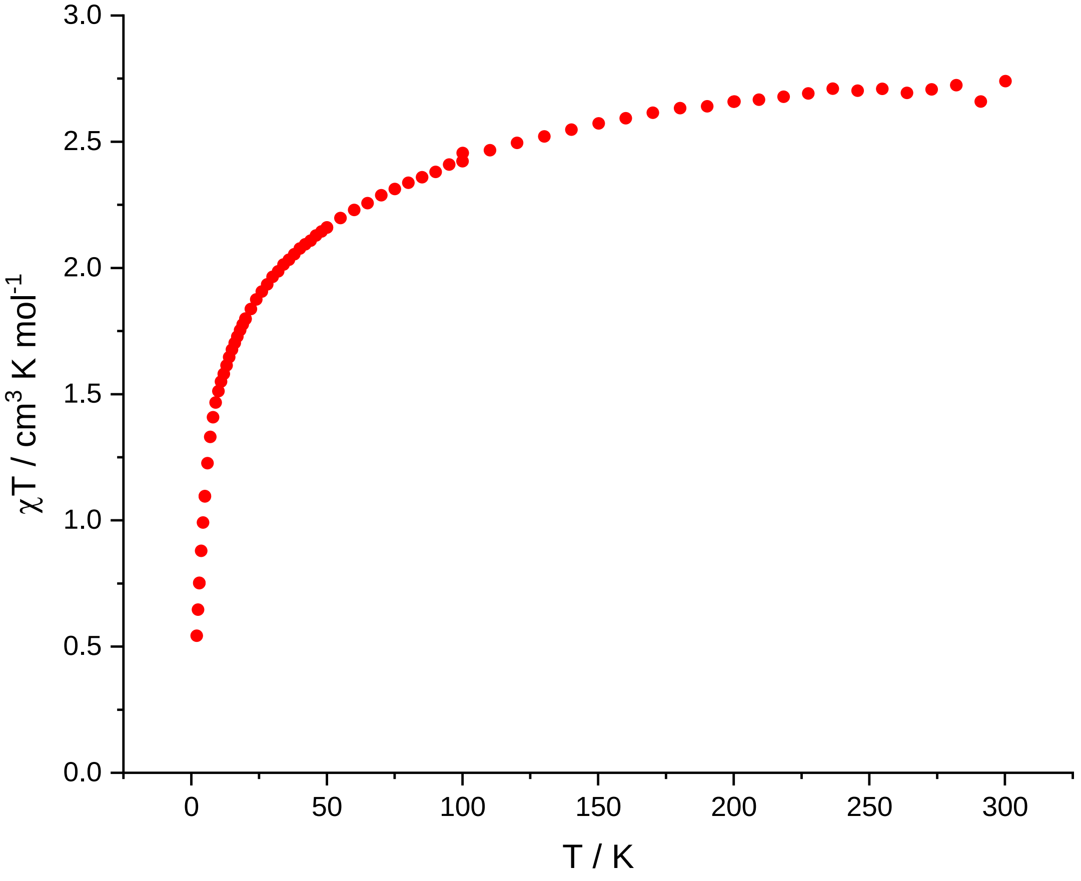

Plot of the magnetometric (SQUID) data for $[Co(tfac)_2(L6)]PF_6$ in the temperature range 2-300 K, measured at 50 mT.

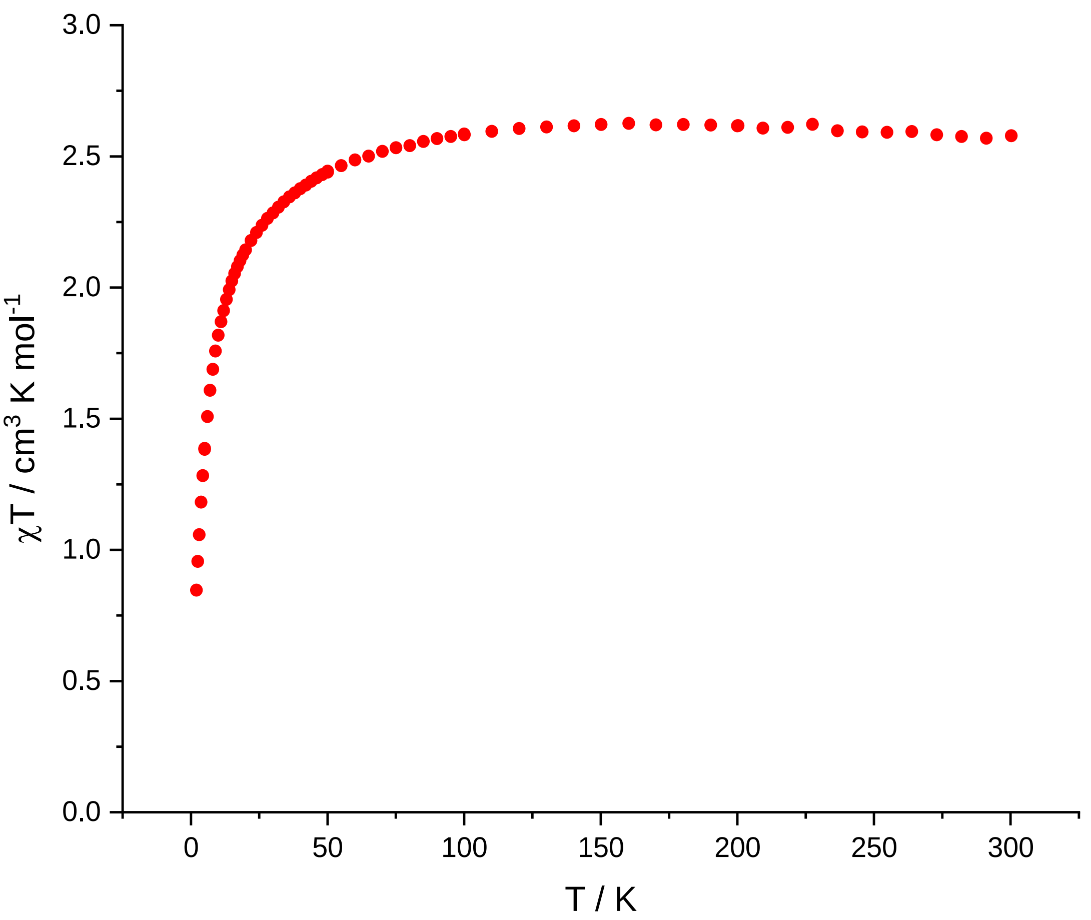

Plot of the magnetometric (SQUID) data for $[Co(tfac)_2(L7)]PF_6$ in the temperature range 2-300 K, measured at 50 mT.

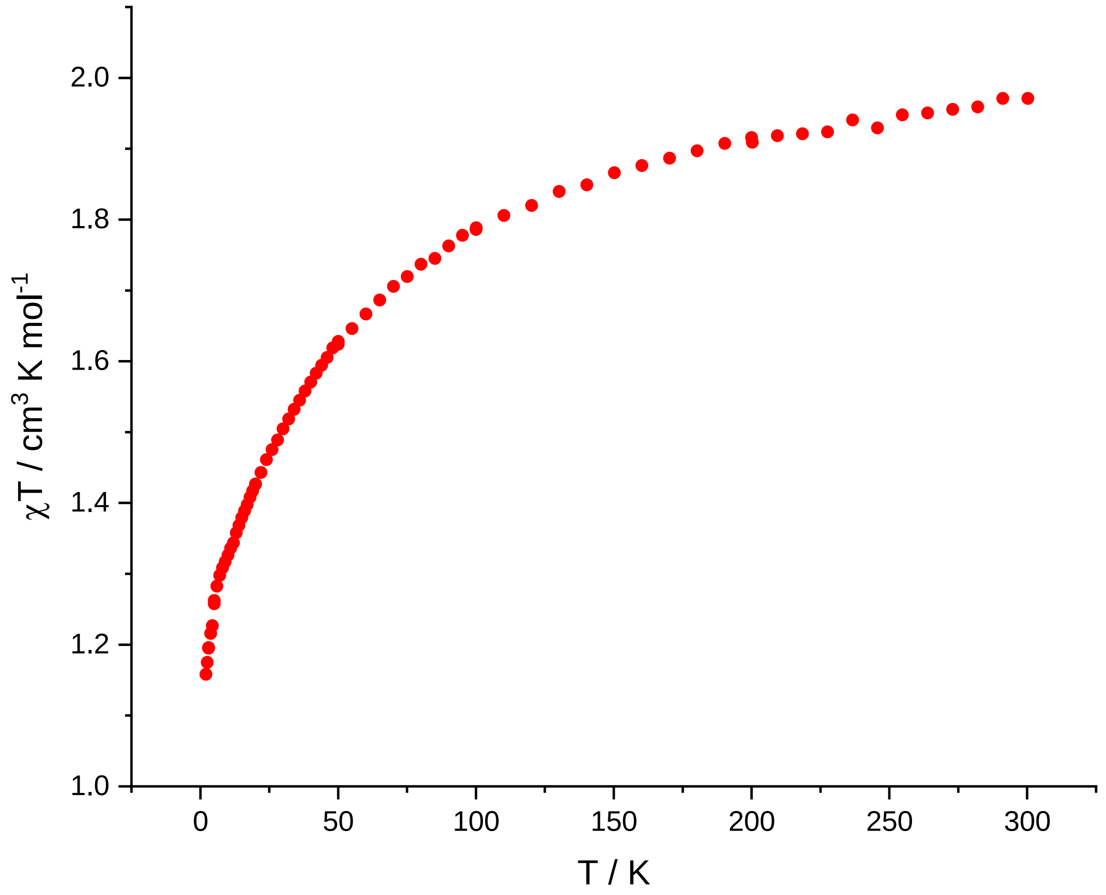

Plot of the magnetometric (SQUID) data for $[Co(acac)_2(L6)]PF_6$ in the temperature range 2-300 K, measured at 50 mT.

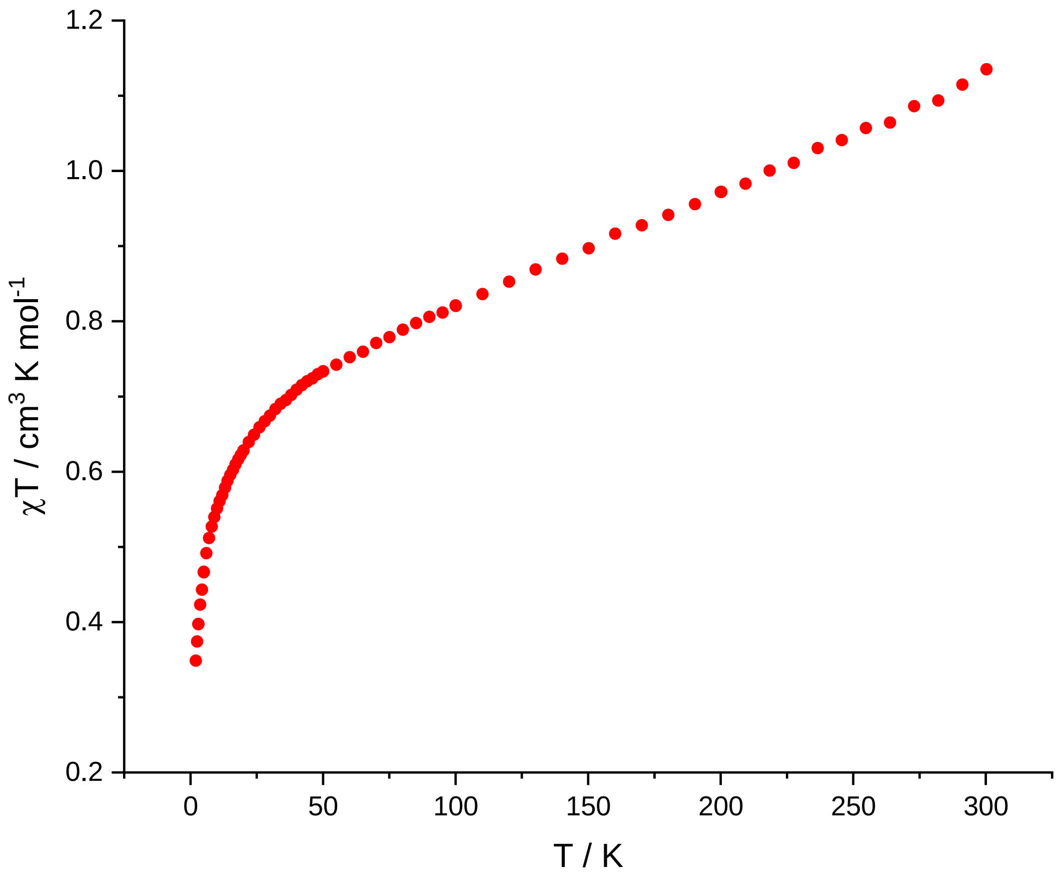

Plot of the magnetometric (SQUID) data for $[Co(acac)_2(L7)]PF_6$ in the temperature range 2-300 K, measured at 50 mT.

Results of the analysis of [Co(acac)$_2$(L7)]PF_6 in dichloromethane solution with the Evans technique

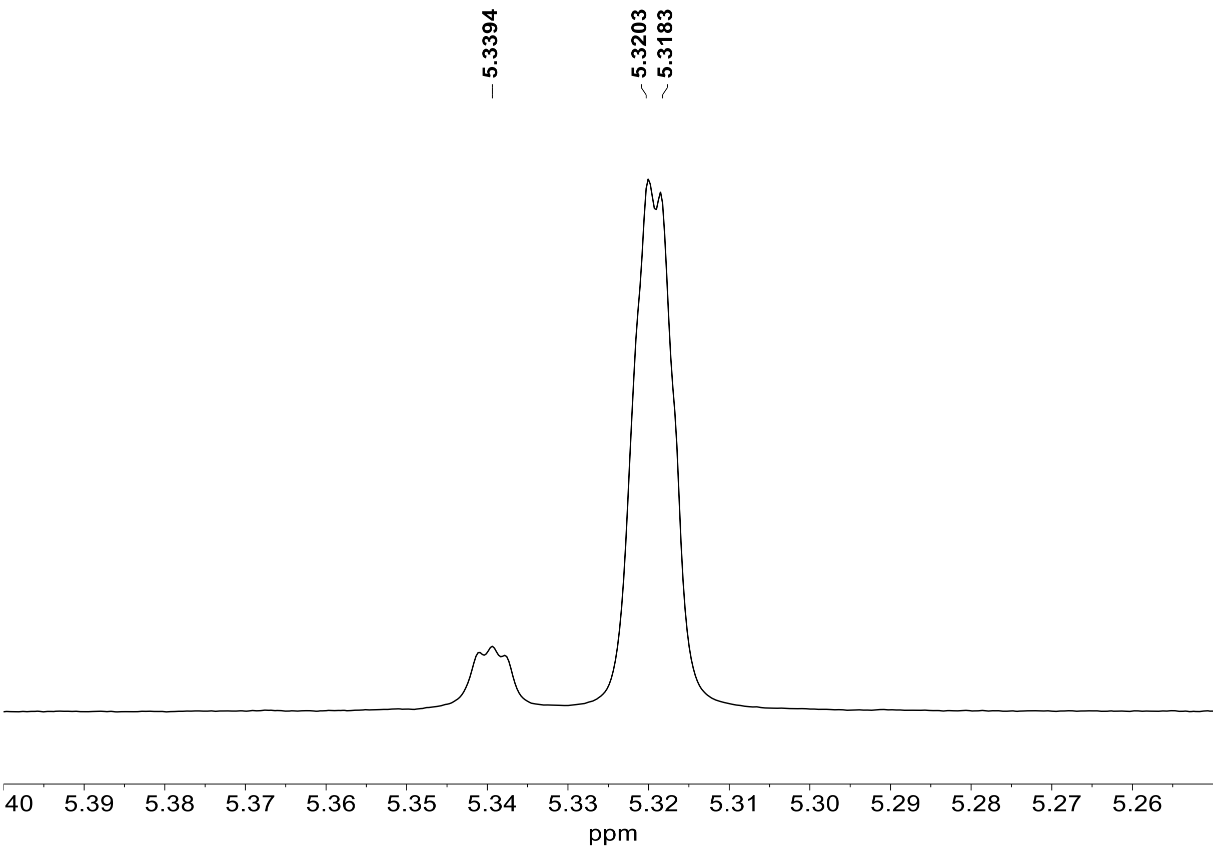

^{1}H Evans NMR of [Co(acac)$_2$(L7)]PF_6 (600 MHz, CD_2Cl_2, 298 K, $2.99 \cdot 10^{-3}$ mol L^{-1}).

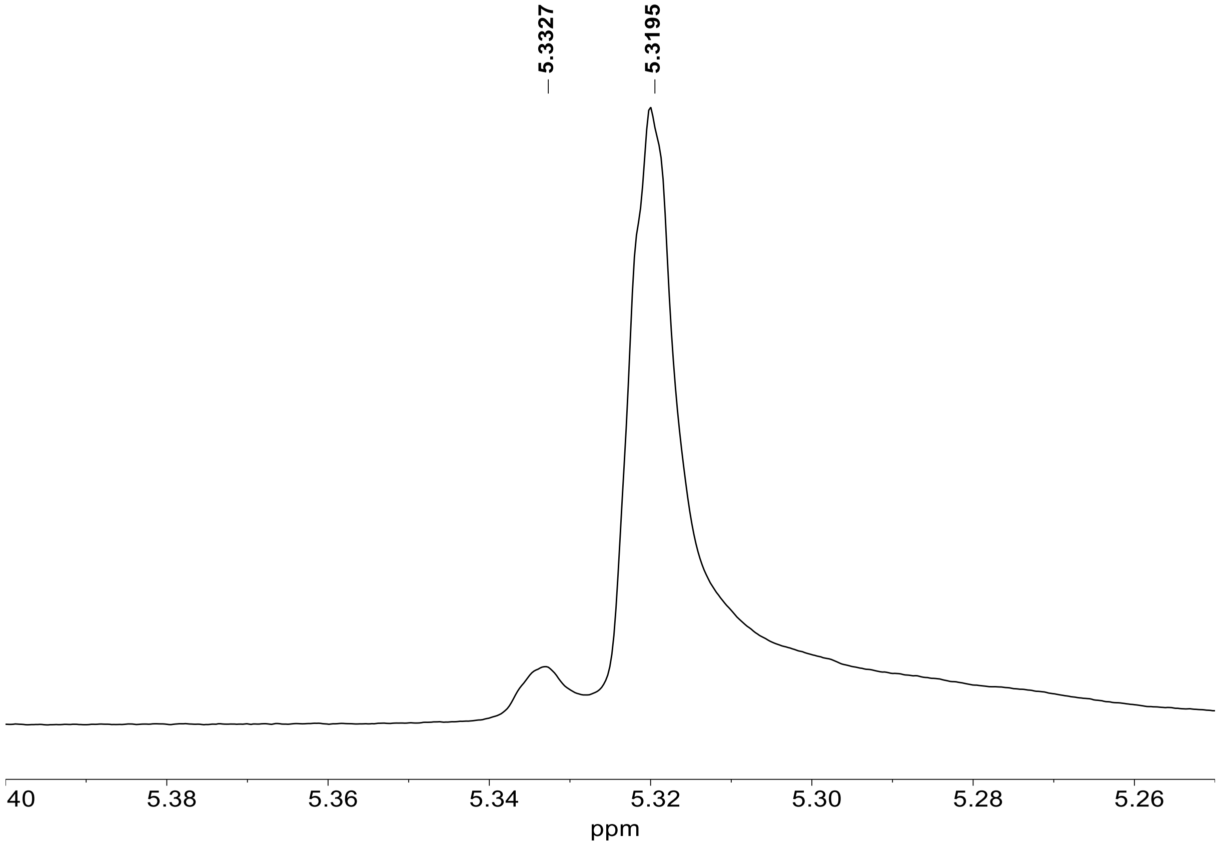

^{1}H Evans NMR of $[Co(acac)_2(L7)]PF_6$ (600 MHz, CD_2Cl_2, 234 K, $2.99{\cdot}10^{-3}$ mol L^{-1}).

RT (298 K):

$\chi_M = 1.92 \cdot 10^{-3}$

$\chi_M T = 0.572$

Low temperature (234 K)

$\chi_M = 1.39 \cdot 10^{-3}$

$\chi_M T = 0.325$

2.5.4 Summary of the electron distributions within the complexes (neutral and monocationic) in the solid state

In total, 42 different complexes (21 reduced, neutral complexes and 21 oxidized, monocationic complexes) were synthesized in this article.

The results show that all 21 reduced, neutral complexes are high-spin Co^{II} complexes with a neutral diguanidine ligand (L1-L7). 16 neutral complexes were crystallized and structurally characterized.

In the case of the monocationic complexes, the following table gives an overview of the most adequate descriptions. All complexes with hfac and tfac coligands are present as high-spin Co^{II} complexes with monocationic, radical ligand, $L^{\cdot+}$. The complexes with acac coligands are present as low-spin Co^{III} complexes with reduced, neutral diguanidine ligand, L^0, for the complexes with L1-L5. On the other hand, the complexes $[Co(acac)_2(L6)]PF_6$ and $[Co(acac)_2(L7)]PF_6$ are present as mixtures between high-spin Co^{II} complexes with monocationic, radical ligand, $L^{\cdot+}$, and low-spin Co^{III} complexes with reduced, neutral diguanidine ligand, L^0. Hence, oxidation is in these two cases both metal- and ligand-centered. The magnetometric data suggest a temperature-dependent equilibrium between both redox isomers.

Complex	Description	Applied methods to interrogate electron distribution
$[Co(acac)_2(L1)]PF_6$	low-spin Co^{III}, $L1^0$	SC-XRD, UV-vis, CV
$[Co(tfac)_2(L1)]PF_6$	high-spin Co^{II}, $L1^{\cdot+}$	UV-vis, CV
$[Co(hfac)_2(L1)]PF_6$	high-spin Co^{II}, $L1^{\cdot+}$	SC-XRD[a], UV-vis, CV
$[Co(acac)_2(L2)]PF_6$	low-spin Co^{III}, $L2^0$	UV-vis, CV
$[Co(tfac)_2(L2)]PF_6$	high-spin Co^{II}, $L2^{\cdot+}$	UV-vis, CV
$[Co(hfac)_2(L2)]PF_6$	high-spin Co^{II}, $L2^{\cdot+}$	SC-XRD[a], UV-vis, CV
$[Co(acac)_2(L3)]PF_6$	low-spin Co^{III}, $L3^0$	UV-vis, CV
$[Co(tfac)_2(L3)]PF_6$	high-spin Co^{II}, $L3^{\cdot+}$	UV-vis, CV
$[Co(hfac)_2(L3)]PF_6$	high-spin Co^{II}, $L3^{\cdot+}$	SC-XRD, UV-vis, CV
$[Co(acac)_2(L4)]PF_6$	low-spin Co^{III}, $L4^0$	UV-vis, CV
$[Co(tfac)_2(L4)]PF_6$	high-spin Co^{II}, $L4^{\cdot+}$	UV-vis, CV
$[Co(hfac)_2(L4)]PF_6$	high-spin Co^{II}, $L4^{\cdot+}$	UV-vis, CV
$[Co(acac)_2(L5)]PF_6$	low-spin Co^{III}, $L5^0$	SC-XRD, SQUID, UV-vis, CV, IR
$[Co(tfac)_2(L5)]PF_6$	high-spin Co^{II}, $L5^{\cdot+}$	SC-XRD, UV-vis, CV, IR
$[Co(hfac)_2(L5)]PF_6$	high-spin Co^{II}, $L5^{\cdot+}$	UV-vis, CV, IR
$[Co(acac)_2(L6)]PF_6$	mixture between low-spin Co^{III}, $L6^0$ and high-spin Co^{II}, $L6^{\cdot+}$	SQUID, UV-vis, CV, IR
$[Co(tfac)_2(L6)]PF_6$	high-spin Co^{II}, $L6^{\cdot+}$	SC-XRD, SQUID, UV-vis, CV, IR
$[Co(hfac)_2(L6)]PF_6$	high-spin Co^{II}, $L6^{\cdot+}$	SC-XRD, UV-vis, CV, IR
$[Co(acac)_2(L7)]PF_6$	mixture between low-spin Co^{III}, $L7^0$ and high-spin Co^{II}, $L7^{\cdot+}$	SQUID, Evans-NMR,[b] UV-vis, CV, IR, DFT calculations
$[Co(tfac)_2(L7)]PF_6$	high-spin Co^{II}, $L7^{\cdot+}$	SC-XRD, SQUID, UV-vis, CV, IR
$[Co(hfac)_2(L7)]PF_6$	high-spin Co^{II}, $L7^{\cdot+}$	SC-XRD, UV-vis, CV, IR

[a] Crystallized with $[Co(hfac)_3]^-$ counterions. [b] Information about complex in CH_2Cl_2 solution.

[1] SAINT (APEX III/IV) Bruker AXS GmbH, Karlsruhe, Germany 2016/2019.
[2] a) G. M. Sheldrick, SHELXT, Program for Crystal Structure Solution, University of Göttingen, Germany 2014-2018; b) G. M. Sheldrick, *Acta Cryst.*, **2015**, *A71*, 3–8.
[3] a) G. M. Sheldrick, SHELXL-20xx, University of Göttingen and Bruker AXS GmbH, Karlsruhe, Germany 2012-2018; b) W. Robinson, G. M. Sheldrick in: N. W. Isaaks, M. R. Taylor (eds.) ”Crystallographic Computing 4“, Ch. 22, IUCr and Oxford University Press, Oxford, UK, 1988; c) G. M. Sheldrick, *Acta Cryst.* **2008**, *A64*, 112–122; (d) G. M. Sheldrick, *Acta Cryst.* **2015**, *C71*, 3–8.
[4] O. V. Dolomanov, L. J. Bourhis, R. J. Gildea, J. A. K. Howard, H. Puschmann, OLEX2: A complete structure solution, refinement and analysis program, *J. Appl. Cryst.* **2009**, *42*, 339–341.
[5] (a) Rollett, J. S., in: Ahmed, F. R.; Hall, S. R.; Huber, C. P. (eds.), Crystallographic Computing. Munksgaard, Copenhagen, Denmark, 1970, p. 167; (b) Watkin, C., in: Isaaks, N. W.; Taylor, M. R. (eds.), Crystallographic Computing 4. Ch.

3. Quantum-chemical calculations on the cobalt complexes

3.1 Details of quantum-chemical calculations

The density functional calculations are performed with the program TURBOMOLE.[1,2,3,4] Three different functionals, BLYP,[5,6] TPSSh,[7,8] and B3LYP,[9,10] are used in connection with the def2-SV(P) and def2-TZVP basis sets.[11] The BLYP functional is used as representative of a gradient corrected functional. The TPSSh and B3LYP functionals are hybrid functionals with different amount of HF exchange, 10 and 20 %, respectively. For the calculation of the two-electron integrals, the resolution-of-the-identity (RI) approximation[12] is used with the appropriate def2-SV(P) and def2-TZVP auxiliary basis set.[13] The calculations include the D3 dispersion correction with Becke-Johnson damping.[14,15] A part of the calculations accounts for the influence of a polarizable environment by the conductor-like screening model (COSMO).[16] Structure optimizations are performed with the def2-SV(P) and def2-TZVP basis sets, the determination of the harmonic vibrational frequencies with the def2-SV(P) basis set only. The electronic excitation energies are obtained by time-dependent density functional calculations.[17,18]

[1] TURBOMOLE V7.7 2022, a development of University of Karlsruhe and Forschungszentrum Karlsruhe GmbH, 1989-2007, TURBOMOLE GmbH, since 2007; available from https://www.turbomole.org.

[2] S. G. Balasubramani, G. P. Chen, S. Coriani, M. Diedenhofen, M. S. Frank, Y. J. Franzke, F. Furche, R. Grotjahn, M. E. Harding, C. Hättig, A. Hellweg, B. Helmich-Paris, C. Holzer, U. Huniar, M. Kaupp, A. Marefat Khah, S. Karbalaei Khani, T. Müller, F. Mack, B. D. Nguyen, S. M. Parker, E. Perlt, D. Rappoport, K. Reiter, S. Roy, M. Rückert, G. Schmitz, M. Sierka, E. Tapavicza, D. P. Tew, C. van Wüllen, V. K. Voora, F. Weigend, A. Wodyński, J. M. Yu, *J. Chem. Phys.* **2020**, *152*, 184107.

[3] R. Ahlrichs, M. Bär, M. Häser, H. Horn, C. Kölmel, *Chem. Phys. Lett.* **1989**, *162*, 165-169.

[4] O. Treutler, R. Ahlrichs, *J. Chem. Phys.* **1995**, *102*, 346-354.

[5] A. D. Becke, *Phys. Rev. A* **1988,** *38,* 3098-3100.

[6] C. Lee, W. Yang, R. G. Parr, *Phys. Rev. B* **1988,** *37,* 785-789.

[7] J. Tao, J. P. Perdew, V. N. Staroverov, G. E. Scuseria, *Phys. Rev. Lett.* **2003,** *91,* 146401.

[8] V. N. Staroverov, G. E. Scuseria, J. Tao, J. P. Perdew, *J. Chem. Phys.* **2003,** *119,* 12129-12137.

[9] A. D. Becke, *J. Chem. Phys.* **1993**, *98*, 5648-5652.

[10] P. J. Stephens, F. J. Devlin, C. F. Chabalowski, M. J. Frisch, *J. Phys. Chem.* **1994**, *98*, 11623-11627.

[11] F. Weigend, R. Ahlrichs, *Phys. Chem. Chem. Phys.* **2005**, *7*, 3297-3305.

[12] K. Eichkorn, O. Treutler, H. Öhm, M. Häser, R. Ahlrichs, *Chem. Phys. Lett.* **1995**, *242*, 652-660.

[13] F. Weigend, *Phys. Chem. Chem. Phys.* **2006**, *8*, 1057-1065.

[14] S. Grimme, J. Antony, S. Ehrlich, H. Krieg, *J. Chem. Phys.* **2010**, *132*, 154104.

[15] S. Grimme, S. Ehrlich, L. Goerigk, *J. Comput. Chem.* **2011**, *32*, 1456-1465.

[16] A. Klamt, G. Schüürmann, *J. Chem. Soc. Perkin Trans. 2* **1993**, 799-805.

[17] R. Bauernschmitt, R. Ahlrichs, *Chem. Phys. Lett.* **1996,** *256,* 454-464.

[18] R. Bauernschmitt, M. Häser, O. Treuler, R. Ahlrichs, *Chem. Phys. Lett.* **1997,** *264,* 573-578.

3.2 Total energies and optimized coordinates

3.2.1 Total energies

Energies by density functional calculations with different functionals using the def2-SV(P) and def2-TZVP basis sets with inclusion of the D3 dispersion correction with Becke-Johnson damping and without and with inclusion of a dielectric environment.

Complex	Functional	ε_r	Term	$E^{SV(P)}$/Hartree	E^{TZVP}/Hartree
$Co(acac)_2(L7)^+$	B3LYP	1	^5A	-3556.750782	-3559.415682
		1	$^{(3)}A$	-3556.748188	-3559.413014
		1	$^{(1)}A$	-3556.732273	-3559.399141
		1	^1A	-3556.747459	-3559.413105
	TPSSh	1	^5A	-3558.244936	-3560.839381
		1	$^{(3)}A$	-3558.239870	-3560.834250
		1	^1A	-3558.255838	-3560.852229
	BLYP	1	^5A	-3557.399570	-3560.095450
		1	^3A	-3557.402657	-3560.100241
		1	^1A	-3557.418850	-3560.115340
$Co(acac)_2(L7)(PF_6)$	B3LYP	1	^5A	-4496.722688	-4500.287530
		1	$^{(3)}A$	-4496.721009	-4500.285735
		1	$^{(1)}A$	-4496.704200	-4500.270864
		1	^1A	-4496.714545	-4500.279701
		37.5	^5A	-4496.771214	-4500.342266
		37.5	$^{(3)}A$	-4496.770034	-4500.341326
		37.5	$^{(1)}A$	-4496.751939	-4500.325015
		37.5	^1A	-4496.763327	-4500.333329
	TPSSh	1	^5A	-4498.456515	-4501.943772
		1	$^{(3)}A$	-4498.453308	-4501.940272
		1	^1A	-4498.463273	-4501.952133
	BLYP	1	^5A	-4497.567518	-4501.170675
		1	^3A	-4497.571105	-4501.175698
		1	^1A	-4497.585279	-4501.188257

3.2.2 Optimized coordinates by B3LYP calculations

Calculations without counterion and without solvent effect

$[Co(acac)_2(L7)]^+$, ^5A, $\varepsilon_r = 1$

Energy = -3559.415681988

C	6.7276545	7.1000667	6.7734387
C	7.4139203	7.1001579	4.0465226
C	6.8957423	8.2945571	6.1088510
C	7.2455048	8.2946048	4.7111138
H	6.4285167	7.0938050	7.8105792
H	7.7130772	7.0939696	3.0093882
O	6.7508740	9.5093602	6.6523523
O	7.3900134	9.5094476	4.1676045

C	6.4010795	9.6076860	8.0293706
H	5.4298974	9.1440121	8.2190857
H	7.1633146	9.1416810	8.6590597
C	6.8806059	5.8675409	6.1059209
C	7.2612668	5.8675896	4.7140172
N	6.6284935	4.6616125	6.6525475
N	7.5137669	4.6617280	4.1673791
Co	7.0710872	2.9395903	5.4100958
C	6.5513478	4.4364352	7.9645708
C	7.5905095	4.4364376	2.8553908
O	5.1057823	2.9617296	4.7709502
O	9.0364304	2.9614182	6.0489504
O	7.4651536	1.6065943	3.9075839
O	6.6768803	1.6071170	6.9130643
N	5.5282243	3.7922300	8.5701267
N	7.5291523	4.6563856	8.8875192
N	8.6132250	3.7916690	2.2497012
N	6.6127217	4.6569285	1.9325148
C	7.7398017	9.6078695	2.7905904
H	7.7925459	10.6708379	2.5750447
H	8.7111252	9.1444876	2.6008897
H	6.9777144	9.1416305	2.1608954
H	6.3480211	10.6706398	8.2449114
C	8.2858010	3.5644401	0.9189100
C	9.8261733	3.3357186	2.9100829
C	7.0158423	4.1197732	0.7122876
C	5.2870197	5.1687707	2.2352039
C	8.9738199	2.9253109	-0.1010710
H	9.8785924	2.2505638	2.8456791
H	10.6946414	3.7887709	2.4316428
H	9.7768875	3.6099862	3.9597711
C	6.3886443	4.0546487	-0.5223009
H	5.2139733	6.2319281	2.0047706
H	4.5597679	4.6161422	1.6437971
H	5.0804613	4.9996092	3.2885238
C	8.3444883	2.8571481	-1.3389086
H	9.9517087	2.4933287	0.0585038
H	5.4098184	4.4822702	-0.6881886
C	7.0762041	3.4106874	-1.5451634
H	8.8464366	2.3645978	-2.1603565
H	6.6195280	3.3368598	-2.5225773
C	6.6280279	0.9748418	3.2003686
C	7.5139916	0.9752230	7.6201899
C	5.2367666	1.1716029	3.2162450
C	7.2070476	-0.0406255	2.2461376
C	8.9053081	1.1714861	7.6038614
C	6.9348728	-0.0398308	8.5747986
C	4.5613647	2.1263582	3.9850914
H	4.6414036	0.5429745	2.5712460
H	7.9111832	-0.6791007	2.7813150
H	6.4436787	-0.6544027	1.7721417

H	7.7660767	0.4829543	1.4659900
C	9.5808120	2.1259185	6.8346808
H	9.5006508	0.5427452	8.2487698
H	6.2304479	-0.6782384	8.0399183
H	7.6981613	-0.6537143	9.0487873
H	6.3761436	0.4840965	9.3549262
C	11.0849202	2.2088744	6.9350690
H	11.3842178	3.2403148	7.1317970
H	11.4933501	1.5582564	7.7058188
H	11.5197320	1.9277604	5.9726614
C	3.0572841	2.2095923	3.8844965
H	2.7581869	3.2410681	3.6876532
H	2.6488337	1.5589882	3.1137456
H	2.6222946	1.9286300	4.8468687
C	5.8554084	3.5647756	9.9009454
C	4.3152642	3.3366475	7.9095065
C	7.1256505	4.1194117	10.1076956
C	5.1669834	2.9259169	10.9208231
H	4.2624617	2.2515157	7.9740203
H	3.4468376	3.7900382	8.3876978
H	4.3648949	3.6108037	6.8598039
C	5.7962017	2.8573102	12.1586901
H	4.1888846	2.4944490	10.7611450
C	7.0648029	3.4100920	12.3650564
H	5.2939021	2.3650347	12.9800876
C	7.7527823	4.0537558	11.3422944
H	7.5214102	3.3358521	13.3424713
H	8.7319186	4.4806547	11.5082122
C	8.8550987	5.1676651	8.5849498
H	8.9286845	6.2306918	8.8158151
H	9.5821111	4.6144204	9.1760769
H	9.0614797	4.9988196	7.5315459

[Co(acac)$_2$(L7)]$^+$, $^{(3)}$A, ε_r = 1

Energy = -3559.413013679

C	-0.2535939	3.0219060	1.3783687
C	0.2651857	3.0257556	-1.3846088
C	-0.1294545	4.2167058	0.7067991
C	0.1379341	4.2186917	-0.7110278
H	-0.4902357	3.0160340	2.4313104
H	0.4972275	3.0212504	-2.4387012
O	-0.2462519	5.4298352	1.2572933
O	0.2498712	5.4333043	-1.2590585
C	-0.5157268	5.5287701	2.6529457
H	-1.4724054	5.0616750	2.8987118
H	0.2839057	5.0661918	3.2368667
C	-0.1378415	1.7864267	0.7062335
C	0.1545286	1.7885447	-0.7137034
N	-0.3530381	0.5874341	1.2775442

N	0.3595799	0.5924644	-1.2904826
Co	0.0239053	-1.1661695	-0.0157064
C	-0.3737924	0.3790352	2.5926493
C	0.3750409	0.3900898	-2.6084892
O	-1.9992851	-1.1142982	-0.5242770
O	2.0107868	-1.0893605	0.5149466
O	0.3141096	-2.4500862	-1.5866463
O	-0.3045794	-2.5234987	1.4785656
N	-1.3561674	-0.2867553	3.2431319
N	0.6219293	0.6532980	3.4835933
N	1.3759282	-0.2309451	-3.2722266
N	-0.6446718	0.6192354	-3.4817474
C	0.5163059	5.5356818	-2.6551243
H	0.5568528	6.5992607	-2.8698915
H	1.4739335	5.0719178	-2.9035148
H	-0.2830413	5.0716939	-3.2382724
H	-0.5598792	6.5918130	2.8696542
C	0.9925758	-0.4311682	-4.5927830
C	2.6200355	-0.6918779	-2.6749785
C	-0.2923977	0.1104463	-4.7291097
C	-1.9592447	1.1156379	-3.1159688
C	1.6417700	-1.0373663	-5.6573484
H	2.6856882	-1.7728402	-2.7825601
H	3.4633342	-0.2085204	-3.1686090
H	2.6088516	-0.4581122	-1.6144359
C	-0.9765583	0.0604725	-5.9336817
H	-2.0743987	2.1625347	-3.3982381
H	-2.7114892	0.5142024	-3.6228486
H	-2.0884125	1.0033289	-2.0435502
C	0.9562903	-1.0890078	-6.8657629
H	2.6317914	-1.4583316	-5.5526134
H	-1.9687623	0.4751336	-6.0435424
C	-0.3284554	-0.5515145	-7.0009009
H	1.4263800	-1.5564288	-7.7200766
H	-0.8293838	-0.6128136	-7.9572760
C	-0.5587346	-2.9547281	-2.3481932
C	0.5449739	-2.9548126	2.3075524
C	-1.9363631	-2.6732726	-2.3161626
C	-0.0455052	-3.9277755	-3.3821434
C	1.9043573	-2.5897391	2.3566143
C	0.0286421	-3.9415254	3.3271665
C	-2.5734882	-1.8164980	-1.4097541
H	-2.5623910	-3.2018251	-3.0193555
H	0.5984280	-4.6617817	-2.8958921
H	-0.8467162	-4.4385218	-3.9124328
H	0.5644368	-3.3882386	-4.1109360
C	2.5586048	-1.7380625	1.4590895
H	2.5112548	-3.0659929	3.1121412
H	-0.5326617	-4.7245990	2.8159346
H	0.8238729	-4.3889229	3.9199559
H	-0.6620754	-3.4316599	4.0031523

C	4.0535116	-1.5642502	1.5863782
H	4.3091789	-0.5043407	1.5359201
H	4.4515051	-1.9949826	2.5033781
H	4.5379942	-2.0509705	0.7361835
C	-4.0786254	-1.6989109	-1.4625178
H	-4.3668240	-0.6459544	-1.4558760
H	-4.5107985	-2.1916235	-2.3314955
H	-4.4991277	-2.1515784	-0.5610259
C	-0.9852137	-0.4674256	4.5700392
C	-2.5868460	-0.7729592	2.6378698
C	0.2725160	0.1297063	4.7255328
C	-1.6258776	-1.0964909	5.6263134
H	-2.6298243	-1.8557637	2.7383418
H	-3.4423321	-0.3117293	3.1320001
H	-2.5773935	-0.5338467	1.5784634
C	-0.9587806	-1.1155781	6.8461287
H	-2.5960921	-1.5579095	5.5074436
C	0.2999114	-0.5247089	6.9998867
H	-1.4231669	-1.5991434	7.6945521
C	0.9387429	0.1113435	5.9408578
H	0.7874887	-0.5620574	7.9643616
H	1.9102551	0.5686651	6.0656295
C	1.9267381	1.1870754	3.1374876
H	2.0077394	2.2376045	3.4182216
H	2.6884145	0.6093880	3.6577186
H	2.0764935	1.0792922	2.0674073

[Co(acac)$_2$(L7)]$^+$, $^{(1)}$A, ε_r = 1

Energy = -3559.399141136

C	-0.2298001	3.1213354	1.3848960
C	0.2915925	3.0338583	-1.3763661
C	-0.0537421	4.2927721	0.6839836
C	0.2134836	4.2476028	-0.7321719
H	-0.4683417	3.1493323	2.4370974
H	0.5240304	2.9946726	-2.4297589
O	-0.1185186	5.5241636	1.2044228
O	0.3754723	5.4429075	-1.3116538
C	-0.3859869	5.6677515	2.5960473
H	-1.3619262	5.2475178	2.8507864
H	0.3921431	5.1859927	3.1935750
C	-0.1699611	1.8660000	0.7424721
C	0.1241701	1.8208648	-0.6744724
N	-0.4460641	0.6915215	1.3314302
N	0.2756656	0.6017015	-1.2244189
Co	-0.0093055	-1.0626128	-0.0338118
C	-0.4270368	0.4446112	2.6289481
C	0.3061257	0.3918407	-2.5472494
O	-1.8712733	-1.0799502	-0.5467396
O	1.8326476	-0.9342397	0.5007531

O	0.4706675	-2.4603580	-1.5492611
O	-0.4219306	-2.4543267	1.2805699
N	-1.3778492	-0.2854590	3.2675857
N	0.5762118	0.7074948	3.5218203
N	1.3246563	-0.1954693	-3.2116621
N	-0.7180902	0.5952202	-3.4186217
C	0.6437369	5.4976684	-2.7093833
H	0.7275950	6.5526852	-2.9522999
H	1.5813227	4.9888823	-2.9465028
H	-0.1745329	5.0517359	-3.2804511
H	-0.3855056	6.7365942	2.7875596
C	0.9447965	-0.4102966	-4.5306727
C	2.5913008	-0.6008967	-2.6224351
C	-0.3534633	0.0989914	-4.6666726
C	-2.0334761	1.0937431	-3.0586609
C	1.6082296	-1.0021289	-5.5949341
H	2.6778862	-1.6840213	-2.6770175
H	3.4095809	-0.1204866	-3.1588411
H	2.5936291	-0.3151927	-1.5751826
C	-1.0358340	0.0343824	-5.8720637
H	-2.1519157	2.1333540	-3.3653799
H	-2.7859902	0.4792356	-3.5493275
H	-2.1551128	1.0040048	-1.9835155
C	0.9243803	-1.0693473	-6.8030763
H	2.6074771	-1.4004663	-5.4897032
H	-2.0377090	0.4250430	-5.9819213
C	-0.3727762	-0.5613474	-6.9387324
H	1.4049472	-1.5261451	-7.6573161
H	-0.8715822	-0.6344871	-7.8953876
C	-0.3936167	-3.0456790	-2.2469628
C	0.4072600	-2.9667143	2.0823846
C	-1.7864442	-2.8097263	-2.1879329
C	0.1164407	-4.0550246	-3.2502031
C	1.7689987	-2.6242734	2.1686269
C	-0.1436926	-4.0001243	3.0321483
C	-2.4263596	-1.8912671	-1.3597307
H	-2.4146704	-3.4096079	-2.8293713
H	0.9023126	-4.6558578	-2.7923867
H	-0.6702148	-4.7043880	-3.6302768
H	0.5576360	-3.5211839	-4.0967458
C	2.3995762	-1.6698518	1.3727081
H	2.3756977	-3.1620793	2.8814654
H	-0.8965988	-4.6008843	2.5232323
H	0.6347299	-4.6451872	3.4357134
H	-0.6278544	-3.4921298	3.8711205
C	3.8861088	-1.4606213	1.5112239
H	4.1154410	-0.3942154	1.5271211
H	4.2914994	-1.9373127	2.4015776
H	4.3851393	-1.8845337	0.6359813
C	-3.9330292	-1.8079562	-1.3912603
H	-4.2482571	-0.7653717	-1.4612631

H	-4.3659582	-2.3765697	-2.2118043
H	-4.3278541	-2.1996633	-0.4504069
C	-0.9810517	-0.5121618	4.5786938
C	-2.6102425	-0.7612781	2.6602789
C	0.2615398	0.1151706	4.7426000
C	-1.5855361	-1.2055269	5.6154109
H	-2.6736140	-1.8412734	2.7774959
H	-3.4662480	-0.2812008	3.1362746
H	-2.5828124	-0.5368226	1.5980474
C	-0.8983120	-1.2603623	6.8240222
H	-2.5455308	-1.6867110	5.4914761
C	0.3444008	-0.6407809	6.9853536
H	-1.3359059	-1.7938984	7.6566103
C	0.9468477	0.0612218	5.9455498
H	0.8485427	-0.7055313	7.9398065
H	1.9056032	0.5429417	6.0775564
C	1.8664398	1.2773924	3.1807357
H	1.9292496	2.3232005	3.4834330
H	2.6443234	0.7063379	3.6844828
H	2.0128891	1.1978278	2.1077202

[Co(acac)$_2$(L7)]$^+$, ^{1}A, ε_r = 1

Energy = -3559.413105168

C	-0.4721501	2.9330299	1.3051994
C	0.4722870	2.9330470	-1.3051697
C	-0.2460194	4.1413826	0.6626533
C	0.2461131	4.1413907	-0.6626214
H	-0.8637502	2.9232211	2.3115701
H	0.8639417	2.9232588	-2.3115191
O	-0.4607313	5.3611003	1.2150114
O	0.4607451	5.3611178	-1.2149912
C	-0.9763629	5.4195766	2.5322210
H	-1.9569540	4.9378706	2.5991767
H	-0.2941725	4.9518235	3.2499327
C	-0.2210350	1.7160574	0.6584699
C	0.2211436	1.7160658	-0.6584673
N	-0.4670591	0.4558937	1.2312057
N	0.4671460	0.4559054	-1.2312250
Co	0.0000489	-1.0135626	-0.0000163
C	-0.4425860	0.2773160	2.5427823
C	0.4426311	0.2773429	-2.5428017
O	-1.8019035	-0.9607828	-0.6241835
O	1.8020031	-0.9608013	0.6241532
O	0.5487196	-2.3894132	-1.2003772
O	-0.5486437	-2.3893784	1.2003770
N	-1.3746478	-0.4374262	3.2351533
N	0.4913898	0.7181923	3.4413333
N	1.3746317	-0.4374370	-3.2352165
N	-0.4913492	0.7182908	-3.4413167

C	0.9763568	5.4196180	-2.5322074
H	1.0761278	6.4757846	-2.7683861
H	1.9569860	4.9379902	-2.5991680
H	0.2941965	4.9518016	-3.2499065
H	-1.0762231	6.4757381	2.7683848
C	1.0259123	-0.4767655	-4.5785327
C	2.6345965	-0.9027085	-2.6818349
C	-0.1503591	0.2741312	-4.7142246
C	-1.7365445	1.3798287	-3.0953877
C	1.6314065	-1.0797851	-5.6699516
H	2.6871737	-1.9886503	-2.7117446
H	3.4564134	-0.4753754	-3.2577160
H	2.6857191	-0.5822592	-1.6457413
C	-0.7624572	0.4425121	-5.9464832
H	-1.6768127	2.4510995	-3.2876413
H	-2.5389501	0.9457173	-3.6901455
H	-1.9389447	1.2170493	-2.0418711
C	1.0172351	-0.9114788	-6.9073265
H	2.5420855	-1.6535965	-5.5681037
H	-1.6670080	1.0239556	-6.0580923
C	-0.1588310	-0.1678975	-7.0418955
H	1.4606735	-1.3643104	-7.7835002
H	-0.6086265	-0.0609988	-8.0194766
C	-0.2228949	-2.9999506	-2.0039506
C	0.2229509	-2.9998938	2.0039839
C	-1.5837448	-2.7394744	-2.1790865
C	0.4402794	-4.0550923	-2.8465608
C	1.5838181	-2.7394736	2.1790776
C	-0.4402641	-4.0549404	2.8466808
C	-2.2921671	-1.7589637	-1.4832325
H	-2.1236781	-3.3423037	-2.8925903
H	1.2136184	-4.5598858	-2.2692707
H	-0.2764059	-4.7815809	-3.2241126
H	0.9168986	-3.5753323	-3.7060569
C	2.2922596	-1.7589983	1.4831941
H	2.1237414	-3.3423047	2.8925867
H	-1.2135633	-4.5598129	2.2694048
H	0.2764006	-4.7813760	3.2243725
H	-0.9169473	-3.5750758	3.7060820
C	3.7691356	-1.5998398	1.7217023
H	4.0289483	-0.5422752	1.7638603
H	4.0945682	-2.0997125	2.6315344
H	4.3092528	-2.0334038	0.8760801
C	-3.7690435	-1.5997982	-1.7217414
H	-4.0289157	-0.5422361	-1.7635340
H	-4.0944115	-2.0993794	-2.6317581
H	-4.3091681	-2.0337071	-0.8762995
C	-1.0259941	-0.4767674	4.5784851
C	-2.6346321	-0.9025821	2.6817184
C	0.1503328	0.2740363	4.7142249
C	-1.6315803	-1.0797436	5.6698769

H	-2.6873253	-1.9885178	2.7116598
H	-3.4564370	-0.4751464	3.2575408
H	-2.6856638	-0.5821561	1.6456136
C	-1.0174724	-0.9114506	6.9072842
H	-2.5422707	-1.6535282	5.5679789
C	0.1586187	-0.1679189	7.0419111
H	-1.4609795	-1.3642547	7.7834372
C	0.7623470	0.4424338	5.9465231
H	0.6083583	-0.0610183	8.0195177
H	1.6668946	1.0238711	6.0581903
C	1.7366195	1.3796836	3.0954420
H	1.6768944	2.4509703	3.2876096
H	2.5389737	0.9456009	3.6902893
H	1.9390870	1.2168171	2.0419504

Calculations with inclusion of the counterion

[Co(acac)$_2$(L7)]PF$_6$, ^{5}A, ε_r = 1

Energy = -4500.287529670

C	6.6246820	7.1061131	6.6636694
C	7.2573205	7.0652579	3.9197758
C	6.7220349	8.2872413	5.9606176
C	7.0287542	8.2646848	4.5528120
H	6.3458440	7.1120294	7.7065430
H	7.5888507	7.0495057	2.8934828
O	6.5337663	9.5101447	6.4840611
O	7.0631380	9.4658919	3.9715810
C	6.2497208	9.6188419	7.8702308
H	5.3096734	9.1191464	8.1208024
H	7.0594583	9.1959392	8.4715448
C	6.8065658	5.8690997	6.0203731
C	7.1649713	5.8457539	4.6227384
N	6.5820681	4.6710094	6.6143087
N	7.4343136	4.6302723	4.1094170
Co	6.9839799	2.9341577	5.3726787
C	6.6378490	4.4676228	7.9177505
C	7.5631976	4.3940243	2.7954497
O	4.9955072	2.9894826	4.7951645
O	8.9342543	2.9318535	6.0602654
O	7.3191879	1.5475875	3.9040854
O	6.5862261	1.5395762	6.8566014
N	5.7099056	3.7682888	8.6217887
N	7.6695714	4.7740462	8.7617537
N	8.5774401	3.7057470	2.2391405
N	6.6287428	4.6412210	1.8358957
C	7.2720766	9.5350694	2.5541436
H	7.2045152	10.5908706	2.3073849
H	8.2492107	9.1424965	2.2825187
H	6.4873033	8.9810376	2.0324983

H	6.1624563	10.6827639	8.0729806
C	8.3107977	3.5112673	0.8937031
C	9.7726857	3.2469890	2.9290998
C	7.0702953	4.1055390	0.6330323
C	5.3197755	5.2156479	2.0656935
C	9.0370473	2.8834502	-0.1051152
H	9.7769054	2.1578458	2.9337871
H	10.6428673	3.6529176	2.4214524
H	9.7389769	3.6030733	3.9517792
C	6.5043327	4.0819178	-0.6319734
H	5.3006772	6.2684080	1.7819964
H	4.5893983	4.6628358	1.4768286
H	5.0703742	5.1153104	3.1181140
C	8.4707333	2.8548991	-1.3727549
H	10.0102415	2.4578638	0.0904413
H	5.5531165	4.5519550	-0.8390502
C	7.2261221	3.4409490	-1.6317877
H	9.0109212	2.3823537	-2.1819493
H	6.8231072	3.4064867	-2.6350360
C	6.4768209	1.0491497	3.1089526
C	7.4239729	0.8784734	7.5307238
C	5.1022064	1.3508359	3.0767788
C	7.0256165	0.0767349	2.0915937
C	8.8151431	1.0721116	7.5261546
C	6.8456448	-0.1809524	8.4418484
C	4.4497248	2.2698602	3.9067682
H	4.5015808	0.8314344	2.3448408
H	7.7082223	-0.6160472	2.5849163
H	6.2424947	-0.4786971	1.5781960
H	7.6005597	0.6332366	1.3468419
C	9.4850571	2.0690142	6.8044981
H	9.4114802	0.4167181	8.1434393
H	6.1266117	-0.7829835	7.8846782
H	7.6090789	-0.8262955	8.8731363
H	6.3054351	0.3084442	9.2570291
C	10.9891832	2.1615719	6.9091714
H	11.2776385	3.1841536	7.1595211
H	11.4044309	1.4738245	7.6438896
H	11.4242278	1.9417873	5.9313160
C	2.9576754	2.4576564	3.7458121
H	2.7385829	3.5162797	3.5914744
H	2.5457106	1.8783888	2.9212132
H	2.4605418	2.1589454	4.6718273
C	6.1583995	3.5908320	9.9216174
C	4.4582674	3.2703858	8.0768808
C	7.4006543	4.2373588	10.0164068
C	5.6006681	2.9311536	11.0047219
H	4.4423579	2.1845844	8.1410313
H	3.6227371	3.7007846	8.6308967
H	4.4048952	3.5445451	7.0267253
C	6.3272296	2.9335353	12.1922552

H	4.6458589	2.4298592	10.9291742
C	7.5647502	3.5750969	12.2865918
H	5.9253280	2.4263165	13.0588887
C	8.1227656	4.2412916	11.1986013
H	8.1027116	3.5546674	13.2246797
H	9.0798563	4.7376863	11.2775376
C	8.9346397	5.3479843	8.3336325
H	8.9375308	6.4317164	8.4517633
H	9.7313060	4.9117329	8.9325542
H	9.1001896	5.0900305	7.2904824
P	10.4675800	6.8793053	1.5357167
F	8.8563461	6.7646103	1.1788180
F	10.1476860	6.1761170	2.9791710
F	10.1959151	8.3345303	2.2100926
F	12.0255164	6.9694604	1.9153229
F	10.7236102	7.5700989	0.1085793
F	10.6932914	5.4067065	0.8809001

$[Co(acac)_2(L7)]PF_6$, $^{(3)}A$, $\varepsilon_r = 1$

Energy = -4500.285735003

C	6.6282537	7.0075019	6.6604779
C	7.2135570	6.9783585	3.9077549
C	6.7103326	8.1912672	5.9615719
C	6.9968433	8.1747018	4.5480866
H	6.3712568	7.0096389	7.7088768
H	7.5316683	6.9695530	2.8775383
O	6.5279039	9.4106749	6.4919691
O	7.0252665	9.3781016	3.9729103
C	6.2673684	9.5148134	7.8836838
H	5.3334184	9.0113068	8.1484571
H	7.0889266	9.0927807	8.4690962
C	6.8036680	5.7706778	6.0127789
C	7.1333243	5.7521569	4.6028247
N	6.6115184	4.5769802	6.6211531
N	7.3926895	4.5445663	4.0702033
Co	6.9643110	2.8266787	5.3649194
C	6.6683990	4.3960924	7.9283887
C	7.5224980	4.3335855	2.7530111
O	4.9787132	2.9015063	4.8110499
O	8.9600602	2.8338397	5.9811917
O	7.2907762	1.4266203	3.9050155
O	6.6122654	1.4815987	6.9047036
N	5.7314749	3.7267594	8.6485443
N	7.7038194	4.7084607	8.7642297
N	8.5302357	3.6417608	2.1865887
N	6.6061204	4.6296267	1.7884589
C	7.2192340	9.4563053	2.5535901
H	7.1481304	10.5135857	2.3144757
H	8.1940949	9.0662819	2.2707069

H	6.4290318	8.9049912	2.0373492
H	6.1804029	10.5779095	8.0904836
C	8.2790758	3.4996067	0.8314000
C	9.7187915	3.1531464	2.8689593
C	7.0521736	4.1225610	0.5755795
C	5.3006855	5.2115902	2.0132133
C	9.0095754	2.8965345	-0.1794166
H	9.7231689	2.0647279	2.8288234
H	10.5938547	3.5775955	2.3851431
H	9.6775064	3.4637276	3.9057937
C	6.5008650	4.1487508	-0.6956885
H	5.2829199	6.2570992	1.7034899
H	4.5632657	4.6464274	1.4448795
H	5.0589905	5.1424342	3.0691763
C	8.4590567	2.9195922	-1.4542770
H	9.9740104	2.4501477	0.0133210
H	5.5590330	4.6394405	-0.8978188
C	7.2258674	3.5312453	-1.7081011
H	9.0029263	2.4673364	-2.2725426
H	6.8347673	3.5360885	-2.7166319
C	6.4826361	1.0831040	3.0018384
C	7.4471542	0.9726456	7.7018863
C	5.1444498	1.5131890	2.8880415
C	7.0220436	0.1375131	1.9539163
C	8.8204616	1.2641844	7.7547733
C	6.8869575	-0.0269991	8.6898701
C	4.4711808	2.3390193	3.7945548
H	4.5712013	1.1158342	2.0634151
H	7.6158396	-0.6373792	2.4392782
H	6.2346565	-0.3186899	1.3562178
H	7.6838733	0.6915096	1.2834871
C	9.4954594	2.1373403	6.8881131
H	9.4119568	0.7355195	8.4875679
H	6.2707507	-0.7515197	8.1556655
H	7.6638474	-0.5468882	9.2478001
H	6.2411059	0.4941125	9.4010615
C	10.9961637	2.2689322	7.0160834
H	11.2778239	3.3226869	6.9900622
H	11.3848985	1.8029052	7.9203566
H	11.4640622	1.7953962	6.1493514
C	2.9957142	2.5942449	3.5812029
H	2.8004360	3.6675963	3.6221526
H	2.6307696	2.1927043	2.6371822
H	2.4352600	2.1345821	4.3989531
C	6.1766683	3.5777552	9.9536588
C	4.4757936	3.2279927	8.1125839
C	7.4291221	4.2059715	10.0310084
C	5.6087214	2.9574246	11.0545629
H	4.4409372	2.1459522	8.2193120
H	3.6427326	3.6919233	8.6427256
H	4.4336330	3.4614756	7.0521448

C	6.3373044	2.9780464	12.2405155
H	4.6451358	2.4711501	10.9936923
C	7.5866528	3.5991700	12.3165114
H	5.9277855	2.5011387	13.1206774
C	8.1542625	4.2271063	11.2110656
H	8.1258574	3.5929174	13.2540859
H	9.1205438	4.7076581	11.2746602
C	8.9713117	5.2692434	8.3295621
H	9.0078956	6.3444966	8.5064756
H	9.7738029	4.7788528	8.8770344
H	9.0977512	5.0682234	7.2692212
P	10.4499133	6.8355305	1.6110718
F	8.8513159	6.7345761	1.1970560
F	10.0832825	6.0846106	3.0187657
F	10.1540362	8.2674543	2.3256580
F	11.9941828	6.9125610	2.0449398
F	10.7527488	7.5750048	0.2179220
F	10.6965022	5.3860080	0.9148477

[Co(acac)$_2$(L7)]PF$_6$, $^{(1)}$A, ε_r = 1

Energy = -4500.270864029

C	6.6667653	7.1186122	6.6733816
C	7.2628132	7.0065490	3.9241568
C	6.7801213	8.2822513	5.9456992
C	7.0639889	8.2228929	4.5331866
H	6.4032524	7.1501206	7.7196783
H	7.5807633	6.9666496	2.8942596
O	6.6271416	9.5193065	6.4452421
O	7.1041905	9.4101693	3.9258284
C	6.3663441	9.6636608	7.8329948
H	5.4193107	9.1906667	8.1075449
H	7.1754556	9.2351188	8.4310227
C	6.8068323	5.8624131	6.0550185
C	7.1490872	5.8010455	4.6504352
N	6.5500583	4.6917524	6.6774108
N	7.3737040	4.5738558	4.1433863
Co	6.9840538	2.9302078	5.3383500
C	6.6420111	4.4604470	7.9621188
C	7.5115480	4.3496437	2.8222527
O	5.1374967	2.9896879	4.7692978
O	8.8026062	2.9918574	5.9623374
O	7.4458368	1.4693490	3.8716263
O	6.4844946	1.5235736	6.6224024
N	5.7445510	3.7043098	8.6591324
N	7.6752053	4.7710563	8.8115916
N	8.5375323	3.6917153	2.2539145
N	6.5768485	4.6062253	1.8668938
C	7.2645409	9.4482433	2.5008704
H	7.1945205	10.4991447	2.2345884

H	8.2295103	9.0446481	2.2048389
H	6.4584212	8.8883945	2.0194759
H	6.3061166	10.7333328	8.0140240
C	8.2722978	3.5160275	0.9052750
C	9.7451081	3.2411996	2.9295130
C	7.0245970	4.0984972	0.6550808
C	5.2690899	5.1817510	2.0979997
C	9.0068165	2.9160408	-0.1049184
H	9.7367034	2.1534763	2.9705096
H	10.6038500	3.6248119	2.3872934
H	9.7391436	3.6314152	3.9392657
C	6.4584573	4.0918665	-0.6104299
H	5.2430039	6.2237631	1.7773439
H	4.5308188	4.6066741	1.5409167
H	5.0390827	5.1179953	3.1569246
C	8.4409621	2.9043083	-1.3725200
H	9.9852857	2.4995103	0.0828693
H	5.5006365	4.5523676	-0.8082621
C	7.1885920	3.4792856	-1.6212717
H	8.9870452	2.4538447	-2.1902919
H	6.7862315	3.4576640	-2.6251507
C	6.6022859	1.0066316	3.0700626
C	7.2762722	0.9580361	7.4256526
C	5.2314040	1.3585088	3.0297440
C	7.1109492	0.0181269	2.0436404
C	8.6421722	1.2512486	7.5664952
C	6.6650054	-0.0925467	8.3223296
C	4.5967762	2.2726574	3.8664591
H	4.6163682	0.8562062	2.2976292
H	7.8474492	-0.6386669	2.5063017
H	6.3118063	-0.5738314	1.5994421
H	7.6120146	0.5705147	1.2438553
C	9.3221015	2.2206561	6.8254077
H	9.2099207	0.6750212	8.2817146
H	5.9199986	-0.6594414	7.7646977
H	7.4129265	-0.7680302	8.7346590
H	6.1592979	0.3994541	9.1580868
C	10.8078197	2.3917288	7.0216536
H	11.0642148	3.4512646	7.0376906
H	11.1649287	1.9083088	7.9295212
H	11.3271656	1.9514875	6.1665139
C	3.1053083	2.4726382	3.7263974
H	2.8805942	3.5381335	3.6501609
H	2.6935695	1.9511157	2.8642121
H	2.6119036	2.1037950	4.6290485
C	6.2101271	3.5075811	9.9496051
C	4.4856233	3.2219248	8.1196404
C	7.4362366	4.1843013	10.0498131
C	5.6813447	2.8078910	11.0213070
H	4.4257057	2.1424350	8.2401325
H	3.6525682	3.7041895	8.6346479

H	4.4605085	3.4444145	7.0563786
C	6.4223124	2.7944168	12.2014289
H	4.7356986	2.2894368	10.9449396
C	7.6449549	3.4606016	12.2988886
H	6.0412684	2.2556355	13.0584151
C	8.1726379	4.1710956	11.2220366
H	8.1951821	3.4278386	13.2294944
H	9.1176195	4.6899243	11.3040368
C	8.9226455	5.3865861	8.3966940
H	8.9263662	6.4572476	8.6039005
H	9.7414870	4.9090404	8.9314883
H	9.0581135	5.2238768	7.3308832
P	10.4766168	6.8466612	1.5389614
F	8.8656562	6.7241436	1.1823915
F	10.1595108	6.1464513	2.9842722
F	10.1997538	8.3024340	2.2094221
F	12.0343210	6.9435261	1.9176936
F	10.7293236	7.5349131	0.1098805
F	10.7080132	5.3735377	0.8871462

$[Co(acac)_2(L7)]PF_6$, ^1A, $\varepsilon_r = 1$

Energy = -4500.279700522

C	5.6794138	6.8347591	6.5315752
C	6.8583582	6.8847284	4.0141914
C	5.7877474	8.0412008	5.8565830
C	6.4051261	8.0656228	4.5851069
H	5.2320534	6.7854931	7.5134994
H	7.4039415	6.9144559	3.0860225
O	5.3448179	9.2386242	6.3330909
O	6.5077089	9.2813092	4.0012701
C	4.7734295	9.2619266	7.6235398
H	3.8752625	8.6367721	7.6785145
H	5.4870363	8.9277119	8.3849872
C	6.1582869	5.6569274	5.9559377
C	6.7057167	5.6643319	4.6819158
N	6.1606790	4.4243277	6.6684276
N	7.1946734	4.4231562	4.2386652
Co	6.4933222	2.9535693	5.3695604
C	6.8798741	4.3937746	7.7824607
C	7.5336262	4.2136966	2.9698749
O	4.7632859	3.2972359	4.6702615
O	8.2134020	2.6703871	6.1611216
O	6.9453339	1.6214830	4.0772665
O	5.6665653	1.5542275	6.3864446
N	6.5604945	3.6224308	8.8672676
N	8.0486991	5.0455643	8.0681788
N	8.6193555	3.5037187	2.5661893
N	6.8585579	4.6081136	1.8432924
C	7.1733904	9.3668438	2.7437741

H	7.1623709	10.4216840	2.4801545
H	8.1999496	9.0078453	2.8104094
H	6.6349048	8.7977380	1.9787121
H	4.5026990	10.2978286	7.8154274
C	8.6553796	3.4546759	1.1834183
C	9.6549325	2.9646459	3.4273732
C	7.5445899	4.1695420	0.7206593
C	5.5381360	5.2002041	1.7956340
C	9.5561635	2.8717681	0.3071440
H	9.5609168	1.8799481	3.4797533
H	10.6210351	3.2644771	3.0314758
H	9.5250486	3.3835224	4.4155953
C	7.2888258	4.3178259	-0.6328177
H	5.5887053	6.2276242	1.4347706
H	4.9119059	4.6072177	1.1281544
H	5.1092503	5.2006325	2.7901378
C	9.3026247	3.0195378	-1.0525051
H	10.4306838	2.3473515	0.6640998
H	6.4382264	4.8803608	-0.9918306
C	8.1876150	3.7250074	-1.5146069
H	9.9914268	2.5904823	-1.7677019
H	8.0262533	3.8236053	-2.5796973
C	6.1840257	1.2472843	3.1333987
C	6.3028111	0.6333438	6.9828088
C	4.8846291	1.7080300	2.9107834
C	6.7952498	0.2504838	2.1866885
C	7.6756795	0.6284028	7.2368664
C	5.4534438	-0.5190555	7.4508042
C	4.2491736	2.6783811	3.6928111
H	4.3356952	1.2882436	2.0822061
H	7.3272342	-0.5148598	2.7520256
H	6.0506237	-0.2131032	1.5428077
H	7.5258449	0.7665846	1.5583632
C	8.5374797	1.6630185	6.8594130
H	8.0878557	-0.1967064	7.7965831
H	5.1268700	-1.0879173	6.5768572
H	5.9921850	-1.1839024	8.1231056
H	4.5552049	-0.1420991	7.9412791
C	9.9676453	1.6550365	7.3254892
H	10.0981580	2.4401495	8.0744946
H	10.2500586	0.7015454	7.7667852
H	10.6283406	1.8889451	6.4903047
C	2.8255974	3.0626728	3.3878449
H	2.7340993	4.1491376	3.3714891
H	2.4803672	2.6470065	2.4433411
H	2.1839204	2.6966430	4.1926068
C	7.5379479	3.7650477	9.8403750
C	5.2862168	2.9648408	9.0705970
C	8.4768651	4.6783849	9.3379376
C	7.6774418	3.1905384	11.0929313
H	5.4362952	1.9018093	9.2525857

H	4.7799745	3.4078267	9.9306594
H	4.6922475	3.0928013	8.1717520
C	8.7993279	3.5500617	11.8358559
H	6.9497873	2.4890867	11.4770732
C	9.7410949	4.4512979	11.3342286
H	8.9437285	3.1217877	12.8185368
C	9.5944988	5.0339514	10.0777835
H	10.6041507	4.7060421	11.9344410
H	10.3245917	5.7357041	9.7004509
C	8.8608398	5.7842009	7.1006722
H	8.4662728	6.7851233	6.9423773
H	9.8726875	5.8487620	7.4897305
H	8.8874717	5.2538774	6.1553046
P	10.6822652	6.6936469	2.8747064
F	9.2838318	6.5214745	2.0226553
F	10.0086150	5.8146980	4.0869585
F	10.0863159	8.0418262	3.5553938
F	12.0369950	6.8300424	3.7327193
F	11.3069996	7.5508113	1.6685896
F	11.2282355	5.3136949	2.2017304

Calculations on the neutral complex

[Co(acac)$_2$(L7)], ^{4}A, ε_r = 1

Energy = -3559.599274761

C	6.5957028	6.9715494	6.7049474
C	7.5448157	6.9718362	4.1152993
C	6.8258205	8.1819899	6.0702018
C	7.3143877	8.1821357	4.7502067
H	6.1933285	6.9587835	7.7071978
H	7.9472546	6.9593000	3.1130724
O	6.6085041	9.4066307	6.6343502
O	7.5314270	9.4069097	4.1862384
C	6.1083050	9.4436555	7.9540118
H	5.1277377	8.9611528	8.0277614
H	6.7942684	8.9618187	8.6596194
C	6.8368103	5.7428843	6.0703055
C	7.3040167	5.7430209	4.7497836
N	6.5215389	4.5172143	6.6631013
N	7.6197371	4.5174689	4.1570068
Co	7.0704890	2.8122897	5.4099193
C	6.5706092	4.3248214	7.9496268
C	7.5712539	4.3249887	2.8704826
O	5.0997599	2.8315124	4.7185414
O	9.0414627	2.8306624	6.1012547
O	7.4562751	1.3678254	3.9673808
O	6.6844563	1.3679291	6.8526317
N	5.6706486	3.5504230	8.6385306
N	7.5107691	4.7584432	8.8640707

N	8.4715301	3.5505097	2.1820752
N	6.6314447	4.7584254	1.9555571
C	8.0319393	9.4442570	2.8667039
H	8.1287915	10.4970592	2.6102076
H	9.0126578	8.9620318	2.7931372
H	7.3462895	8.9623353	2.1608509
H	6.0112247	10.4963937	8.2106842
C	8.0946838	3.4614382	0.8536747
C	9.6771788	2.9940842	2.7639872
C	6.9304043	4.2338518	0.7048458
C	5.3703467	5.3725995	2.3206835
C	8.6567601	2.7920868	-0.2194084
H	9.6335949	1.9067278	2.7546510
H	10.5470935	3.3440655	2.2043636
H	9.7325074	3.3169946	3.8003086
C	6.2942211	4.3453898	-0.5185360
H	5.4011024	6.4559048	2.1960528
H	4.5841971	4.9523365	1.6948721
H	5.1567659	5.1405280	3.3605312
C	8.0167799	2.9065670	-1.4543891
H	9.5526602	2.1976421	-0.1044016
H	5.3972375	4.9376258	-0.6372655
C	6.8560543	3.6642788	-1.6000457
H	8.4298582	2.3945901	-2.3132394
H	6.3803777	3.7292335	-2.5695223
C	6.6825240	0.9534190	3.0658615
C	7.4582911	0.9527080	7.7536843
C	5.3492747	1.3603205	2.8704530
C	7.2645127	-0.0664604	2.1102606
C	8.7918308	1.3587893	7.9487925
C	6.8760148	-0.0671118	8.7091825
C	4.6367448	2.2252067	3.7116690
H	4.8116468	0.9134713	2.0470989
H	7.8101913	-0.8200657	2.6791760
H	6.5050877	-0.5464854	1.4950397
H	7.9774964	0.4299501	1.4475003
C	9.5044763	2.2237568	7.1077600
H	9.3294866	0.9113650	8.7718155
H	6.3289716	-0.8197501	8.1403018
H	7.6354570	-0.5483562	9.3234315
H	6.1642046	0.4297028	9.3729093
C	10.9740045	2.4537104	7.3913770
H	11.1896616	3.5229540	7.3584330
H	11.2869024	2.0452181	8.3513898
H	11.5634827	1.9828605	6.6006059
C	3.1673457	2.4557495	3.4278725
H	2.9518984	3.5250018	3.4617641
H	2.8546221	2.0482021	2.4673992
H	2.5775514	1.9842706	4.2180347
C	6.0480211	3.4616396	9.9668048
C	4.4646913	2.9940313	8.0572207

C	7.2122532	4.2342468	10.1150397
C	5.4864824	2.7923890	11.0402359
H	4.5080658	1.9066753	8.0669001
H	3.5950321	3.3443635	8.6170257
H	4.4090761	3.3166460	7.0208196
C	6.1268564	2.9073126	12.2749706
H	4.5906591	2.1977400	10.9256858
C	7.2874330	3.6653645	12.4200680
H	5.7142024	2.3954282	13.1340800
C	7.8487900	4.3462789	11.3381923
H	7.7633927	3.7306956	13.3893802
H	8.7457085	4.9387058	11.4564589
C	8.7716403	5.3727116	8.4983263
H	8.7407193	6.4560589	8.6225475
H	9.5580592	4.9528299	9.1240576
H	8.9849494	5.1402545	7.4585047

Calculations with inclusion of the counterion and the solvent effect

$[Co(acac)_2(L7)]PF_6$, ^5A, $\varepsilon_r = 37.5$

Energy = -4500.342266298

C	6.6877346	6.9381833	6.7007791
C	7.2726465	6.9543696	3.9505106
C	6.7687993	8.1327192	6.0239613
C	7.0533252	8.1387023	4.6131624
H	6.4302575	6.9248051	7.7483302
H	7.5563361	6.9612960	2.9106916
O	6.5855955	9.3429480	6.5753647
O	7.0800232	9.3556220	4.0467383
C	6.3165639	9.4143452	7.9795158
H	5.3871884	8.8955183	8.2215149
H	7.1422235	8.9856488	8.5509825
C	6.8707052	5.7116120	6.0318811
C	7.1945817	5.7182600	4.6256337
N	6.6981202	4.5031212	6.6116010
N	7.4551884	4.5170935	4.0663339
Co	7.0565805	2.7972823	5.3391434
C	6.6943513	4.3348949	7.9361071
C	7.5281104	4.3304990	2.7433361
O	5.0530837	2.8604773	4.7864591
O	9.0402532	2.7733741	5.9307378
O	7.3375949	1.4017141	3.8455684
O	6.6971577	1.3884325	6.8163027
N	5.7337598	3.6718268	8.6205757
N	7.6892316	4.6683655	8.8035960
N	8.5152282	3.6419521	2.1281066
N	6.5851955	4.6532605	1.8146217
C	7.2077725	9.4375608	2.6202550
H	7.1400420	10.4951991	2.3822879

H	8.1661973	9.0393456	2.2927726
H	6.3919406	8.8955069	2.1375743
H	6.2202521	10.4717080	8.2072369
C	8.2103063	3.5128729	0.7818438
C	9.7177393	3.1306102	2.7656292
C	6.9835773	4.1572455	0.5795821
C	5.2866089	5.2413906	2.0907614
C	8.8880312	2.8974845	-0.2588107
H	9.7274019	2.0449754	2.6891427
H	10.5871698	3.5578489	2.2728107
H	9.7014360	3.4065410	3.8142585
C	6.3830433	4.2042702	-0.6693927
H	5.2716741	6.2966448	1.8202458
H	4.5331599	4.7086259	1.5142841
H	5.0670749	5.1345856	3.1480739
C	8.2886107	2.9417001	-1.5126261
H	9.8394010	2.4086821	-0.1044574
H	5.4371059	4.7029211	-0.8285501
C	7.0587105	3.5817613	-1.7142776
H	8.7848037	2.4726978	-2.3519842
H	6.6247049	3.5954143	-2.7053125
C	6.5084946	1.0377198	2.9609089
C	7.5003297	0.9790945	7.7058935
C	5.1645143	1.4402250	2.8857990
C	7.0393585	0.0985104	1.9044680
C	8.8555714	1.3317891	7.8078225
C	6.9221866	0.0369265	8.7351772
C	4.5133054	2.2911069	3.7926764
H	4.5785264	1.0508217	2.0664738
H	7.4734866	-0.7797974	2.3869499
H	6.2709773	-0.2176452	1.2014687
H	7.8395152	0.5957271	1.3520439
C	9.5487476	2.1705690	6.9191783
H	9.4160921	0.9097227	8.6287953
H	6.4948051	-0.8315670	8.2290984
H	7.6625179	-0.2964979	9.4599476
H	6.1084785	0.5358001	9.2657271
C	11.0287332	2.3840434	7.1315085
H	11.2568149	3.4497709	7.0795067
H	11.3786622	1.9792252	8.0796468
H	11.5745127	1.8979253	6.3183672
C	3.0423629	2.5695263	3.5933975
H	2.8713694	3.6477533	3.5918596
H	2.6552853	2.1393583	2.6714028
H	2.4859761	2.1561783	4.4386298
C	6.1159077	3.5615880	9.9508296
C	4.4776761	3.1970623	8.0612897
C	7.3600373	4.1963173	10.0691631
C	5.4944324	2.9694959	11.0398637
H	4.3727127	2.1335686	8.2648130
H	3.6492163	3.7447148	8.5103647

H	4.4965138	3.3463520	6.9861743
C	6.1689828	3.0262916	12.2553757
H	4.5334134	2.4826942	10.9501780
C	7.4153170	3.6544053	12.3722439
H	5.7197967	2.5741099	13.1294988
C	8.0340875	4.2540669	11.2795358
H	7.9089359	3.6765954	13.3346499
H	8.9937970	4.7425266	11.3743623
C	8.9626762	5.2627663	8.4324465
H	8.9682771	6.3323197	8.6400733
H	9.7525403	4.7790297	9.0026911
H	9.1329297	5.0945007	7.3732648
P	10.5543727	6.8762465	1.3633302
F	8.9627818	6.7653541	1.0448524
F	10.3116680	6.1900470	2.8120890
F	10.3211547	8.3463028	2.0238186
F	12.1451204	6.9948439	1.6826498
F	10.7920333	7.5736627	-0.0856464
F	10.7970882	5.4174566	0.7004196

$[Co(acac)_2(L7)]PF_6$, $^{(3)}$A, ε_r = 37.5

Energy = -4500.341326180

C	6.7041366	6.8499374	6.7054695
C	7.2721777	6.8807809	3.9533210
C	6.7713168	8.0471485	6.0340799
C	7.0533018	8.0610338	4.6211151
H	6.4560836	6.8325839	7.7550134
H	7.5491966	6.8945473	2.9120027
O	6.5806392	9.2524289	6.5897815
O	7.0755148	9.2799063	4.0622263
C	6.3149226	9.3179907	7.9954260
H	5.3918078	8.7887844	8.2383460
H	7.1471483	8.8967822	8.5627250
C	6.8964625	5.6241600	6.0351309
C	7.1996687	5.6380177	4.6189174
N	6.7604835	4.4193310	6.6272846
N	7.4436788	4.4454281	4.0381294
Co	7.0731934	2.7007256	5.3327075
C	6.7280519	4.2779585	7.9557255
C	7.5055166	4.2835948	2.7119225
O	5.0699974	2.7488710	4.8271447
O	9.0814211	2.6269834	5.8502423
O	7.3267799	1.2956388	3.8341419
O	6.7517189	1.3081640	6.8526470
N	5.7522337	3.6291635	8.6313551
N	7.6974904	4.6391779	8.8397302
N	8.4762065	3.5858570	2.0796008
N	6.5712844	4.6509388	1.7902170
C	7.2070607	9.3724155	2.6362579

H	7.1378024	10.4316064	2.4061727
H	8.1671659	8.9780955	2.3092864
H	6.3934180	8.8323489	2.1479820
H	6.2083990	10.3737914	8.2253946
C	8.1663108	3.4923558	0.7311724
C	9.6792548	3.0541628	2.6991766
C	6.9545458	4.1684849	0.5454809
C	5.2818583	5.2526691	2.0763383
C	8.8279629	2.8841753	-0.3236078
H	9.6865301	1.9706712	2.5962955
H	10.5481662	3.4895398	2.2124655
H	9.6695039	3.3031856	3.7543646
C	6.3510890	4.2528320	-0.7000689
H	5.2746445	6.3061237	1.7984929
H	4.5161742	4.7238130	1.5124712
H	5.0718789	5.1583647	3.1364010
C	8.2265820	2.9668557	-1.5746150
H	9.7684465	2.3710354	-0.1820330
H	5.4157202	4.7749251	-0.8460572
C	7.0101379	3.6368960	-1.7595579
H	8.7102893	2.5041395	-2.4246922
H	6.5736099	3.6795253	-2.7486658
C	6.5358056	1.0858917	2.8688975
C	7.5298963	1.0600478	7.8179011
C	5.2335268	1.6034591	2.7540010
C	7.0430482	0.1736656	1.7775200
C	8.8538977	1.5184814	7.9374620
C	6.9822777	0.1674306	8.9069830
C	4.5629843	2.3440172	3.7396729
H	4.6767105	1.3450580	1.8652959
H	7.1195040	-0.8434015	2.1713558
H	6.3911222	0.1645692	0.9059619
H	8.0446391	0.4817615	1.4759608
C	9.5620292	2.2167530	6.9465679
H	9.3940656	1.2425264	8.8312142
H	6.9179409	-0.8559965	8.5271469
H	7.6062876	0.1692686	9.7989296
H	5.9715559	0.4801327	9.1701913
C	11.0316817	2.4887970	7.1657001
H	11.2539647	3.5269009	6.9140829
H	11.3479968	2.2811007	8.1867349
H	11.6120513	1.8599940	6.4850605
C	3.1053641	2.6771933	3.5273324
H	2.9333422	3.7306083	3.7538700
H	2.7698533	2.4592839	2.5146799
H	2.5037587	2.0930186	4.2289309
C	6.1036270	3.5481172	9.9726919
C	4.5038669	3.1533201	8.0548333
C	7.3429027	4.1887625	10.1058380
C	5.4599616	2.9757856	11.0591900
H	4.3852177	2.0946567	8.2755583

H	3.6716377	3.7147324	8.4794357
H	4.5430689	3.2832105	6.9776220
C	6.1083760	3.0571592	12.2873498
H	4.5027533	2.4838534	10.9580265
C	7.3512077	3.6896267	12.4188616
H	5.6417480	2.6201811	13.1600732
C	7.9919569	4.2699960	11.3285139
H	7.8245331	3.7299985	13.3908229
H	8.9491113	4.7613525	11.4337904
C	8.9774487	5.2298525	8.4894946
H	8.9957730	6.2903263	8.7388844
H	9.7641550	4.7144962	9.0362016
H	9.1421722	5.1054360	7.4238952
P	10.5479258	6.8221003	1.3749299
F	8.9566602	6.7353522	1.0476140
F	10.2920180	6.1071181	2.8071980
F	10.3273286	8.2798841	2.0666069
F	12.1384553	6.9166461	1.7028267
F	10.7989703	7.5485068	-0.0575488
F	10.7775282	5.3755425	0.6809612

$[Co(acac)_2(L7)]PF_6$, $^{(1)}$A, ε_r = 37.5

Energy = -4500.325014613

C	6.7528987	6.9891677	6.7154416
C	7.3034039	6.9235304	3.9591171
C	6.8561886	8.1639755	6.0102518
C	7.1190684	8.1280667	4.5950420
H	6.5082946	7.0056507	7.7660411
H	7.5727337	6.9021121	2.9155242
O	6.7125507	9.3921421	6.5339512
O	7.1589388	9.3298321	3.9978506
C	6.4649350	9.5039527	7.9392570
H	5.5254556	9.0162949	8.2058933
H	7.2863886	9.0663163	8.5100137
C	6.8881559	5.7393079	6.0745317
C	7.1990971	5.7035440	4.6615239
N	6.6724529	4.5587567	6.6794446
N	7.4117429	4.4880038	4.1132588
Co	7.0791820	2.8204636	5.3254643
C	6.6724662	4.3692197	7.9897649
C	7.4801931	4.3055875	2.7854237
O	5.2197490	2.8558290	4.7918447
O	8.9162698	2.8984684	5.8844284
O	7.5020980	1.3433571	3.8473940
O	6.6268544	1.4257504	6.6531603
N	5.7208030	3.6574338	8.6470827
N	7.6509202	4.6983962	8.8837755
N	8.4737549	3.6442058	2.1531720
N	6.5259458	4.6277139	1.8696184

C	7.2347927	9.3750838	2.5659261
H	7.1728350	10.4276058	2.3045621
H	8.1744418	8.9555579	2.2130615
H	6.3937730	8.8334799	2.1280546
H	6.4001292	10.5686243	8.1429573
C	8.1558156	3.5221621	0.8090766
C	9.6986793	3.1585784	2.7685913
C	6.9181768	4.1509612	0.6257538
C	5.2312670	5.2156997	2.1621165
C	8.8315847	2.9273244	-0.2448515
H	9.7155103	2.0719451	2.7207201
H	10.5477071	3.5839318	2.2408407
H	9.7056852	3.4609030	3.8095658
C	6.3046045	4.2036298	-0.6168880
H	5.2097609	6.2664963	1.8749515
H	4.4673899	4.6730670	1.6089020
H	5.0343073	5.1245809	3.2251366
C	8.2191793	2.9766904	-1.4918539
H	9.7914749	2.4510299	-0.1054230
H	5.3502110	4.6905897	-0.7611241
C	6.9784617	3.6019051	-1.6746891
H	8.7133004	2.5236099	-2.3411358
H	6.5345840	3.6197792	-2.6612901
C	6.6717470	0.9827309	2.9736119
C	7.3987475	1.0304656	7.5751505
C	5.3262440	1.4031875	2.9036944
C	7.1739537	0.0226369	1.9183183
C	8.7313403	1.4371633	7.7465121
C	6.8109662	0.0376779	8.5467300
C	4.6846775	2.2530380	3.8094834
H	4.7259137	1.0050977	2.0990120
H	7.6164270	-0.8497754	2.4036239
H	6.3895626	-0.3011782	1.2364664
H	7.9625539	0.5095092	1.3402181
C	9.4186428	2.2983749	6.8832799
H	9.2797599	1.0219294	8.5786609
H	6.4886795	-0.8529041	8.0025117
H	7.5174947	-0.2518105	9.3220153
H	5.9249186	0.4680150	9.0172156
C	10.8882885	2.5453483	7.1091639
H	11.1116050	3.6049204	6.9813259
H	11.2151059	2.2144719	8.0934000
H	11.4581553	1.9994674	6.3522169
C	3.2033885	2.4972559	3.6506607
H	2.9945935	3.5654368	3.7251007
H	2.8188206	2.1134693	2.7074533
H	2.6737448	2.0059085	4.4712785
C	6.0931365	3.5124397	9.9758663
C	4.4723275	3.1997210	8.0587978
C	7.3221820	4.1706600	10.1272771
C	5.4771759	2.8707083	11.0389966

H	4.3335999	2.1439302	8.2817156
H	3.6400880	3.7733964	8.4679012
H	4.5321036	3.3255555	6.9818747
C	6.1410169	2.8989175	12.2626454
H	4.5271855	2.3674902	10.9250114
C	7.3718605	3.5481433	12.4115905
H	5.6942920	2.4070246	13.1164299
C	7.9852834	4.1991091	11.3440550
H	7.8581731	3.5476370	13.3780240
H	8.9332607	4.7048708	11.4638513
C	8.9205333	5.3195861	8.5492236
H	8.9225704	6.3749393	8.8204948
H	9.7151289	4.8071411	9.0875828
H	9.0912990	5.2193581	7.4818646
P	10.5674527	6.8570106	1.3054869
F	8.9707690	6.7356394	1.0193354
F	10.3552644	6.1895882	2.7676700
F	10.3419989	8.3349279	1.9508883
F	12.1638627	6.9853637	1.5925965
F	10.7748245	7.5361607	-0.1567071
F	10.8037596	5.3904523	0.6571730

$[Co(acac)_2(L7)]PF_6$, ^1A, $\varepsilon_r = 37.5$

Energy = -4500.333329473

C	5.9224406	6.8676005	6.5513811
C	6.9224235	6.8948648	3.9610931
C	6.0344752	8.0757051	5.8775948
C	6.5453015	8.0845087	4.5647210
H	5.5349528	6.8323015	7.5590478
H	7.3786808	6.9139719	2.9861507
O	5.6792741	9.2886788	6.3992343
O	6.6387170	9.3079496	3.9652677
C	5.2017954	9.3137154	7.7399025
H	4.2853198	8.7267946	7.8455716
H	5.9561629	8.9340571	8.4347001
C	6.3142655	5.6737983	5.9416352
C	6.7939210	5.6761559	4.6362463
N	6.2514601	4.4308247	6.6157935
N	7.2343180	4.4333430	4.1581756
Co	6.6061981	2.9585289	5.3312468
C	6.8350866	4.3540102	7.8095773
C	7.4949253	4.2260388	2.8725017
O	4.8492637	3.2413967	4.6582285
O	8.3568081	2.7607557	6.0644437
O	7.0673974	1.6131257	4.0629526
O	5.8530765	1.5673835	6.3980449
N	6.3489517	3.6223371	8.8506710
N	8.0117136	4.9221124	8.2112298
N	8.5594033	3.5160537	2.4094316

N	6.7632291	4.6290507	1.7860474
C	7.0499815	9.3488033	2.5998759
H	7.0225987	10.3972956	2.3127656
H	8.0609498	8.9587470	2.4792560
H	6.3598064	8.7812598	1.9692154
H	4.9929014	10.3567196	7.9649501
C	8.5140029	3.4599195	1.0266626
C	9.6428931	2.9865384	3.2173569
C	7.3818495	4.1816758	0.6269553
C	5.4284875	5.2004888	1.8160024
C	9.3542172	2.8585674	0.1023129
H	9.5664804	1.9024149	3.2782327
H	10.5877705	3.2819518	2.7682702
H	9.5574038	3.4032831	4.2128444
C	7.0443055	4.3233666	-0.7100454
H	5.4395282	6.2289383	1.4565731
H	4.7762967	4.6025100	1.1806001
H	5.0573543	5.1835617	2.8333380
C	9.0205854	3.0024788	-1.2422795
H	10.2291151	2.3026984	0.4089981
H	6.1699666	4.8787006	-1.0202589
C	7.8855344	3.7175800	-1.6409021
H	9.6534081	2.5501140	-1.9943907
H	7.6553400	3.8036614	-2.6946269
C	6.2914634	1.1799629	3.1546428
C	6.5316638	0.7107876	7.0477623
C	4.9785306	1.6056577	2.9411669
C	6.8984179	0.1488219	2.2444310
C	7.9064370	0.7779275	7.2838468
C	5.7407492	-0.4468409	7.5904927
C	4.3322699	2.5848747	3.7018738
H	4.4248396	1.1467292	2.1368298
H	7.4252730	-0.5997351	2.8369975
H	6.1510061	-0.3315959	1.6167897
H	7.6351054	0.6362706	1.6004512
C	8.7273615	1.8065740	6.8180579
H	8.3541797	0.0087171	7.8936130
H	5.4643314	-1.0990219	6.7575421
H	6.3084759	-1.0256059	8.3158950
H	4.8146832	-0.0905759	8.0427533
C	10.1700827	1.8593462	7.2365753
H	10.3135459	2.7091061	7.9087701
H	10.4794703	0.9517335	7.7499468
H	10.8013459	2.0250474	6.3626112
C	2.8981031	2.9258862	3.4044483
H	2.7765997	4.0089336	3.3600160
H	2.5565769	2.4779288	2.4738326
H	2.2733183	2.5609877	4.2236817
C	7.2239492	3.7090871	9.9236501
C	5.0153189	3.0511943	8.9201796
C	8.2746145	4.5472479	9.5233728

C	7.1823221	3.1451034	11.1892514
H	5.0735540	1.9911571	9.1565600
H	4.4442898	3.5611462	9.6967756
H	4.5412532	3.1784473	7.9534570
C	8.2385900	3.4404350	12.0473004
H	6.3689868	2.5028473	11.4975360
C	9.2927825	4.2687757	11.6462519
H	8.2436659	3.0192253	13.0438249
C	9.3277057	4.8410718	10.3770958
H	10.0997618	4.4724876	12.3375610
H	10.1414174	5.4862943	10.0770377
C	8.9384060	5.6449311	7.3484005
H	8.6676492	6.6976088	7.2851774
H	9.9356905	5.5512627	7.7703362
H	8.9319008	5.2043290	6.3568568
P	10.7792134	6.8117503	2.8153662
F	9.4406498	6.5315059	1.9362616
F	10.1791328	5.9385276	4.0354571
F	10.1053383	8.1528197	3.4332114
F	12.1314150	7.0940582	3.6785221
F	11.3892051	7.6927407	1.5875565
F	11.4677114	5.4782121	2.1853579

3.2.3 Optimized coordinates by TPSSh calculations

Calculations without counterion and without solvent effect

[Co(acac)$_2$(L7)]$^+$, ^{5}A, ε_r = 1

Energy = -3560.839380921

C	6.6995197	7.1083183	6.7666263
C	7.4423149	7.1083345	4.0534207
C	6.8806421	8.3052009	6.1021656
C	7.2613378	8.3052088	4.7179362
H	6.3763907	7.1016805	7.7988491
H	7.7654183	7.1017053	3.0211899
O	6.7198158	9.5236800	6.6454068
O	7.4223059	9.5236949	4.1747503
C	6.3373725	9.5945933	8.0186483
H	5.3647958	9.1189127	8.1738056
H	7.0912592	9.1204240	8.6540898
C	6.8732204	5.8810007	6.0986722
C	7.2684912	5.8810057	4.7213261
N	6.6192157	4.6586937	6.6355399
N	7.5223232	4.6586794	4.1844225
Co	7.0708033	2.9994256	5.4099748
C	6.5464845	4.4546046	7.9538862
C	7.5950076	4.4546113	2.8660681
O	5.1358686	2.9790672	4.7559627
O	9.0057482	2.9791669	6.0639702

O	7.5036710	1.6276284	3.9828523
O	6.6380064	1.6276422	6.8371297
N	5.5386703	3.7944371	8.5708443
N	7.5096755	4.7327040	8.8782835
N	8.6027647	3.7943993	2.2490684
N	6.6317674	4.7327134	1.9417294
C	7.8047506	9.5946256	2.8015104
H	7.8691001	10.6548711	2.5726538
H	8.7772670	9.1188307	2.6463251
H	7.0508016	9.1205812	2.1660496
H	6.2731540	10.6548357	8.2475557
C	8.2825668	3.6227118	0.9090165
C	9.8128097	3.3239638	2.9096068
C	7.0315263	4.2246505	0.7091398
C	5.3184246	5.2745006	2.2590630
C	8.9655212	3.0012287	-0.1273820
H	9.8833253	2.2437393	2.7925290
H	10.6812727	3.8154133	2.4679149
H	9.7307464	3.5547782	3.9696832
C	6.4152710	4.2244648	-0.5345069
H	5.2881418	6.3511404	2.0849401
H	4.5822478	4.7773350	1.6289824
H	5.1021035	5.0551895	3.3027963
C	8.3476696	2.9995438	-1.3738363
H	9.9305897	2.5360896	0.0255803
H	5.4509425	4.6886353	-0.6953807
C	7.0974695	3.5979034	-1.5728467
H	8.8452573	2.5240976	-2.2095147
H	6.6507437	3.5742383	-2.5586858
C	6.6760620	0.8741725	3.3834319
C	7.4656499	0.8742523	7.4365841
C	5.2787650	1.0098962	3.4307500
C	7.2780867	-0.2185038	2.5348463
C	8.8629408	1.0100422	7.3892635
C	6.8636778	-0.2184410	8.2851851
C	4.5933725	2.0372484	4.0885791
H	4.6890877	0.2891800	2.8806234
H	8.0079857	-0.7748130	3.1266078
H	6.5262363	-0.9019099	2.1420340
H	7.8128112	0.2348694	1.6946328
C	9.5482861	2.0373954	6.7313869
H	9.4526508	0.2893876	7.9394359
H	6.1337908	-0.7747813	7.6934382
H	7.6155597	-0.9018157	8.6779918
H	6.3289500	0.2349189	9.1254038
C	11.0550060	2.0792794	6.8102293
H	11.3794532	3.0724113	7.1302983
H	11.4586860	1.3276956	7.4875574
H	11.4684760	1.9118692	5.8113986
C	3.0866548	2.0791002	4.0096770
H	2.7622020	3.0722104	3.6895457

H	2.6830147	1.3274762	3.3323698
H	2.6731490	1.9117321	5.0084998
C	5.8587610	3.6228108	9.9109285
C	4.3286547	3.3240100	7.9102478
C	7.1098144	4.2247010	10.1108644
C	5.1757294	3.0013610	10.9472959
H	4.2580971	2.2437935	8.0273758
H	3.4601760	3.8155075	8.3518561
H	4.4107967	3.5547685	6.8501657
C	5.7934979	2.9996972	12.1937917
H	4.2106748	2.5362121	10.7942754
C	7.0437175	3.5979966	12.3928586
H	5.2958119	2.5243466	13.0294660
C	7.7260116	4.2244894	11.3545395
H	7.4903720	3.5743597	13.3787307
H	8.6903710	4.6885836	11.5154485
C	8.8230824	5.2743786	8.5610240
H	8.8534463	6.3510185	8.7351317
H	9.5591762	4.7771597	9.1911599
H	9.0394541	5.0550334	7.5173087

$[Co(acac)_2(L7)]^+$, $^{(3)}A$, $\varepsilon_r = 1$

Energy = -3560.834249963

C	-0.2406564	3.0090605	1.3783233
C	0.2831823	3.0134580	-1.3885221
C	-0.1100139	4.2049372	0.7046576
C	0.1602400	4.2071999	-0.7105521
H	-0.4848090	3.0029930	2.4317201
H	0.5209999	3.0082920	-2.4435128
O	-0.2258683	5.4211734	1.2564011
O	0.2810696	5.4249733	-1.2574788
C	-0.4972969	5.4967569	2.6573172
H	-1.4554741	5.0239356	2.8893033
H	0.3044557	5.0215988	3.2296753
C	-0.1289446	1.7770259	0.7009303
C	0.1651130	1.7793540	-0.7147352
N	-0.3602589	0.5699432	1.2619702
N	0.3695309	0.5746246	-1.2853423
Co	0.0275060	-1.1424618	-0.0242868
C	-0.3789733	0.3750039	2.5820492
C	0.3789278	0.3904634	-2.6095749
O	-1.9885514	-1.1012780	-0.5283351
O	1.9968411	-1.0599422	0.5064425
O	0.3290276	-2.4134036	-1.5773980
O	-0.3304298	-2.5106960	1.4229677
N	-1.3558552	-0.2970735	3.2365884
N	0.6083028	0.6770556	3.4755245
N	1.3755505	-0.2266745	-3.2844793
N	-0.6407168	0.6421260	-3.4777513

C	0.5494491	5.5043054	-2.6589091
H	0.5974458	6.5659336	-2.8844832
H	1.5044569	5.0270077	-2.8948044
H	-0.2562699	5.0355877	-3.2309310
H	-0.5399306	6.5577829	2.8867842
C	0.9917449	-0.3963610	-4.6081805
C	2.6215577	-0.6881342	-2.6852037
C	-0.2909882	0.1562473	-4.7338753
C	-1.9490425	1.1498572	-3.0944896
C	1.6407212	-0.9823767	-5.6864502
H	2.7252268	-1.7561595	-2.8698501
H	3.4595937	-0.1418318	-3.1212624
H	2.5653674	-0.5242972	-1.6108242
C	-0.9765346	0.1375162	-5.9405339
H	-2.0450011	2.2061905	-3.3508772
H	-2.7096248	0.5709719	-3.6169320
H	-2.0690147	1.0091806	-2.0227142
C	0.9541619	-1.0020321	-6.8960161
H	2.6304315	-1.4095345	-5.5913004
H	-1.9673783	0.5607334	-6.0421384
C	-0.3289118	-0.4545475	-7.0201152
H	1.4226561	-1.4522275	-7.7619362
H	-0.8290672	-0.4917676	-7.9795176
C	-0.5460976	-2.9851669	-2.2966264
C	0.5174082	-2.9686666	2.2471179
C	-1.9308643	-2.7528678	-2.2392851
C	-0.0191184	-3.9857560	-3.2971753
C	1.8786300	-2.6128765	2.3093306
C	-0.0076419	-3.9850190	3.2332523
C	-2.5694497	-1.8658596	-1.3618649
H	-2.5580794	-3.3338758	-2.9019075
H	0.6372280	-4.6932541	-2.7862455
H	-0.8172102	-4.5267604	-3.8041455
H	0.5813942	-3.4635038	-4.0476274
C	2.5410037	-1.7413394	1.4359228
H	2.4821251	-3.1136085	3.0546570
H	-0.5381919	-4.7700195	2.6906897
H	0.7835880	-4.4274395	3.8372423
H	-0.7282324	-3.5035128	3.9004421
C	4.0382071	-1.5861458	1.5650614
H	4.3072989	-0.5280448	1.5282230
H	4.4269955	-2.0348154	2.4789095
H	4.5180021	-2.0702884	0.7093627
C	-4.0783915	-1.7895385	-1.3799814
H	-4.3953442	-0.7442707	-1.4086562
H	-4.5147135	-2.3298548	-2.2196382
H	-4.4649018	-2.2183706	-0.4506853
C	-0.9918559	-0.4493421	4.5679031
C	-2.5860213	-0.7826150	2.6236015
C	0.2577938	0.1675571	4.7218380
C	-1.6343941	-1.0673857	5.6318652

H	-2.6683798	-1.8541862	2.7986327
H	-3.4390987	-0.2592170	3.0590553
H	-2.5264136	-0.6101257	1.5505555
C	-0.9736387	-1.0553846	6.8560684
H	-2.6002243	-1.5414525	5.5156510
C	0.2782509	-0.4466702	7.0076208
H	-1.4381879	-1.5289421	7.7115834
C	0.9182949	0.1791861	5.9422981
H	0.7595051	-0.4613288	7.9772446
H	1.8842912	0.6513600	6.0655737
C	1.9075149	1.2274732	3.1235903
H	1.9679886	2.2832318	3.3928963
H	2.6751717	0.6655879	3.6543507
H	2.0527088	1.1071905	2.0527470

[Co(acac)$_2$(L7)]$^+$, ^{1}A, ε_r = 1

Energy = -3560.852228949

C	-0.4752361	2.9188011	1.3074393
C	0.4749128	2.9187856	-1.3075749
C	-0.2478411	4.1270190	0.6621010
C	0.2473526	4.1270107	-0.6623060
H	-0.8710441	2.9090706	2.3145136
H	0.8704320	2.9090634	-2.3147623
O	-0.4647337	5.3491330	1.2136562
O	0.4640526	5.3491224	-1.2139428
C	-0.9818175	5.3848730	2.5356419
H	-1.9590750	4.8933835	2.5906790
H	-0.2925266	4.9091467	3.2424903
C	-0.2212423	1.7022574	0.6589258
C	0.2212193	1.7022336	-0.6589523
N	-0.4682429	0.4395558	1.2257658
N	0.4682376	0.4394933	-1.2257040
Co	-0.0000686	-1.0193929	0.0000554
C	-0.4395486	0.2704471	2.5411280
C	0.4396551	0.2703103	-2.5410581
O	-1.7905798	-0.9546894	-0.6168290
O	1.7904566	-0.9549737	0.6169441
O	0.5627222	-2.3864907	-1.1808442
O	-0.5630902	-2.3863955	1.1809546
N	-1.3652617	-0.4478733	3.2398576
N	0.4839658	0.7394017	3.4372655
N	1.3654523	-0.4480340	-3.2396488
N	-0.4837003	0.7393119	-3.4373416
C	0.9811529	5.3848552	-2.5359212
H	1.0858293	6.4386754	-2.7827293
H	1.9585275	4.8935925	-2.5908949
H	0.2919869	4.9088961	-3.2427359
H	-1.0867381	6.4386925	2.7823492
C	1.0242684	-0.4585223	-4.5850152

C	2.6230924	-0.9188662	-2.6788387
C	-0.1411701	0.3117791	-4.7146940
C	-1.7202584	1.4157790	-3.0793201
C	1.6303109	-1.0509972	-5.6839382
H	2.6837922	-2.0039282	-2.7401675
H	3.4484815	-0.4658431	-3.2313627
H	2.6455060	-0.6217393	-1.6329698
C	-0.7459726	0.5105672	-5.9478316
H	-1.6403041	2.4891251	-3.2569568
H	-2.5281523	0.9995117	-3.6812465
H	-1.9158670	1.2374931	-2.0253432
C	1.0244402	-0.8511180	-6.9216684
H	2.5339841	-1.6390335	-5.5872406
H	-1.6434628	1.1061435	-6.0543202
C	-0.1424004	-0.0898229	-7.0498633
H	1.4674652	-1.2939117	-7.8046756
H	-0.5852321	0.0393066	-8.0293027
C	-0.2172838	-3.0233152	-1.9635550
C	0.2168154	-3.0233641	1.9636505
C	-1.5815468	-2.7756294	-2.1303257
C	0.4462853	-4.0969255	-2.7838974
C	1.5811209	-2.7759068	2.1304096
C	-0.4469301	-4.0968770	2.7839782
C	-2.2865168	-1.7762993	-1.4567994
H	-2.1260962	-3.3987447	-2.8255043
H	1.2333930	-4.5761501	-2.2017359
H	-0.2725039	-4.8409800	-3.1248879
H	0.9039598	-3.6407152	-3.6674326
C	2.2862533	-1.7766784	1.4568989
H	2.1255776	-3.3991457	2.8255492
H	-1.2340404	-4.5760390	2.2017654
H	0.2717541	-4.8409954	3.1250507
H	-0.9046381	-3.6405924	3.6674588
C	3.7650756	-1.6214113	1.6912803
H	4.0297861	-0.5640253	1.7274524
H	4.0857036	-2.1195617	2.6052148
H	4.3024569	-2.0651222	0.8477907
C	-3.7653191	-1.6208001	-1.6911580
H	-4.0298857	-0.5633726	-1.7271944
H	-4.0860154	-2.1187944	-2.6051553
H	-4.3027599	-2.0645523	-0.8477249
C	-1.0238420	-0.4584126	4.5851653
C	-2.6229159	-0.9188508	2.6791990
C	0.1417423	0.3117030	4.7146387
C	-1.6296313	-1.0510145	5.6841618
H	-2.6834913	-2.0039204	2.7405592
H	-3.4482942	-0.4659034	3.2318037
H	-2.6454750	-0.6217519	1.6333227
C	-1.0234223	-0.8513527	6.9217630
H	-2.5332674	-1.6391324	5.5875910
C	0.1436051	-0.0903045	7.0497358

H	-1.4663550	-1.2940802	7.8048513
C	0.7469329	0.5101951	5.9476319
H	0.5867829	0.0385347	8.0290569
H	1.6445063	1.1056702	6.0539668
C	1.7203349	1.4161093	3.0790542
H	1.6402481	2.4894185	3.2568454
H	2.5284313	0.9998995	3.6807448
H	1.9157350	1.2379860	2.0250128

Calculations with inclusion of the counterion

[Co(acac)$_2$(L7)]PF_6, ^5A, $\varepsilon_r = 1$

Energy = -4501.943772372

C	6.5837859	7.1130859	6.6602597
C	7.2748359	7.0753598	3.9264872
C	6.6903420	8.2960360	5.9563237
C	7.0313056	8.2759626	4.5600897
H	6.2818775	7.1161861	7.6990362
H	7.6334579	7.0600404	2.9061409
O	6.4812923	9.5213918	6.4774775
O	7.0829063	9.4812440	3.9801307
C	6.1627351	9.6019883	7.8616744
H	5.2214002	9.0863458	8.0764165
H	6.9651201	9.1739839	8.4709214
C	6.7905012	5.8811978	6.0160364
C	7.1644288	5.8598992	4.6302128
N	6.5735637	4.6691263	6.6011181
N	7.4408358	4.6319317	4.1274971
Co	6.9841307	2.9840357	5.3775590
C	6.6340567	4.4885991	7.9117262
C	7.5651973	4.4093079	2.8085620
O	5.0157623	3.0143515	4.8022742
O	8.9081233	2.9517793	6.0742250
O	7.3495292	1.5824549	3.9606727
O	6.5536096	1.5642726	6.7986669
N	5.7231435	3.7789849	8.6286697
N	7.6538018	4.8447637	8.7512846
N	8.5682649	3.7098139	2.2423342
N	6.6365068	4.6900237	1.8506292
C	7.3390463	9.5277276	2.5656742
H	7.2891235	10.5822785	2.3066832
H	8.3218970	9.1196208	2.3349542
H	6.5631159	8.9718681	2.0310002
H	6.0608948	10.6628461	8.0768615
C	8.2990269	3.5413313	0.8946367
C	9.7634736	3.2423600	2.9324880
C	7.0695684	4.1637110	0.6414794
C	5.3371306	5.2865787	2.0953657
C	9.0171788	2.9152370	-0.1135313

H	9.7831544	2.1533593	2.8933346
H	10.6331659	3.6834372	2.4514691
H	9.6988551	3.5630616	3.9675153
C	6.5031458	4.1712769	-0.6251953
H	5.3440837	6.3474842	1.8399123
H	4.5987361	4.7629333	1.4881637
H	5.0910289	5.1604642	3.1473338
C	8.4505124	2.9187153	-1.3822799
H	9.9837076	2.4689497	0.0759731
H	5.5601290	4.6628802	-0.8264042
C	7.2160393	3.5317142	-1.6334292
H	8.9831528	2.4493115	-2.1999419
H	6.8138968	3.5199092	-2.6389144
C	6.5018327	0.9876348	3.2328490
C	7.3871689	0.7969296	7.3658790
C	5.1163572	1.2328841	3.2265684
C	7.0624274	-0.0387130	2.2764756
C	8.7837450	0.9399305	7.3286735
C	6.7936948	-0.3352529	8.1737968
C	4.4619806	2.2052438	3.9921972
H	4.5109085	0.6371369	2.5563731
H	7.7671241	-0.6815808	2.8074230
H	6.2832473	-0.6451314	1.8147940
H	7.6157989	0.4784936	1.4869468
C	9.4602663	1.9990332	6.7096014
H	9.3775854	0.2039689	7.8542607
H	6.0583480	-0.8656786	7.5651876
H	7.5506148	-1.0345783	8.5287671
H	6.2680907	0.0796427	9.0394997
C	10.9670675	2.0580729	6.7944474
H	11.2763340	3.0425569	7.1533266
H	11.3795249	1.2850642	7.4426678
H	11.3834832	1.9408852	5.7900321
C	2.9623660	2.3472633	3.8583571
H	2.7136484	3.3851790	3.6230557
H	2.5470386	1.6912052	3.0935043
H	2.4951775	2.1133815	4.8192480
C	6.1676013	3.6529053	9.9353371
C	4.4741907	3.2649747	8.0859245
C	7.3918595	4.3366279	10.0179938
C	5.6183867	3.0118794	11.0361287
H	4.4408230	2.1853335	8.2199857
H	3.6341202	3.7431992	8.5942511
H	4.4577258	3.4807047	7.0190795
C	6.3379890	3.0723762	12.2273030
H	4.6760482	2.4833842	10.9714866
C	7.5588387	3.7494573	12.3089030
H	5.9434045	2.5825462	13.1087551
C	8.1080339	4.3970789	11.2043720
H	8.0906180	3.7725413	13.2518978
H	9.0516222	4.9227895	11.2736140

C	8.9048592	5.4426449	8.3055705
H	8.8719103	6.5296871	8.3917091
H	9.7104879	5.0450292	8.9211340
H	9.0718719	5.1555245	7.2688482
P	10.5446433	6.8931468	1.5653767
F	8.9367413	6.8216972	1.1924116
F	10.1962440	6.1799787	2.9961885
F	10.3028196	8.3451813	2.2554063
F	12.1009309	6.9407887	1.9590949
F	10.8310828	7.5943581	0.1495110
F	10.7414315	5.4238930	0.8941930

[Co(acac)$_2$(L7)]PF$_6$, $^{(3)}$A, ε_r = 1

Energy = -4501.940272009

C	6.6022800	6.9916654	6.6623405
C	7.2166777	6.9672968	3.9101367
C	6.6840425	8.1768163	5.9616484
C	6.9882986	8.1633466	4.5536281
H	6.3327077	6.9901877	7.7098652
H	7.5529317	6.9603018	2.8823993
O	6.4857329	9.3976379	6.4910015
O	7.0228158	9.3708473	3.9813459
C	6.2078011	9.4754738	7.8853493
H	5.2751762	8.9559510	8.1258308
H	7.0298925	9.0493896	8.4685448
C	6.7943687	5.7590757	6.0114714
C	7.1318821	5.7435008	4.6069222
N	6.6108576	4.5565450	6.6134119
N	7.4042623	4.5293432	4.0819955
Co	6.9664448	2.8453688	5.3699670
C	6.6670965	4.3959644	7.9273806
C	7.5261200	4.3318334	2.7599570
O	4.9980250	2.9309586	4.8193986
O	8.9537609	2.8430993	5.9781716
O	7.3184322	1.4422274	3.9497990
O	6.5998152	1.5154194	6.8904126
N	5.7331018	3.7315533	8.6561679
N	7.6987962	4.7309997	8.7593657
N	8.5251695	3.6338776	2.1821944
N	6.6104447	4.6516342	1.8002410
C	7.2445148	9.4302883	2.5608085
H	7.1825845	10.4866654	2.3125085
H	8.2239886	9.0292537	2.3053950
H	6.4580619	8.8744947	2.0419208
H	6.1087636	10.5357141	8.1041384
C	8.2683119	3.5118567	0.8270298
C	9.7169949	3.1422723	2.8628823
C	7.0469200	4.1521168	0.5818153
C	5.3120988	5.2488538	2.0419636

C	8.9915303	2.9119377	-0.1933953
H	9.7541441	2.0581448	2.7561108
H	10.5875336	3.6219054	2.4214661
H	9.6338309	3.3930075	3.9157739
C	6.4898182	4.1992027	-0.6880378
H	5.3113815	6.3004798	1.7500592
H	4.5666208	4.7015815	1.4647927
H	5.0813854	5.1619311	3.1006986
C	8.4355695	2.9570057	-1.4661094
H	9.9532521	2.4531802	-0.0091475
H	5.5514077	4.7024671	-0.8818711
C	7.2067915	3.5846680	-1.7089510
H	8.9722337	2.5088999	-2.2930207
H	6.8130063	3.6050577	-2.7175953
C	6.5074593	1.0692822	3.0538658
C	7.4387176	0.9479076	7.6514046
C	5.1656320	1.4854968	2.9334226
C	7.0514728	0.0963639	2.0329855
C	8.8205458	1.1947906	7.6831836
C	6.8664642	-0.0736351	8.6099134
C	4.4884686	2.3312158	3.8197173
H	4.5907150	1.0590618	2.1218173
H	7.6431572	-0.6667398	2.5412803
H	6.2626527	-0.3740713	1.4460238
H	7.7161983	0.6322792	1.3488832
C	9.4968344	2.0938922	6.8431196
H	9.4142748	0.6199518	8.3816384
H	6.2321727	-0.7692286	8.0565019
H	7.6415796	-0.6258877	9.1410616
H	6.2358680	0.4345861	9.3455719
C	11.0024618	2.1912849	6.9445804
H	11.3060962	3.2402360	6.9411848
H	11.3947472	1.6919143	7.8309158
H	11.4447141	1.7287252	6.0572838
C	3.0077107	2.5611072	3.6119146
H	2.7947621	3.6325237	3.6382221
H	2.6474817	2.1382377	2.6739100
H	2.4580335	2.1023226	4.4388916
C	6.1741265	3.6133552	9.9651144
C	4.4776912	3.2305496	8.1145080
C	7.4229863	4.2521254	10.0339429
C	5.6036332	3.0134260	11.0783221
H	4.4107890	2.1595182	8.2967461
H	3.6434329	3.7534269	8.5871008
H	4.4829421	3.3982905	7.0388827
C	6.3289803	3.0662687	12.2660795
H	4.6414666	2.5205681	11.0253643
C	7.5754840	3.6971283	12.3331954
H	5.9187499	2.6068273	13.1566914
C	8.1454194	4.3047520	11.2168158
H	8.1107305	3.7149714	13.2743327

H	9.1093516	4.7937095	11.2736097
C	8.9586164	5.3048277	8.3106712
H	8.9757035	6.3835890	8.4738913
H	9.7683841	4.8315723	8.8645555
H	9.0760830	5.0875931	7.2509830
P	10.4918286	6.8551097	1.6115039
F	8.8926020	6.7800536	1.2006375
F	10.1200240	6.0950844	3.0118240
F	10.2177240	8.2827895	2.3413578
F	12.0380640	6.9075701	2.0408286
F	10.8006698	7.6043221	0.2252081
F	10.7173110	5.4101765	0.9003388

[Co(acac)$_2$(L7)]PF$_6$, ^{1}A, ε_r = 1

Energy = -4501.952132591

C	5.6905580	6.8113362	6.5398639
C	6.8771491	6.8669874	4.0182290
C	5.8047378	8.0195603	5.8662949
C	6.4237698	8.0468736	4.5951559
H	5.2421910	6.7615566	7.5234293
H	7.4322448	6.8988907	3.0921385
O	5.3661706	9.2192121	6.3470560
O	6.5299522	9.2669093	4.0169987
C	4.7983447	9.2175028	7.6439194
H	3.9019120	8.5875904	7.6858580
H	5.5197334	8.8688840	8.3925075
C	6.1642285	5.6336434	5.9564128
C	6.7156635	5.6448841	4.6831604
N	6.1601640	4.3951158	6.6597355
N	7.2110135	4.4034542	4.2459107
Co	6.5055308	2.9418443	5.3673638
C	6.8765111	4.3772574	7.7798792
C	7.5341841	4.2042822	2.9683243
O	4.7904513	3.3037088	4.6760373
O	8.2158299	2.6664247	6.1519783
O	6.9715690	1.6176356	4.0940050
O	5.6629912	1.5565574	6.3623890
N	6.5515679	3.6225425	8.8749234
N	8.0358349	5.0448828	8.0652182
N	8.6145703	3.4954236	2.5469184
N	6.8536409	4.6207348	1.8525496
C	7.1972670	9.3342446	2.7543491
H	7.1901623	10.3880275	2.4839843
H	8.2214031	8.9671607	2.8286706
H	6.6498793	8.7581622	1.9998083
H	4.5283227	10.2512001	7.8512035
C	8.6410541	3.4683177	1.1639571
C	9.6567898	2.9554207	3.4036791
C	7.5299524	4.1957605	0.7193897

C	5.5344094	5.2228086	1.8312381
C	9.5359187	2.8941909	0.2732987
H	9.5865697	1.8667971	3.4207815
H	10.6188392	3.2921404	3.0244333
H	9.4957984	3.3454304	4.4015904
C	7.2638984	4.3666038	-0.6310428
H	5.5918924	6.2602037	1.4984812
H	4.9025022	4.6487169	1.1511367
H	5.1203137	5.1946926	2.8338106
C	9.2721667	3.0649983	-1.0823715
H	10.4122015	2.3610787	0.6168007
H	6.4124069	4.9388472	-0.9762999
C	8.1554344	3.7818196	-1.5265633
H	9.9549570	2.6446243	-1.8102737
H	7.9876087	3.8962815	-2.5903573
C	6.2001842	1.2266592	3.1587808
C	6.2971127	0.6265335	6.9568117
C	4.8985660	1.6832259	2.9380705
C	6.8056898	0.2101605	2.2281494
C	7.6703700	0.6124512	7.2108290
C	5.4387496	-0.5236744	7.4161642
C	4.2690899	2.6684510	3.7069722
H	4.3429237	1.2492994	2.1187380
H	7.3379814	-0.5464466	2.8067128
H	6.0520319	-0.2625550	1.5996119
H	7.5338246	0.7112258	1.5835777
C	8.5370577	1.6471207	6.8428985
H	8.0790396	-0.2207612	7.7648209
H	5.1260710	-1.0974079	6.5390389
H	5.9714043	-1.1845516	8.0991144
H	4.5331639	-0.1444232	7.8931276
C	9.9694098	1.6276453	7.3049097
H	10.1078817	2.4027293	8.0640346
H	10.2434134	0.6647001	7.7337635
H	10.6297226	1.8659064	6.4694839
C	2.8428194	3.0500258	3.4071165
H	2.7405095	4.1362501	3.4213091
H	2.5092551	2.6553295	2.4480445
H	2.2001789	2.6515545	4.1971258
C	7.5139227	3.7973856	9.8574598
C	5.2755481	2.9587520	9.0632040
C	8.4498104	4.7111384	9.3471768
C	7.6421893	3.2519513	11.1260733
H	5.4288126	1.8939147	9.2382060
H	4.7628531	3.3982477	9.9220596
H	4.6959142	3.0983860	8.1544567
C	8.7504175	3.6441384	11.8744876
H	6.9172892	2.5496192	11.5174652
C	9.6896272	4.5455313	11.3643305
H	8.8869926	3.2407779	12.8701486
C	9.5551687	5.0984953	10.0924381

H	10.5419299	4.8239969	11.9716152
H	10.2822503	5.8014973	9.7075452
C	8.8479698	5.7684140	7.0832876
H	8.4569608	6.7716412	6.9216514
H	9.8636828	5.8226037	7.4681277
H	8.8534637	5.2209677	6.1450546
P	10.7223834	6.7011977	2.8385480
F	9.3212678	6.5951655	1.9829627
F	10.0240781	5.8067428	4.0231414
F	10.1669283	8.0446459	3.5597800
F	12.0801825	6.7718769	3.6999633
F	11.3732971	7.5750858	1.6592363
F	11.2290399	5.3276770	2.1243944

3.2.4 Optimized coordinates by BLYP calculations

Calculations without counterion and without solvent effect

[Co(acac)$_2$(L7)]$^+$, ^{5}A, ε_r = 1

Energy = -3560.095450382

C	6.6803188	7.1383787	6.7648865
C	7.4610454	7.1385413	4.0552308
C	6.8708551	8.3475005	6.1016684
C	7.2702722	8.3475818	4.7185332
H	6.3455751	7.1288635	7.7972641
H	7.7957407	7.1291766	3.0228369
O	6.7020800	9.5785597	6.6537111
O	7.4388429	9.5787102	4.1665809
C	6.2974172	9.6566350	8.0333290
H	5.3167565	9.1830547	8.1830657
H	7.0430333	9.1842005	8.6889717
C	6.8658421	5.9067267	6.0972409
C	7.2758093	5.9067978	4.7227903
N	6.6061475	4.6703788	6.6451355
N	7.5360267	4.6705198	4.1749528
Co	7.0709201	3.0181059	5.4100015
C	6.5429073	4.4462500	7.9624942
C	7.5991792	4.4463292	2.8576090
O	5.1186694	2.9879452	4.7527477
O	9.0231267	2.9874159	6.0673302
O	7.4997806	1.6314552	3.9681089
O	6.6417110	1.6313262	6.8516809
N	5.5305747	3.7656312	8.5872312
N	7.5293739	4.6894305	8.8974530
N	8.6114957	3.7656510	2.2329017
N	6.6126980	4.6894492	1.9226282
C	7.8435405	9.6569587	2.7869835
H	7.9092570	10.7238932	2.5633958
H	8.8243039	9.1835900	2.6372508

H	7.0980451	9.1844142	2.1312828
H	6.2314901	10.7235413	8.2569896
C	8.2663214	3.5417699	0.8973309
C	9.8462267	3.3191481	2.8808801
C	6.9996831	4.1372941	0.6943993
C	5.2951752	5.2448135	2.2347854
C	8.9345809	2.8789734	-0.1324120
H	9.8962226	2.2265871	2.8488537
H	10.7087161	3.7566715	2.3646865
H	9.8203086	3.6315632	3.9265544
C	6.3633748	4.0874010	-0.5460800
H	5.2642763	6.3224906	2.0360208
H	4.5484209	4.7353744	1.6187120
H	5.0776407	5.0546524	3.2882220
C	8.2935929	2.8263859	-1.3767193
H	9.9072244	2.4198190	0.0231045
H	5.3919563	4.5463794	-0.7100926
C	7.0326410	3.4180604	-1.5788821
H	8.7819818	2.3177202	-2.2043471
H	6.5667706	3.3568134	-2.5594325
C	6.6749848	0.9361708	3.2798350
C	7.4663576	0.9355945	7.5396743
C	5.2727642	1.1035885	3.2885297
C	7.2926623	-0.1061908	2.3658078
C	8.8686279	1.1026171	7.5309329
C	6.8484449	-0.1068081	8.4534983
C	4.5764575	2.0932924	4.0057249
H	4.6877533	0.4345866	2.6644370
H	8.0491037	-0.6775844	2.9170357
H	6.5471246	-0.7898398	1.9481663
H	7.8020375	0.4016085	1.5341543
C	9.5651525	2.0923866	6.8140421
H	9.4534942	0.4332700	8.1547909
H	6.0918603	-0.6779092	7.9021634
H	7.5938226	-0.7907213	8.8709923
H	6.3391970	0.4009429	9.2852604
C	11.0785179	2.1574771	6.9156006
H	11.3910556	3.1783418	7.1707619
H	11.4811682	1.4574612	7.6540441
H	11.5150272	1.9236680	5.9345549
C	3.0631177	2.1588392	3.9040542
H	2.7508816	3.1798879	3.6492604
H	2.6603409	1.4592156	3.1653082
H	2.6264596	1.9247718	4.8849717
C	5.8757037	3.5419086	9.9228416
C	4.2958559	3.3190840	7.9392557
C	7.1424053	4.1373254	10.1257100
C	5.2072354	2.8795668	10.9527427
H	4.2458207	2.2265305	7.9714056
H	3.4333635	3.7567007	8.4553654
H	4.3218233	3.6313779	6.8935461

C	5.8479413	2.8275870	12.1972190
H	4.2346739	2.4202472	10.7972024
C	7.1089315	3.4192005	12.3993315
H	5.3592743	2.3195008	13.0250388
C	7.7785437	4.0878257	11.3662922
H	7.5745347	3.3585147	13.3800439
H	8.7501293	4.5464942	11.5301831
C	8.8467755	5.2450672	8.5852583
H	8.8773939	6.3227895	8.7838213
H	9.5936309	4.7359342	9.2014602
H	9.0643920	5.0547506	7.5318624

$[Co(acac)_2(L7)]^+$, ^3A, $\varepsilon_r = 1$

Energy = -3560.100240933

C	-0.2271764	3.0973824	1.3773163
C	0.4084730	3.0040282	-1.3657062
C	-0.0093245	4.2813474	0.6797983
C	0.3196883	4.2338482	-0.7205785
H	-0.5063853	3.1257370	2.4254516
H	0.6809713	2.9575097	-2.4153383
O	-0.0864818	5.5307465	1.2127527
O	0.5262599	5.4434412	-1.3079231
C	-0.4215714	5.6555624	2.6071355
H	-1.4162992	5.2350171	2.8123528
H	0.3294925	5.1585975	3.2381204
C	-0.1408393	1.8411845	0.7315929
C	0.1848905	1.7971211	-0.6642678
N	-0.4387982	0.6335828	1.3166444
N	0.3057615	0.5432324	-1.2291168
Co	0.0331952	-1.0261479	-0.0264150
C	-0.4445071	0.4074742	2.6242165
C	0.2675275	0.3473370	-2.5585402
O	-1.8283414	-1.1210046	-0.5650330
O	1.8798580	-0.8969815	0.4769592
O	0.5882462	-2.5119179	-1.4598981
O	-0.3861792	-2.4181175	1.2561969
N	-1.4070942	-0.3394298	3.2672061
N	0.5400430	0.7041592	3.5564070
N	1.2418543	-0.2800383	-3.2875276
N	-0.7869132	0.6171986	-3.4032384
C	0.8639942	5.4713993	-2.7066594
H	0.9782348	6.5281715	-2.9575560
H	1.8077801	4.9397312	-2.8946503
H	0.0609710	5.0295817	-3.3143745
H	-0.4237286	6.7284997	2.8109868
C	0.8007369	-0.4480538	-4.6026927
C	2.5534967	-0.6792927	-2.7702130
C	-0.4868595	0.1324075	-4.6809102
C	-2.0641353	1.1938854	-2.9847611

C	1.4009298	-1.0453704	-5.7121310
H	2.6467699	-1.7678071	-2.8128187
H	3.3366191	-0.2001561	-3.3691152
H	2.6184967	-0.3708525	-1.7255027
C	-1.2147711	0.1318647	-5.8717612
H	-2.1147968	2.2570323	-3.2475209
H	-2.8743726	0.6517788	-3.4816124
H	-2.1605627	1.0724548	-1.9046321
C	0.6690806	-1.0475805	-6.9053791
H	2.3894472	-1.4928518	-5.6529419
H	-2.2034337	0.5781980	-5.9385188
C	-0.6139334	-0.4715789	-6.9829732
H	1.1012695	-1.5046890	-7.7922153
H	-1.1508005	-0.4945809	-7.9282302
C	-0.2506592	-3.1067798	-2.2058770
C	0.4382628	-2.8992101	2.1113226
C	-1.6495156	-2.8635990	-2.2197208
C	0.3123434	-4.1316956	-3.1790993
C	1.7945989	-2.5302736	2.2263547
C	-0.1382787	-3.9241051	3.0677834
C	-2.3385433	-1.9486547	-1.4167005
H	-2.2486708	-3.4676188	-2.8947603
H	1.1486771	-4.6633610	-2.7122831
H	-0.4438820	-4.8491862	-3.5141036
H	0.6979984	-3.6092549	-4.0671512
C	2.4420509	-1.6041312	1.3981778
H	2.3919509	-3.0401248	2.9759663
H	-0.9261250	-4.4978753	2.5688354
H	0.6319012	-4.6031717	3.4475477
H	-0.5841369	-3.4067552	3.9296772
C	3.9356966	-1.3849010	1.5338779
H	4.1683288	-0.3144561	1.4849478
H	4.3293018	-1.8066725	2.4637699
H	4.4484098	-1.8686963	0.6906144
C	-3.8535438	-1.8819317	-1.5001972
H	-4.1868028	-0.8373778	-1.5295347
H	-4.2427846	-2.4173238	-2.3714799
H	-4.2826368	-2.3330967	-0.5945054
C	-1.0340397	-0.5400341	4.5982686
C	-2.6490773	-0.8076537	2.6511350
C	0.2022293	0.1205865	4.7839543
C	-1.6530709	-1.2330584	5.6383347
H	-2.7098091	-1.8973329	2.7325180
H	-3.5077802	-0.3460568	3.1539462
H	-2.6305245	-0.5422997	1.5927935
C	-0.9904877	-1.2555870	6.8739370
H	-2.6060636	-1.7366134	5.4988772
C	0.2432812	-0.6063141	7.0562375
H	-1.4423095	-1.7862005	7.7085477
C	0.8616874	0.0970024	6.0122714
H	0.7283228	-0.6460579	8.0285763

H	1.8119921	0.6032586	6.1608021
C	1.8395445	1.2947436	3.2438445
H	1.8943188	2.3334008	3.5912748
H	2.6221145	0.7043131	3.7316983
H	1.9898457	1.2656675	2.1639259

[Co(acac)$_2$(L7)]$^+$, ^{1}A, ε_r = 1

Energy = -3560.115339644

C	-0.4777218	2.9582767	1.3156967
C	0.4776510	2.9583112	-1.3155954
C	-0.2495159	4.1743226	0.6676890
C	0.2495859	4.1743368	-0.6674967
H	-0.8712288	2.9464644	2.3272345
H	0.8710836	2.9465621	-2.3271632
O	-0.4651887	5.4087106	1.2213405
O	0.4653439	5.4087368	-1.2210878
C	-0.9924893	5.4629953	2.5542273
H	-1.9781385	4.9772425	2.6140460
H	-0.3070833	4.9909739	3.2750914
C	-0.2224760	1.7335079	0.6647153
C	0.2222972	1.7335273	-0.6646762
N	-0.4659918	0.4688497	1.2434877
N	0.4657815	0.4689320	-1.2435044
Co	-0.0000767	-1.0247593	-0.0000574
C	-0.4396352	0.2816665	2.5600127
C	0.4396686	0.2817518	-2.5600260
O	-1.8215971	-0.9565969	-0.6252152
O	1.8214199	-0.9566125	0.6251531
O	0.5701310	-2.4196690	-1.1998555
O	-0.5702515	-2.4196991	1.1997317
N	-1.3748540	-0.4535406	3.2590073
N	0.5039929	0.7218860	3.4747175
N	1.3749558	-0.4534064	-3.2589452
N	-0.5039379	0.7219237	-3.4748184
C	0.9926269	5.4630698	-2.5539779
H	1.0923332	6.5258608	-2.7874837
H	1.9782424	4.9772540	-2.6138466
H	0.3071730	4.9911459	-3.2748609
H	-1.0921248	6.5257772	2.7878032
C	1.0165615	-0.5066447	-4.6079005
C	2.6499710	-0.9100322	-2.7037530
C	-0.1660185	0.2568141	-4.7500673
C	-1.7543465	1.3961226	-3.1277966
C	1.6169436	-1.1310649	-5.7014212
H	2.7055377	-2.0019067	-2.7192773
H	3.4700467	-0.4854517	-3.2953828
H	2.7057902	-0.5732797	-1.6668841
C	-0.7846911	0.4133140	-5.9907495
H	-1.6873869	2.4721744	-3.3253569

H	-2.5638463	0.9614468	-3.7230621
H	-1.9551593	1.2372165	-2.0674921
C	0.9945614	-0.9737604	-6.9473513
H	2.5293706	-1.7121421	-5.5951064
H	-1.6906893	1.0023401	-6.1073825
C	-0.1852429	-0.2204667	-7.0878279
H	1.4347516	-1.4431100	-7.8238206
H	-0.6416534	-0.1236272	-8.0699877
C	-0.2094981	-3.0037684	-2.0382276
C	0.2093703	-3.0038103	2.0380903
C	-1.5703102	-2.7135504	-2.2326631
C	0.4558688	-4.0606089	-2.8962729
C	1.5701919	-2.7136148	2.2325350
C	-0.4559756	-4.0606759	2.8961246
C	-2.2970842	-1.7443013	-1.5238761
H	-2.1044282	-3.2978542	-2.9753356
H	1.2609661	-4.5476970	-2.3367627
H	-0.2633443	-4.8099584	-3.2415674
H	0.8943342	-3.5829523	-3.7844898
C	2.2969498	-1.7443459	1.5237742
H	2.1043062	-3.2979484	2.9751858
H	-1.2610899	-4.5477572	2.3366340
H	0.2632545	-4.8100179	3.2413952
H	-0.8944235	-3.5830492	3.7843673
C	3.7809125	-1.5810048	1.7830523
H	4.0564136	-0.5210453	1.7542072
H	4.0788849	-2.0181796	2.7409130
H	4.3410044	-2.0870683	0.9839466
C	-3.7810249	-1.5809093	-1.7832830
H	-4.0565121	-0.5209519	-1.7542807
H	-4.0788965	-2.0179191	-2.7412496
H	-4.3412023	-2.0871141	-0.9843285
C	-1.0164021	-0.5066933	4.6079360
C	-2.6499322	-0.9101010	2.7039094
C	0.1661557	0.2568177	4.7500212
C	-1.6167139	-1.1310591	5.7015319
H	-2.7055369	-2.0019705	2.7193539
H	-3.4699401	-0.4855454	3.2956512
H	-2.7058463	-0.5732465	1.6670780
C	-0.9944044	-0.9734684	6.9474600
H	-2.5289674	-1.7124127	5.5952451
C	0.1853065	-0.2200174	7.0878673
H	-1.4345732	-1.4427322	7.8239869
C	0.7846976	0.4136964	5.9907148
H	0.6417157	-0.1230348	8.0700133
H	1.6904462	1.0031066	6.1073440
C	1.7543724	1.3961407	3.1276772
H	1.6874186	2.4721698	3.3253491
H	2.5638942	0.9614178	3.7228851
H	1.9551670	1.2372915	2.0673636

Calculations with inclusion of the counterion

[Co(acac)$_2$(L7)]PF$_6$, ^{5}A, ε_r = 1

Energy = -4501.170674563

C	6.5697740	7.1400523	6.6730761
C	7.2876812	7.1037203	3.9387098
C	6.6832812	8.3352987	5.9703832
C	7.0358394	8.3160009	4.5728648
H	6.2595755	7.1390966	7.7132894
H	7.6504310	7.0874276	2.9165882
O	6.4677333	9.5738458	6.5008080
O	7.0886964	9.5330186	3.9820097
C	6.1334050	9.6608094	7.8935790
H	5.1849912	9.1458643	8.1081086
H	6.9316265	9.2354068	8.5200680
C	6.7842441	5.9041728	6.0263270
C	7.1693416	5.8836090	4.6423082
N	6.5602246	4.6779918	6.6186899
N	7.4500716	4.6400836	4.1292250
Co	6.9886371	3.0079640	5.3824757
C	6.6233292	4.4749581	7.9292833
C	7.5716866	4.3996960	2.8104995
O	5.0032472	3.0254558	4.7915502
O	8.9303426	2.9604197	6.0698540
O	7.3520135	1.5888995	3.9565433
O	6.5581128	1.5772833	6.8256658
N	5.6996889	3.7527700	8.6492441
N	7.6679447	4.7885711	8.7828888
N	8.5835949	3.6862393	2.2398541
N	6.6268919	4.6537498	1.8385333
C	7.3400680	9.5871393	2.5529730
H	7.2813530	10.6480422	2.2985521
H	8.3283642	9.1865046	2.3102828
H	6.5634624	9.0274815	2.0126088
H	6.0291225	10.7281259	8.1038592
C	8.3022541	3.4848025	0.8897495
C	9.8020790	3.2431659	2.9211329
C	7.0588195	4.1025351	0.6296456
C	5.3227894	5.2631439	2.0723521
C	9.0165928	2.8358121	-0.1160809
H	9.8140956	2.1484424	2.9472852
H	10.6636808	3.6473478	2.3855519
H	9.7823238	3.6244908	3.9410536
C	6.4850325	4.0815182	-0.6413708
H	5.3344934	6.3261694	1.8037457
H	4.5778157	4.7362510	1.4672662
H	5.0671018	5.1547247	3.1283251
C	8.4402977	2.8099349	-1.3903261
H	9.9902304	2.3956409	0.0771132

H	5.5369400	4.5709745	-0.8486583
C	7.1967313	3.4188368	-1.6484435
H	8.9732112	2.3231081	-2.2039260
H	6.7865282	3.3873561	-2.6553770
C	6.5166133	1.0383234	3.1639636
C	7.3915796	0.8589659	7.4735284
C	5.1307166	1.3153378	3.1203005
C	7.1012614	0.0447080	2.1741849
C	8.7945538	1.0203061	7.4561926
C	6.7858537	-0.2187751	8.3603838
C	4.4587815	2.2577304	3.9202166
H	4.5382580	0.7641544	2.3956263
H	7.8208425	-0.6047844	2.6866423
H	6.3307506	-0.5647842	1.6906728
H	7.6465587	0.5960664	1.3951344
C	9.4819364	2.0416946	6.7735141
H	9.3856541	0.3269409	8.0476718
H	6.0153295	-0.7636641	7.8016264
H	7.5360560	-0.9225904	8.7356319
H	6.2972347	0.2578079	9.2226891
C	10.9968770	2.1117975	6.8639389
H	11.3041119	3.1189308	7.1739514
H	11.4120583	1.3727196	7.5567685
H	11.4232287	1.9439259	5.8654241
C	2.9554492	2.4256038	3.7572326
H	2.7213318	3.4805749	3.5629264
H	2.5479049	1.8085860	2.9498262
H	2.4579346	2.1532504	4.6983004
C	6.1637032	3.5716552	9.9520250
C	4.4265506	3.2689722	8.1147258
C	7.4100544	4.2385063	10.0430296
C	5.6183676	2.8955618	11.0428105
H	4.4011637	2.1765636	8.1624084
H	3.5997416	3.6991760	8.6936117
H	4.3603594	3.5651092	7.0653674
C	6.3575311	2.9002044	12.2346559
H	4.6633269	2.3816575	10.9708086
C	7.5957070	3.5597452	12.3250635
H	5.9639134	2.3815151	13.1058005
C	8.1426607	4.2439129	11.2295105
H	8.1431504	3.5413297	13.2646615
H	9.0985966	4.7554582	11.3065626
C	8.9284412	5.3933735	8.3472447
H	8.8994200	6.4844290	8.4488790
H	9.7378726	4.9856350	8.9595552
H	9.1026704	5.1252299	7.3022200
P	10.5034550	6.8750270	1.4528721
F	8.8558762	6.7721904	1.1395227
F	10.2132616	6.1852342	2.9364034
F	10.2669734	8.3638844	2.1238063
F	12.0951029	6.9505931	1.7900650

F	10.7279030	7.5509186	-0.0115672
F	10.6962203	5.3678592	0.8018502

[Co(acac)$_2$(L7)]PF$_6$, ^{3}A, ε_r = 1

Energy = -4501.175698317

C	6.6532477	7.1008716	6.7011689
C	7.3337108	7.0073862	3.9601993
C	6.7887534	8.2821336	5.9813391
C	7.1187252	8.2340940	4.5789438
H	6.3595260	7.1211304	7.7457374
H	7.6798951	6.9706813	2.9327305
O	6.6137580	9.5333362	6.4973030
O	7.1831808	9.4417051	3.9691191
C	6.3011268	9.6474610	7.8930157
H	5.3429068	9.1601233	8.1275507
H	7.0966696	9.2096899	8.5143986
C	6.8194526	5.8480815	6.0696325
C	7.1847568	5.7992029	4.6808104
N	6.5562144	4.6430476	6.6793668
N	7.3997058	4.5417915	4.1578787
Co	7.0249475	2.9808874	5.3446584
C	6.6244408	4.4248466	7.9773114
C	7.4972224	4.3257389	2.8253782
O	5.1718333	2.9876622	4.7646679
O	8.8526418	3.0350294	5.9329259
O	7.5370151	1.4496043	3.9383839
O	6.5127343	1.5763358	6.5935847
N	5.7097029	3.6589309	8.6774070
N	7.6522468	4.7433540	8.8601226
N	8.5035489	3.6434209	2.2109419
N	6.5399352	4.6213572	1.8801175
C	7.3736595	9.4715013	2.5306620
H	7.3155108	10.5291029	2.2623236
H	8.3459097	9.0570533	2.2513010
H	6.5681567	8.9126140	2.0331392
H	6.2274713	10.7198355	8.0902356
C	8.1966684	3.4897724	0.8599715
C	9.7501867	3.1987443	2.8425333
C	6.9499536	4.1172872	0.6450399
C	5.2537800	5.2551045	2.1452176
C	8.8935258	2.8797892	-0.1826693
H	9.7498922	2.1060465	2.8971715
H	10.5844847	3.5832736	2.2531471
H	9.7857343	3.6057249	3.8512439
C	6.3541700	4.1478048	-0.6163529
H	5.2737256	6.3089387	1.8432969
H	4.4781131	4.7229703	1.5847819
H	5.0395720	5.1837353	3.2123936
C	8.2958355	2.9051041	-1.4457359

H	9.8703636	2.4335338	-0.0239198
H	5.4029783	4.6449725	-0.7876130
C	7.0481053	3.5250679	-1.6591791
H	8.8141111	2.4507880	-2.2870664
H	6.6216227	3.5327577	-2.6597780
C	6.7104864	0.9817464	3.0991682
C	7.2993571	1.0305378	7.4447937
C	5.3387913	1.3456836	3.0075882
C	7.2541604	-0.0262135	2.0956103
C	8.6640659	1.3375620	7.6083401
C	6.6597725	-0.0060989	8.3515900
C	4.6658420	2.2559277	3.8296302
H	4.7454853	0.8417201	2.2498536
H	8.0159547	-0.6496926	2.5765192
H	6.4675315	-0.6590300	1.6702395
H	7.7352525	0.5185879	1.2703603
C	9.3644112	2.2800614	6.8390031
H	9.2212699	0.7804859	8.3557709
H	5.8598525	-0.5264016	7.8140678
H	7.3932191	-0.7321249	8.7183719
H	6.2186210	0.4940217	9.2259995
C	10.8593218	2.4511616	7.0311139
H	11.1294263	3.5119675	6.9776846
H	11.2024886	2.0251455	7.9796561
H	11.3865386	1.9429308	6.2113167
C	3.1661196	2.4400669	3.6569667
H	2.9201038	3.5086453	3.6202075
H	2.7907556	1.9464465	2.7549912
H	2.6483609	2.0179955	4.5296827
C	6.1575318	3.4706449	9.9839412
C	4.4363204	3.1963997	8.1284110
C	7.3903708	4.1583645	10.1023197
C	5.6095214	2.7719054	11.0586658
H	4.3624748	2.1093409	8.2279229
H	3.6053664	3.6784290	8.6600174
H	4.4123314	3.4460367	7.0655668
C	6.3353294	2.7678344	12.2607231
H	4.6607994	2.2488327	10.9697360
C	7.5620982	3.4415197	12.3755646
H	5.9374242	2.2313517	13.1190953
C	8.1102929	4.1528846	11.2958640
H	8.0995819	3.4163159	13.3207738
H	9.0566509	4.6790270	11.3918414
C	8.9102801	5.3774983	8.4712834
H	8.9153423	6.4424174	8.7334991
H	9.7336111	4.8689346	8.9830540
H	9.0406254	5.2715931	7.3927978
P	10.4913599	6.7962569	1.3584211
F	8.8325353	6.7069927	1.1016170
F	10.2445457	6.1065451	2.8494300
F	10.2926363	8.2854791	2.0382577

F	12.0941321	6.8555694	1.6402038
F	10.6704275	7.4717297	-0.1124894
F	10.6484855	5.2880593	0.6981009

[Co(acac)$_2$(L7)]PF$_6$, ^{1}A, ε_r = 1

Energy = -4501.188256578

C	5.6699070	6.8521702	6.5389392
C	6.8582205	6.8981879	4.0007449
C	5.7813654	8.0655471	5.8575341
C	6.4053453	8.0883715	4.5764044
H	5.2234193	6.8031366	7.5274219
H	7.4110769	6.9254049	3.0703546
O	5.3380741	9.2787830	6.3344316
O	6.5102392	9.3170313	3.9896028
C	4.7528700	9.2976865	7.6389143
H	3.8500736	8.6679687	7.6858652
H	5.4684043	8.9599900	8.4056758
C	6.1521733	5.6655664	5.9604253
C	6.7009053	5.6707767	4.6728545
N	6.1596416	4.4316424	6.6813731
N	7.1907707	4.4244924	4.2248879
Co	6.4861896	2.9294136	5.3732686
C	6.8781493	4.3979749	7.8024643
C	7.5239636	4.2084823	2.9493945
O	4.7387446	3.2900763	4.6782332
O	8.2331672	2.6597089	6.1601555
O	6.9553280	1.5813055	4.0708481
O	5.6417624	1.5128058	6.3862144
N	6.5592046	3.6115492	8.8971254
N	8.0621768	5.0535924	8.0987159
N	8.6177674	3.4863972	2.5348940
N	6.8417494	4.6068894	1.8084597
C	7.1849654	9.3964313	2.7139942
H	7.1724885	10.4576289	2.4517171
H	8.2154002	9.0322885	2.7854760
H	6.6390209	8.8222494	1.9497443
H	4.4816008	10.3402851	7.8271279
C	8.6493923	3.4391793	1.1441629
C	9.6654650	2.9443184	3.3981049
C	7.5305561	4.1647201	0.6792008
C	5.5141539	5.2090650	1.7642000
C	9.5524151	2.8505175	0.2595562
H	9.5811522	1.8523842	3.4354459
H	10.6333911	3.2627374	3.0047480
H	9.5258498	3.3503654	4.3966188
C	7.2734823	4.3160638	-0.6829628
H	5.5700120	6.2408170	1.3982972
H	4.8791975	4.6134957	1.0976884
H	5.0895391	5.2137181	2.7665512

C	9.2964753	3.0027251	-1.1089361
H	10.4301824	2.3199279	0.6169318
H	6.4202042	4.8853629	-1.0426142
C	8.1758056	3.7168546	-1.5721738
H	9.9874880	2.5704365	-1.8291319
H	8.0123354	3.8175484	-2.6428490
C	6.1878838	1.2335579	3.1023135
C	6.2944942	0.6104363	7.0241040
C	4.8879053	1.7173002	2.8764231
C	6.8034772	0.2455147	2.1322200
C	7.6705388	0.6365480	7.3043582
C	5.4496163	-0.5545260	7.5041861
C	4.2360737	2.6783052	3.6701178
H	4.3438846	1.3146970	2.0277410
H	7.3573931	-0.5226127	2.6838950
H	6.0484146	-0.2256522	1.4953289
H	7.5193282	0.7757001	1.4882494
C	8.5451088	1.6637811	6.9057900
H	8.0816500	-0.1730306	7.8991297
H	5.1773107	-1.1754987	6.6393488
H	5.9808812	-1.1773472	8.2307105
H	4.5149537	-0.1853483	7.9430858
C	9.9773543	1.6727656	7.3995422
H	10.0882373	2.4549457	8.1634937
H	10.2636244	0.7131983	7.8407401
H	10.6553310	1.9211867	6.5749042
C	2.8044123	3.0672703	3.3552891
H	2.6974680	4.1577550	3.3916173
H	2.4843988	2.6949340	2.3771931
H	2.1426955	2.6494188	4.1264567
C	7.5452565	3.7483151	9.8740820
C	5.2730633	2.9532078	9.0998863
C	8.4926004	4.6732465	9.3729995
C	7.6894524	3.1590982	11.1292630
H	5.4214297	1.8838853	9.2806190
H	4.7647670	3.3994054	9.9649260
H	4.6789581	3.0856809	8.1946110
C	8.8218388	3.5130812	11.8776741
H	6.9585700	2.4505219	11.5106650
C	9.7698638	4.4230278	11.3783322
H	8.9688691	3.0727370	12.8612428
C	9.6195512	5.0220610	10.1191303
H	10.6406057	4.6715516	11.9808856
H	10.3541885	5.7291659	9.7437423
C	8.8766421	5.8021938	7.1248908
H	8.4595610	6.7979359	6.9522114
H	9.8875977	5.8922667	7.5268095
H	8.9217494	5.2596038	6.1802667
P	10.6978748	6.7110470	2.8691356
F	9.2796738	6.5354804	1.9989353
F	10.0161676	5.8061305	4.0918048

F	10.0826681	8.0699470	3.5711145
F	12.0685006	6.8500037	3.7427444
F	11.3261806	7.5943369	1.6543605
F	11.2593644	5.3192199	2.1753104

3.3 Calculated UV-vis spectra for $[Co(acac)_2(L7)]$ and $[Co(acac)_2(L7)]^+$

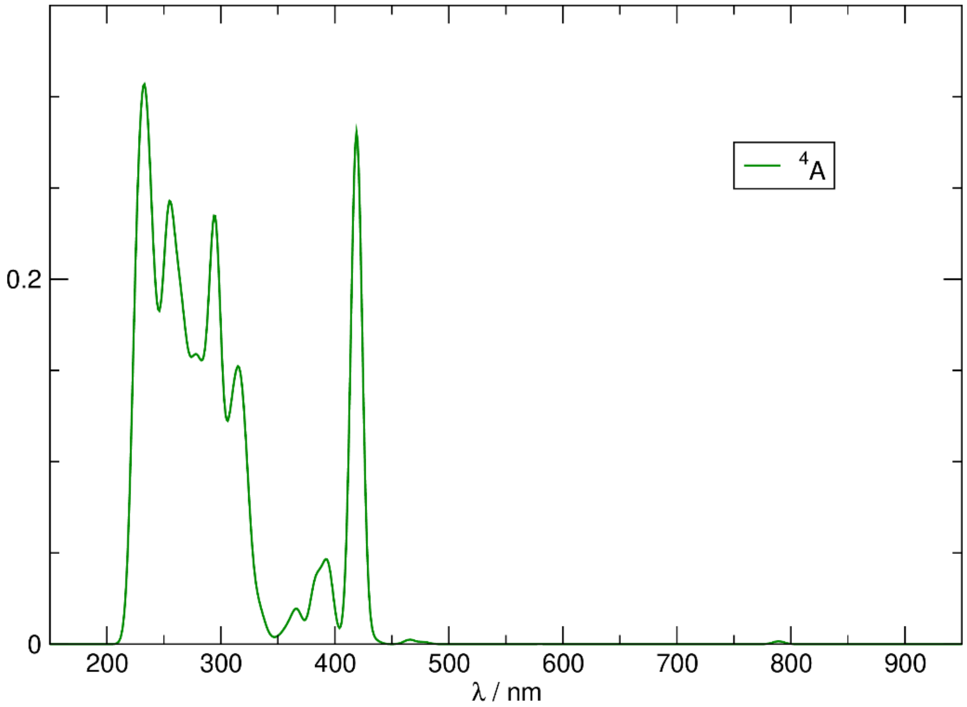

UV-vis spectrum of $[Co(acac)_2(L7)]$ by TDDFT calculations with the B3LYP functional and the def2-TZVP basis set.

Leading contributions of lower-lying transitions of the UV-vis spectrum of $[Co(acac)_2(L7)]$

State	λ/nm	leading contribution	weight
^4A	789.2	β-185a → β-196a	29.0 %
	478.2	α-189a → α-190a	44.0 %
	465.6	β-186a → β-187a	92.6 %
	434.2	β-186a → β-189a	51.7 %
	419.1	β-186a → β-188a	50.4 %

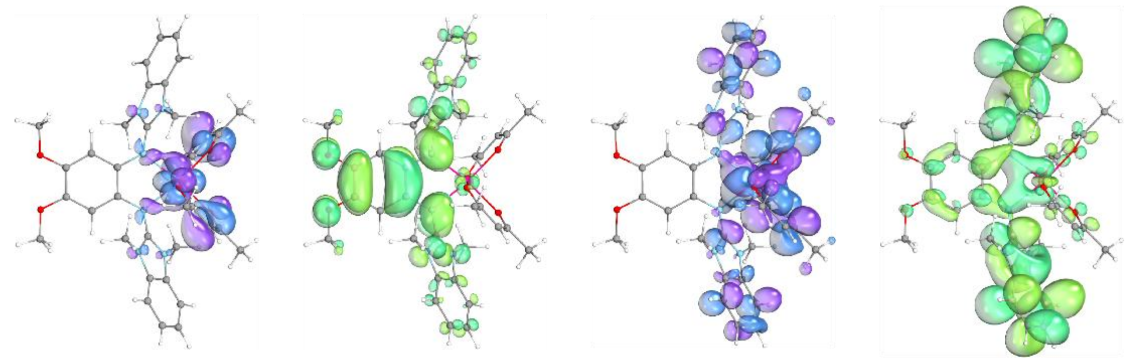

β-185a β-186a β-HOMO β-187a β-LUMO β-188a

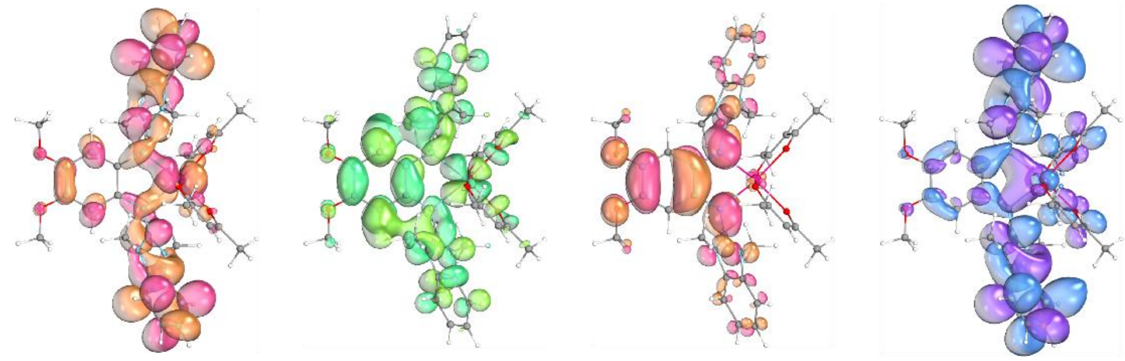

β-189a β-196a α-189a α-HOMO α-190a α-LUMO

Isosurfaces of the orbitals that are involved in the lower-lying transitions of the ^{4}A state of [Co(acac)$_2$(L7)].

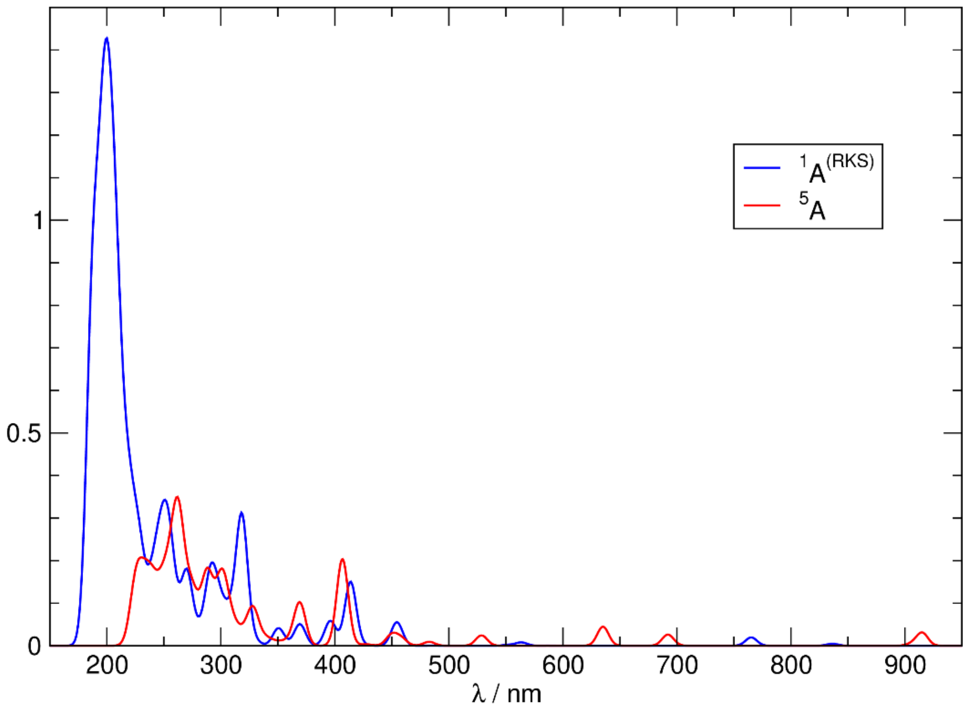

UV-vis spectra of [Co(acac)$_2$(L7)]$^+$ by TDDFT calculations with the B3LYP functional and the def2-TZVP basis set.

Leading contributions of lower-lying transitions of the UV-vis spectra of two different states of $[Co(acac)_2(L7)]^+$

State	λ/nm	leading contribution	weight
^1A	836.4	187a → 188a	92.5 %
	765.2	187a → 189a	80.6 %
	563.6	174a → 188a	46.2 %
	454.4	186a → 188a	41.4 %
	413.8	187a → 192a	90.2 %
^5A	915.3	β-185a → β-186a	95.0 %
	691.8	β-183a → β-186a	93.9 %
	635.2	β-181a → β-186a	84.6 %
	528.6	β-180a → β-186a	96.5 %
	482.7	β-179a → β-186a	96.6 %
	458.3	β-178a → β-186a	68.4 %
	449.8	β-175a → β-186a	47.3 %
	406.6	α-189a → α-190a	92.6 %

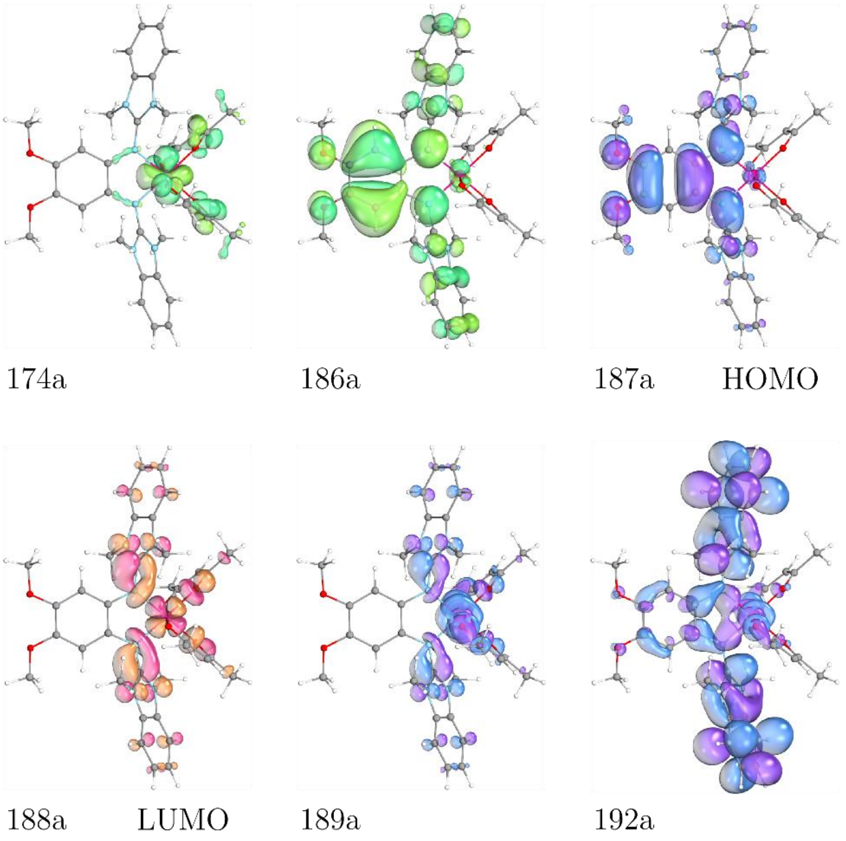

Isosurfaces of the orbitals that are involved in the lower-lying transitions of the ^1A state of $[Co(acac)_2(L7)]^+$.

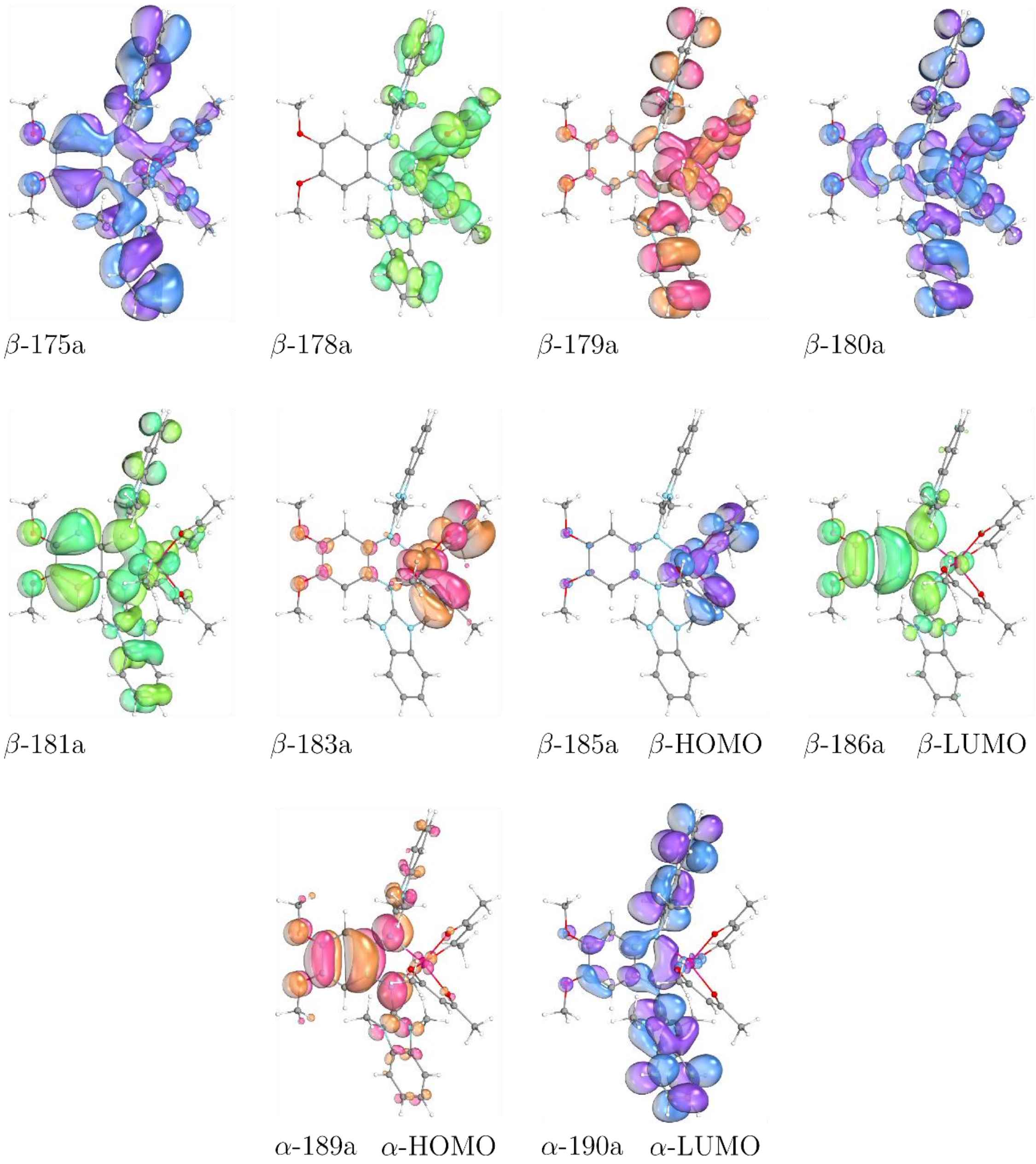

Isosurfaces of the orbitals that are involved in the low-lying transitions of the ^5A state of $[Co(acac)_2(L7)]^+$.

3.4 Calculated vibrational spectra of [Co(acac)$_2$(L7)] and[Co(acac)$_2$(L7)]$^+$

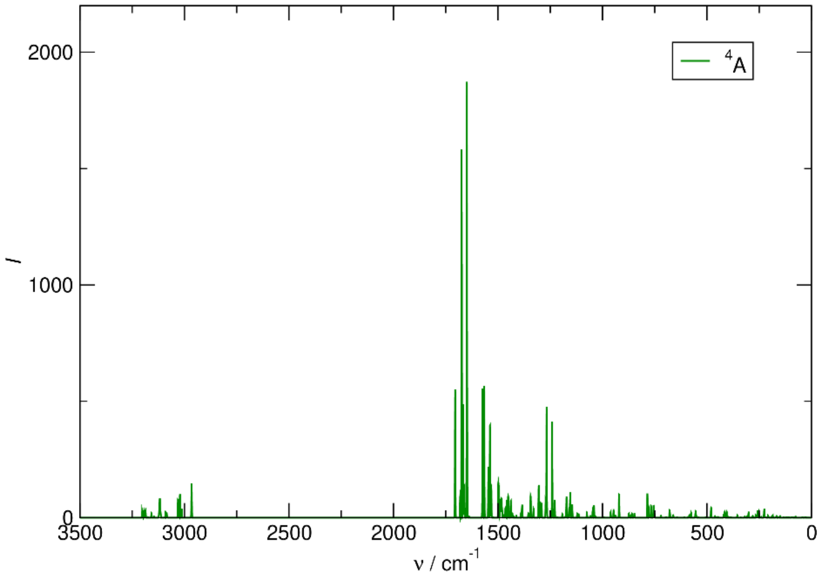

IR spectrum of [Co(acac)$_2$(L7)] by density functional calculations with the B3LYP functional and the def2-SV(P) basis set.

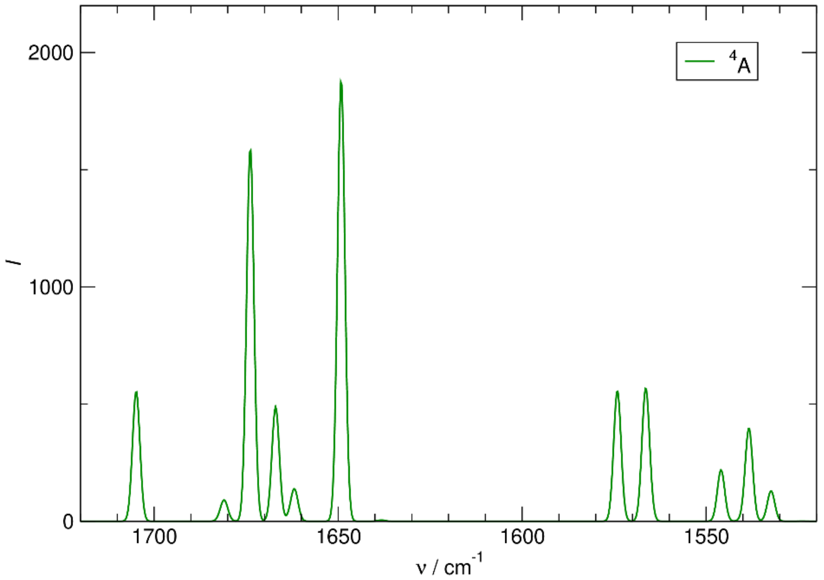

Detail of the IR spectrum of [Co(acac)$_2$(L7)] by density functional calculations with the B3LYP functional and the def2-SV(P) basis set.

Among the vibrational modes of [Co(acac)$_2$(L7)], the modes with quanta of 1704.9, 1673.9 and 1649.2 cm^{-1} show large contributions of the C=N stretching motion. The symmetric combination of the C=N stretching vibrations contributes to the band at 1704.9 cm^{-1}, whereas the antisymmetric combination contributes to the bands at 1673.9 and 1649.2 cm^{-1}.

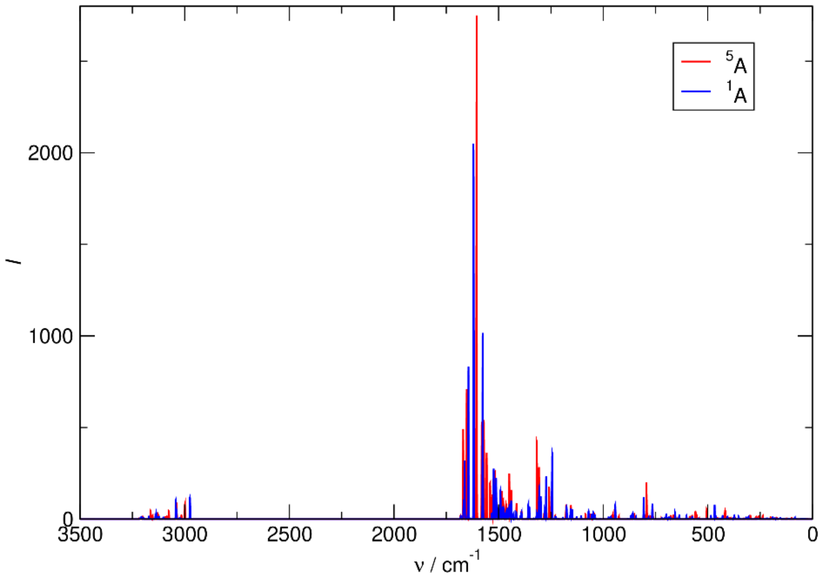

IR spectra of $[Co(acac)_2(L7)]^+$ by density functional calculations with the B3LYP functional and the def2-SV(P) basis set.

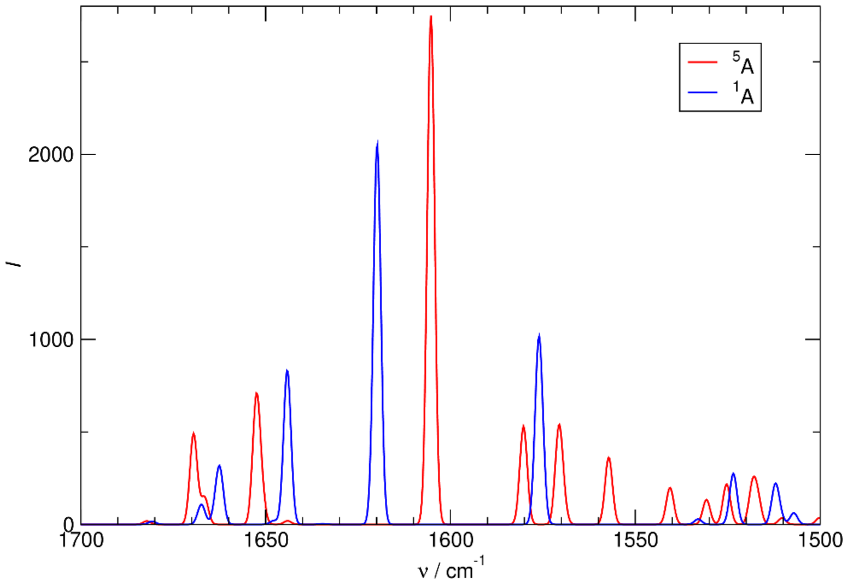

Detail of the IR spectra of $[Co(acac)_2(L7)]^+$ by density functional calculations with the B3LYP functional and the def2-SV(P) basis set.

Among the vibrational modes of the ^1A and ^5A states of $[Co(acac)_2(L7)]^+$, the modes with quanta of 1619.8 and 1634.6 cm^{-1} (^1A) and of 1605.3 and 1644.1 cm^{-1} (^5A) exhibit large contributions of the C=N stretching vibrations. The symmetric combination contributes essentially to the weak bands at 1634.6 and 1644.1 cm^{-1}, and the antisymmetric combination to the strong bands at 1619.8 and 1605.3 cm^{-1}. The (symmetric) C=N stretching vibration is also involved in the modes with quanta of 1662.4 and 1667.4 cm^{-1} (^1A) and of 1669.5 cm^{-1} (^5A).
